# Purely Covalent Molecular Cages and Containers for Guest Encapsulation

**DOI:** 10.1021/acs.chemrev.2c00198

**Published:** 2022-07-22

**Authors:** Giovanni Montà-González, Félix Sancenón, Ramón Martínez-Máñez, Vicente Martí-Centelles

**Affiliations:** † Instituto Interuniversitario de Investigación de Reconocimiento Molecular y Desarrollo Tecnológico (IDM) Universitat Politècnica de València, Universitat de València. Camino de Vera, s/n 46022, Valencia, Spain; ‡ CIBER de Bioingeniería, Biomateriales y Nanomedicina, Instituto de Salud Carlos III, 28029 Madrid, Spain; § Centro de Investigación Príncipe Felipe, Unidad Mixta UPV-CIPF de Investigación de Mecanismos de Enfermedades y Nanomedicina, Valencia, Universitat Politècnica de València, 46012 Valencia, Spain; ∥ Instituto de Investigación Sanitaria la Fe, Unidad Mixta de Investigación en Nanomedicina y Sensores, Universitat Politènica de València, 46026 València, Spain; ⊥ Departamento de Química, Universitat Politècnica de València, 46022 Valencia, Spain

## Abstract

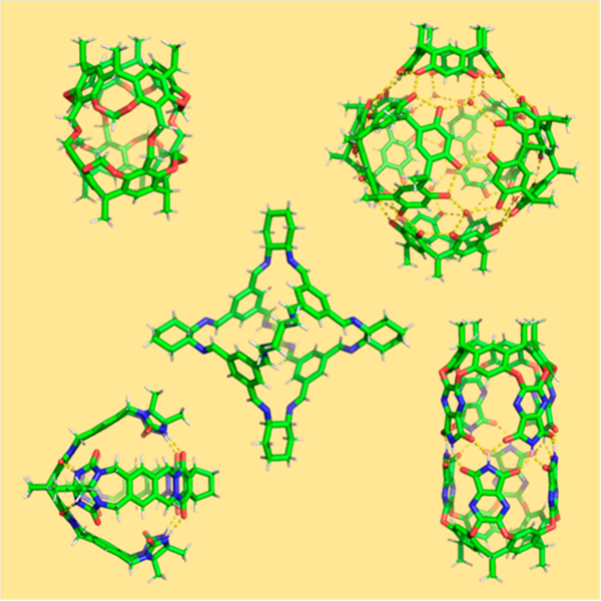

Cage compounds offer unique binding pockets similar to enzyme-binding sites, which can be customized in terms of size, shape, and functional groups to point toward the cavity and many other parameters. Different synthetic strategies have been developed to create a toolkit of methods that allow preparing tailor-made organic cages for a number of distinct applications, such as gas separation, molecular recognition, molecular encapsulation, hosts for catalysis, etc. These examples show the versatility and high selectivity that can be achieved using cages, which is impossible by employing other molecular systems. This review explores the progress made in the field of fully organic molecular cages and containers by focusing on the properties of the cavity and their application to encapsulate guests.

## 1 Introduction

Molecular encapsulation is a key step for many processes found in Nature. For example, endocytosis is a crucial process for cells to internalize macromolecules and particles using transport vesicles that isolate a substance from surroundings by encapsulation.^1^ Moreover, enzyme-binding pockets formed by amino acid residues result in selective binding and catalytic performance.^2^ Nonpolar residues in pockets provide a hydrophobic environment to the binding cavity, which also contains buried polar and charged units to minimize solvation from water molecules.^3^ Supramolecular architectures, particularly molecular cages, provide synthetic cavities to effectively bind guest molecules by multiple noncovalent interactions with the cage-building blocks surrounding the cavity.^4,5^ The inclusion of guest molecules in suitable host-cage derivatives results in restricted systems that confer entrapped substrates novel properties. With their discovery, the possibility of preparing cage structures with specific cavities has drawn the attention of supramolecular chemists, who have developed numerous systems for the encapsulation of guest molecules for different applications, such as stabilization of species,^6^ gas separation,^7^ catalysis,^8−10^ among many others.^11,12^

**Figure 1:**
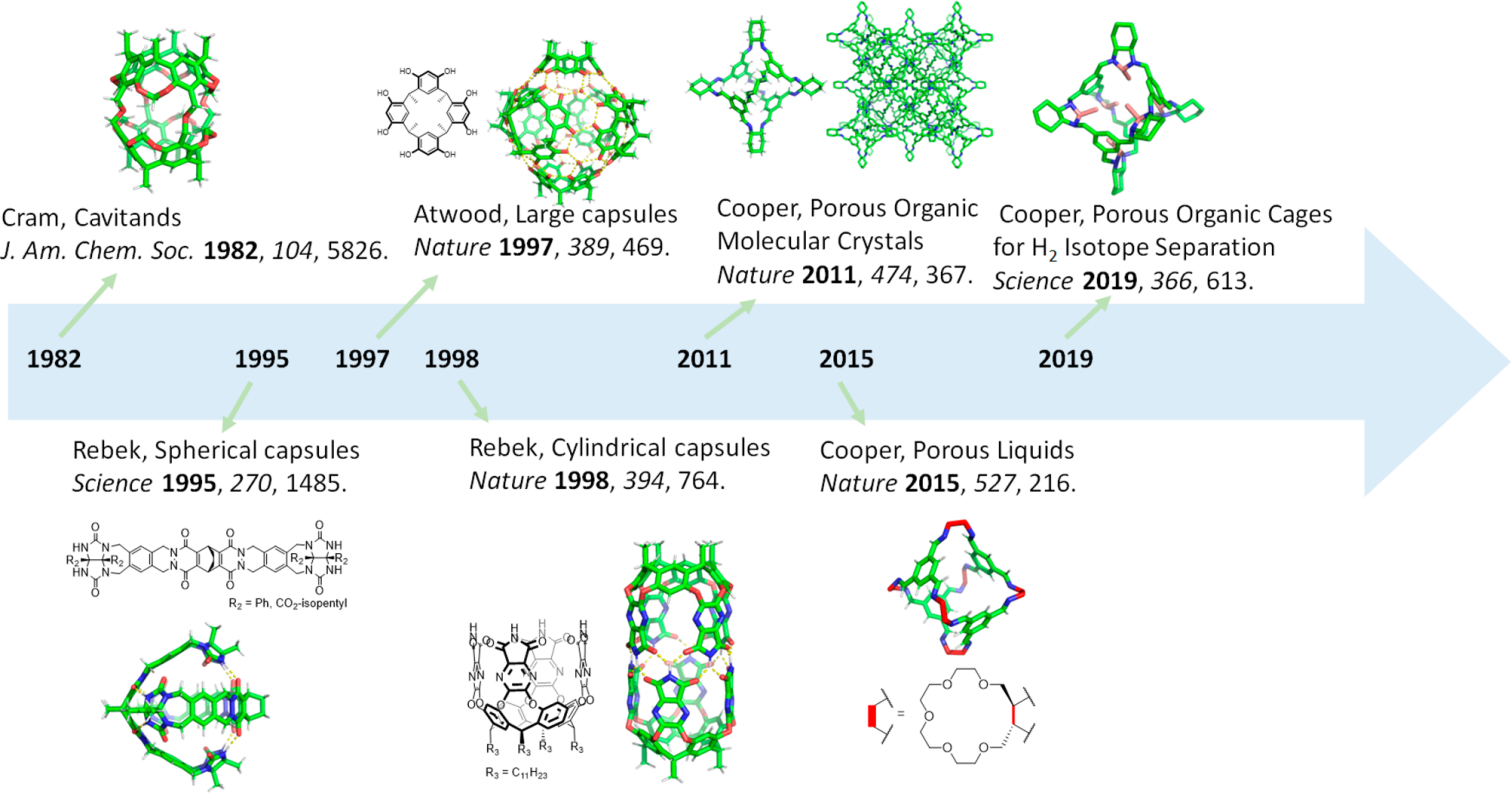
Timeline showing major advances in organic cages.

Sometimes cage compounds offer a unique binding pocket compared to enzyme-binding sites, which can be tailored in terms of the size, shape, and functional groups pointing toward the cavity, and many other parameters by customizing the structure of the cage. In this review, we explore and analyze the properties of different families of fully organic cages and containers by focusing on the properties of the cavity, including information like cavity size and volume, wherever possible, and encapsulated guests as well as main applications (Figure 1). For this purpose, we divide the review into different sections that group distinct families of cages by their properties or the methodology employed for their synthesis. The first section describes the general strategies widely used to prepare organic cages, including irreversible bonds, reversible bonds, and templates. After this, the review includes two sections that group cages according to their solubility in organic solvents and their solubility in water.

Despite the wide scope of this area, most of the reviewed work in the field has to date referred to very specific cage families and applications. We intend to herein provide a broader overview by emphasizing the synthesis and properties of the cage cavity for the encapsulation of guest molecules. In addition, we have attempted to bring together different families of molecules with a central cavity, including hemicarcerands, carcerands, cryptands, cavitands, capsules, and cages. This approach aims to provide a general conceptual view of this area. We hope that the compiled examples provide a comprehensive guide to understand the field that fuels the development of novel molecular structures with new or enhanced properties and applications.

### 1.1 General Considerations

The nomenclature of the cage compounds that we describe below follows the convention adopted in the literature.^13,14^ As such, we consider hemicarcerands, carcerands, cryptands, cavitands, capsules, and cages. Whenever appropriate, the size of the cavity (volume and diameter) described in the original reference is included. For those cages that do not have this information available, we prepared molecular models using the wave function Spartan software and determined the volume and diameter of the cavity using pywindow.^15,16^

### 1.2 Scope of the Review

This review explores different families of purely organic cages. The reported examples reveal the wide diversity of cage systems that can be prepared by using building blocks of different shapes and sizes.^17^ We highlight distinct possible synthetic protocols to prepare organic cages, the properties of the cavity of the cage, and their applications. The review does not include, in general, cages with small cavities to encapsulate inorganic anions, and the reader can find information elsewhere.^18^ The review is not focused on coordination cages containing metals and organic ligands. In this area, the reader can find seminal works from Fujita, Stang, Nitschke, Raymond, and others in comprehensive reviews covering these families of cages.^19−23^

The examples included in the review are mainly from the past decade, with a large number of examples from the last five years. Literature search has been included up to the first semester of 2021.

## 2 General Concepts in Organic Cage and Container Synthesis

This section describes the main strategies used to prepare molecular cages, including examples that explore the effect of different reaction conditions on the outcome of the cage formation reaction. We also include representative examples that show the effect of the preorganization and rigidity of building blocks on the cage formation reaction, which highlights the possible geometries and variety of cages that can be obtained. This section also includes a description of the main water-solubilizing groups used to obtain water-soluble cages that are further explained in detail in the examples described in Section 4. The last part of this section includes the molecular modeling strategies adopted to simulate and predict the properties of molecular systems, including the porosity and the outcome of the cage self-assembly reaction from a set of building blocks.

### 2.1 Main Synthetic Strategies

Cage synthesis involves the assembly of convergent building blocks to form a hollow structure with a central cavity. The design of cage-building blocks is a key issue for successful cage synthesis. The most important factors are the shape, conformational preferences, and the reactive functional groups that will link together the building blocks within the cage framework. This section describes the main synthetic strategies used to prepare organic cages and is divided into two main categories depending on the bond reaction used to assemble the cage from building blocks, i.e., irreversible and reversible bond formation. Whereas the first examples of cages were obtained using irreversible bonds, for instance S_N_2 reactions to form C–O bonds, most recent cage formation methods are based on reversible bonds. In fact, the recent advances made in the field of covalent molecular cages can be attributed to important progress in dynamic covalent chemistry, allowing the synthesis of cage compounds in a few steps and usually with good yields.^24^ Most of the cages described in the literature based on reversible bonds contain imine bonds built up from amine- and aldehyde-containing blocks. Other also widely employed reactions for the construction of cages based on reversible bonds employ hydrogen, chalcogen, boronate ester, and disulfide bonds.

The main synthetic advantage of reversible bond formation in the synthesis of cages is the self-correction of the mistakes made in the multistep self-assembly reaction pathway, which finally allow obtaining of a thermodynamic more stable cage product.^25,26^ In contrast, irreversible bonds usually result in low cage formation yields because they normally favor oligomers or polymers instead of cages. While self-assembly kinetics of metal coordination organic cages has been extensively studied,^27^ related studies for purely organic cages remain mainly unexplored with relatively very little available information.

A key aspect in the cage formation kinetics is the effective molarity (EM) of the reactive groups, which can be increased by employing templates. These concepts have been widely used in the synthesis of macrocycles,^28^ and similar conceptual ideas have been followed for the formation of cages. EM is a key factor that defines the cooperativity of self-assembled systems; in particular, the product of the equilibrium constant *K* of the building block self-assembly reaction by EM determines the extent to which the self-assembled structure is formed. If product *K*·EM > 1, the cooperative intramolecular interaction between the self-assembled structure building blocks is favored, and this structure will be the main self-assembled product. These concepts are valid for any self-assembled structure held together by cooperative intramolecular interactions regardless of its architecture, including molecular cages.^29^

#### 2.1.1 Assembly through Irreversible Bonds

**Figure 2:**
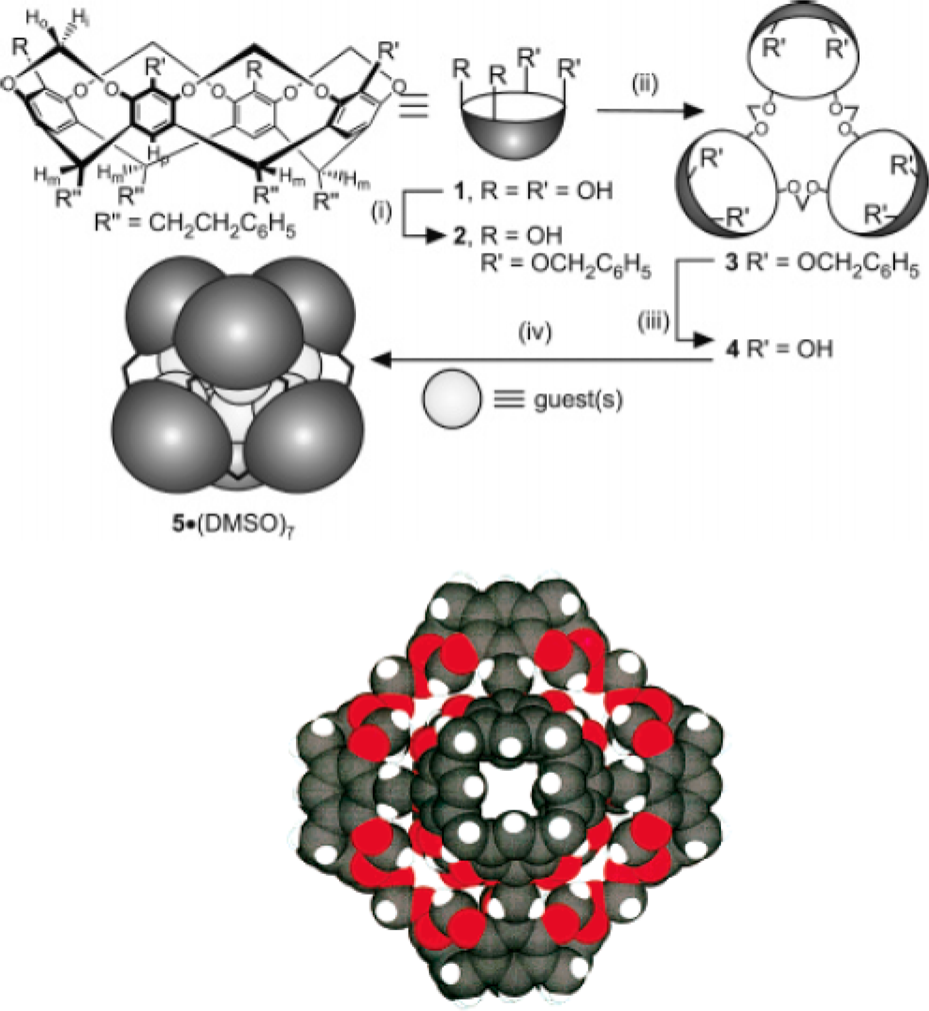
Synthesis of the six-bowl carceplex **5** and a simplified structure with [4] cavitand phenylethyl groups replaced with hydrogens.^31^ Adapted with permission from ref (31). Copyright 2005 American Chemical Society.

Despite the versatility and the large number of irreversible organic synthesis reactions, their applications in the synthesis of organic cages are quite limited. As stated above, irreversible bonds do not allow the mistakes made during cage formation to be corrected, which limits the synthesis of large cage structures because cage assembly process efficiency decreases with the required number of bonds to be formed during cage assembly. Back in 1969, Lehn, Sauvage, and Dietrich reported one of the first examples of molecular cages (i.e., macrobicycles) by irreversible bond-forming reactions. They obtained overall yields of ca. 25%.^30^ However, when cage complexity increases, the formation of large assemblies through irreversible bonds becomes inefficient and requires additional protection/deprotection steps to minimize side reactions. This is exemplified, for instance, in the reported carceplex synthesis by Sherman and co-workers. Synthesis involves four steps: transforming the starting material **1** into intermediates **2**–**4** with yields of 26%, 16%, 58%, and 35%, which results in an overall carceplex **5** formation yield of 0.8%. A low overall yield limits the preparation of large quantities (Figure 2).^31^

**Figure 3:**
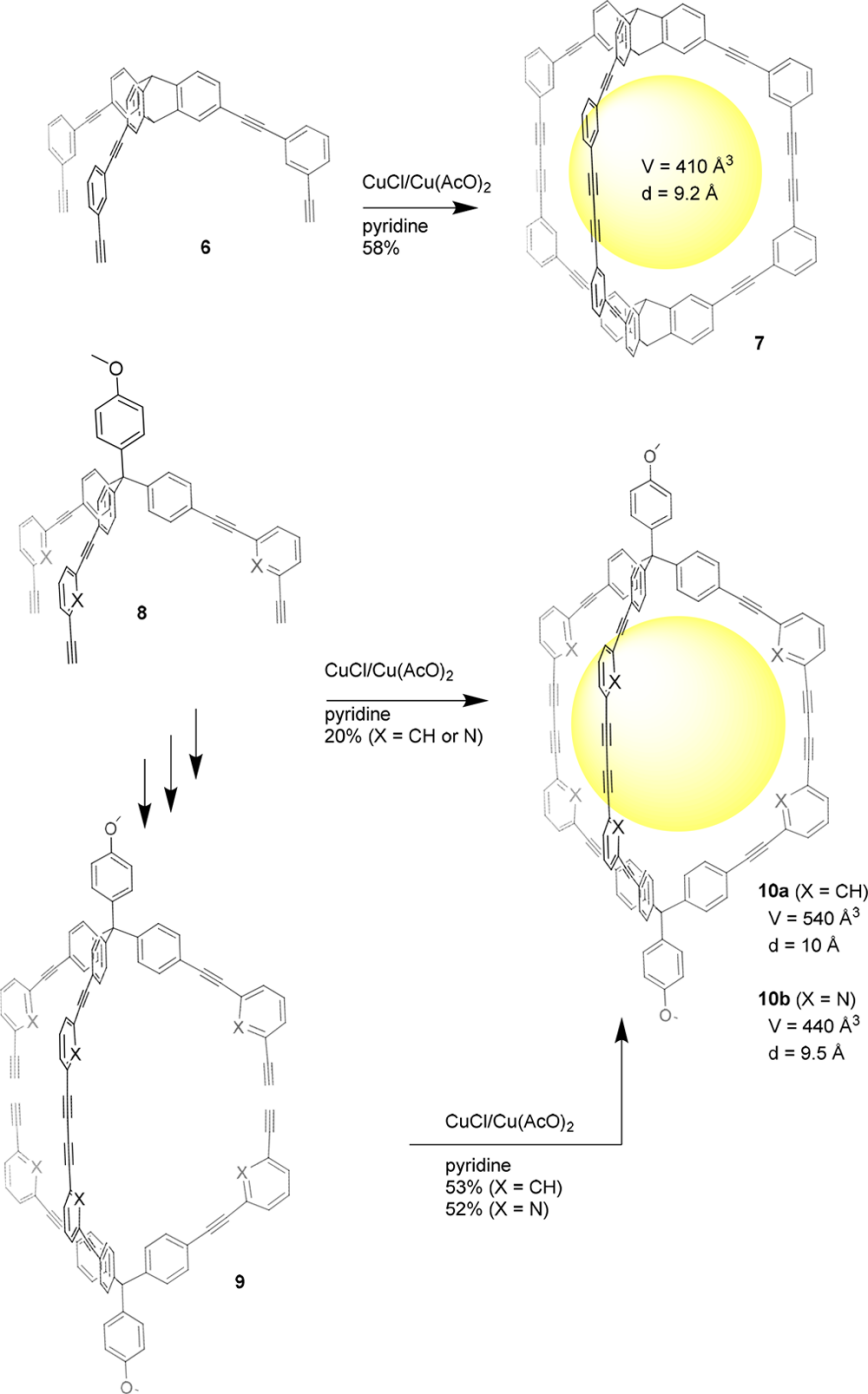
Synthesis of trigonal-prismatic cage **7** (top) and endohedrally functionalized analogues **10** (bottom).^32−34^

For smaller assemblies that involve the assembly of two building blocks, overall cage formation yields are much higher and allow, for instance, the use of carbon–carbon formation reactions to prepare different cage structures. Chen and co-workers proved the efficient use of Eglington–Glaser coupling for cage synthesis from precursor **6** by reporting the synthesis of an all-carbon triptycene-based molecular cage **7** with a 58% yield (Figure 3 top).^32^ Following a similar approach, Doonan, Sumby, and co-workers prepared precursor **8**, which allowed the synthesis of the organic cage **10**. Whereas cage synthesis using the same synthetic strategy from precursor **8** has only a 20% yield, that of synthesis form intermediate **9** is 52–53% (Figure 3 bottom).^33,34^ Cage **10a** has a modeled structure with a cavity shape of a distorted triangular prism and internal vertical and horizontal diameters of 13.5 and 12 Å, respectively. The authors found that the gas adsorption properties of this material very much depended on the crystallization polymorph. Slow crystallization yielded **10aα**, which is nonporous to N_2_ gas. In contrast, rapid precipitation led to the polymorph **10aβ**, which displayed a remarkably large BET surface area of 1153 m^2^/g, which is rare for molecular cages. The calculated pore size distributions showed two voids: one with a diameter of 11 Å associated with the internal cavity and another with a diameter of 6 Å associated with extrinsic voids. Six-pyridine functionalization (cage **10b**) brings about cage structure distortion that results in different packing and gas absorption performance and is nonporous to N_2_. The authors predicted that the precise control of the internal functionality of the cage cavity would help in tuning gas absorption performance.

**Figure 4:**
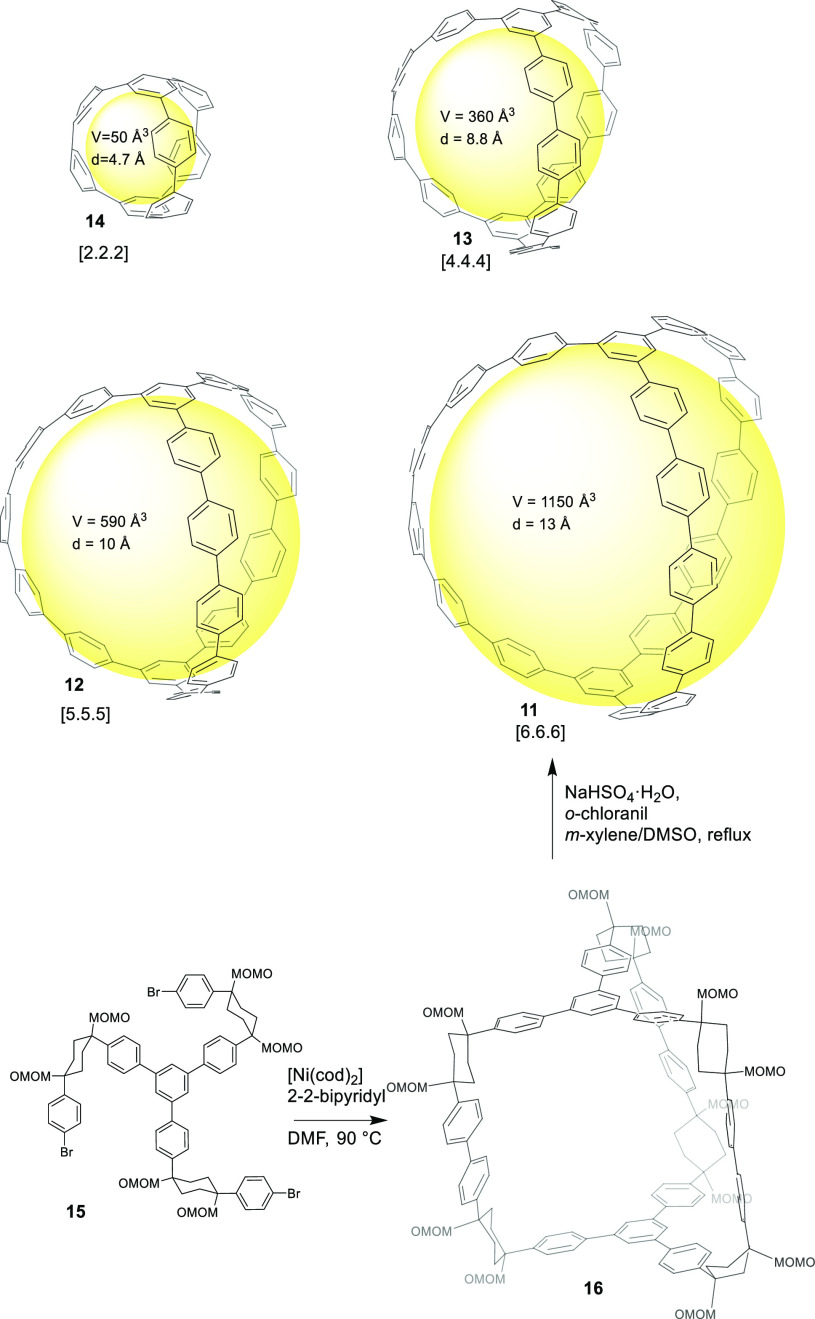
Structures of [n.n.n] carbon nanocages as segments branched carbon nanotubes. Synthetic route for [6.6.6] cage **11**.^35−37^

The formation of carbon cages with strained structures is also possible using building blocks with the appropriate geometry. Itami, Kamada, and co-workers reported the first synthesis of an all-benzene [6.6.6] carbon nanocage **11**, which resembled the junction unit of branched carbon nanotubes. These structures can be obtained by means of specific synthetic protocols that involve L-shaped *cis*-diphenylcyclohexane units and linear phenylene groups coupled using Suzuki–Miyaura coupling or nickel-mediated homocouplings to afford the nonstrained cage intermediate that is subsequently transformed into the strained cage structure (Figure 4).^35,36^ By this methodology, it was possible to prepare carbon nanocages **12** [5.5.5],^36^**13** [4.4.4],^36^ and **14** [2.2.2].^37^ The synthesis of cage **11** from precursor **15** through strained cage intermediate **16** is described in Figure 4.^35^ The absorption wavelength of cages are red-shifted by increasing cage size as a result of a combination of factors associated with the special HOMO and LUMO properties of these fully conjugated systems.

**Figure 5:**
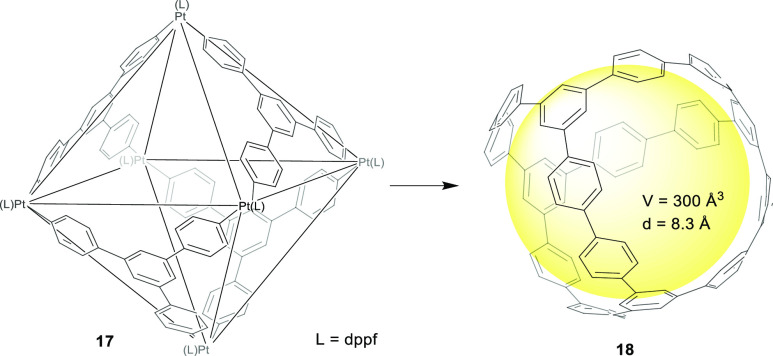
Synthesis of cage **18** from a hexanuclear platinum octahedral cage **17**.^38^ dppf = 1,1′-bis(diphenylphosphino)ferrocene.

The success of the above method is based on the cage formation step from building block precursors with the appropriate geometry to yielding a strain-free intermediate cage. Yamago and co-workers used cage precursor **17** to synthesize carbon nanocage **18** by a different strategy but one also based on preparing an intermediate strain-free cage that was later transformed into the final cage product. The developed method was based on employing a hexanuclear platinum octahedral cage **17** consisting of four building blocks of a stannylated trisubstituted benzene derivative. The reductive elimination of platinum yielded the target carbon nanocage **18** with a 19% yield (Figure 5).^38^

**Figure 6:**
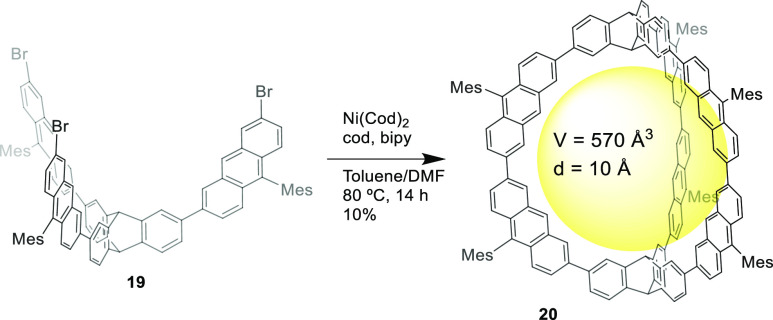
Synthesis of fully aromatic hydrocarbon cage **20** that features two triptycene bridge units.^39^

Toyota and co-workers prepared from precursor **19** a fully aromatic hydrocarbon cage **20** that features two triptycene bridge units using Suzuki–Miyaura coupling in the cage-formation step with a 10% yield. Cage **20** has an approximately spherical and rigid cavity surrounded by multiple hydrogen atoms pointing toward the cavity with good complementarity for fullerenes. In particular, the cage encapsulates guests C_60_ or C_70_ in toluene with association constants of 1.3 × 10^4^ M^–1^ and 3.3 × 10^5^ M^–1^, respectively. The selectivity toward C_70_ over C_60_ defined as *K*_assoc_(C_70_)/*K*_assoc_(C_60_) is 25, which highlights the better size, shape, and electronic properties of C_70_ to be coordinated in the cage cavity. These authors also did DFT calculations, which showed that several CH···π contacts played an important role in complex formation and predicted higher interaction energy with C_70_ compared to C_60_ (Figure 6).^39^

**Figure 7:**
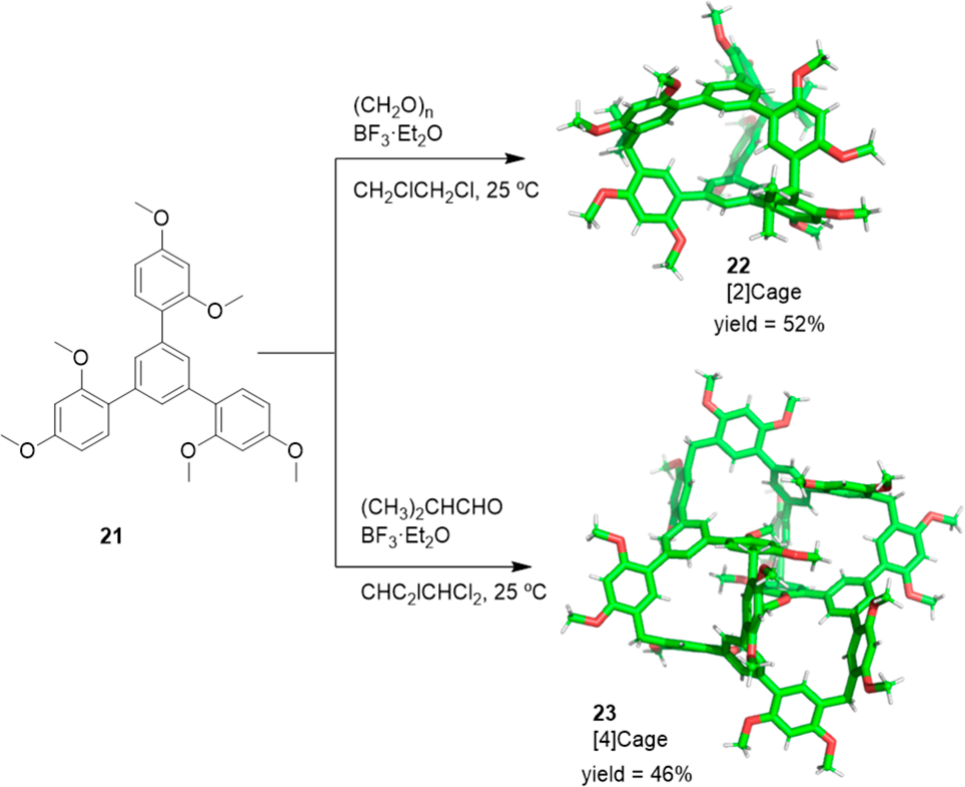
One-pot synthesis of covalent cages **22** and **23** from 1,3,5-tris(2,4-dimethoxyphenyl)benzene monomer and *para*-formaldehyde or isobutyraldehyde.^40^

Li and co-workers reported the one-pot synthesis of covalent organic cages from 1,3,5-tris(2,4-dimethoxyphenyl)benzene **21** monomer and paraformaldehyde or isobutyraldehyde to yield the dimeric cage **22** with a 52% yield and the tetrameric cage **23** with a 46% yield, respectively. The [2]cage **22** has a cavity composed of three nearly identical buckets with a size of 10.5 Å × 9.4 Å and an additional central cavity of 9.4 Å × 5.7 Å. The [2]cage **22** was used as the stationary phase in gas chromatography, which allowed the separation of benzene/cyclohexane and toluene/methylcyclohexane mixtures (Figure 7).^40^

**Figure 8:**
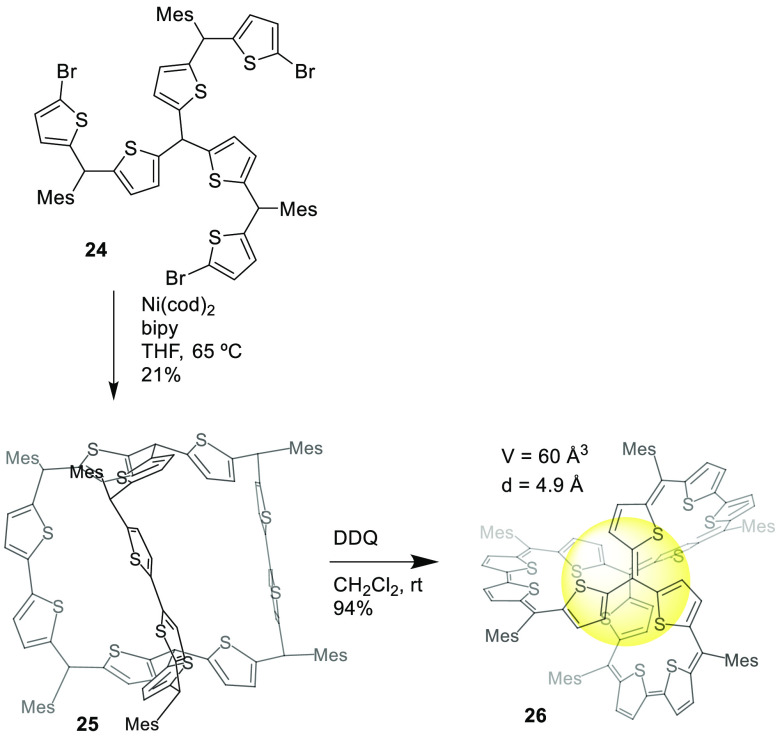
Synthesis of cage **26** with 3D global aromaticity.^41^

At this point, the different examples of molecular cages presented above in this section consist of sp^2^ or sp^2^–sp hybridized carbons. In addition to these structures, Wu and co-workers prepared a cage structure with 3D global aromaticity in an entirely conjugated diradicaloid. The cage-formation step involved a Yamamoto homocoupling of precursor **24** to yield nonaromatic cage **25** with a yield of 21%, which was oxidized using DDQ to the final conjugated cage **26** with a yield of 21%. The obtained cage showed 3D global aromaticity/antiaromaticity behavior depending on the number of delocalized π electrons and the spin state. For the prepared cages, the neutral compound displayed an open-shell singlet ground state with the 38π monocyclic conjugation pathway showing aromaticity; the dication had a triplet ground state with a dominant 36π monocyclic conjugation pathway displaying aromaticity, the tetracation had 52 π-electrons (open-shell singlet) delocalized along the 3D rigid framework showing 3D global antiaromaticity, and the hexacation possesses *D*_3_ symmetry with 50 delocalized π-electrons presenting global aromaticity (Figure 8).^41^

**Figure 9:**
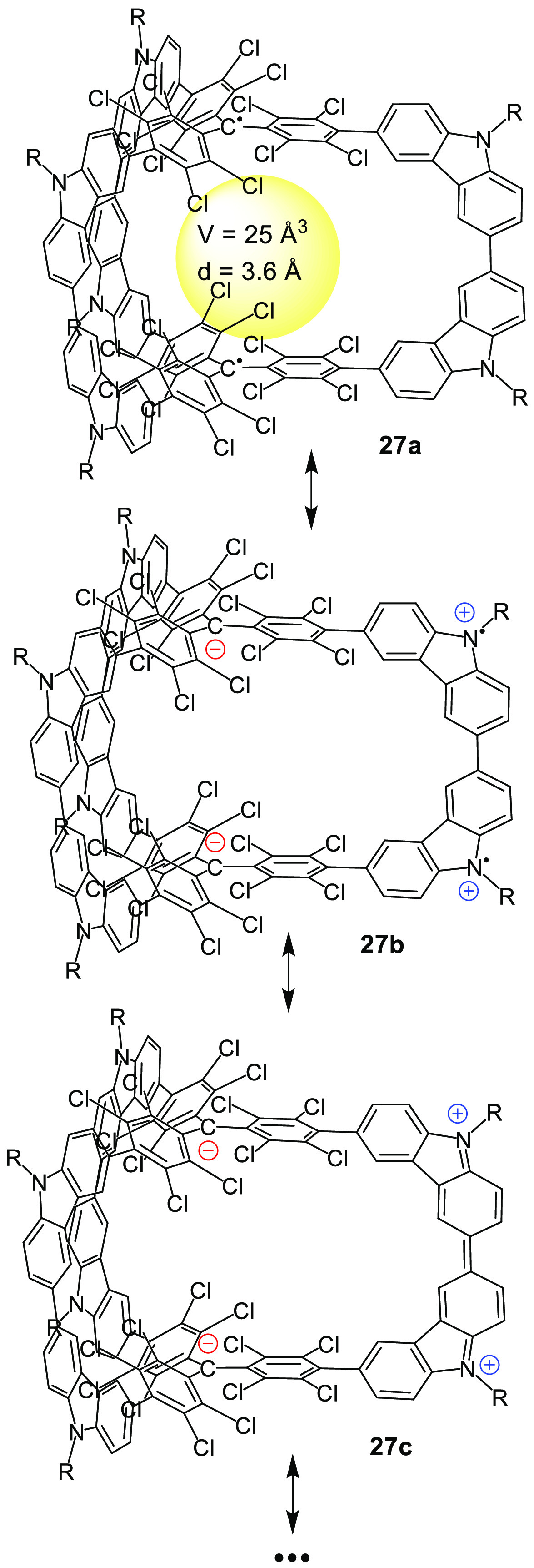
π-Conjugated diradical cage **27** and its three representative resonance forms.^42^

By a similar approach, Wu and co-workers reported the first π-conjugated diradical molecular cage **27** in which two polychlorotriphenylmethyl (PTM) radicals were linked by three bis(3,6-carbazolyl) bridges. The two carbon-centered PTM radicals, which were separated by a long distance, were weakly coupled via electronic interactions through the carbazole spacers (see the resonant structures **27a**–**27c** in Figure 9).^42^

**Figure 10:**
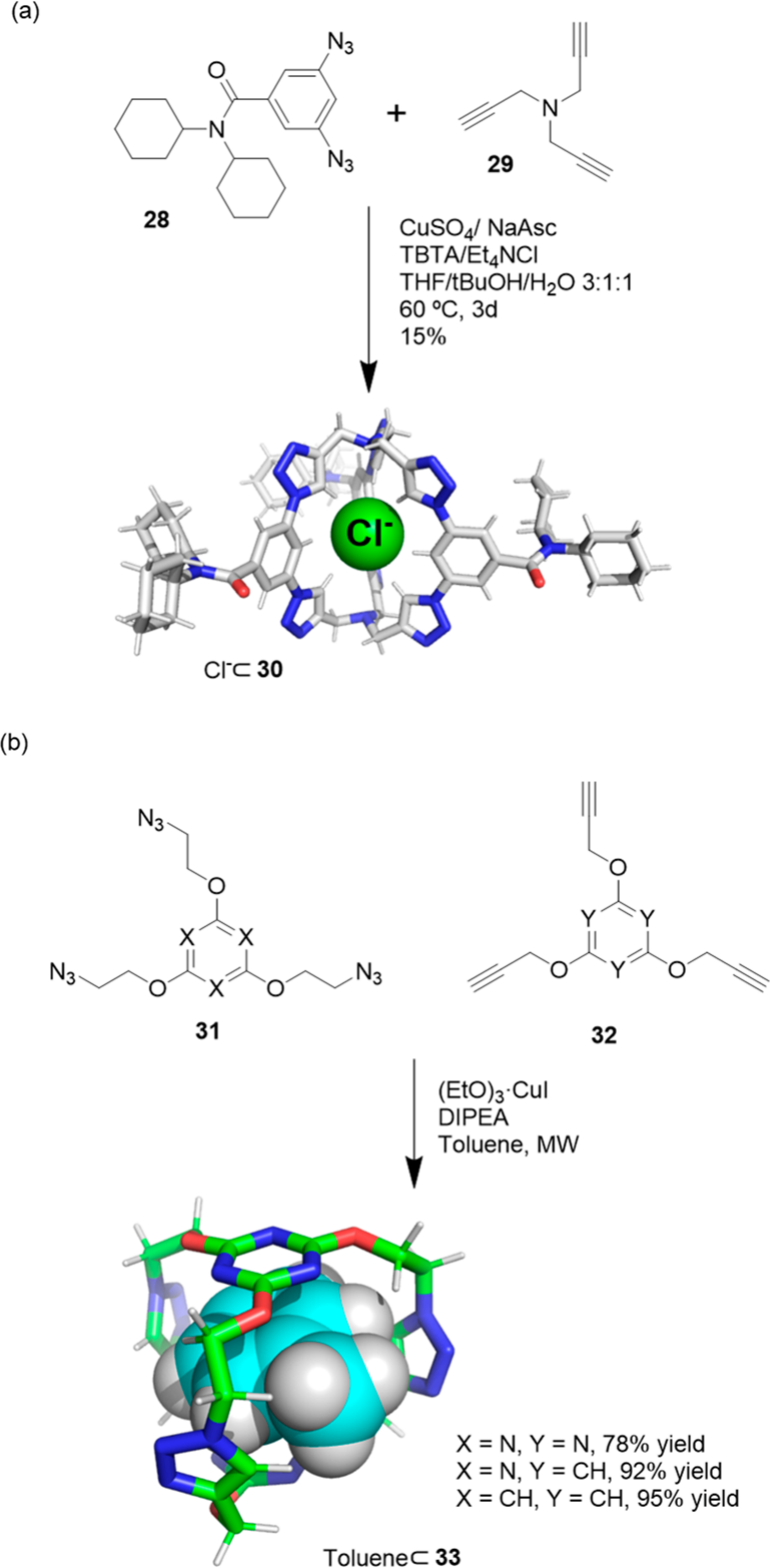
(a) Synthesis of the chloride-binding cage **30**.^43^ (b) Synthesis of toluene-binding cage **33**.^44^

Flood and co-workers used copper(I)-catalyzed alkyne–azide cycloaddition of precursors **28** and **29** to prepare cage **30** with a 15% yield. After purification, the X-ray crystal structure of **30** showed a complex with NaCl, and the chloride anion located in the cavity of the cage stabilized by nine hydrogen bonds, which indicates very high strong affinity (Figure 10a). Note that NaCl was not intentionally added, and the authors suggest that it was most probably scavenged from the silica used in the chromatographic purification of the cage. It was possible to remove 90% of the chloride from the cage by performing six extractions with deionized water. Host–guest experiments revealed nanomolar affinity toward chloride (*K*_assoc_ = ∼10^8^ M^–1^) and lower affinity toward larger anions (*K*_assoc_ bromide > nitrate > iodide) and anti-Hofmeister behavior. These exceptional properties allowed the authors to use the cage for both salt extraction and corrosion inhibition.^43^ Santoyo-Gonzalez and co-workers performed the copper(I)-catalyzed alkyne–azide cycloaddition of precursors **31** and **32** in the presence of toluene as a template to obtain cages **33** with high yields (>75%), which highlights the efficiency of the followed synthetic approach (Figure 10b).^44^

#### 2.1.2 Assembly through Reversible Bonds

**Figure 11:**
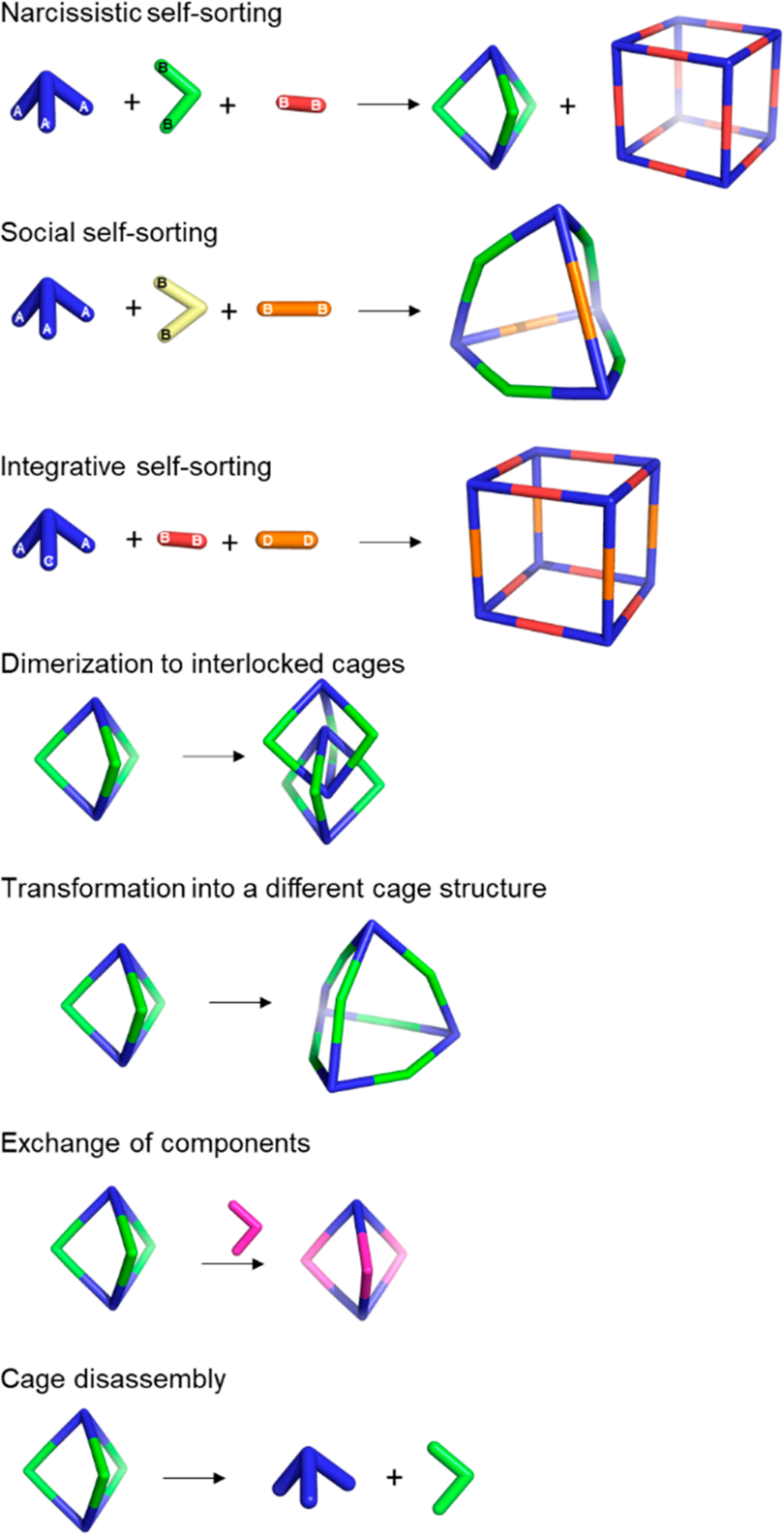
Types of self-sorting in the synthesis of cages from mixtures of one tritopic and two ditopic building blocks and transformations of cage structures.^46,47^ Building blocks’ reactivity complementarity A–B and C–D.

Dynamic covalent chemistry (DCC) allows thermodynamic equilibrium, which is a concept that has been key to developing cage structures.^45^ The use of reversible bonds allows cage self-assembly from simple binary mixtures of building blocks, and it is also possible to prepare more complex structures by employing the self-sorting of complex mixtures containing three building blocks or more. If self-sorting takes place by a narcissistic pathway, no complexity is introduced into the cage system, and the same products obtained from binary mixtures are generated. In contrast, if self-sorting occurs with a social or integrative pathway, it is possible to achieve complex cage structures. In social self-sorting, different building blocks with the same reactive groups are incorporated into the cage structure. In integrative self-sorting, building blocks with distinct reactive groups are incorporated into the cage structure. These different self-sorting methods are still very limited to date (Figure 11).^46^ Besides, once the cage is formed, the dynamic nature of cages can also generate dimerization in interlocked cages, transformation into distinct cage structures (transforming a cage into a another cage structure containing the same building blocks), exchange of components (transforming the cage structure to another cage with the same topology), and cage disassembly (breaking up a cage into its building block components).^47^

Of the various reversible bonds, imine bond formation is one of the most widely used in cage formation for a thermodynamically stable cage among the different possible cage derivatives to be prepared. Mastalerz and co-workers carefully studied imine bond dynamics in organic cage synthesis (see below). The literature generally describes that imine cages are thermodynamically controlled rather than kinetically controlled products. This criterion has been generally used to predict cage stability by molecular modeling, which assumes that self-correction mechanisms will take place until the thermodynamic equilibrium is reached and, therefore, the stablest cage structure is formed. This assumption is generally true because imine bonds are chemically labile. In some cases, however, the outcome of the reaction is the kinetic product of the reaction.^48^

**Figure 12:**
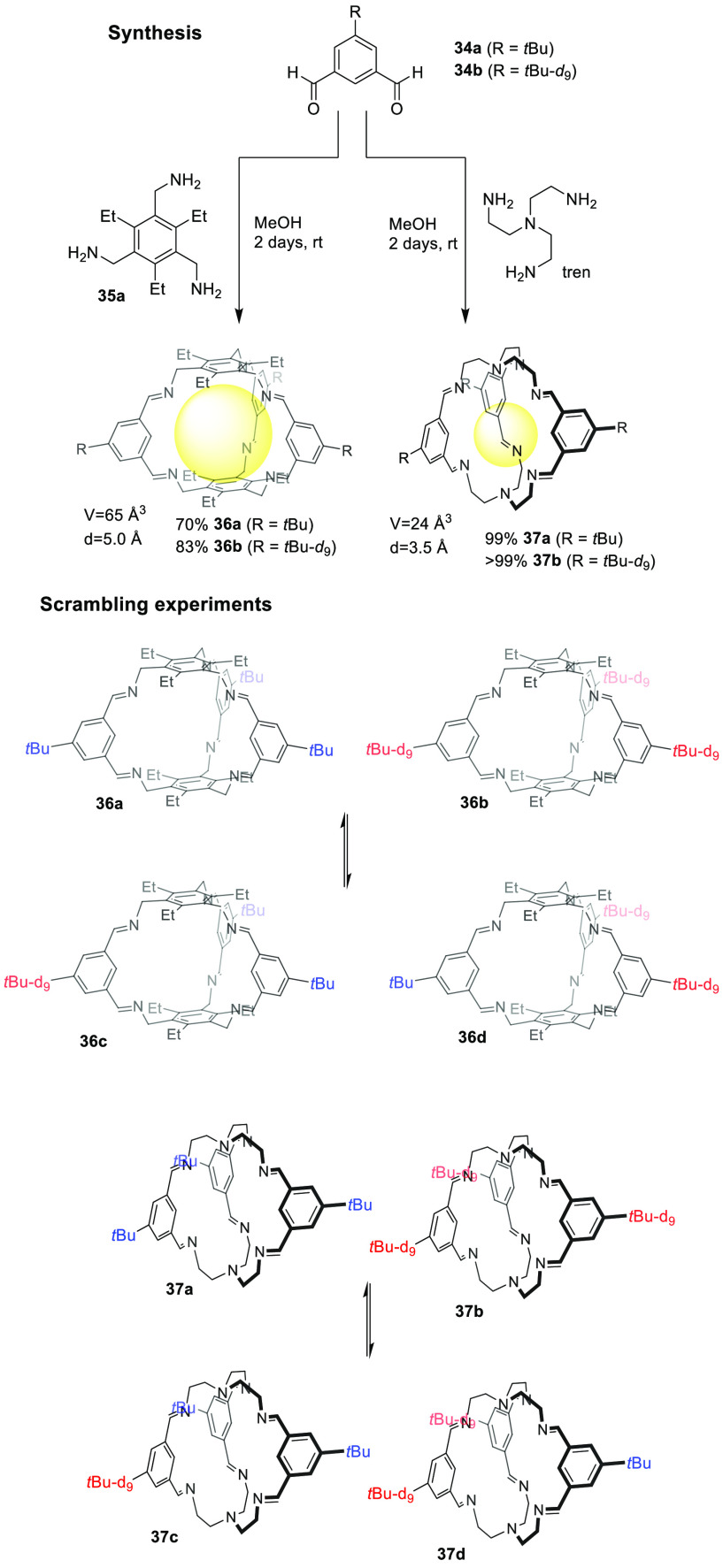
Synthesis of [2 + 3] imine cages **36** and **37** (deuterated and non deuterated) and scrambling experiments.^48^

On the one hand, the solvent in which the self-assembly reaction is performed plays an important role. For instance, an important key parameter is cage solubility, which is highly solvent-dependent. The reaction of **34** and **35** results in cage **36**, which precipitates from the reaction mixture in MeOH, EtOH, MeCN, CHCl_3_, THF, and dioxane, whereas it remains in solution in CH_2_Cl_2_ and toluene (Table 1). In contrast, cage **37** is more soluble and only precipitates in MeCN, THF, and dioxane. The same reactions (Figure 12 top) were also performed in the presence of 2 mol % trifluoroacetic acid (TFA) as a catalyst. The obtained results are comparable to those obtained without acid, with higher yields except for methanol.^48^ To determine imine cage formation dynamics, the scrambling of [2 + 3] imine cages with their deuterated analogues under different conditions like those reported in Table 1 and Table 2 was studied (Figure 12 bottom). For cage **36**, an exchange was observed only in CH_2_Cl_2_ after 7 days. Even after adding water or catalytic amounts of acid to the reaction mixture to facilitate imine hydrolysis in solvents other than CH_2_Cl_2_, no scrambling was observed. So it can be concluded that reasonable cage solubility, as observed in CH_2_Cl_2_, is necessary for dynamic exchange. Moreover, although the cage is soluble in toluene, no dynamic exchange was observed in this solvent, just as no exchange was observed in MeOH, EtOH, MeCN, CHCl_3_, THF, and dioxane, for which the cage had limited solubility. For cage **37**, no exchange was observed in any solvent under the same conditions, and it can be concluded that the aliphatic imine bonds in cage **37** were more stable than the aromatic imine bonds in cage **36**. It is necessary to add acid to produce the scrambling of cage **37** in MeCN or EtOH. Exchange experiments of cages with deuterated aldehyde building blocks and opposite amines provide further information systems’ dynamics and stability (Figure 12).^48^

**Table 1:** Cage **36** Synthesis Experiments in Different Solvents^b^

	precipitate (%)	mother liquor (%)	total (%)
MeOH	80	0	80
EtOH	40	15	55
MeCN	73	0	73
CH_2_Cl_2_	a	44	44
CHCl_3_	73	15	88
THF	58	0	58
dioxane	64	9	73
toluene	a	46	46

a No precipitate formed.

b Reaction carried out for 2 days at room temperature.^48^

**Table 2:** Cage **37** Synthesis Experiments in Different Solvents^b^^,^^48^

	precipitate (%)	mother liquor (%)	total (%)
MeOH	a	94	94
EtOH	a	93	93
MeCN	59	8	67
CH_2_Cl_2_	a	92	92
CHCl_3_	a	72	72
THF	68	21	89
dioxane	69	7	76
toluene	a	68	68

b For 2 days at room temperature: room temperature.

a No precipitate formed.

**Figure 13:**
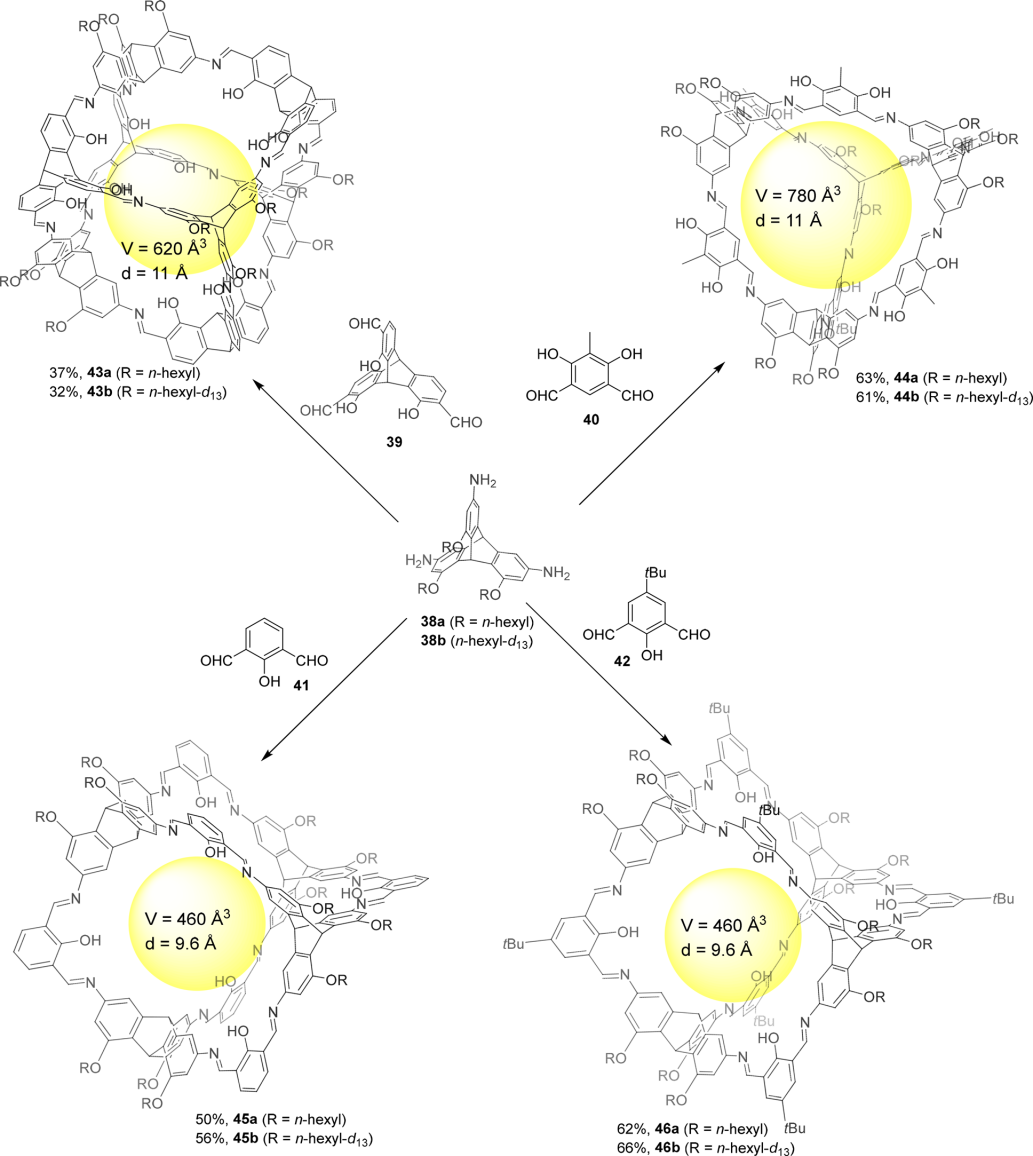
Synthesis of soluble cages **43**–**46** using *n*-hexyloxy functionalized triaminotriptycene.^49^

To study the dynamics of imine cage formation for larger cage systems, it is necessary to use solubilizing groups to solubilize cage and reaction intermediates. Along these lines, Mastalerz and co-workers introduced three deuterated and nondeuterated solubilizing *n*-hexyloxy groups into triptycene triamine **38**, which was reacted with dialdehydes **39**–**42** and resulted in cages **43**–**46** (Figure 13). Cages and intermediates were fully soluble, which allowed cage formation kinetics to be explored. Whereas the [4 + 6] endo cages **45** and **46** quickly formed (approximately 1 h), the [4 + 4] cage **43** formed slowly, even at 150 °C. The authors found in the reaction mixture that larger species (oligomers and polymers) were formed, which were converted into the thermodynamically controlled [4 + 4] cage product. These results fall in line with scrambling experiments, which revealed that the cubic [4 + 4] cage **43** and its deuterated analogue did not undergo exchange. In contrast, the [4 + 6] exofunctionalized cage **44** scrambled in the presence of catalytic amounts of TFA or *p*-toluidine. The [4 + 6] endo cages **45** and **46** showed only significant exchange with *p*-toluidine and TFA and slow exchange in the presence of TFA and water (Figure 13).^49^

**Figure 14:**
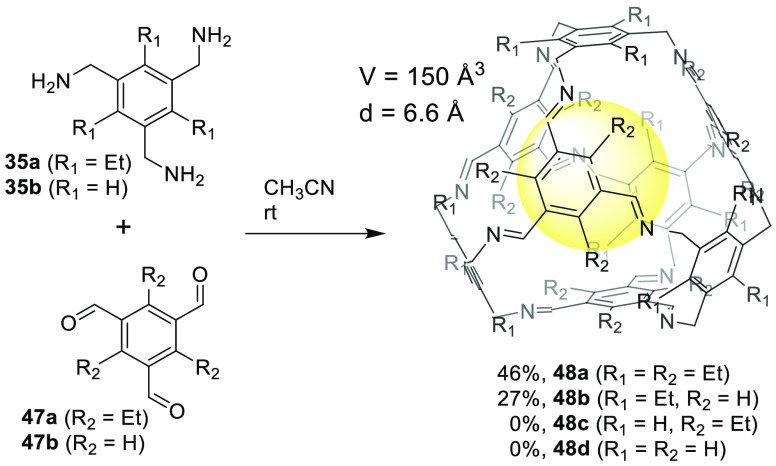
Synthesis of cage **48** by [4 + 4]-condensation of trimethylamines and trialdehydes.^50^

Another key aspect that defines the success or failure in the synthesis of organic cages is the geometry and rigidity of building blocks, which must have the appropriate conformation and preorganization. Mastalerz and co-workers studied the influence of building block conformational rigidity on the formation of a [4 + 4] imine cage with a truncated tetrahedral geometry over time (Figure 14). The rotational freedom of the C_sp2_–C_sp3_ bonds in at least one of the precursors needs to be highly restricted for the formation of [4 + 4] imine cage. The preorganization of reacting groups is crucial if cage formation is kinetically controlled. The stablest conformation of **35a** has alternating substituents by placing all the methylamines on one side of the benzene ring with a very large methylamine C_sp2_–C_sp3_ rotational barrier (+227.7 kcal/mol). In contrast, **35b** has a very small rotation barrier (+3.5 kcal/mol), which results in fast rotation under reaction conditions and, therefore, amine groups are not preorganized. For the **47a** building block, the most stable conformation contains aldehyde groups slightly out of plane (dihedral angle ∼40°) by the steric strain of ethyl moieties. However, this preorganization is not favorable because C=O bonds are not orthogonally oriented to the molecular plane. The rotational barrier for **47a** is +34.5 kcal/mol. The most stable conformation for **47b** is that with all the aldehyde groups in the molecular plane. The rotational barrier is +42.4 kcal/mol.^50^

As a consequence of the non-appropriate preorganization of building blocks, it is not surprising that the reaction between **35b** and **47b** does not produce the corresponding **48d** cage and yields polymeric byproducts. The reaction of **35b** and **47a** also produces polymeric byproducts rather than cage **48c**. In contrast, the reaction of the favorable preorganized **35a** building block with **47b** yields cage **48b** (27% yield) in acetonitrile at room temperature in 3 days in the absence of catalytic trifluoroacetic acid. This scenario suggests that cage formation is kinetically controlled, and thermodynamic products are the least structurally ordered polymers to form in the presence of catalytic acid. The reaction of **35a** and **47a** also yields the corresponding **48a** cage with a 46% yield. The solid-state structures of **48a** and **48b** show that cage windows are blocked by alkyl chains. Activation of solids by the exchange first with *n*-pentane and then with liquid ethane results in porous solids with BET surface areas of 443 m^2^/g for cage **48b** and 71 m^2^/g for cage **48a** (Figure 14).^50^

**Figure 15:**
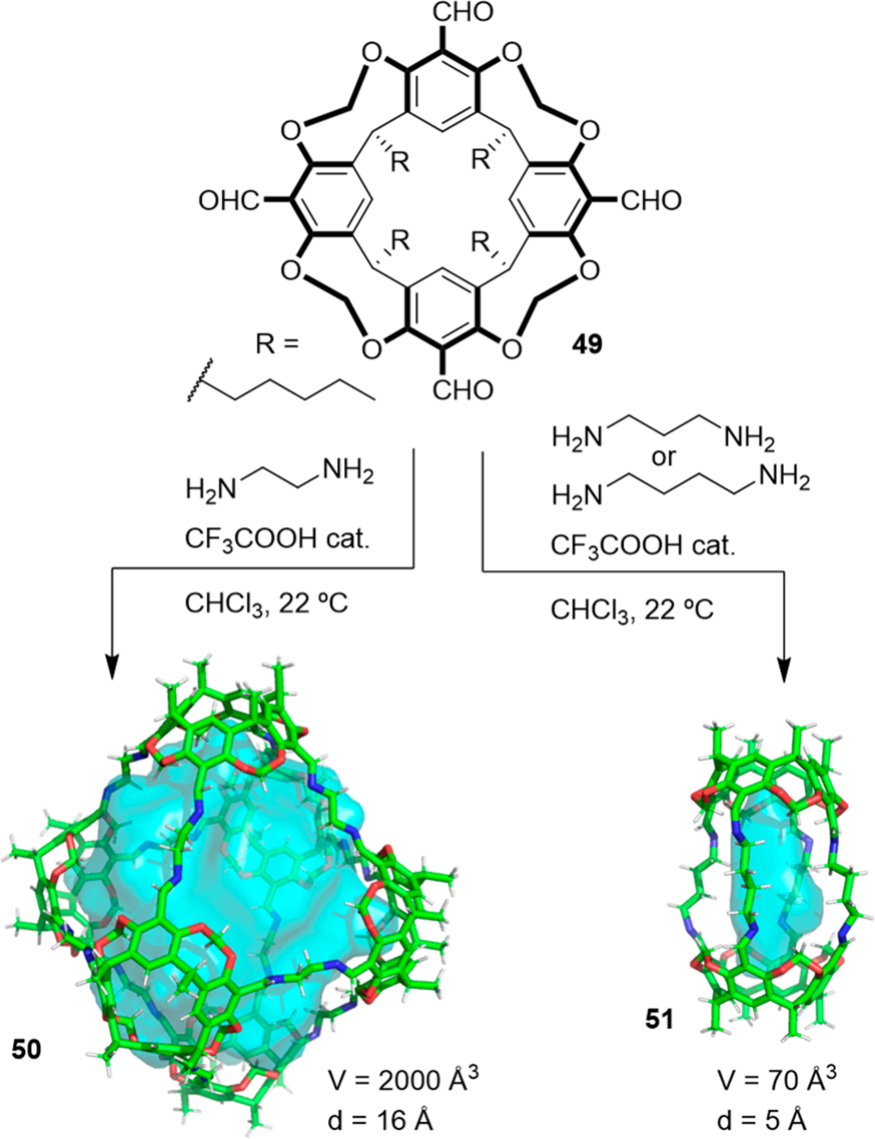
Thermodynamically controlled synthesis of octahedral nanocage **50** and hemicarcerand **51**.^53^

Subtle differences in building block size/geometry can result in completely different cage structures, as shown in the following example. Stoddart, Cram, and co-workers demonstrated that the reaction of tetraformylcavitand **49** with 1,3-phenylenediamine under thermodynamic control quantitatively yielded the corresponding hemicarcerand formed by two cavitands connected together with four diamines through eight imine bonds.^51,52^ On the basis of this cage motif, Warmuth and co-workers reported the synthesis of octahedral nanocage **50** by the condensation of a tetraformylcavitand and ethylene-1,2-diamine catalyzed by trifluoroacetic acid in CHCl_3_ with an 82% yield. This cage structure was formed by six cavitands, connected together with 12 diamino bridging units through 24 imine bonds, and its cavity size was approximately 1700 Å^3^ (Figure 15). When the authors performed the reaction using 1,3-diaminopropane or 1,4-diaminobutane, they obtained hemicarcerand **51** with a yield over 95%. It has the same topology as that obtained by Cram when performing the same reaction with 1,3-phenylenediamine.^52^

An analysis of the molecular models of the octahedral cage **50** structure shows that the ethylenediamine units are forced into a *gauche* conformation. In contrast, they are in an *anti* conformation in hemicarcerand **51** by minimizing the linker strain. This is supported by the fact that the same reaction with (*R*,*R*)-1,2-diaminocyclohexane, which has the amino groups in a *trans* conformation, only produces polymeric products and no hemicarcerand is detected, which is expected given the unfavorable conformation of this diamine. The authors also found that using CHCl_3_ as the solvent is key for obtaining the octahedral cage. Thus by performing the reaction in CH_2_ClCH_2_Cl or CHCl_2_CHCl_2_, it resulted in a cage formation yield below 20%. Overall, this simple efficient methodology demonstrates that it is possible to synthesize nanosized cage molecules with almost 20 components in a single step.^53^

**Figure 16:**
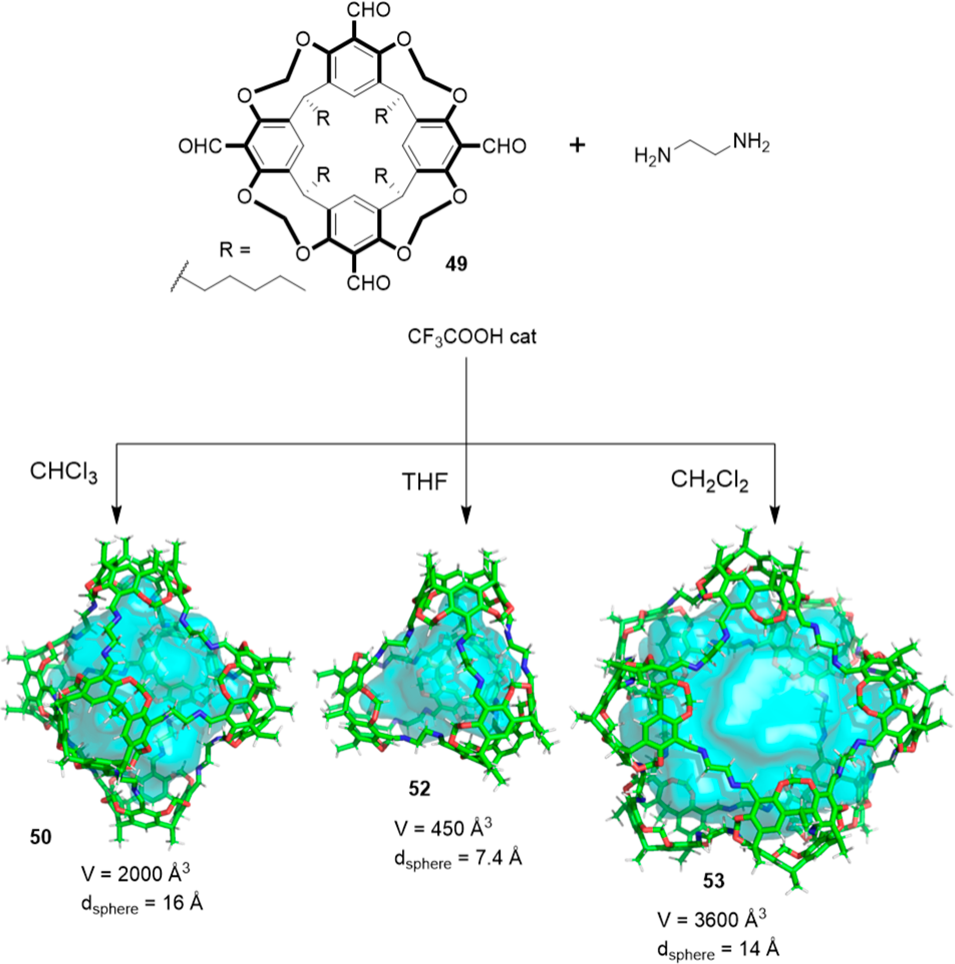
Synthesis of octahedral (**50**), tetrahedral (**52**), and square antiprismatic (**53**) cages by the reaction of **49** with ethylene-1,2-diamine in different solvents.^54^

Further studies by Warmuth and co-workers allowed determining the solvent effect in the self-assembly of the tetraformylcavitand **49** and 1,2-ethylenediamine building blocks. The authors found that the final outcome of the reaction could be fine-tuned by changing the solvent to predominately obtain tetrahedral cage **52** in THF, octahedral cage **50** in CHCl_3_ and square antiprismatic cage **51** in CH_2_Cl_2_. These results highlight the key role of the solvent in the cage self-assembly process, and the importance of solvent optimization for maximizing cage formation yield (Figure 16).^54^

**Figure 17:**
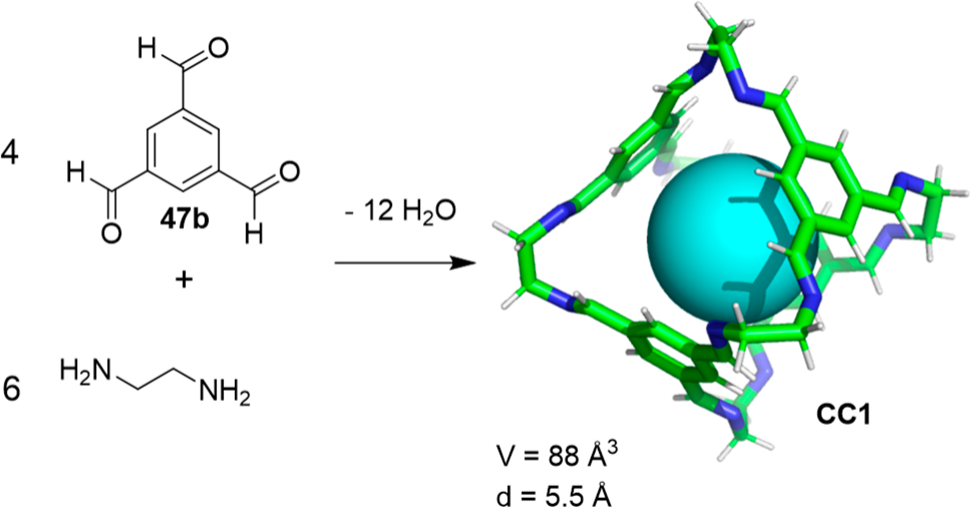
Synthesis of cage **CC1**.^55^

In another example, solvent effects are also important and define the **CC1** cage formation outcome. Cooper and co-workers described an improved multigram synthesis for the **CC1** [4 + 6] imine cage by obtaining the cage with yields close to 100%, whereas the original synthesis reported a yield of approximately 35% (Figure 17).^55,56^ The first reported synthesis of cage **CC1** consisted in performing the reaction by direct crystallization via the slow vapor diffusion of solid aldehyde **47b** in a concentrated solution containing ethylenediamine, whereas the aldehyde solution is slowly added to the amine solution in the new approach. Solvent screening allowed CH_2_Cl_2_ to be selected as the optimal solvent (Table 3). In addition, optimizing the concentration of reagents allowed isolation of the cage with a 96% yield. Above a critical benzene-1,3,5-tricarboxaldehyde (23.13 mM) concentration, conversion decreases from 100% to 98%, and product isolation becomes more difficult.^57^ This synthetic strategy allows the synthesis of cages with different properties by the modification of building blocks to obtain self-assembled porous molecular materials for selective guest binding in either the crystalline or amorphous solid state.^58^

**Table 3:** Summary of the Experimental Conditions and Yields for Synthesis of Cage **CC1**(57)

solvent	initial aldehyde conc (mM)^a^	volume (L)^b^	isolated (yield%)^c^	conversion (%)
*i*PrOH	4.40	0.700	100	86
EtOAc	4.40	0.700	94	83
EtOH	4.40	0.700	95	72
CH_2_Cl_2_	4.40	0.700	100	87
THF	4.40	0.700	100	89
CH_2_Cl_2_	7.70	0.700	96	100
CH_2_Cl_2_	9.63	0.700	93	100
CH_2_Cl_2_	15.4	0.700	96	100
CH_2_Cl_2_	19.25	0.700	90	100
CH_2_Cl_2_	19.28	1.050	95	100
CH_2_Cl_2_	20.11	2.000	96	100
CH_2_Cl_2_	23.13	0.700	nd	98
CH_2_Cl_2_	26.98	0.700	nd	98

a Molar aldehyde/amine ratio = 1:1.5.

b Total final volume of the reaction mixture after adding aldehyde solution (volume ratio of aldehyde/amine solutions = 4:3). Aldehyde solution added over 48 h.

c nd = not determined.

**Figure 18:**
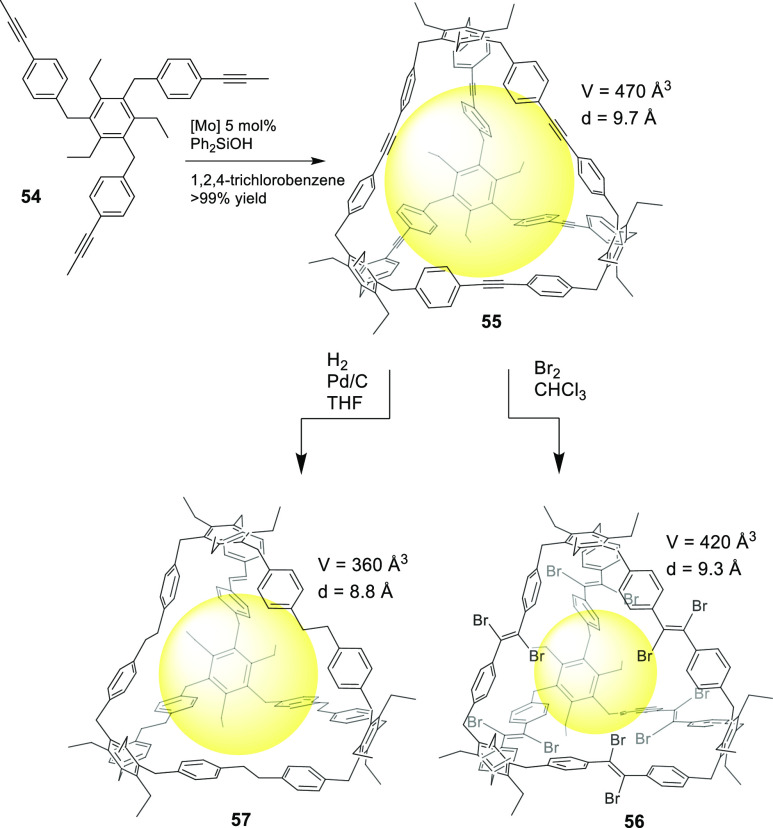
Synthesis of molecular cage **55**, followed by postsynthetic modifications to give cages **56** and **57**.^61^

Besides imine bond formation, other reversible reactions also allow to obtain cage structures. For example, alkyne metathesis is a reversible reaction that can be used to prepare cages, which generally provides thermodynamically stable products upon equilibrium.^59^ Moore and co-workers used precursor **54** for the synthesis of tetrahedral cage **55** using alkyne metathesis with high yields. The authors suggest that the formed cages are thermodynamically very stable and create large kinetic barriers to prevent cage disassembly.^60^ Using tetrahedral cage **55**, Moore, Zhang, and co-workers made postsynthetic modifications to obtain organic cages with alkynyl (**55**), alkenyl (**56**), and alkyl (**57**) edges. This allowed them to study the effects of building block shape persistence, as well as the building blocks’ rigidity/flexibility effects, on cage porosity. Once cages had been prepared, both the rapidly crystallized and slowly crystallized solids were studied by nitrogen adsorption experiments, which illustrated a general porosity trend: alkynyl (**55**) > alkenyl (**56**) > alkyl (**57**). Additional molecular dynamics calculations demonstrated that the microporosity of the molecular cages directly correlated with their relative shape persistence (Figure 18).^61^

**Figure 19:**
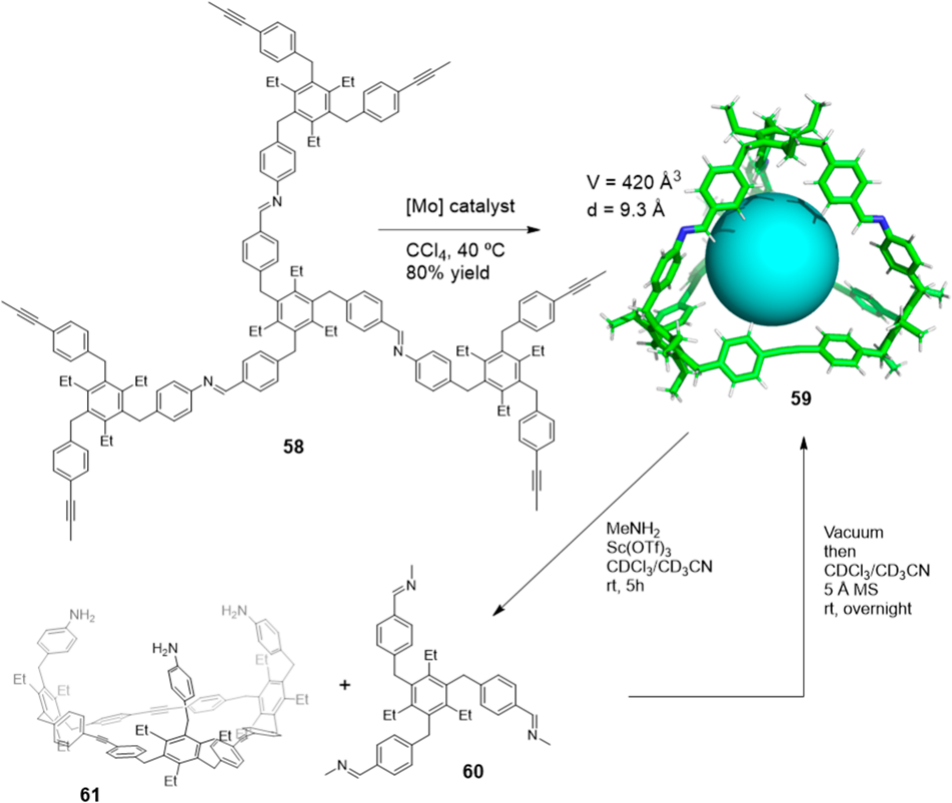
Synthesis of the organic cage **59** by a tandem imine condensation and alkyne metathesis. Sc(III) catalyzed transimination vertex replacement.^62^

Alkyne metathesis is also compatible with imine bonds by allowing complex cage structures to be prepared. Moore and co-workers reported the first system to combine orthogonal alkyne and imine bonds to prepare a molecular cage with a reversibly removable vertex. Monitoring the cage formation reaction from precursor **58** with [Mo] catalyst by GPC showed that higher-molecular-weight oligomeric products are initially formed, which reversibly corrected to form tetrahedral cage **59** as the favored reaction product. The imine-linked vertex can be removed through an Sc(III)-catalyzed transamination to produce cage disassembly by forming compounds **60** and **61**. Cage reassembly can be performed by removing volatile methylamine and redissolving the residue in the presence of 5 Å molecular sieves to yield the initial organic cage. The authors pointed out that the developed method offered a strategy for preparing cages of altered symmetry and one that provides new possibilities to prepare cages that can be selectively modified (Figure 19).^62^

**Figure 20:**
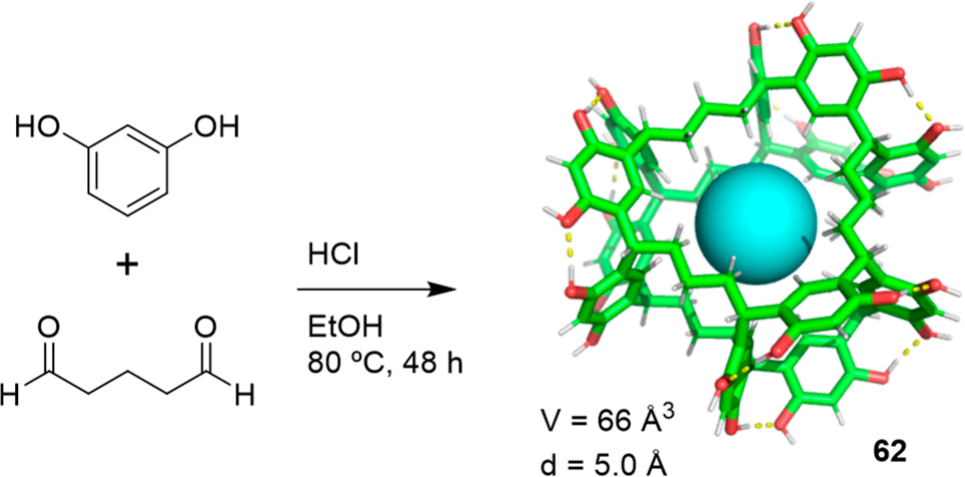
Reaction of resorcinol with 1,5-pentanedial to form cage **62**.^64^

The reversible reaction between resorcinol and α,ω-alkanedials can also be used to prepare stable cages.^63^ The first example using this reaction for cage formation was reported by Nishikubo and co-workers, who prepared a cage structure from the condensation of resorcinol and 1,5-pentanedial whose structure (**62**) resembles a molecular waterwheel. Cage **62** is obtained with an 83% yield through a mechanism that involves the reaction of resorcinol and 1,5-pentanedial to initially yield oligomeric and polymeric species, which evolve to the final cage product with time. The cage showed high selectivity for the complexation of Rb^+^ versus Na^+^, K^+^, and Cs^+^, which was presumably encapsulated in the central hydrophobic cavity (Figure 20).^64^

**Figure 21:**
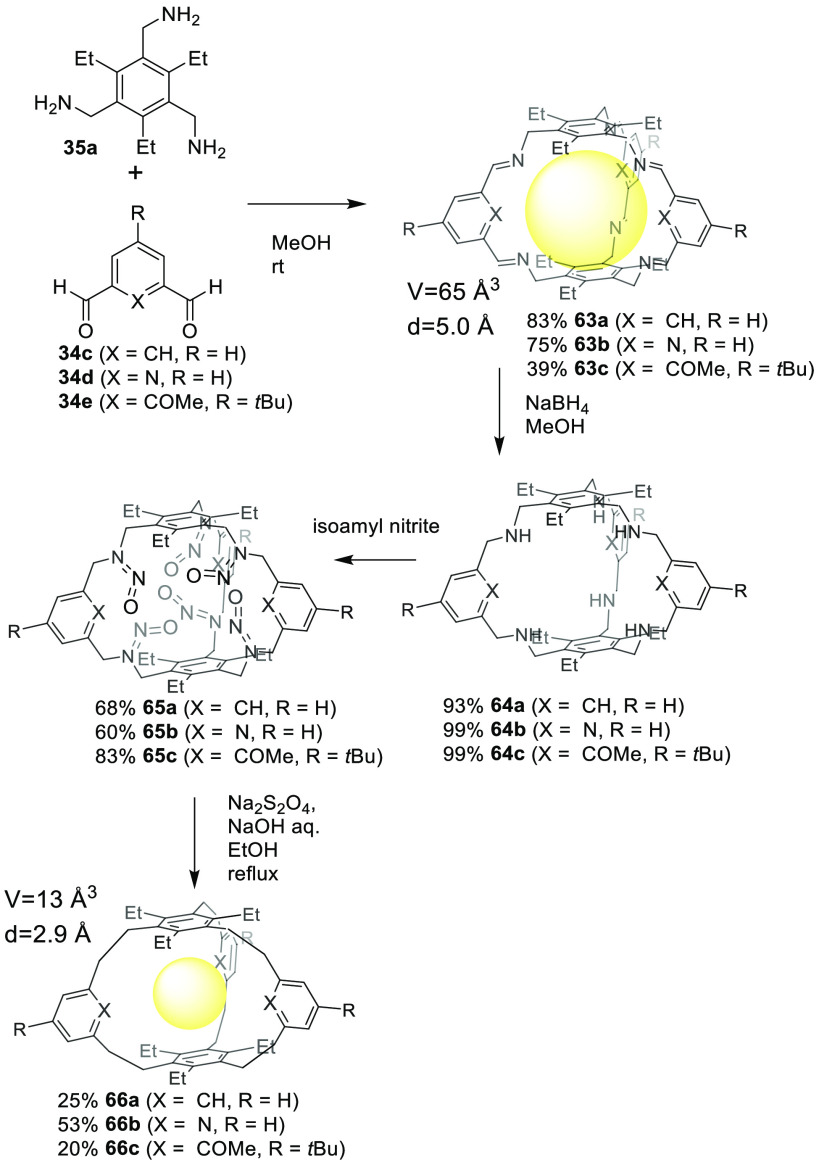
Transformation of imine cages **63** into hydrocarbon cages **66**, through intermediates 64 and **65**.^67^

Cages self-assembled by reversible bonds may have limited stability that can reduce their applications. This can be overcome by transforming the final reversible bonds formed in the cage into irreversible bonds.^65,66^ A typical example is the transformation by the reduction of reversible imine bonds to amino groups. In a more sophisticated study, Mastalerz and co-workers reported the transformation of imine cages **63** in only three steps involving cages **64** and **65** to yield the final hydrocarbon cages **66**. This robust methodology allowed the preparation of an even larger cage structure C_72_H_72_, which was predicted but was impossible to synthesize until this methodology was developed. The main advantage of this methodology was the use of high-yielding imine bond formation in the first step to generate the cage scaffold, unlike the direct synthesis of hydrocarbon cages using an irreversible bond formation that normally results in low yields (Figure 21).^67^

**Figure 22:**
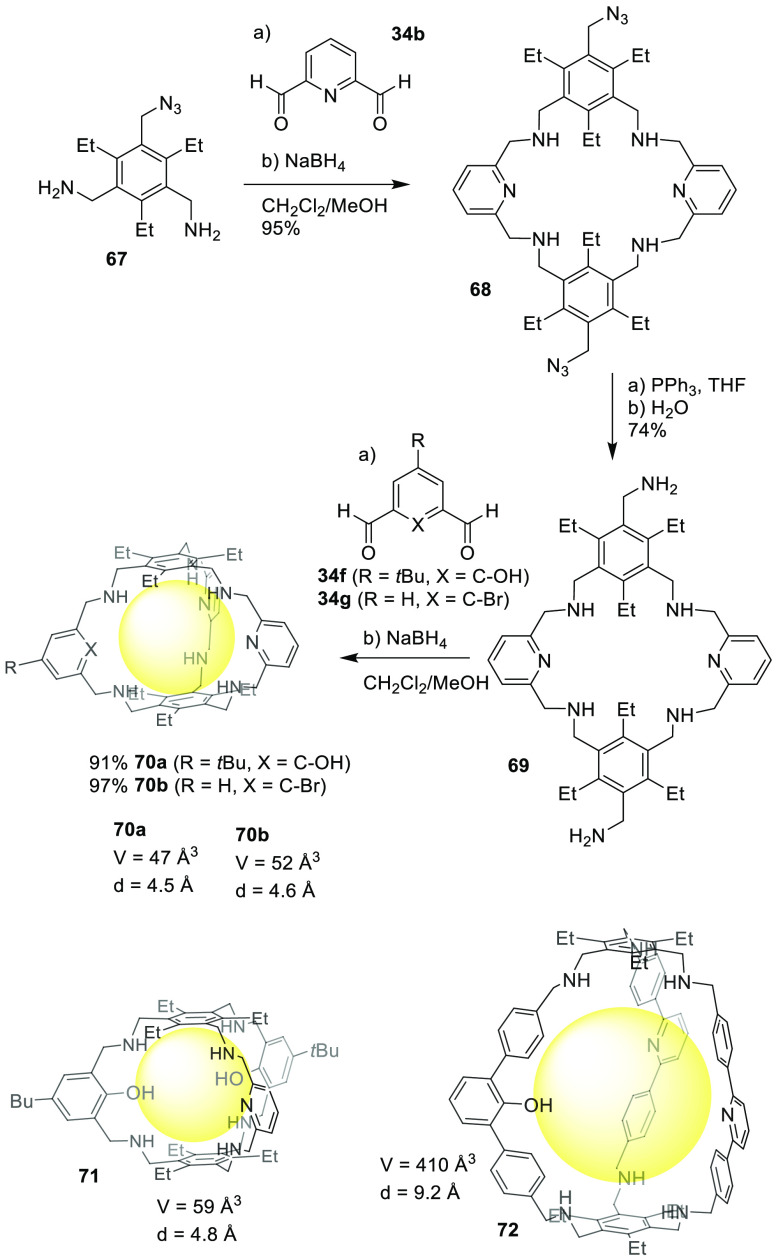
Synthesis of heterosequenced cages **70**, **71**, and **72**.^68^

Otte and co-workers described a robust synthetic protocol using amine and aldehyde building blocks to prepare heteroleptic cages. One example is described in Figure 22. It started from reactant **67**, which was obtained by the transformation of one of the amino groups in triamine building block **35a** into an azide moiety, which was further condensed with dialdehyde **34b** to obtain intermediate **68**, in which azide groups were further converted into amines to give intermediate **69**. Condensation with another dialdehyde resulted in the formation of the final cage **70**. Following a similar strategy, the authors prepared cages **71** and **72** (Figure 22).^68^

#### 2.1.3 Template Synthesis

**Figure 23:**
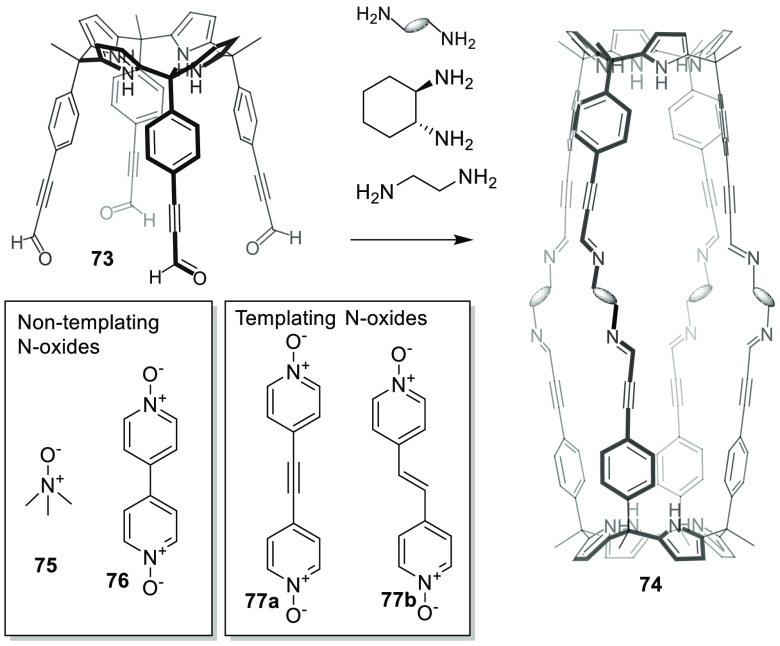
Templated formation of capsule **74** from tetraaldehyde calix[4]pyrrole **73**.^69^

Whereas the synthesis of cages requires building blocks with the appropriate geometry and preorganization, in some instances the expected cage product is not obtained. As an alternative, different research groups have developed interesting methods using guest templates to obtain the target cage structure. With this general approach, Ballester and co-workers prepared chiral polyimine molecular capsules with polar interiors using *N*-oxides as templates for the efficient self-assembly of tetraaldehyde calix[4]pyrrole **73** with diamines in capsule **74** (Figure 23). Initial attempts to assemble capsules from their building blocks yielded insoluble precipitate materials that precluded capsule formation. The same results were obtained when *N*-oxides **75** or **76** were used as templates. In contrast, the addition of one equivalent of bispyridyl-*N*-oxide templates **77a** or **77b** resulted in a quantitative assembly of capsule **74**. The template formed hydrogen bonds with two tetraaldehyde calix[4]pyrrole units in **73** and brought them to close proximity by favoring capsule formation by the reaction with the diamine and by avoiding undesired oligomerization reactions (Figure 23).^69^ Further examples include the templated formation of hydrogen-bonded capsules, which are described in detail in section 3.2.3.

**Figure 24:**
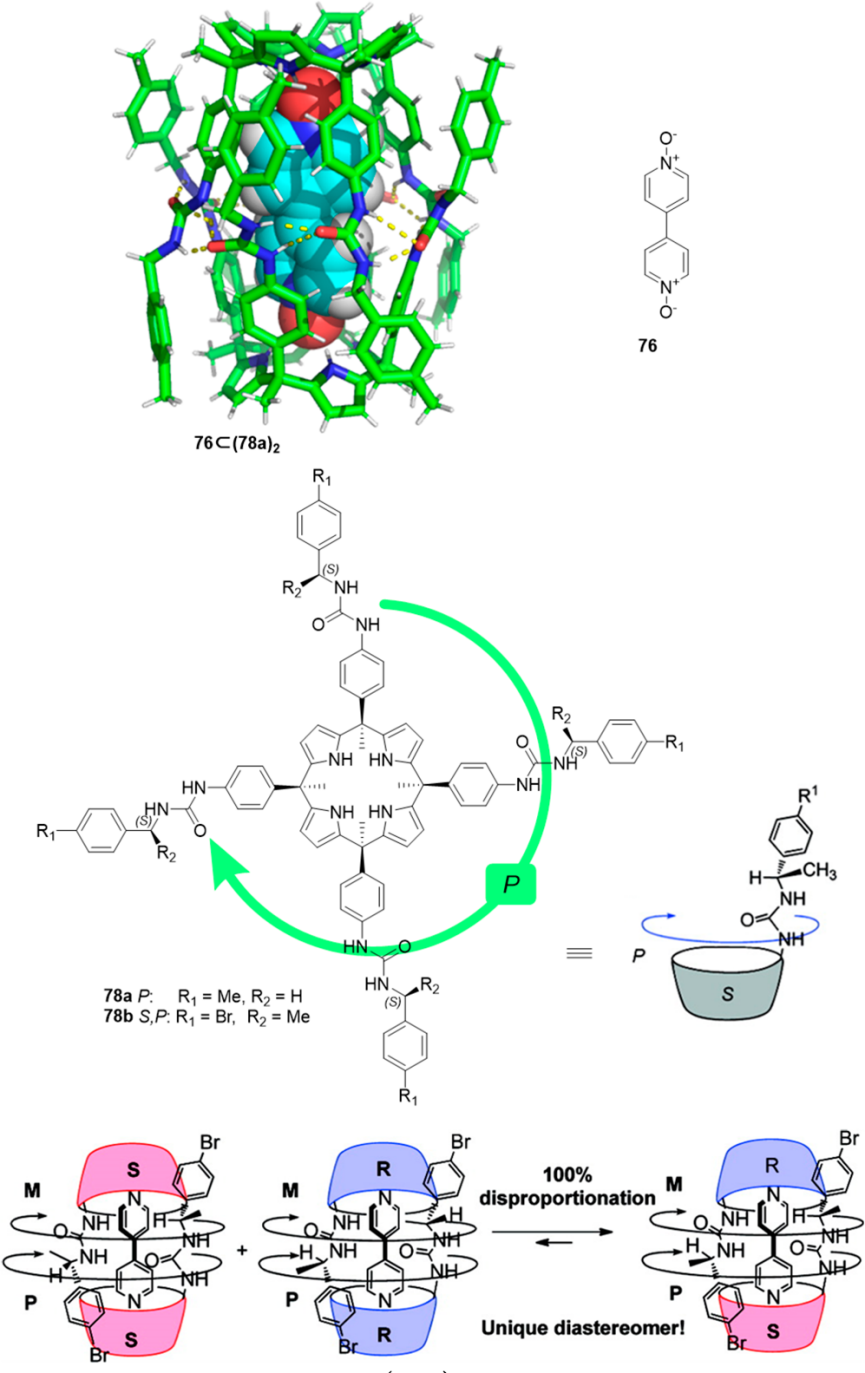
Structure of capsule (**78a**)_2_ with encapsulated bipyridine bis-*N*-oxide (top). Self-sorting of a calix[4]pyrrole **78a** incorporation chiral groups (bottom).^70−72^ Adapted with permission from ref (72). Copyright 2010 American Chemical Society.

Using the same templating bispyridyl-*N*-oxide motifs, Ballester and his team used tetraurea calix[4]pyrrole **78** to form the corresponding dimeric tetraurea calix[4]pyrrole capsules. The capsule was formed reversibly by a cyclic array of 16 hydrogen bonds in nonpolar solvents in the presence of an appropriate template (4,4′-bipyridine-bis-*N*-oxide **76**). The template was required to obtain the capsule because no evidence of cage formation was observed in the absence of the template. The template significantly stabilized capsule assembly by the formation of hydrogen bonds between *N*-oxide oxygen atoms and the calixpyrrole (Figure 24).^70,71^ By changing one hydrogen atom for a methyl group at each benzylic carbon, four stereogenic centers with the same chirality were created. These chiral groups produced cyclochiral diastereoisomers, designated as *S*,*P*-**78b** and *S*,*M*-**78b** or *R*,*P*-**78b** and *R*,*M*-**78b**, depending on the configuration of the chiral group. Using an enantiomerically pure calixpyrrole in the presence of 4,4′-bipyridine-bis-*N*-oxide **76**, capsule self-assembly only took place with conformationally cyclochiral enantiomers ((*S*,*P*·*S*,*M*)-(**78b**)_2_ or (*R*,*P*·*R*,*M*)-(**78b**)_2_) with urea groups rotated 180° to favor hydrogen bonding. In contrast, when the assembly was performed using racemic calixpyrrole in the presence of **76**, a mixture of kinetically trapped capsule products following an approximate statistical mixture was obtained. Interestingly, the mixture evolved to the equilibrium by the addition of a small amount of hydrogen-bonding solvent THF to yield a single heterodimeric capsule (*S*,*M*·*R*,*P*)-(**78b**)_2_. When self-assembly was performed in the presence of trimethylamine-*N*-oxide, the single heterodimeric capsule (*S*,*M*·*R*,*P*)-(**78b**)_2_ was obtained even in the absence of THF.^72^

**Figure 25:**
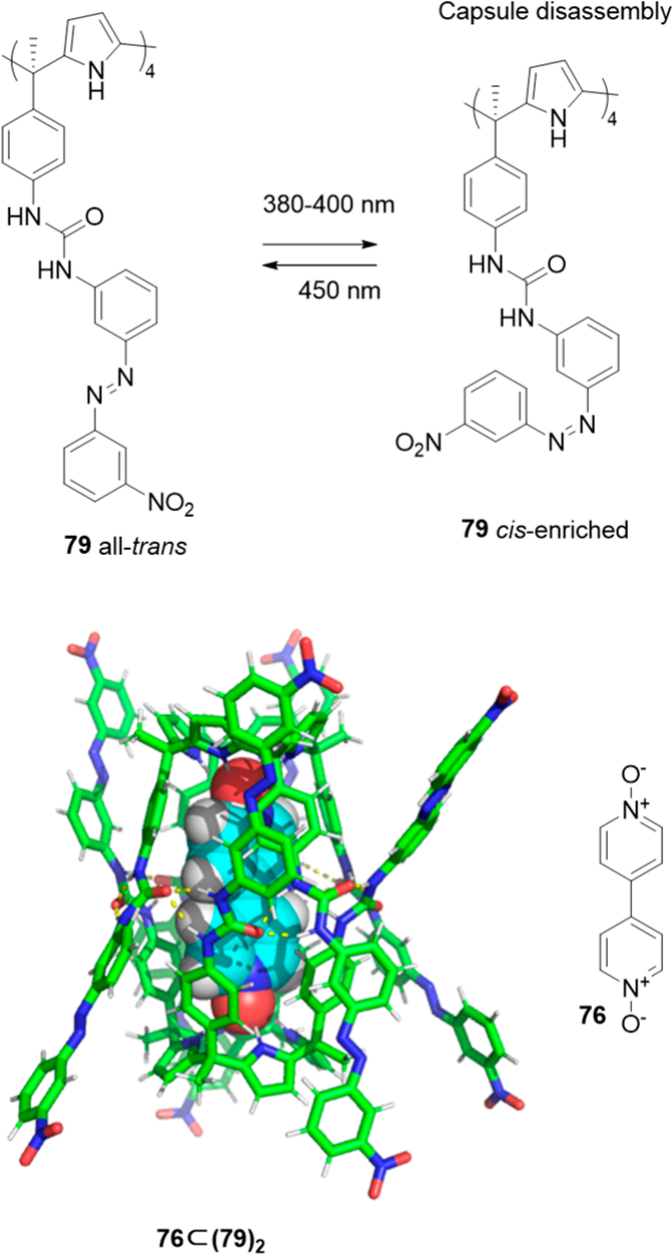
Photoisomerization of the azobenzene group of the tetraurea-calix[4]pyrrole **79** (top). Structure of capsule (**79**)_2_ with encapsulated 4,4′-bipyridine-bis-*N*-oxide **76** (bottom).^74^

On the basis of these systems, a light-controlled cage assembly/disassembly of the above-described hydrogen-bonded cages was possible by introducing photoactive chemical units into the calix[4]pyrrole building block.^73^ Functionalization of the calix[4]pyrrole with azobenzene groups allowed azobenzene light-induced *trans*-to-*cis*-isomerization upon irradiation, which resulted in the disassembly of cage **76⊂(79)**_**2**_. The authors suggested that the isomerization of only one azo group probably sufficed to prompt capsule disruption. Capsule reassembly was achieved by reverse *cis*-to-*trans* relaxation in the dark. As described above in the parent system, the presence of template bipyridine bis-*N*-oxide **76** was also neceassary here to observe capsule formation (Figure 25).^74^

**Figure 26:**
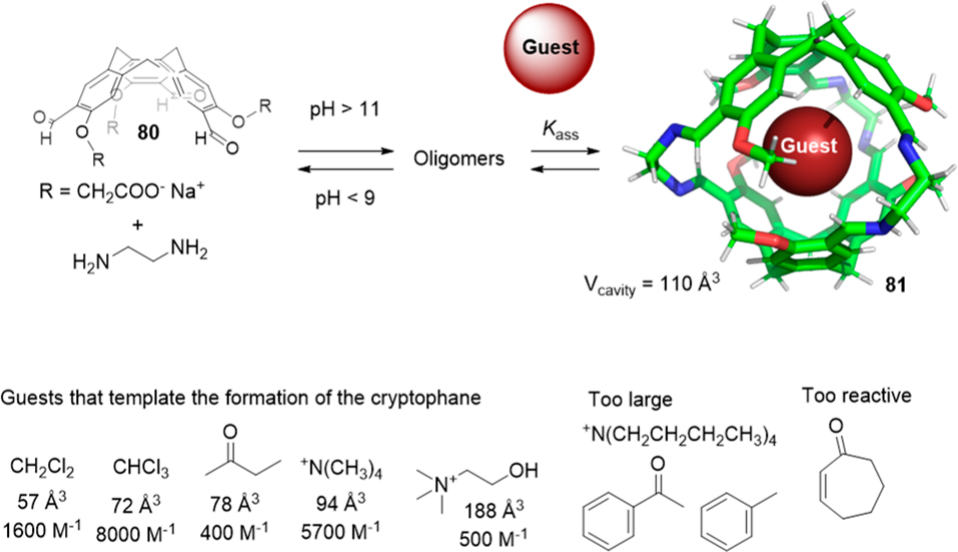
Templated cryptophane **81** formation in D_2_O. The R group omitted for clarity in the cryptophane model.^75^

Besides templated hydrogen bonding, templated imine chemistry has also been used to favor the synthesis of certain cages. For instance, Warmuth and co-workers used cavitand **80** and ethylene diamine to form water-soluble dynamic hexaimine cryptophane **81**, which was assembled in aqueous media in the presence of different templates. The cage formation mechanism takes advantage of the binding of the cryptophane derivative block to a range of guests, with van der Waals volumes ranging from 57 Å^3^ (CH_2_Cl_2_) to 118 Å^3^ (choline). The binding versatility of cryptophane was attributed to its ability to adapt to the size of the guest by wrapping/unwrapping motion around the polar C_3_ axis. The maximum binding strength was observed for CHCl_3_ (*K*_assoc_ = 8000 M^–1^). The guests that are too large in one dimension or more (e.g., acetophenone, toluene or [N(*n*-Bu)_4_]^+^) or are too reactive (i.e., 2,3-cyclohexenone) did not template the formation of cryptophane **81** (Figure 26).^75^

**Figure 27:**
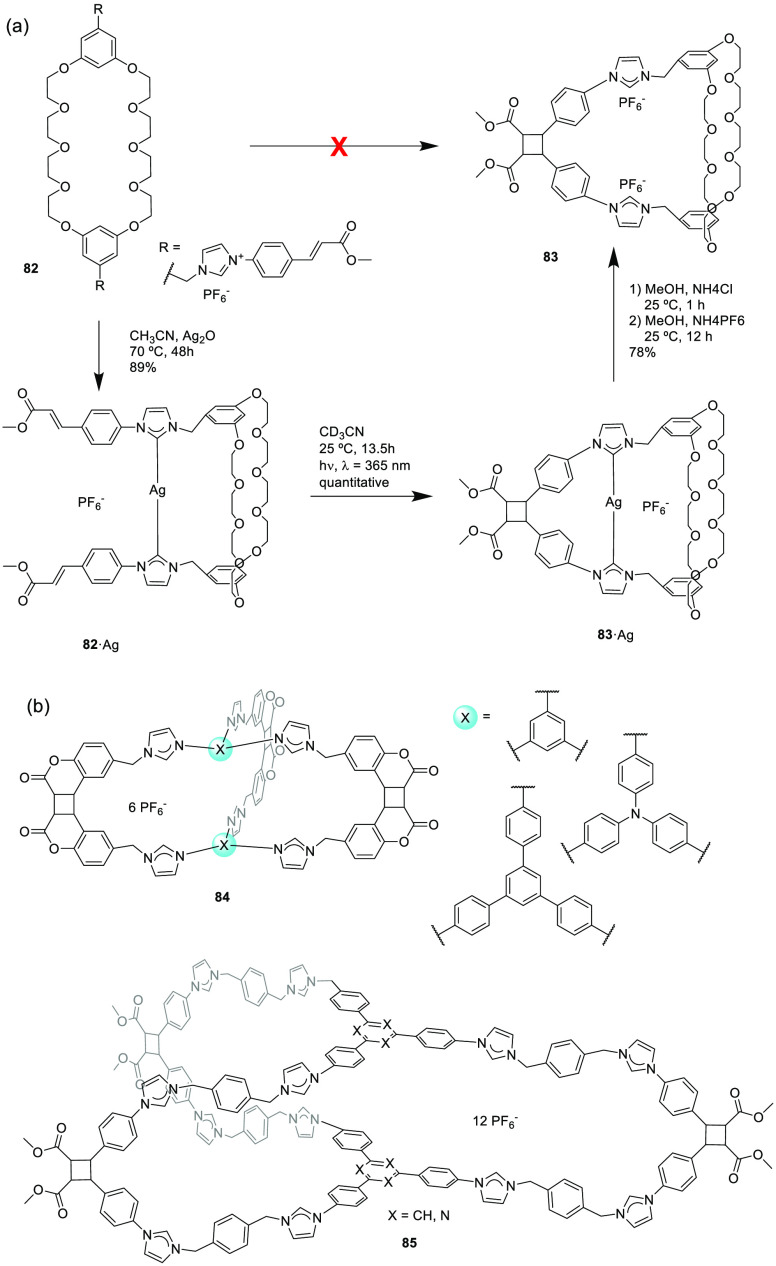
(a) Metal templated synthesis of cage **83** formation.^76^ (b) Examples of cages **84** and **85** prepared by metal templated synthesis.^77,78^

The postassembly modification of metal–organic assemblies provides an efficient strategy for synthesizing pure covalent organic cages that are otherwise difficult to synthesize by conventional direct methods. Han, Sun, and co-workers used macrocyclic precursor **82** to prepare cage **83**, which cannot be obtained by a direct reaction using a metal template strategy (Figure 27a). Synthesis involves the formation of a silver(I) carbene cage **82·Ag** from **82** with a 89% yield. Complex **82·Ag** is irradiated with a high-pressure mercury lamp to produce a quantitative photochemical, a [2 + 2] cycloaddition reaction, to yield metal–organic cage **83·Ag**. The demetallization of the complex generates covalent cage **83** with a 78% yield.^76^ The authors proved the versatility of this method for the synthesis of multiple cage structures, including cages **84** and **85** as representative examples (Figure 27b).^77,78^

### 2.2 Strategies for Achieving Water Solubility

Cage structures usually contain a large number of nonpolar groups that result in hydrophobic structures, which are soluble in organic solvents, but are not water-soluble in most cases. However, water insolubility limits the potential application of cages in aqueous media. One possibility to overcome this problem is to incorporate water-solubilizing groups, which are usually anchored to outer cage walls.

**Figure 28:**
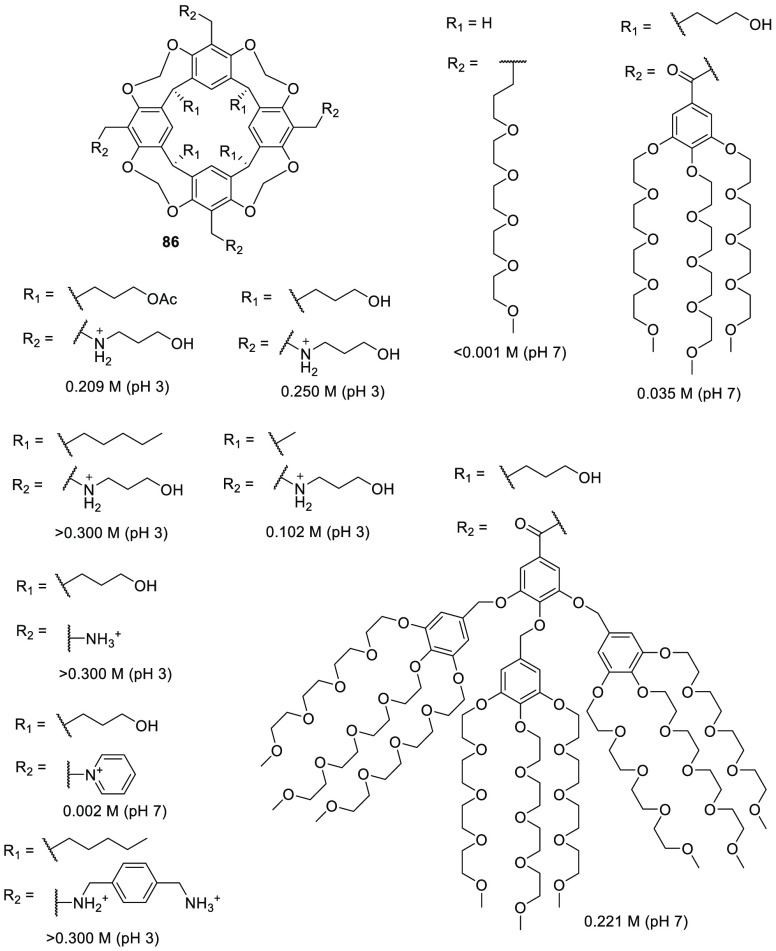
Water-solubilizing groups used in cavitand **86** with the maximum concentration achieved in water at a given pH.^79^

Following this approach, Verboom, Reinhoudt, and co-workers synthesized and determined the solubility of cavitand **86** by incorporating a wide range of water-solubilizing groups, including a series of charged groups and neutral hydrophilic tetraethylene glycol chains. The solubility of neutral cavitands with ethylene glycol increases with the number of ethylene glycol chains and decreases with temperature. The cavitands containing ammonium groups and lower-rim pentyl groups have the highest water solubility as a result of an amphiphilic character. The cavitands with pyridinium substituents and lower-rim methyl, hydroxypropyl, and propyl acetate substituents are still soluble in water but to a much lesser extent (Figure 28).^79^ Scherman, Bare, and Nikan achieved water solubility in the same cavitand motif structure by using phosphate groups in the R_1_ position and produced octa-anionic species.^80^

**Figure 29:**
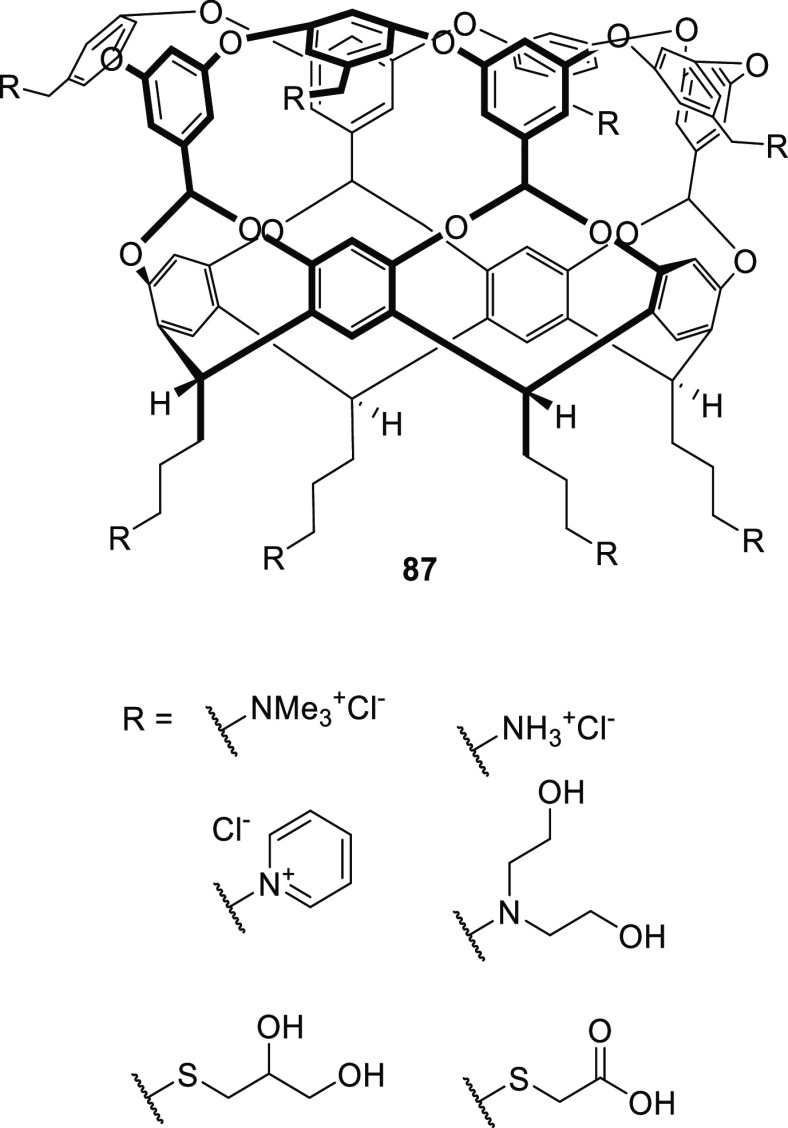
Water-solubilizing groups used in deep-cavitand **87**.^81^

Gibb and co-workers employed different cationic, anionic, and uncharged water-solubilizing groups to solubilize in water deep cavitand **87** (Figure 29).^81^ Additional examples of water-solubilizing groups (including carboxylates, ammonium groups, pyridinium groups, etc.) used in cavitands are described in Section 4 of this review.

### 2.3 Molecular Modeling

Computational approaches and molecular modeling have been used to predict cage structure and topology,^82^ cavity size, and related properties of large data sets of porous organic cages to guide synthetic researchers toward materials with the required properties. In fact the integration of computational and experimental fields is a promising tool that can significantly accelerate the discovery of new cage systems.^83,84^ In particular, rapid screening can provide a methodology in organic cages that could significantly help to develop materials with specific characteristics,^85^ whereas evolutionary algorithms can be used to determine the required size of precursors to achieve a given cage cavity size and properties.^86^

Of the different parameters that can be modeled, the shape persistence of organic cages can be predicted by modeling. To achieve this goal, Jelfs and co-workers prepared a large data set of 63472 organic cages with many topologies by employing an automated computational assembly from building blocks through several chemical reactions. The authors found that the cages obtained by imine condensation of trialdehydes and diamines in a [4 + 6] reaction were the most likely ones to form shape persistent cages. In contrast, thiol reactions were most likely to give collapsed cages. Using this library and machine-learning models, the authors were able to predict shape persistence with an accuracy of up to 93%.^87^

One of the main applications of porous materials based on organic cages is the separation of gas molecules. During this process, the diffusion of gases through the material is a key aspect that can also be modeled computationally based on the packing of molecular cages. In fact the solid-state porosity of organic cages is directly related to the packing of individual cages and the connectivity between cages because interconnected channels are necessary for guest molecules to pass through the material.

**Figure 30:**
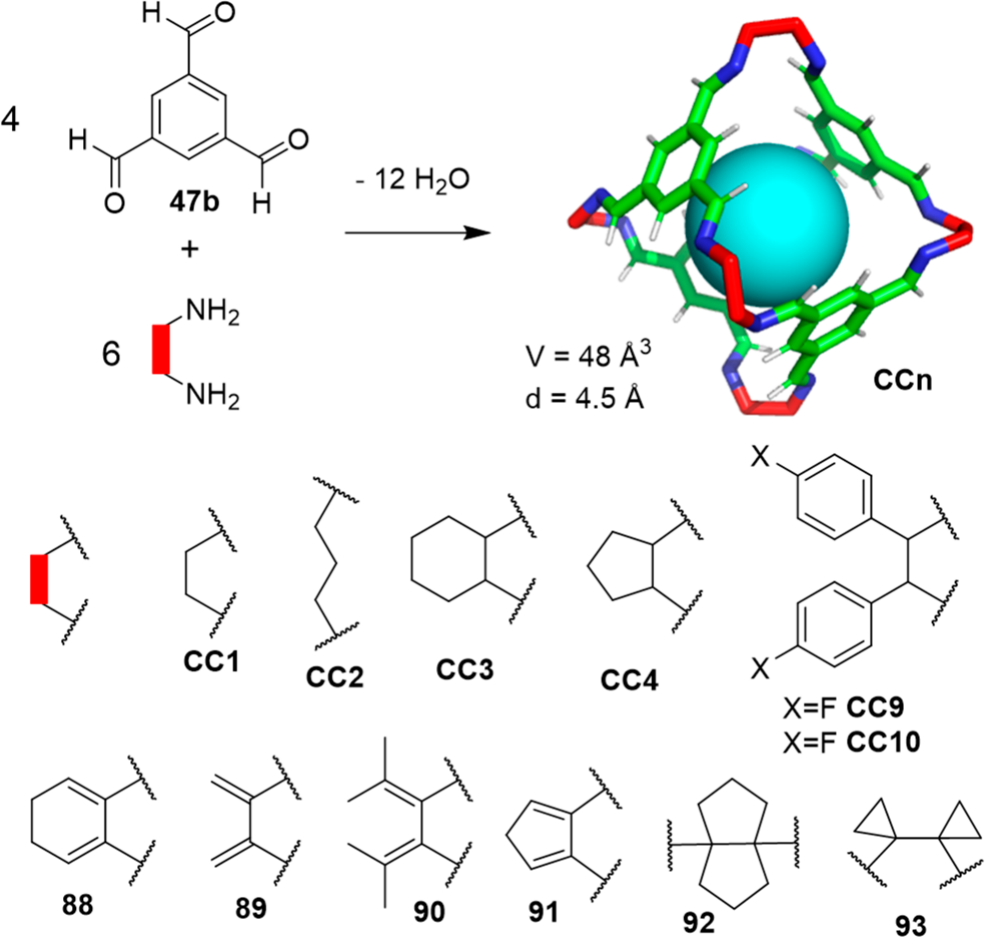
Synthesis of **CC***n* cages (top). Linkers employed to in silico synthesize (i.e., theoretically predicted structures) the new porous cages **88**–**93** (bottom). The largest cavity diameters for **88**, **89**, **90**, **91**, **92**, and **93** are 4.95, 5.30, 5.36, 4.97, 4.78, and 5.21 Å, respectively.^88^

On the basis of the core structure of cage **CC1**, it is possible to obtain a large family of isostructural cages (see structures **CC***n*, **88**–**93** in Figure 30) by changing the diamine building block. On the basis of this concept, by means of density functional theory (DFT) calculations and molecular dynamics (MD) simulations, Haranczyk, Bernabei, and co-workers investigated the thermodynamic stability of cages **CC***n*, which have been synthesized and their crystallographic data are summarized in Table 4. These cages have pore-limiting diameters within the 1.67–3.97 Å range, whose porosity is defined mainly by the diamine linker, and by the solvent used in the crystallization because it can yield different crystal polymorphs with a distinct porosity. By searching the PubChem3D database, which has ca. 80 million structures, the authors selected a set of six vicinal diamines that form porous organic cages with benzene-1,3,5-tricarboxaldehyde (**88**–**93**). The computational calculation allowed the determination of the solid-state crystal structure of cages **88**–**93** by theoretical calculations (note that cages **88**–**93** were predicted computation structures and were not, therefore, synthesized in a chemistry laboratory). The analysis of the resulting energy–structure–porosity maps showed structures with unusually high porosity. This computational approach constitutes the first step toward the high-throughput screening of porous organic cages for the assembly of functional materials (Figure 30).^88^

**Table 4:** Pore-Limiting Diameters and Densities of the Desolvated Crystal of the [4 + 6] **CC***n* Cages Developed by Cooper and Coworkers^88^^,^^a^

**CC***n* cage and CSD ref code	pore-limiting diameter (Å)	density (g cm^–3^)
**CC1β′** (ELALAF)	2.32	0.98
**CC1α′** (PUDWUH)	1.67	1.03
**CC2** (PUDXAO)	3.90	0.87
**CC3α** (PUDXES)	3.65	0.97
**CC3β** (PUDXES02)	3.41	0.92
**CC4α** (OZECAY)	3.57	0.93
**CC4β** (OZECAY03)	3.97	0.96
**CC9-R3** (GANDAC)	2.19	1.02
**CC10** (GANDUW)	2.78	1.17

a The Cambridge Structural Database references codes (CSD ref code) for each CC*n* cage are included.

**Figure 31:**
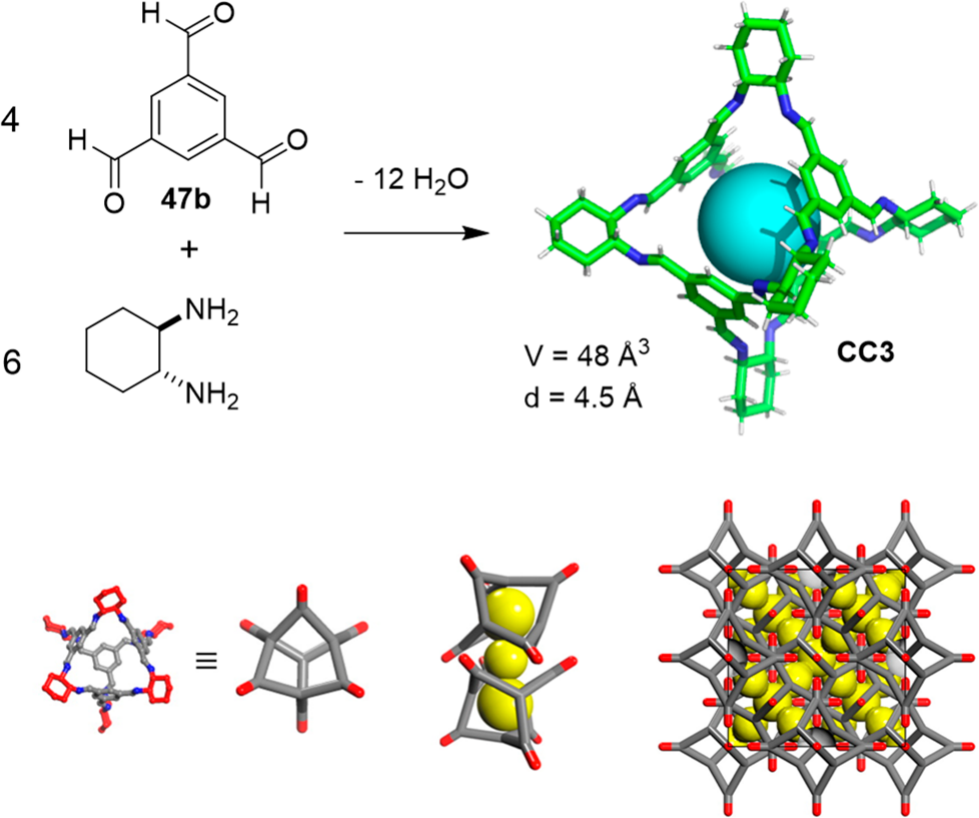
Porous organic cage **CC3** with a cavity of diameter 4.5 Å (top) and solid-state packing with the cage (dimers and 3D packing, bottom).^89^ Adapted with permission from ref (89). Copyright 2014 American Chemical Society.

Cooper and co-workers used molecular dynamics simulations to calculate the diffusion of six small gas molecules (H_2_, N_2_, CO_2_, CH_4_, Kr, and Xe) in flexible crystalline porous organic cage **CC3**. The experimental determination of the porosity of **CC3** (Figure 31) revealed a BET surface area of 624 m^2^/g (N_2_, 77 K).^57^ The modeling showed that the cage in the solid state has a dynamic cavity that undergoes size and shape fluctuations by playing a key role in the diffusion of molecules through solid material. This would explain that large gas molecules Kr and Xe, whose size was larger than the cage pore-limiting diameter (3.62 Å), could pass through. The simulations also predicted that the larger gas molecule SF_6_ was unable to diffuse in this materials’ pores (Figure 31).^89^ This method illustrates the consequences of cage flexibility, particularly the cage’s flexibility in the solid state, for the system’s porosity, rather than intermolecular displacements.

**Figure 32:**
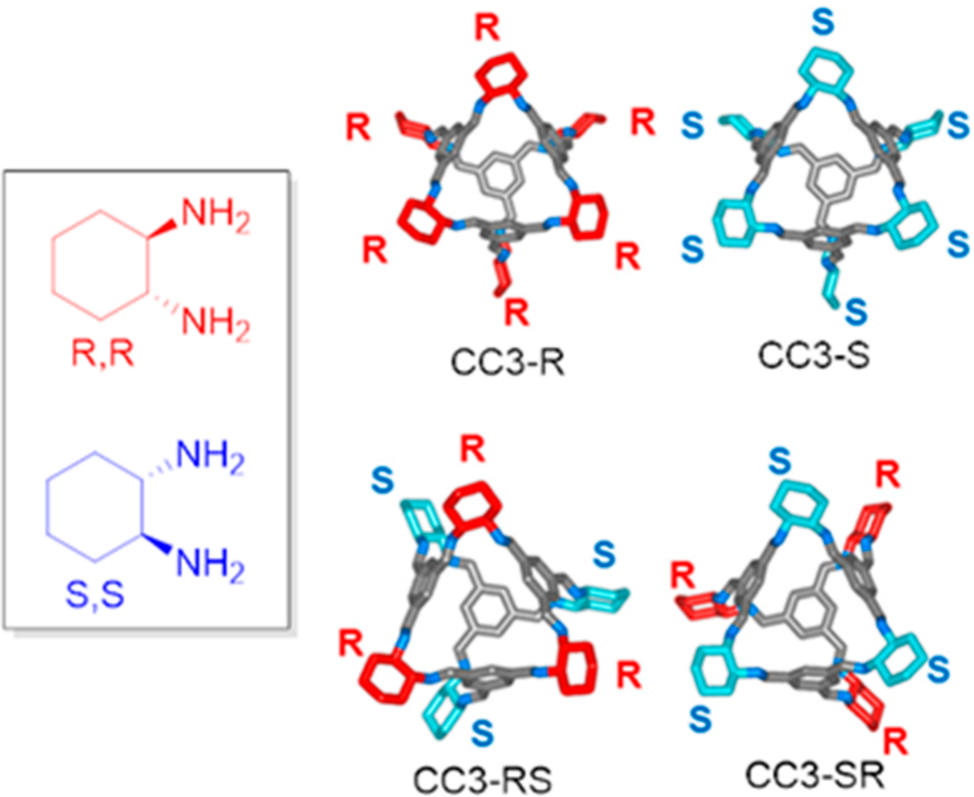
Two trans enantiomers (1*R*,2*R*)- and (1*S*,2*S*)- of diaminocyclohexane and the molecular models of the two types of **CC3** chiral cages.^90^ Adapted with permission from ref (90). Copyright 2019 American Chemical Society.

Whereas most of the materials based on organic cages, i.e., solid materials formed by organic cages, are built using cages with the same chirality. The materials built with cages with a different chirality are much less known. Sholl and co-workers performed molecular simulations of the **CC3**-racemic material to determine its structural properties because it is challenging to experimentally obtain the structure of this racemic crystal. They determined from the calculations that the composition of **CC3**-racemic material is made up of four distinct molecular species as so: two homochiral cages (**CC3**-*R* and **CC3**-*S*) and two heterochiral cages (**CC3**-*RS* and **CC3**-*SR*) in a minor proportion that varied from 8% to 28%. This computational information is valuable because it is not experimentally possible to obtain this information due to the differences that the distinct cage isomers produce in X-ray powder diffraction are too small to be quantified (Figure 32).^90^

**Figure 33:**
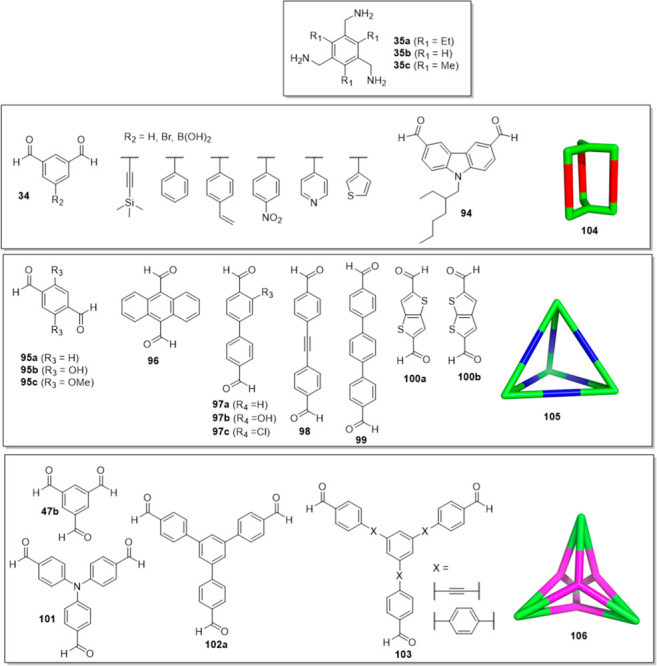
Cage precursors: triamines and three types of aldehydes to form capsules **104**, tetrahedrons **105**, and tetrapods **106**.^91^

Cooper, Jelfs and co-workers developed a hybrid discovery workflow that fuses computation with robotic synthesis to find new organic cage molecules. These authors studied 78 building block combinations (see structures **34**, **35**, **47b**, and **94**–**103** in Figure 33), which allowed 33 cages (32 of which were new) to be synthesized using a robot synthesizer in a one-pot reaction with no work up (see structures **104**–**106** in Figure 33). To increase the chances of obtaining a target cage predicted by the calculation, the cage must be strongly favored energetically. If cage formation is favored by a small energy quantity, other experimental factors, such as solvent stabilization, can change the outcome predicted by simulation and allow cages to be experimentally prepared that are not favored according to the simulation. This method is powerful because it helps to focus experiments by identifying linkers that are more reliable for cage formation. One example of building blocks and the corresponding cages is described in Figure 33.^91^ Cage structure **105** can be tuned by replacing terephthalaldehyde at any given ratio with electron-deficient and highly reactive tetrafluoroterephthalaldehyde. This substitution increases the stability and crystallinity of the obtained material in a stepwise manner without influencing crystal packing. This methodology can be used to fine-tune the cavity and to precisely control guest encapsulation selectivity.^92^

**Figure 34:**
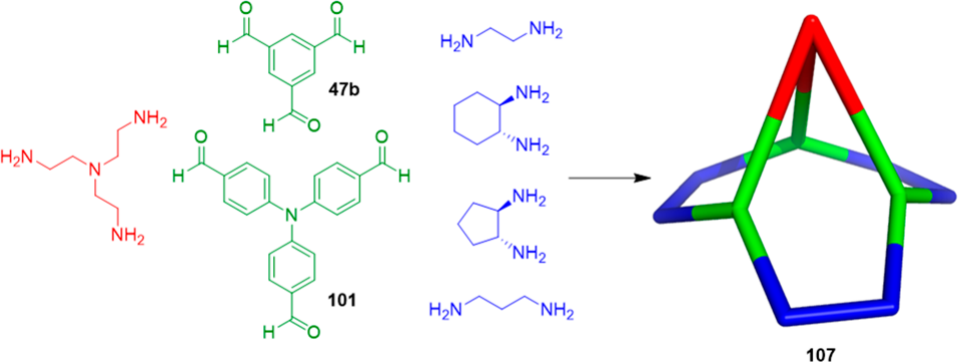
Design elements incorporated into the multicomponent cage pot **107**: cage window from **CC3**, capping triamine from **CC11**. Chemical structure of the precursors selected for screening and the schematic organic cage pot.^93^

Jelfs, Cooper, Day, and co-workers reported a powerful hybrid workflow that merges computations with experiments to guide the targeted discovery of new sophisticated supramolecular materials. This methodology allows the social self-sorting of a mixture of three components (triamine, diamine, trialdehyde; see Figure 34) to be predicted, unlike the methodology described in Figure 33, which involves only two components. The authors used this methodology to prepare organic cage pots **107** from three different components without exploiting orthogonal reactivities to direct the assembly. For this purpose, initial computational modeling was used to predict the synthetic viability of the organic cage pots by comparing the formation energies of the socially self-sorted organic cage pots and the formation energies of the separate binary organic cages, which would be obtained if narcissistic self-sorting happened (Figure 34).^93^ The authors indicated that these chiral organic cage pots have analogies to hemicryptophanes and, therefore, suggested that they have the potential to form guest–host complexes to encapsulate target guests.

**Figure 35:**
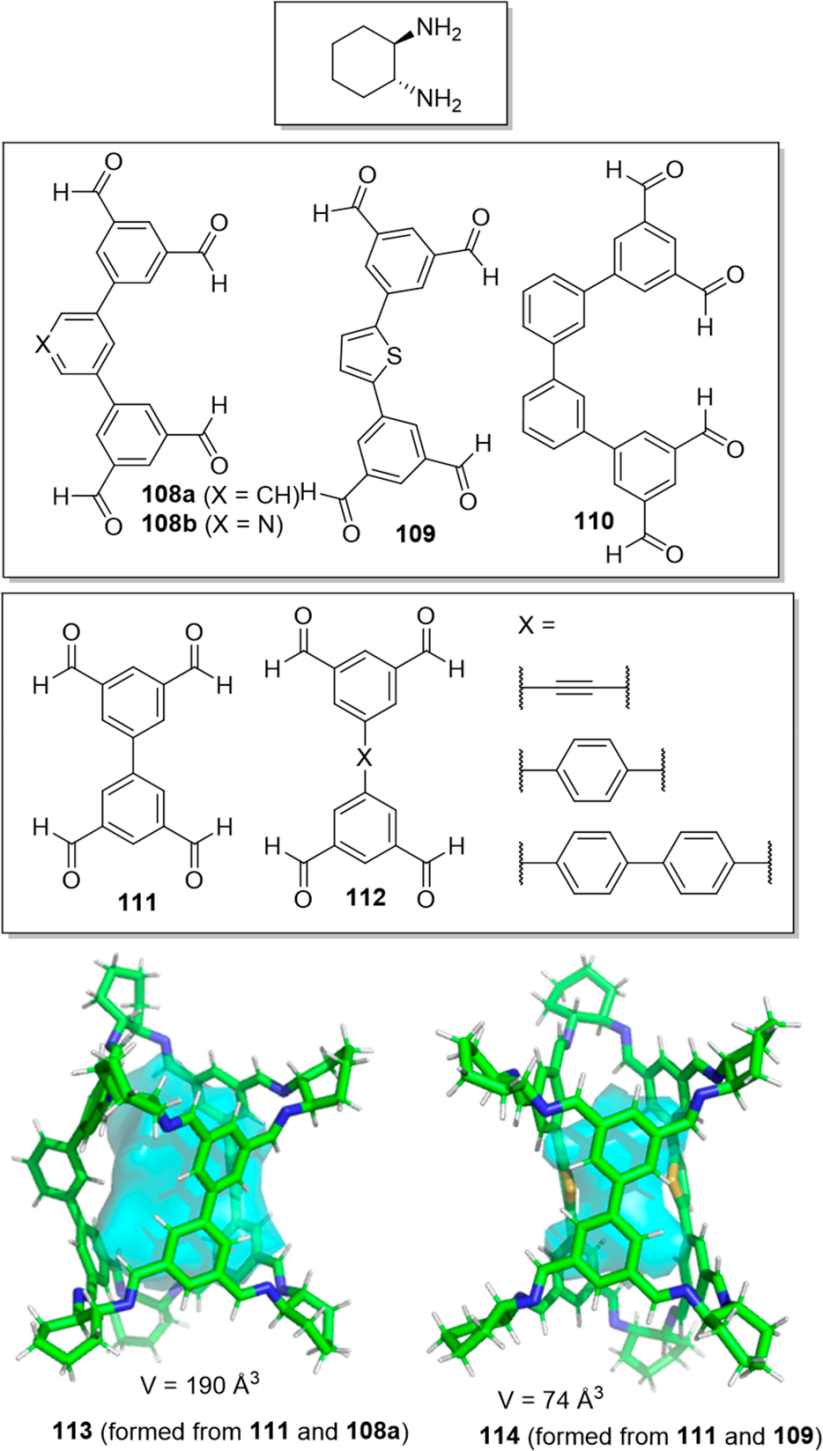
Building blocks and crystal structures of heteroleptic cages **113** and **114**. The crystallization of the cage formed by **111** and **108b** was unsuccessful.^94^

Slater and co-workers used computational modeling to rationalize the outcomes of the self-assembly of 1,2-cyclohexanediamine with a series of tetra-aldehydes **108**–**112** of different sizes and shapes. Experimentally, aldehyde **108a** did not self-assemble into high-symmetry cages but generated low-symmetry socially self-sorted heteroleptic cages [**111** + **108a**] (cage **113**) and [**111** + **108b**] and [**111** + **109**] (cage **114**). The computational modeling results agreed with the experiments because they predicted that these heteroleptic cages were more stable than the corresponding homoleptic cages (Figure 35).^94^

Although organic cages have been used as building blocks to prepare mechanically interlocked molecules,^95^ cage⊂cage complexes have not yet been synthesized. These interesting systems would have a central cavity whose size would correspond to the size of the guest cage cavity, which would allow confined space applications. The power of the molecular modeling methodologies developed by Jelfs and co-workers permitted them to model complexes in which one guest cage encapsulated into a host-cage cavity yielded a nested organic cage complex. The authors believed that the predicted feasible structures would help synthetic chemists to obtain the first nested cage structure.^96^

## 3 Cages and Containers Soluble in Organic Solvents and Their Applications

This section describes examples of cages and containers soluble in organic solvents and also includes solid materials based on molecular cages. The section is divided into different subsections based on the type of container and on the type of reaction employed to self-assemble the cage. Cage properties are described in detail by focusing on the several geometries with the distinct cavity sizes that can be obtained by using slightly different building blocks. Most of the examples described in this section have been used as host systems for the encapsulation of different guest molecules, and their host–guest properties are also described. In some examples, cages have been employed to host gas molecules. The corresponding gas sorption experiments are also described, especially examples that link together porosity in the solid state with the flexibility and rigidity of cage molecules; i.e., rigid cages display solid-state porosity and flexible cages are not porous in the solid state.

### 3.1 Cavitands, Hemicarcerands, Carcerands, and Cryptands

**Figure 36:**
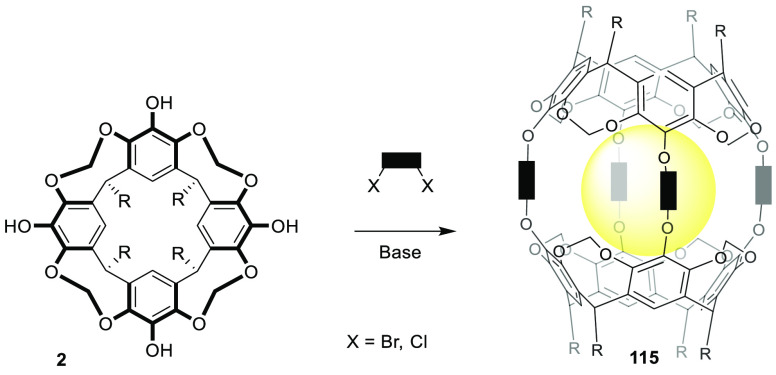
General synthesis of carcerands and hemicarcerands from a cavitand building block.

The term cavitand was formulated by Cram in 1982 to define molecules with a cavity for hosting guest molecules. Since then, the expansion of this field has given rise to the development of numerous cavitands types.^97−99^ Such systems have allowed the preparation of carcerands and hemicarcerands by putting the convergent geometry of cavitands to good use, which makes them ideal building blocks to prepare hollow molecular structures.^100,101^ Of the different structures, calixarenes are one of the most widely used cavitand motifs. One of the simplest carcerand structures (**115**) can be obtained by the condensation of two calixarenes **2** with two difunctional spacers (Figure 36).

**Figure 37:**
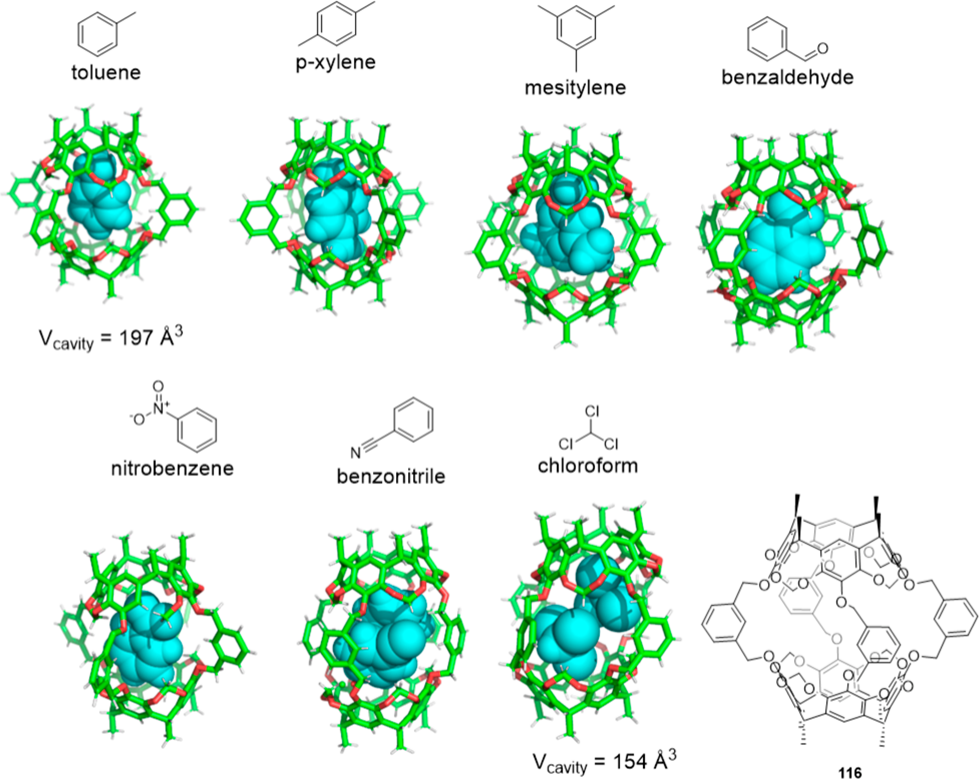
Crystal structures of the host–guest complexes of adaptive resorcinarene hemicarcerand **116**. Top left to right: toluene (CCDC: 2047577), *p*-xylene (CCDC: 2047581), mesitylene (CCDC: 2047578). Bottom left to right: benzaldehyde (CCDC: 2047582), nitrobenzene (CCDC: 2047576), benzonitrile (CCDC: 2047580), chloroform (CCDC: 2047579). Comparison of calculated cavities.^102^ CCDC: Cambridge Crystallographic Data Centre numbers.

Hemicarcerand structures based on resorcinarenes present a combination of rigid resorcinarene moiety and linker building blocks, which can have different degrees of flexibility. Dyker and co-workers showed that resorcinarene hemicarcerand **116** can adapt its shape and size according to guests’ steric demand by aligning its guests to match the complementary electrostatic potential. By way of example, the volume of the cavity in **116** changes from 197 to 154 Å^3^ when toluene and chloroform guests are, respectively, encapsulated (Figure 37).^102^

**Figure 38:**
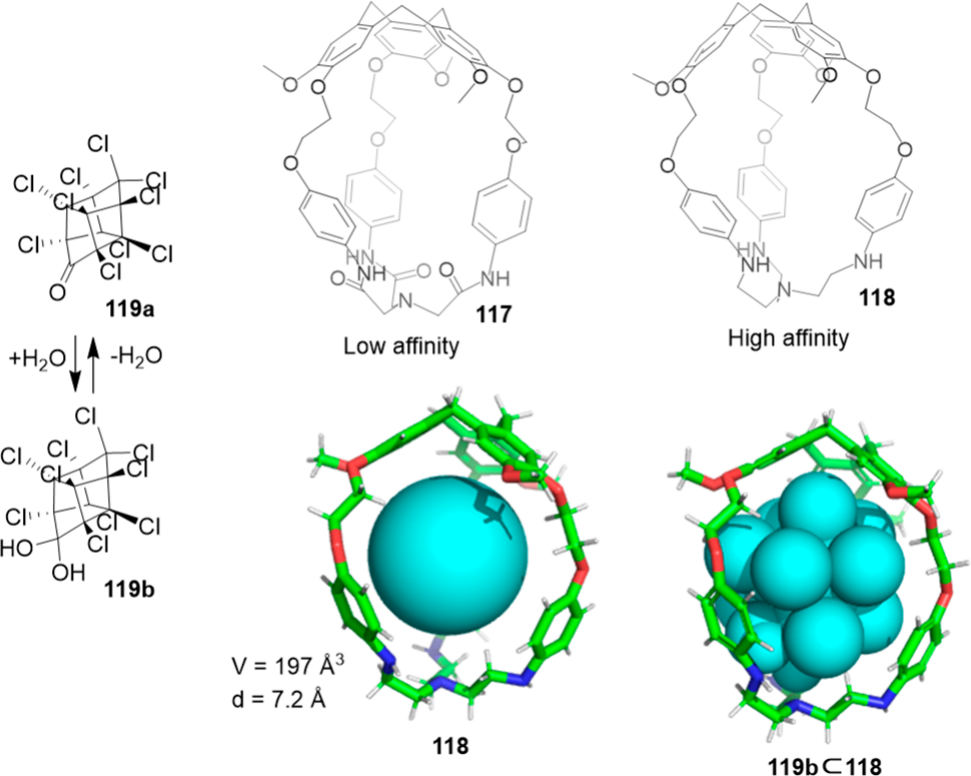
Chemical structure of hemicryptophanes **117** and **118**. Encapsulated hydrated chlordecone (**119b**) in hemicryptophane **118**. Equilibrium of chlordecone (**119a**) and hydrated chlordecone (**119b**).^103^

Martínez, Saaidi, and co-workers showed that subtle differences in the functional groups that envelope the cavity of cages **117** and **118** play a key role in defining affinity to encapsulate a certain guest. This is nicely demonstrated in the encapsulation of organochlorine insecticide chlordecone **119** by **117** and **118**. Whereas hemicryptophane **117**, which contains amide groups, displays a moderate binding constant of 126 M^–1^, receptor **118**, which contains amine groups, shows a much higher association constant of 2.1 × 10^4^ M^–1^. The structure of the latter complex is stabilized by the hydrogen bonding between the geminal diol of **119b** and the nitrogen atom of cage amino groups and by the halogen−π interactions between the guest chlorine atoms and hemicryptophane aromatic rings (Figure 38).^103^

Cavitands that incorporate water-solubilizing groups have been widely used as molecular hosts in water to put the hydrophobic nature of the resulting cavity to good use. Numerous examples are included in section 4.1 of this review.

**Figure 39:**
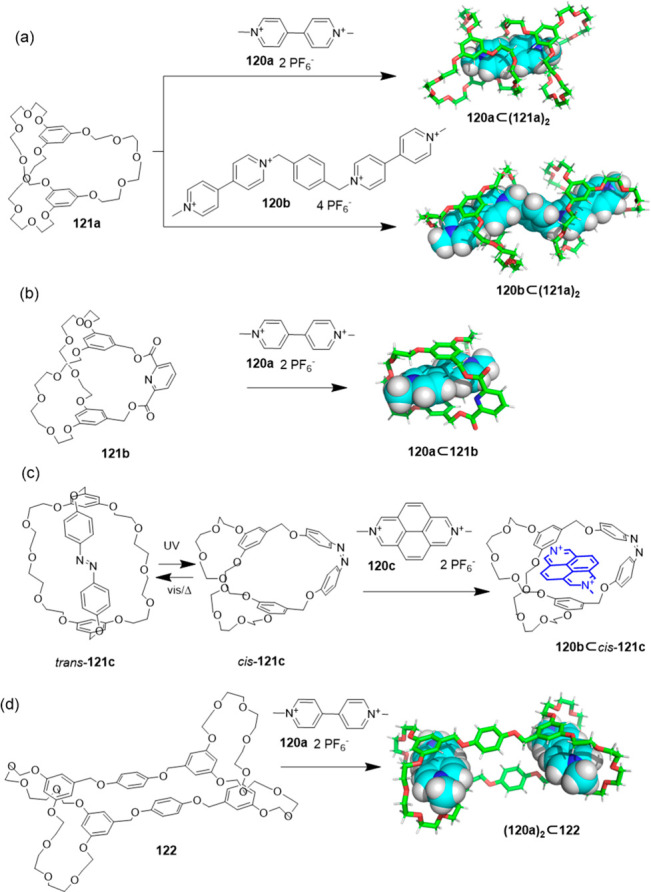
Encapsulation of paraquat derivatives **120** by cryptands **121** and **122**. (a) Formation of [3]pseudorotaxanes.^111,112^ (b) Formation of 1:1 host–guest complexes.^114^ (c) Azobenzene containing cryptand with a *cis*–*trans* isomerism that switches binding ON/OFF.^117^ (d) Formation of 1:2 host–guest complexes using a host with two cryptand cavities.^118^

Cryptands are also an important class of organic cages, which show high binding affinity to guest molecules, especially paraquat derivatives.^104^ These host–guest properties allow the formation of pseudorotaxanes,^105−107^ rotaxanes,^108^ catenanes,^108,109^ and polymers.^110^ The complexation of paraquat **120a** by cryptand **121a** results in a [3]pseudorotaxane host–guest complex, where the guest is encapsulated in the cavities of two cryptand molecules stabilized by multiple face-to-face π-stacking interactions.^111^ Using the same cryptand **121a** and bisparaquat guest **120b**, a [3]pseudorotaxane is formed by cooperative complexation with association constants *K*_1_ = 1.2 × 10^3^ M^–1^ and *K*_2_ = 2.0 × 10^4^ M^–1^ (Figure 39a).^112,113^ Introducing a pyridine moiety into one of the bridges of **121a** yields cryptand structure **121b**. This cryptand forms a 1:1 host–guest complex with paraquat **120a** (Figure 39b).^114−116^ Cryptand **121c** is obtained by introducing an azobenzene into the bridge. *Trans* and *cis* conformations are controlled by irradiation with UV light (to form the *cis* isomer) or visible light or by heating (to form the *trans* isomer). This isomerism allows switching the binding with 2,7-diazapyrenium **120c** between OFF for the *trans* isomer and ON for the *cis* isomer (Figure 39c).^117^ The more elaborated geometry of host **122**, which has two cryptand cavities, is able to encapsulate two paraquat molecules **120a**, as determined by ^1^H NMR and the single-crystal X-ray structure, which highlights the versatility of cryptands in sophisticated host structures (Figure 39d).^118−120^

### 3.2 Capsules and Cages

#### 3.2.1 Imine

As described in section 2.1, imine chemistry is one of the most well-established reversible reactions in cage synthesis. The versatility of imine bond formation from amines and aldehydes has allowed the preparation of numerous cage structures since the first reported example in 1991 by Cram and Quan.^17,51,121^ In this section, we describe representative examples of different families of organic cages based on imine chemistry as reported in the literature.

**Figure 40:**
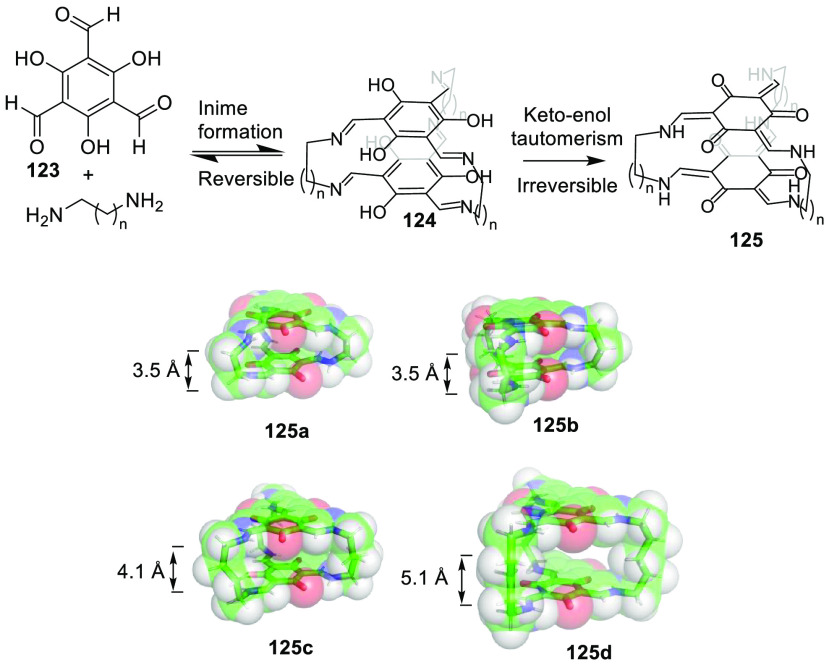
One-pot synthesis of imine-linked organic cages **125**.^122^

Cages can be prepared with customized cavity volumes, from small to large, by changing the size of the building blocks that preserve the same cage structure type; for example, [2 + 3] cages from difunctional and trifunctional building blocks with complementary reactivity. Cages with very small cavities have been reported by Banerjee and co-workers, who performed a one-pot synthesis of amine-linked organic cages **125** from **123** and a diamine through cage intermediate **124** (Figure 40). The procedure additionally removed the complications associated with the postsynthetic modifications of imine-linked porous organic cages to obtain amine cages by reduction. In the prepared cage structures, an increasing chain length resulted in an odd–even alternation between the eclipsed and gauche conformations of cages by controlling cage conformation and cavity size. Synthesized cages **125** in the solid state had moderate porosity for **125d** (202 m^2^ g^–1^) and **125c** (87 m^2^ g^–1^), whereas **125a** (17 m^2^ g^–1^) and **125b** (23 m^2^ g^–1^) were nonporous. These cages had excellent and much better chemical stability in water, acids, and bases than imine-linked porous organic cages.^122^

**Figure 41:**
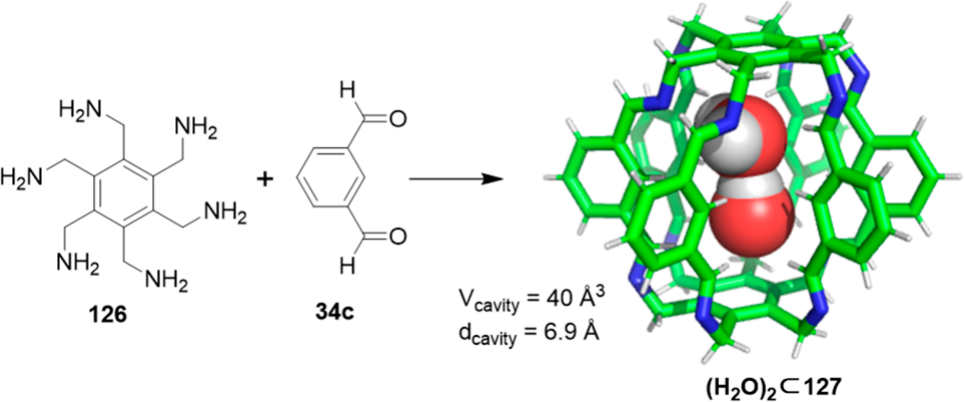
Synthetic route of superphane **127** with the crystal structure containing two encapsulated water molecules.^123^

Cages whose size was slightly larger had enough space to allocate small solvent molecules in their cavity. He and co-workers reported a [2 + 6] one-pot synthesis that involved *m*-phthalaldehyde **34c** and hexakis-amine **126** building blocks to yield a superphane cage **127** by imine bond formation. The synthesis required heating to favor the formation of the cage product in DMSO. The cage cavity was able to encapsulate two water molecules to form water dimers stabilized by multiple hydrogen bonds with the cage (Figure 41).^123^

**Figure 42:**
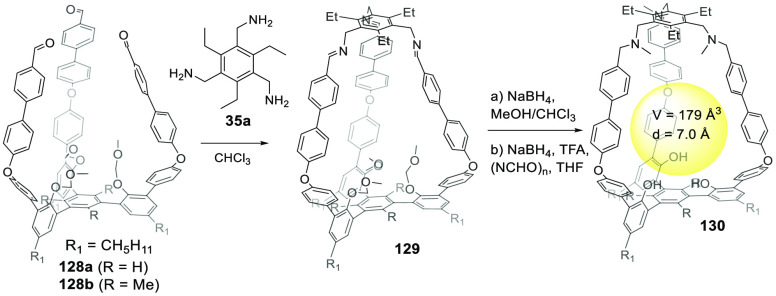
Synthesis of the phenol hydroxyl groups’ endofunctionalized cage **130**.^124^

In contrast to the above examples, which show a relatively small cage volume, the use of larger building blocks allowed bigger cavity sizes to be acquired. For instance, Chen and co-workers used building blocks **35a** and **128** to prepare cage **129** by imine bond formation. Reduction of imine bonds, deprotection of phenol hydroxyl groups and methylation of amine groups yielded the final cage **130** with three endohedral phenol hydroxyl groups. Cage **130** was used as catalyst in a Friedel–Crafts reaction and displayed a moderate catalytic effect (Figure 42).^124^

**Figure 43:**
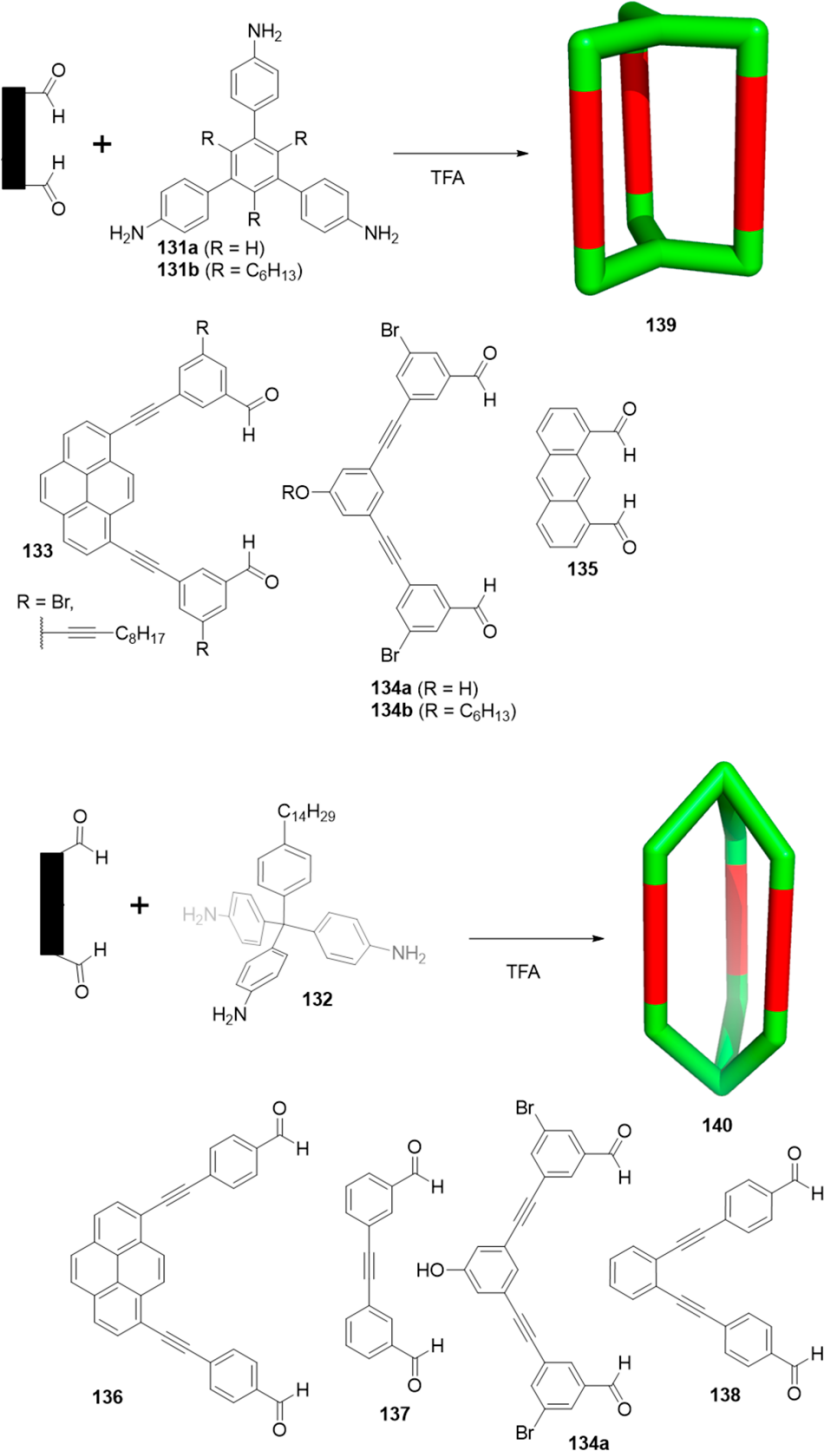
Synthesis of [2 + 3] cages **139** and **140** obtained by the condensation of different triamines and dialdehydes.^126^

In addition to the selected examples of the above-described [2 + 3] cages, tris-primary amines, aliphatic and aromatic, have been extensively used to prepare cage structures. Some of these triamines are commercially available and others can be prepared by following the synthetic protocols in the literature.^125^ Zhang and co-workers showed that a complementary geometry between triamine **131** (planar) or **132** (pyramidal) and dialdehydes **133**–**138** is key for obtaining cage structures. These authors suggested that the angle and directionality of reactive groups are essential for achieving an alignment of the aldehyde and amino moieties in the building blocks with an appropriate angle. Additionally, rotational freedom in building blocks can reduce the angle strain established by cage formation and, hence, facilitate the synthesis of cages **139** and **140** (Figure 43).^126^

**Figure 44:**
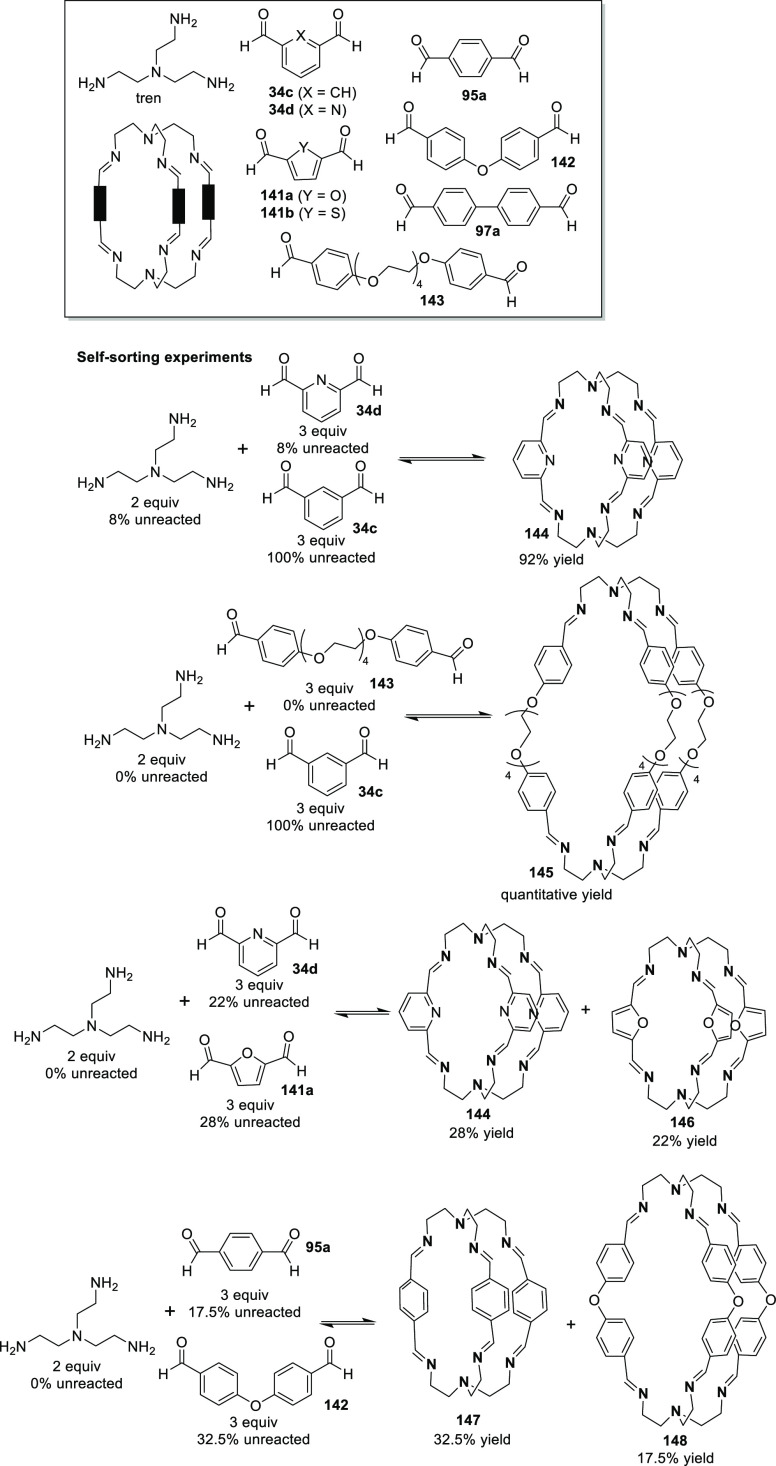
Reaction schemes of “tren” with different dialdehyde building blocks showing the product distribution upon equilibrium (cages **144**–**148**).^127^

Further examples of [2 + 3] cages were prepared by Lehn and co-workers, who demonstrated that triamine “tren” (tris(2-aminoethyl)-amine) with different dialdehyde building blocks (**34c**, **34d**, **95a**, **97a**, **141**, **142**, **143**) showed significant self-sorting behavior in the formation of imine-based macrobicyclic cryptand-type organic cages (**144**–**148**). Using eight different dialdehyde building blocks, the authors prepared the corresponding cages. Afterward, self-sorting experiments run with one aldehyde building block or several revealed high selectivity toward the formation of some cages over others, which highlights the influence of the aldehyde structure. The presence of a heteroatom kinetically and thermodynamically favors the formation of cages **144**. This is highlighted in a competitive self-sorting experiment of dialdehydes **34c** and **34d** with “tren”, which exclusively yields cage **144** with a 92% yield (Figure 44). The flexibility of components also plays a key role in self-sorting, as highlighted in the self-sorting of a mixture containing **34c**, **143**, and “tren”, which yielded the homoleptic cage **145** in a roughly quantitative yield. For other combinations of aldehydes, selective cage formation has not been observed; for example, in the case of a mixture of either **34d**, **141a**, and “tren” or **95a**, **142**, and “tren”, the formation of a mixture of two cages has been observed. More complex self-sorting experiments using a mixture of six dialdehyde components has pointed out that the presence of a heteroatom in the aldehyde aromatic ring favors cage formation. Additionally, intramolecular hydrogen-bonding interactions, and the antiparallel orientation of dipole groups, also play a key role in cage formation (Figure 44).^127^

**Figure 45:**
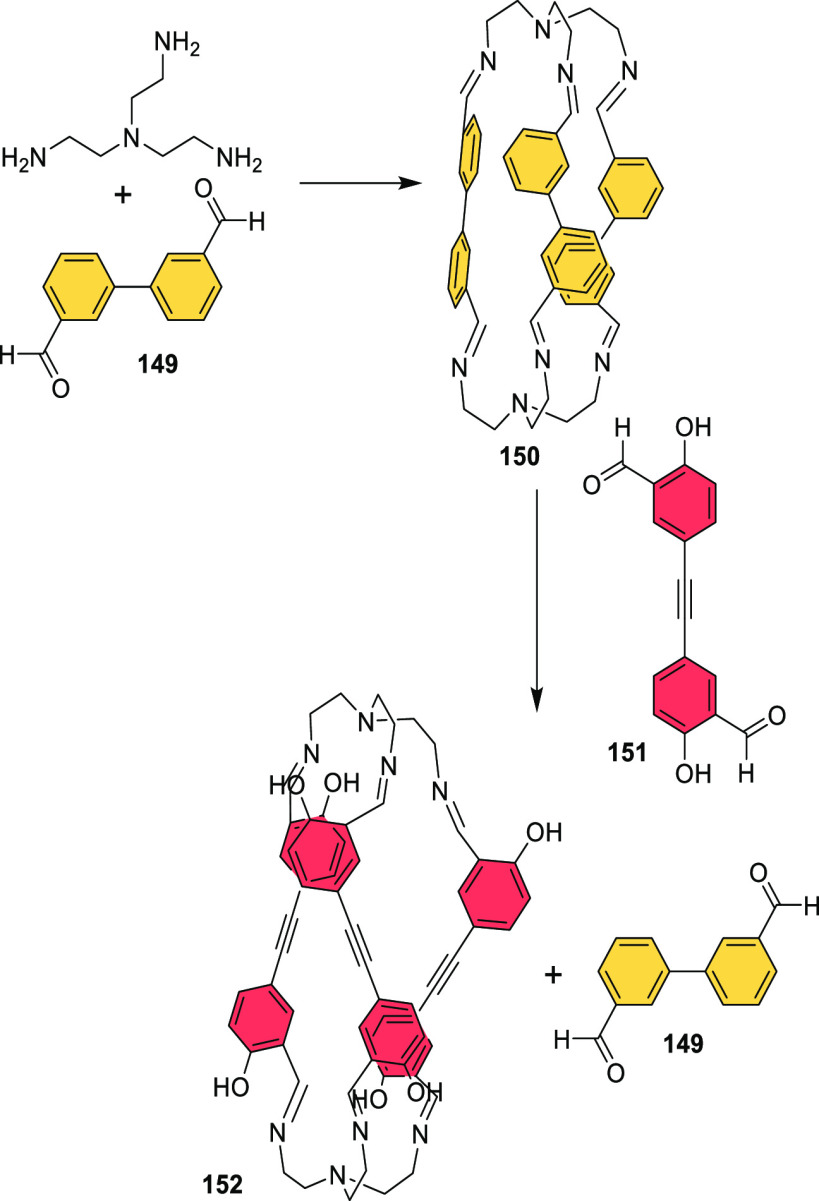
Synthesis of cage **150** followed by the hydrogen-bond-driven transformation into cage **152**.^128^

By following the same approach based on using “tren” and different dialdehyde building blocks, Mukherjee and co-workers showed that intramolecular H-bonding plays a key role in the selective formation of imine organic cages. In particular, they used dialdehyde **149** and “tren” to prepare cage **150**, and dialdehyde **151** and “tren” to prepare cage **152**. The treatment of cage **150** with aldehyde **151**, which contains phenolic groups, displaced aldehyde **149** from the original cage to yield cage **152**. The authors found that the reaction was driven by intramolecular H-bonding stabilization (Figure 45).^128^

**Figure 46:**
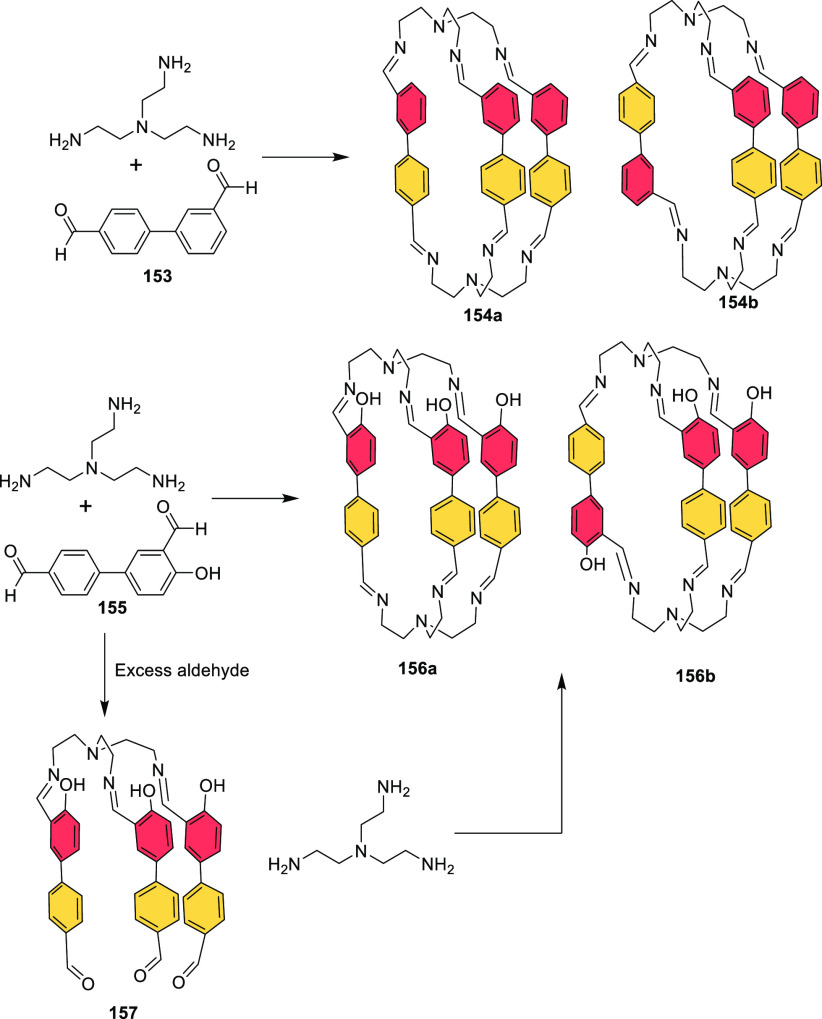
Possible isomeric cages (**154**, **156**) that can form in the [3 + 2] self-assembly of an unsymmetrical aldehyde (**153**, **155**) and a flexible amine.^129^

Mukherjee and co-workers employed related systems to report the selective formation of a single isomer of a [3 + 2] self-assembled organic cage from an unsymmetrical dialdehyde and the flexible triamine “tren”. The reaction of “tren” with aldehyde **153** exclusively formed single isomer **154b**. In contrast, when “tren” reacted with aldehyde **155** under the same reaction conditions, a mixture of isomeric cages **156a** and **156b** was obtained. The same results were found if the reaction was performed from intermediate **157**. The only difference between aldehydes **153** and **155** was one hydroxy group and, therefore, intramolecular hydrogen bonding with the nearby formyl or imine groups played a key role in the observed isomer selection differences. The experimental results indicate that the formation of a single cage isomer depends on the shape and size of the building aldehyde, while theoretical calculations suggest that the energy difference between isomers is the key factor in isomer selection (Figure 46).^129^

**Figure 47:**
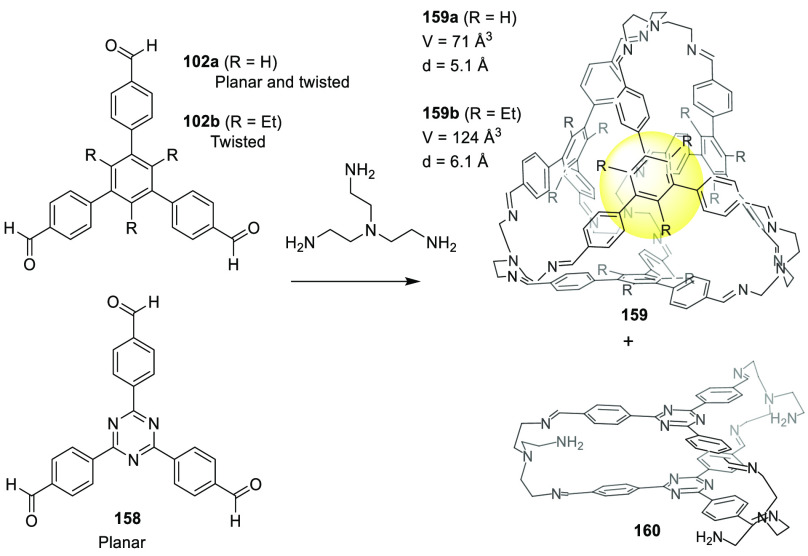
Self-sorting reaction that yields tetrahedrons **159** and prisms **160**.^130^

The previous examples show the formation of [3 + 2] cages by the reaction of di- and trifunctional building blocks of complementary reactivity. In addition to this topology, [4 + 4] tetrahedral cages can be obtained from trifunctional building blocks with complementary reactivity. The reaction of “tren” with trialdehyde building blocks with the appropriate geometry results in the formation of tetrahedral cages, with “tren” units placed in corners and trialdehyde moieties on the tetrahedron faces. By following this concept, Li and co-workers prepared tetrahedral cages and triangular prisms using a trialdehyde and “tren” and found that the self-assembly process was driven toward the products that maximized the intramolecular π–π and CH−π interactions. The conformational preferences of trialdehyde building blocks **102** and **158** also impacted the self-assembly outcome. While a twisted conformation (**102b**) favored the formation of tetrahedron **159**, rigid and planar building blocks **158** favored the formation of prism **160**. For trialdehyde **102a**, which had more conformational freedom and could adopt both twisted and planar conformations, both tetrahedral **159** and prismatic **160** cages were obtained with good yields (Figure 47).^130^ Tetrahedral cage **159** efficiently encapsulated white phosphorus (P_4_) by reducing its intrinsic reactivity toward oxygen. In this complex, each phosphorus atom in P_4_ pointed toward the adjacent central **102b** phenyl ring to produce a host–guest orbital overlap. This interaction could be responsible for the stability of the supramolecular complex.^131^

**Figure 48:**
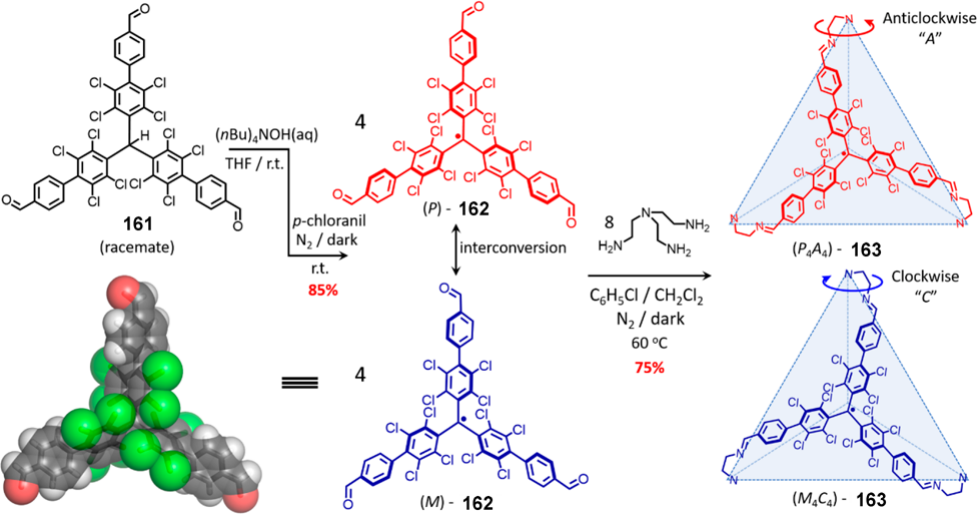
Synthesis of tetrahedral PTM cage **163**.^132^ Reproduced with permission from ref (132). Copyright 2021 Wiley-VCH.

Li, Cao, Jiao, and co-workers adopted the same strategy to obtain a tetrahedral cage structure. The synthetic route used building block **161** to prepare polychlorotriphenylmethyl (PTM) radical units **162**. This building block was reacted with “tren” to obtain the [4 + 4] tetrahedral cage **163**. Cage formation was favored by the intramolecular CH···π and hydrogen-bonding interactions in the cage structure, which also favored the stability of the cage framework’s homochirality. In each cage structure, the PTM subunits adopted the same helical conformation (all P or all M), and the “tren” vertices all had the same clockwise (C) or anticlockwise (A) orientation. This resulted in chiral structures P_4_A_4_ or M_4_C_4_ cages. The magnetic measurements showed that each cage had 3.58 spins and the four PTM radicals present in the cage were weakly coupled through space with a distance between the two central PTM radical carbon atoms of 9.74 Å (Figure 48).^132^

**Figure 49:**
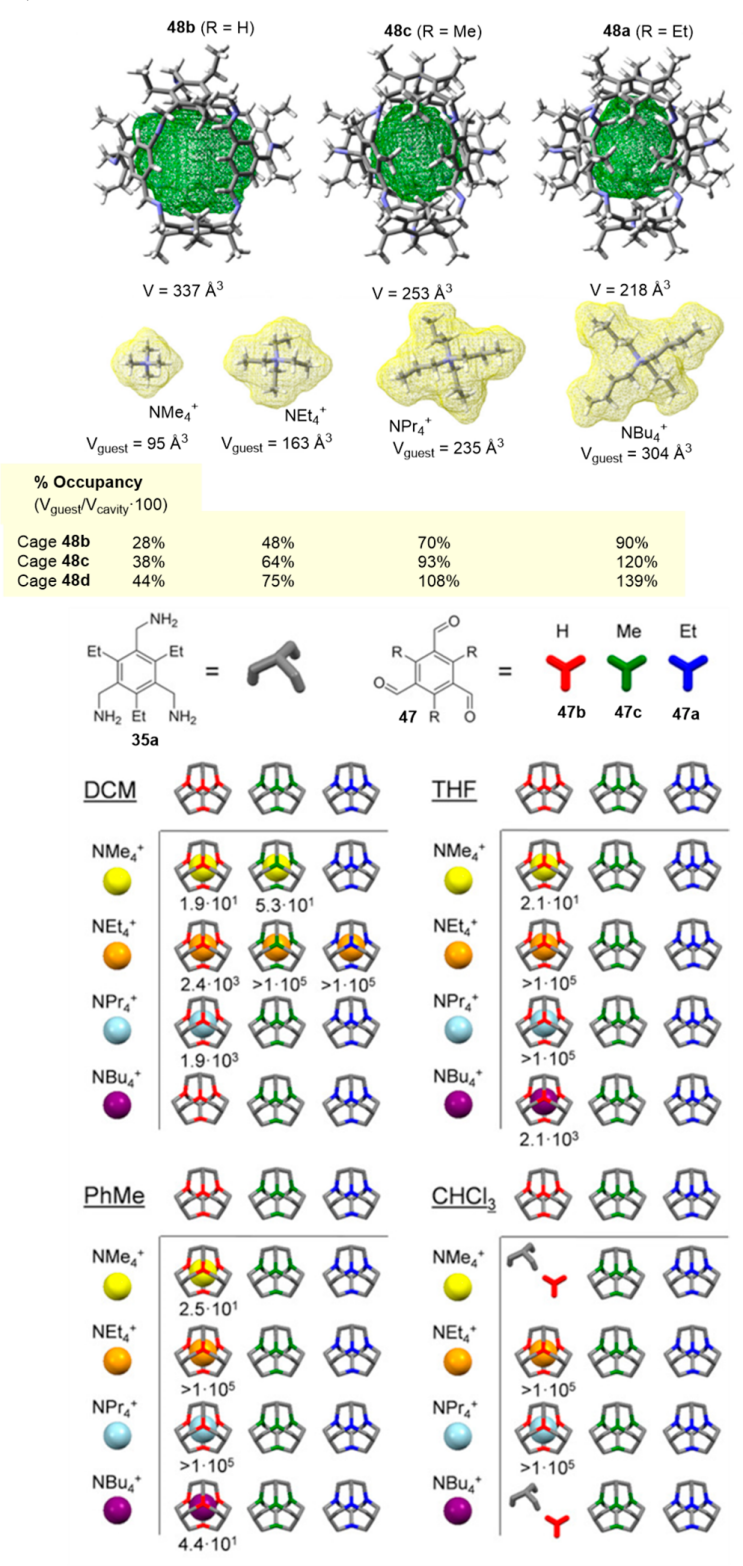
Structures of cages **48** and the binding experiments with guests NMe_4_^+^, NEt_4_^+^, NPr_4_^+^, and NBu_4_^+^. Size selectivity with association constants *K*_assoc_ (M^–1^) in different solvents.^133^ Adapted with permission from ref (133). Copyright 2020 Wiley-VCH.

Replacing “tren” with 1,3,5-tris(aminomethyl)-benzene **35a** and performing the reaction with trialdehyde building blocks **47a**–**47c** resulted in the formation of the [4 + 4] tetrahedral cages **48** (see Figure 14). Following this approach, Mastalerz and co-workers prepared shape-persistent tetrahedral imine cages **48**, which had the same structure but different substituents, and all with similar cavity volumes and window sizes. They used these cages to study the encapsulation of tetra-*n*-alkylammonium cations.^50,133^ The thermodynamic and kinetic results showed that guest uptake was highly solvent-dependent and suggested that it was favored by a squeezing mechanism, in which the cage remained intact and the guest entered the cage cavity through the window rather than by a gate-opening mechanism through imine bond cleavage. Guest encapsulation involved folded chain conformations, and guest packing coefficients of >65%, led to restricted guest movement in the cage cavity (Figure 49).^133^

**Figure 50:**
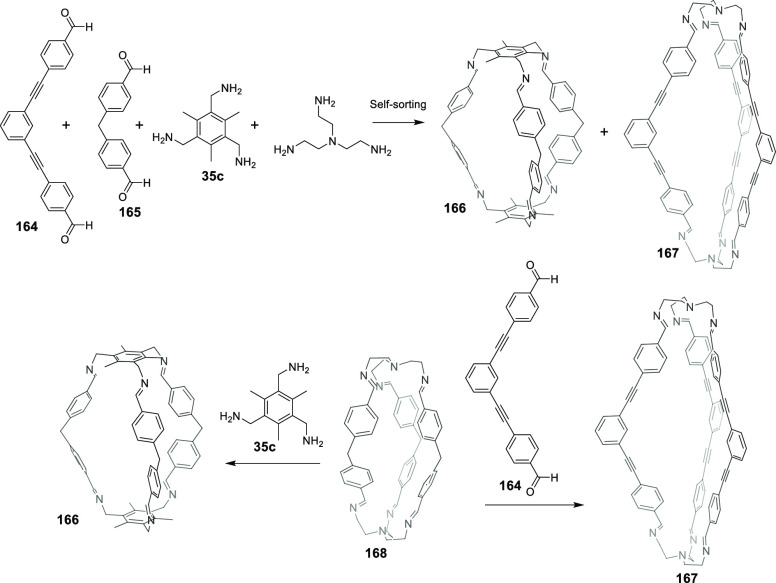
Molecular marriage through partner preferences. Self-sort cage formation (**166** and **167**) with dialdehyde building blocks **164** and **165** and triamines **35c** and tren.^134^

The different properties of “tren” and 1,3,5-tris(aminomethyl)-benzene **35c** have been used to selectively self-sort cage formation with dialdehyde building blocks **164** and **165** by Mukherjee and co-workers. Of a mixture of triamines “tren” and **35c**, and dialdehydes **164** and **165**, only two cages formed (**166** and **167**). In contrast, when components were separately assembled, it was possible to obtain the four corresponding different cages. The two nonpreferred cages could be transformed into the two preferred combinations by reacting them with the appropriate triamine or dialdehyde (Figure 50 shows an example of the transformation of cage **168**). The authors indicated that the behavior of these dynamic systems could hardly be rationalized from the individual component’s behavior (Figure 50).^134^

**Figure 51:**
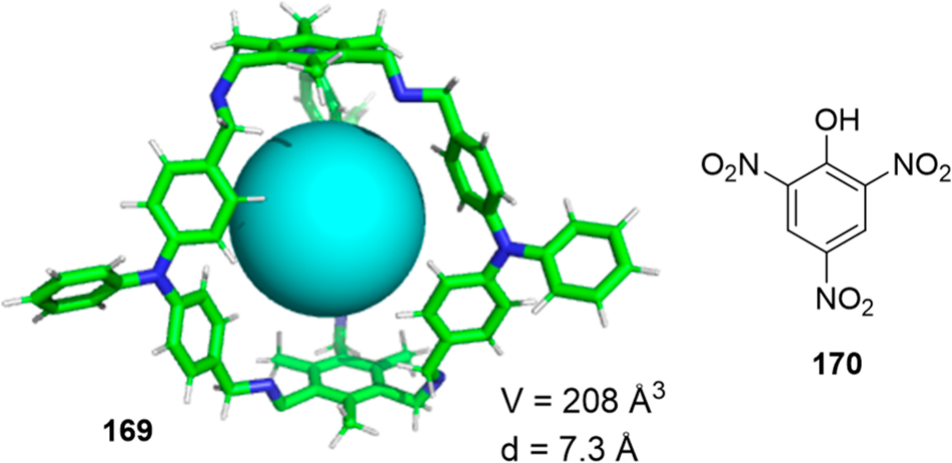
Fluorescent organic cage **169** used for picric acid (**170**) sensing.^135^

On the basis of the [2 + 3] cage shapes described in Figure 50, with a suitable cavity to encapsulate guest molecules, Mukherjee and co-workers prepared fluorescent cage **169** to detect explosive picric acid (**170**). Sensing of picric acid took place by quenching the cage’s fluorescence emission, which was reduced by 94%. Picric acid encapsulation allowed this explosive to be detected at a concentration as low as 6.4 ppb. Guest binding took place by the secondary amine groups of the cage that interacted with the phenol group of picric acid. This interaction resulted in the proton transfer from the acidic phenolic OH group to the basic amino group to form a strong ion pair picrate–protonated cage. The probe was selective for picric acid over other nitro-aromatic compounds, such as 2,4-dinitrophenol, 4-nitrophenol, trinitrotoluene, 2,4-dinitrotoluene, 3,4-dinitrotoluene, 4-nitrotoluene, 4-nitrobenzoic acid, 1,3-dinitrobenzene, and nitrobenzene (Figure 51).^135^

**Figure 52:**
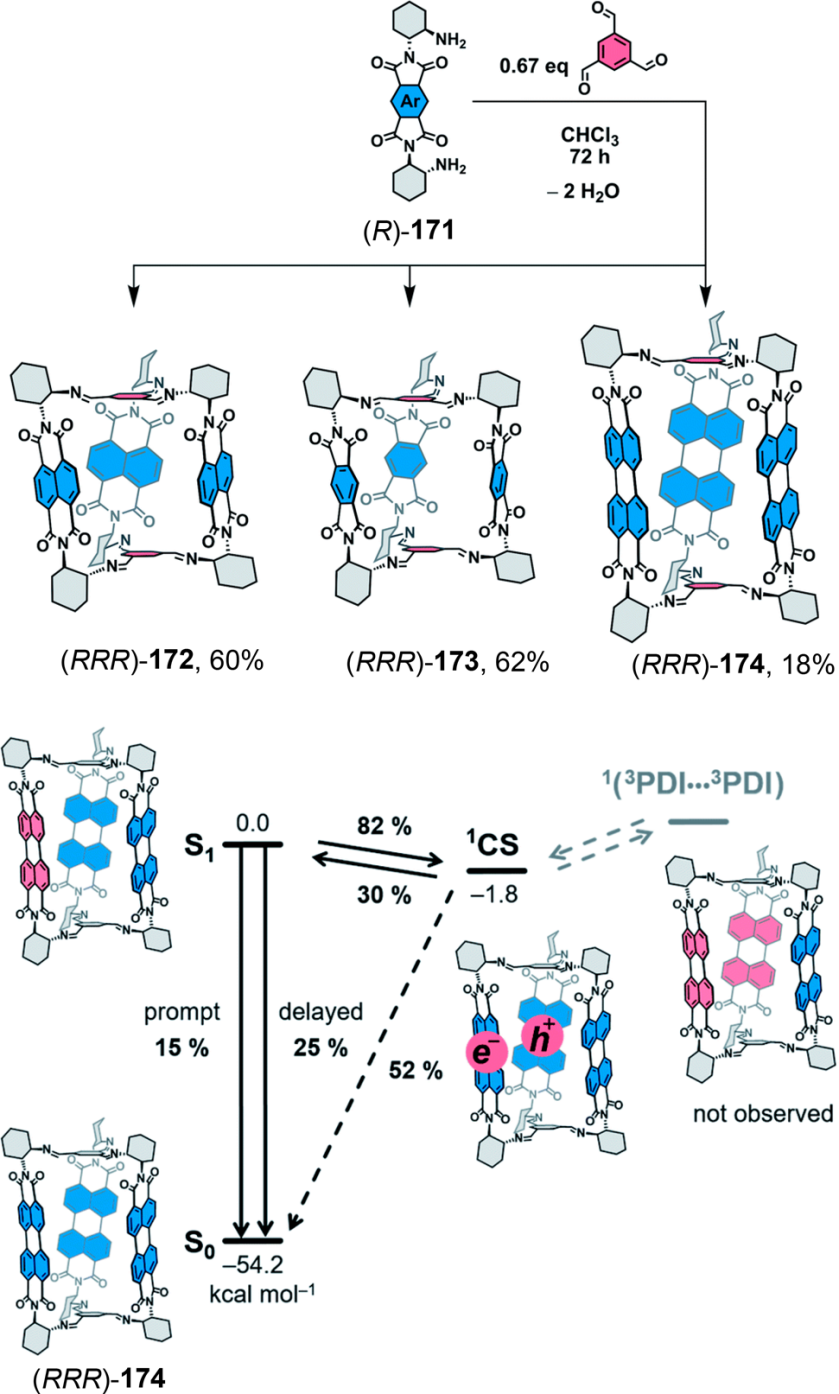
Synthesis of cages **172**, **173**, and **174** (top). Energy-level schematic (kcal/mol) for the photoprocesses in **174**. The quantum yields obtained for benzonitrile solutions are given (bottom). CS: charge-separated state. Dashed arrows: not observed.^136^ Adapted with permission from ref (136). Copyright 2021 Royal Society of Chemistry.

Several authors have obtained chiral cages using appropriate chiral building blocks. For instance, Šolomek, Coskun, and co-workers reported the synthesis of chiral [2 + 3] imine cages using three built-in rylene units by means of imine bonds from precursors **171**. The naphthalene-1,4:5,8-bis(dicarboximide) **172** (NDI) and pyromellitic diimide **173** (PMDI) cages showed good selectivity for CO_2_ adsorption over N_2_ and CH_4_, whereas the cage containing perylene-3,4:9,10-bis(dicarboximide) (PDI) could not be synthesized on a large scale to perform gas adsorption studies. In addition to these experiments, the authors found that cage **174** displayed efficient delayed fluorescence emission, which was attributed to the precise spatial arrangement of the PDI units in the cage structure. The spectroscopic and electrochemical data suggested that a rapid (pseudo)equilibrium was struck between the singlet excited state of **174** and the intracage charge-separated state. The repopulation of the bright singlet excited state by radical ions recombination produced the observed delayed fluorescence (Figure 52).^136^ This work demonstrates that rylene cages provide a precise spatial arrangement of PDI moieties, which is an important requirement for electronic materials by allowing electronic communication between them. The authors point out that these cage systems can be used in the future to develop new porous materials with unique photochemical and electronic properties.

**Figure 53:**
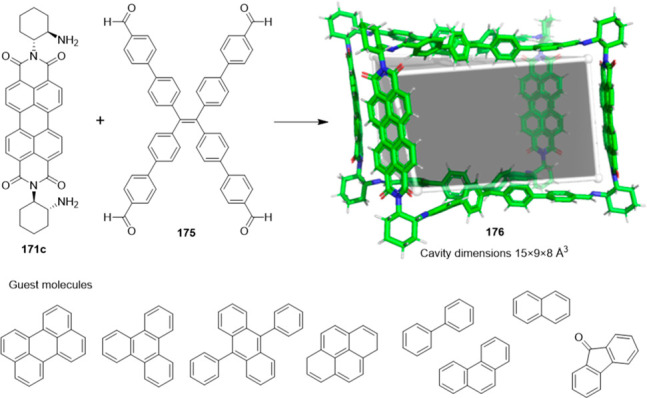
Synthesis of the PDI-TFBE **176** cage and the encapsulated guest structures.^137^

On the basis of the same amino chiral building blocks, but by changing trialdehyde to tetraaldehyde, Zhang, Ke, and co-workers prepared enantiopure [4 + 2] imine cages using perylene diimide **171c** and electron-rich TFBE building block **175** to yield cage **176** with a cavity size of ca. 1.5 × 0.9 × 0.8 nm^3^. Cage (*R*,*R*,*R*,*R*)-**176** encapsulated from planar polycyclic aromatic hydrocarbons (PAHs) to fullerene guest molecules (Figure 53). Molecular modeling indicated that PAHs molecules were near the cage’s PDI cores, and each PDI-TFBE cage could accommodate 10 guest perylene molecules. The visible-light-driven Smiles rearrangement of 2-aryloxybenzoic acids to aryl salicylates with good efficiency was achieved in the presence of the imine cage that was used as a catalyst. The authors suggested that the reaction took place by a single-electron transfer (SET) between the excited (*R*,*R*,*R*,*R*)-176* cage and the carboxylate group from the substrate (Figure 53).^137^

**Figure 54:**
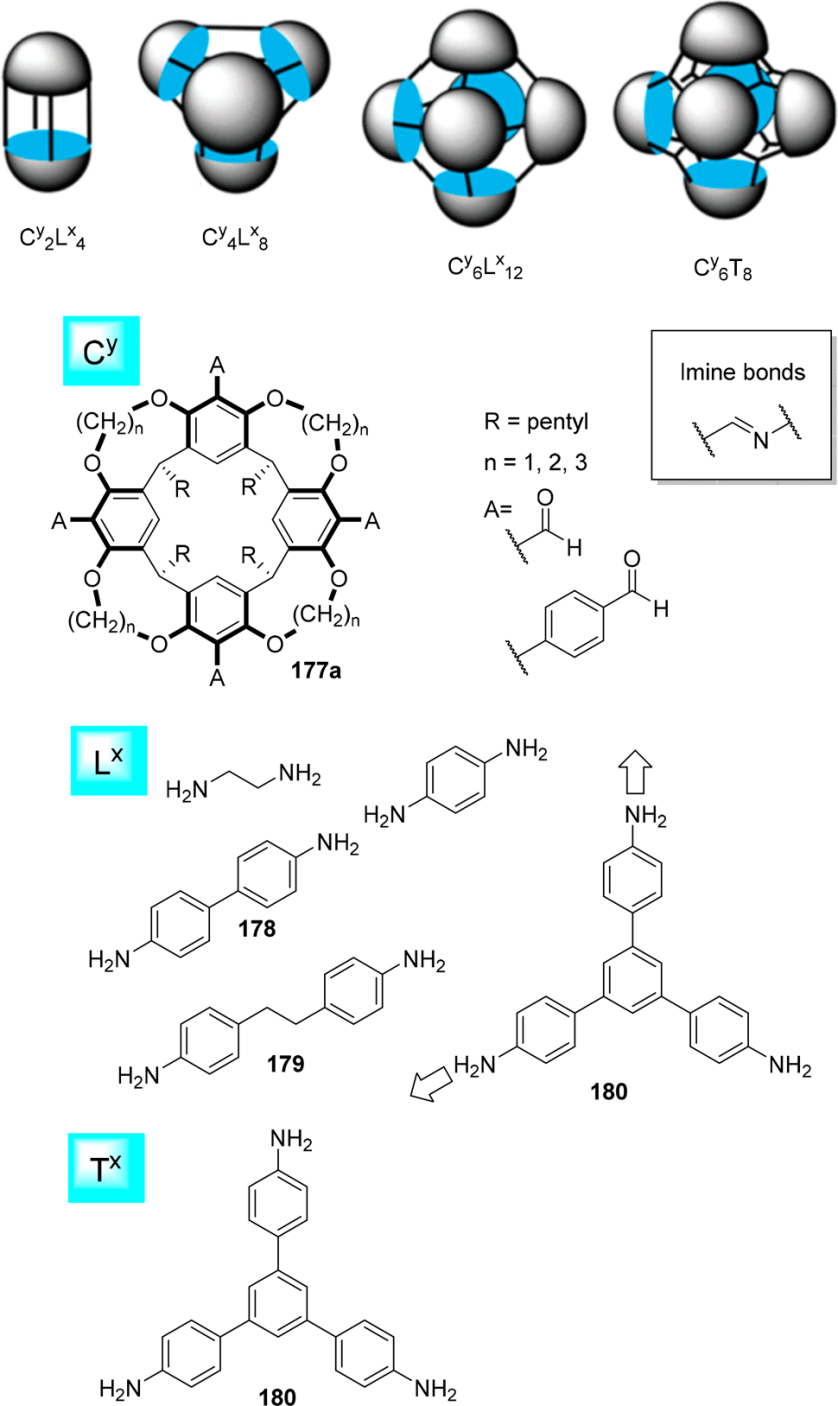
Cartoon representation of the C^y^_*n*_L^*x*^_*m*_ capsules and the structure of functionalized cavitands **177a**, amine building blocks, and the corresponding capsules.^138^ Adapted with permission from ref (138). Copyright 2011 Royal Society of Chemistry.

Warmuth and co-workers developed an approach using imine bond formation to prepare multicomponent nanometer-sized octahedrons. Octahedral capsules (C^y^_6_L^x^_12_ and C^y^_6_T^x^_8_) products were obtained from tetra-formyl resorcinarene cavitands **177** and amine derivatives (ethylenediamine, *p*-benzenediamine and compounds **178**–**180**). To obtain this cage structure, it was crucial that the cage building blocks had a high degree of conformational rigidity with the appropriate geometry to exclude other possible capsule products to maximize selectivity. Variation in diamine length for octahedrons or the size of the triamine for rhombicuboctahedrons allowed cavity size to be customized to obtain large cavity volumes ranging from 7400 Å^3^ to 13000 Å^3^. These volumes were on a scale of sizes of smaller globular proteins or other biomacromolecules, and the authors suggested that these cages could find biomedical, biochemical, or materials science applications (Figure 54).^138,139^ One advantage of these systems was their robust synthetic methodology, which allowed scaling up synthesis to a multigram scale. For example, the synthesis of the octahedral nanocontainer with an ethylenediamine spacer was obtained on a 5 g scale.^140^ In addition to the symmetric capsules described in Figure 54, the same research group prepared a chiral capsule using a cavitand with less symmetry. The obtained chiral capsule had a large enantiomerization free energy barrier of 21.5 kcal/mol.^141^

**Figure 55:**
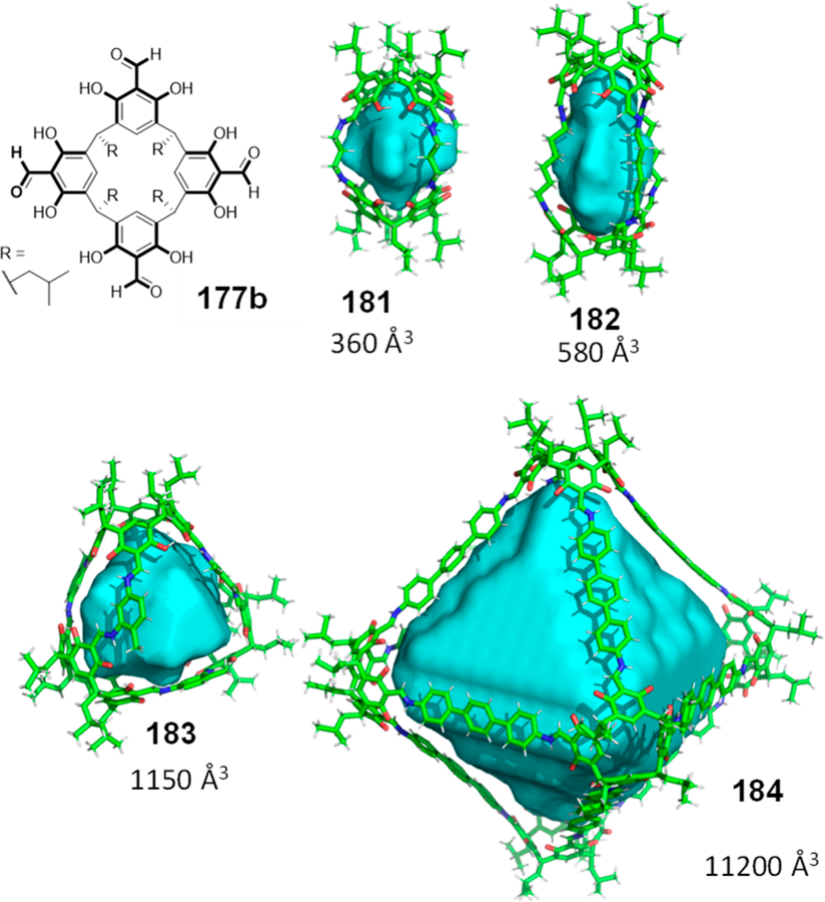
Imine cages **181**–**184** with cavity volumes ranging from 360 Å^3^ to 11200 Å^3^.^142^

By means of the resorcinarene core motif **177b**, Yuan and co-workers prepared different imine cage structures with cavity volumes ranging from 360 Å^3^ to 11 200 Å^3^. Synthesis involved tetraformylresorcin[4]arene cavitand **177b** with different diamine linkers (1,2-ethylenediamine, 1,5-pentanediamine, 4-methyl-1,3-phenylenediamine, 4,4′′-diaminoterphenyl, etc.). The authors prepared the [2 + 4] dimeric cages **181** and **182**, the [3 + 6] trimeric triangular prism cage **183**, and the [6 + 12] hexameric octahedral cage **184** (Figure 55), as well as several isostructural cages (not shown in Figure 55). The cavity of these systems was formed by π-rich aromatic rings with surrounding phenol groups along with imine groups. The gas adsorption measurements in the solid state suggested that the prepared cages were all porous materials with BET values above 1000 m^2^ g^–1^.^142^ These solids exhibited solvatomorphism, which resulted from changes in their crystallographic packing in the solid state. These changes in solid-state packing modified the materials’ gas sorption properties,^143^ which have been used to separate ethane from an ethane/ethylene mixture using the cage formed by cavitand **177b** and *p*-phenylenediamine.^144^

**Figure 56:**
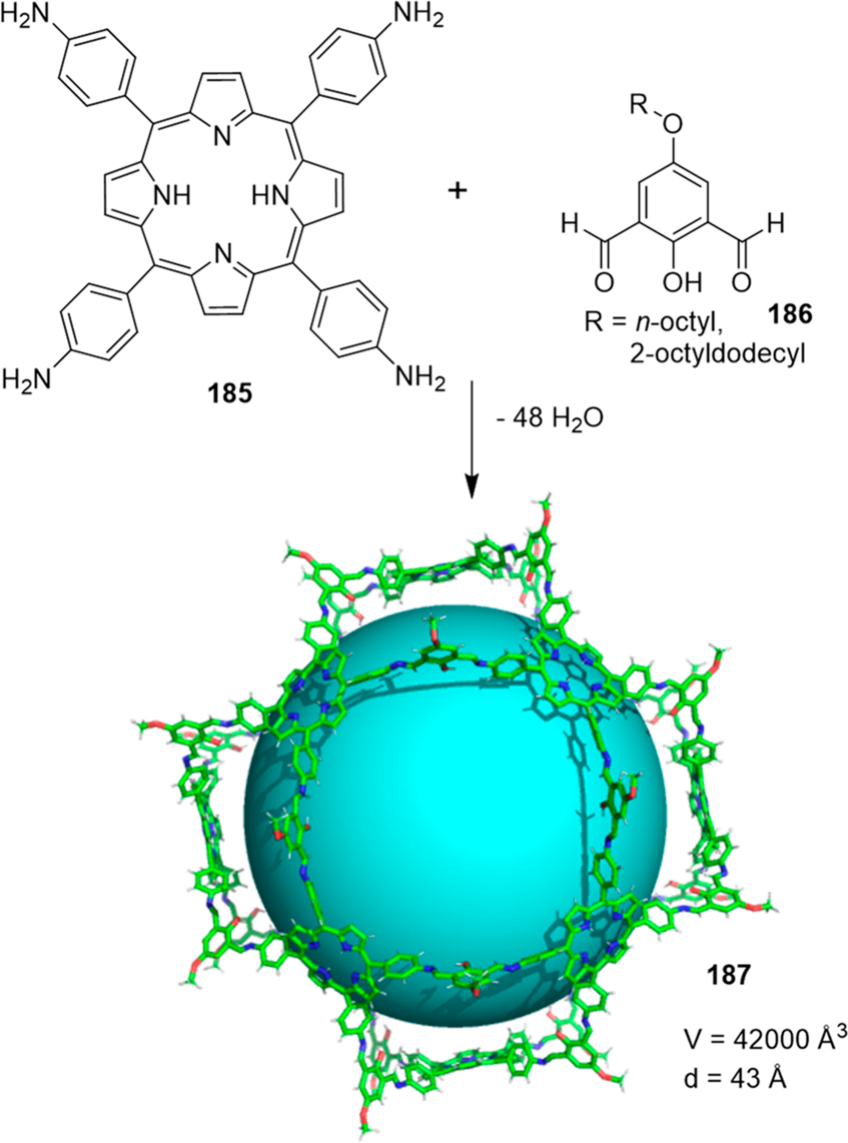
Structure of the gigantic porphyrinic cages P_12_L_24_ (**187**). R groups (*n*-octyl) omitted for clarity.^145^

Kim, Baik, and co-workers used tetramino-porphyrin **185** and dialdehyde building block **186** to prepare the gigantic porphyrinic organic cage structure **187**. This cage has a P_12_L_24_ topology cavity with a diameter of 4.3 nm. The authors managed to synthesize the cage after numerous attempts, and the main challenges were the solubility of systems, and the formation of both polymeric byproducts and small-sized cages that were entropically favored. The X-ray crystal structure showed that **187** has the largest cavity in a pure organic cage reported to date (Figure 56).^145^

**Figure 57:**
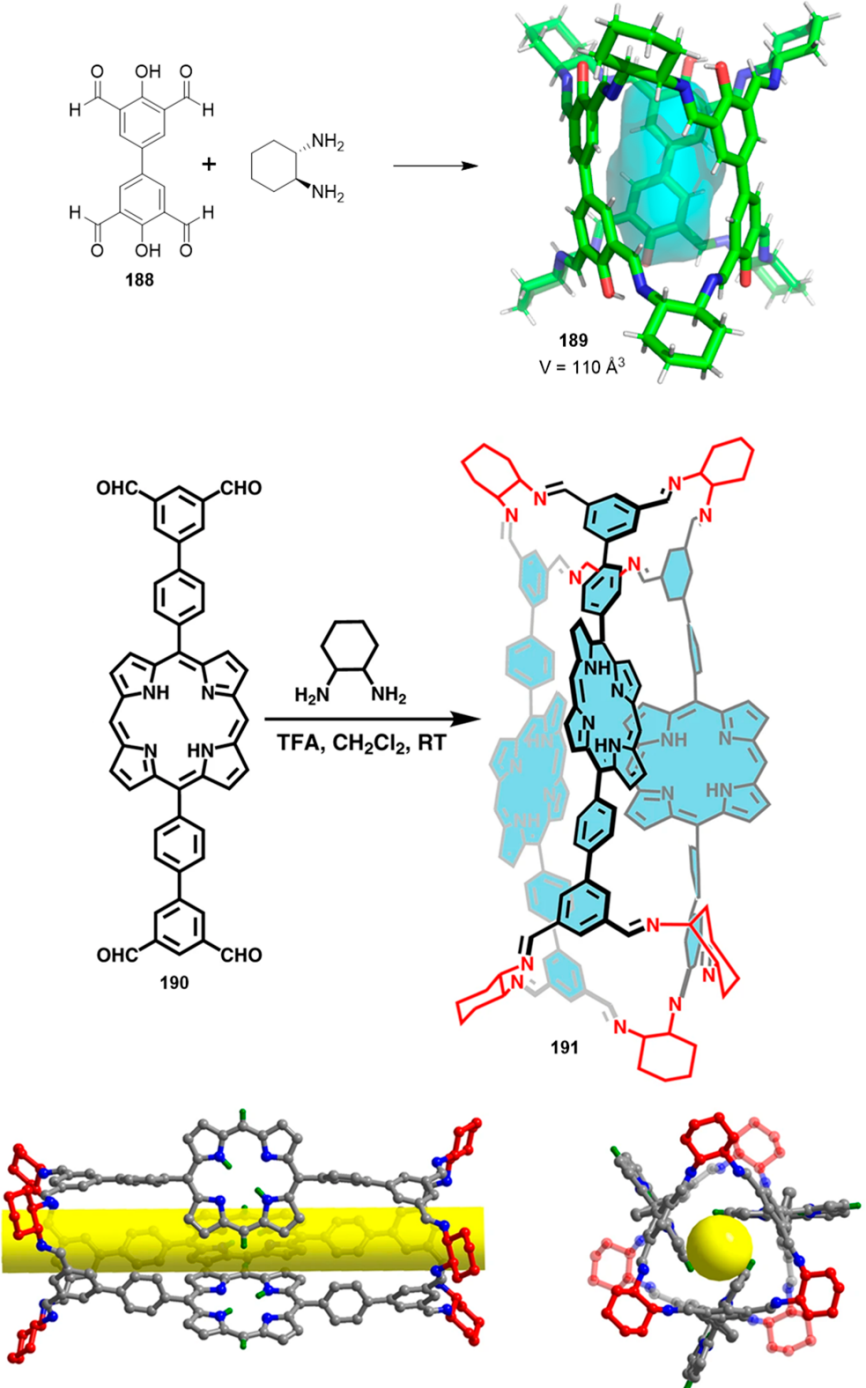
Synthesis of [3 + 6] imine cages **189** and **191** (cylindrical cavity of cage **191** represented in yellow).^146,147^ Reproduced with permission from ref (147) with the Creative Commons CC BY license http://creativecommons.org/licenses/by/4.0/. Copyright 2020, the authors of the original publication.

Pan, Huang, and co-workers described the [3 + 6] condensation of tetraaldehyde **188** with 1,2-cyclohexanediamine to form imine cage **189**. Crystallization of the cage in the presence of excess NaN_3_ produced the coordination of Na^+^ to the cage and resulted in a hierarchically porous structure with interconnected channels and excellent CO_2_ storage capacity (Figure 57 top).^146^ Jiang, Chen, Wang, and co-workers adopted a similar synthetic strategy with tetraaldehyde **190** and 1,2-cyclohexanediamine to prepare porphyrin cage **191** with good efficiency. This cage has been used as a high-efficacy singlet oxygen generation system (Figure 57 bottom).^147^

**Figure 58:**
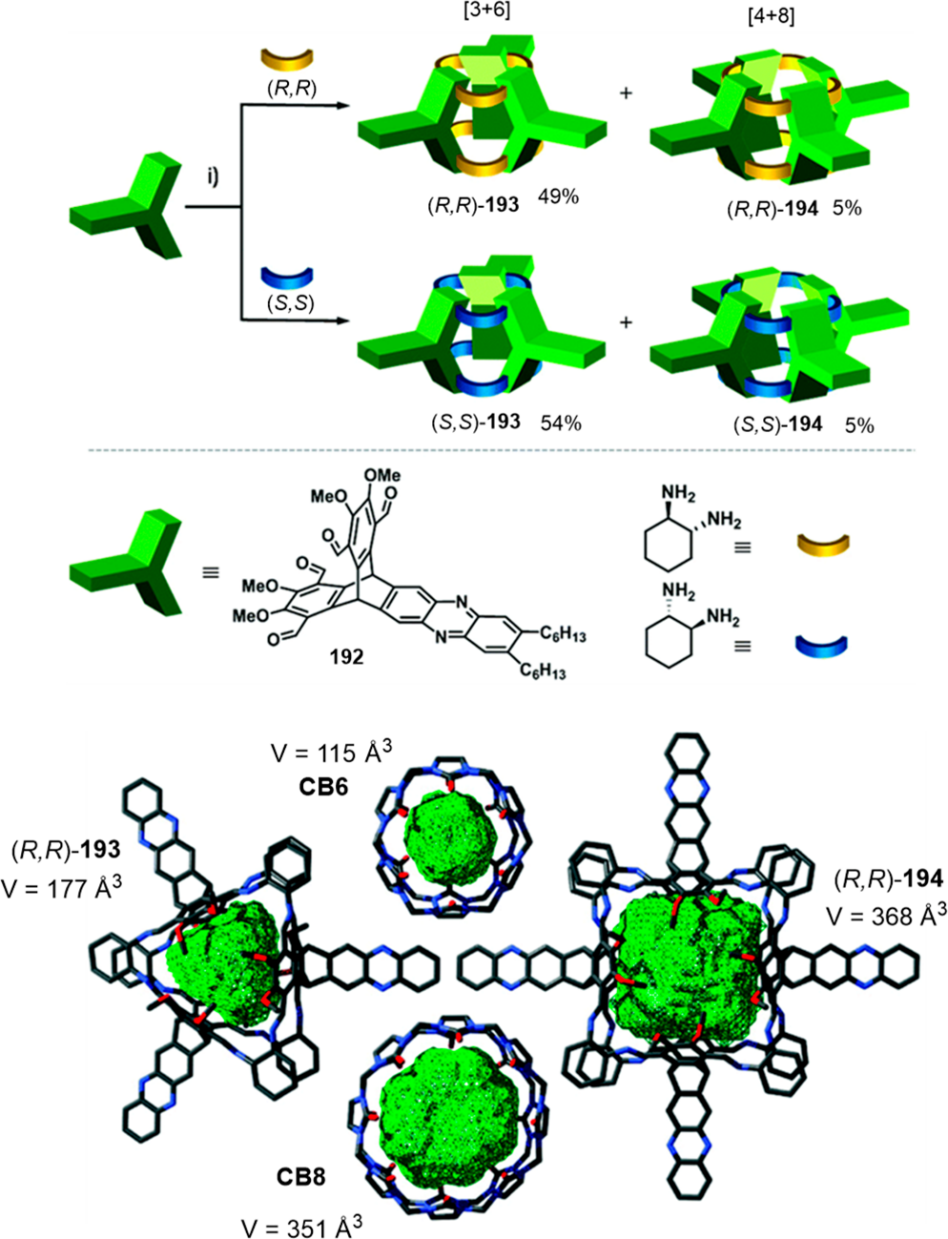
Synthesis of cucurbitimines **193** and **194**. (i) DCM, 2 mol % TFA, rt, 7 days. Comparison of the molecular pore volumes of (*R*,*R*) cucurbitimines [3 + 6] and [4 + 8] with cucurbiturils CB6 and CB8.^148^ Adapted with permission from ref (148). Copyright 2021 the authors of the original publication. Published by the Royal Society of Chemistry with the Creative Commons CC BY license http://creativecommons.org/licenses/by/4.0/.

Mastalerz and co-workers applied a [3 + 6] imine condensation of triptycene tetraaldehydes **192** with 1,2-cyclohexyl-diamines to prepare pumpkin-shaped cages **193** and **194** with concave walls, named cucurbitimines, for resembling cucurbiturils. Besides the [3 + 6] **193** cage, which was obtained with a ca. 50% yield, the [4 + 8] cage **194** was also formed with only a 5% yield for both enantiopure (*R,R*)- and (*S,S*)-1,2-cyclohexyldiamines. The [3 + 6] cage **193** encapsulated neutral guests, such as CH_2_Cl_2_, THF, or toluene. Guest exchange was also studied, and the authors found that THF was exchanged by toluene on more than 2 days at room temperature or in less than 1 h at 100 °C (Figure 58).^148^

**Figure 59:**
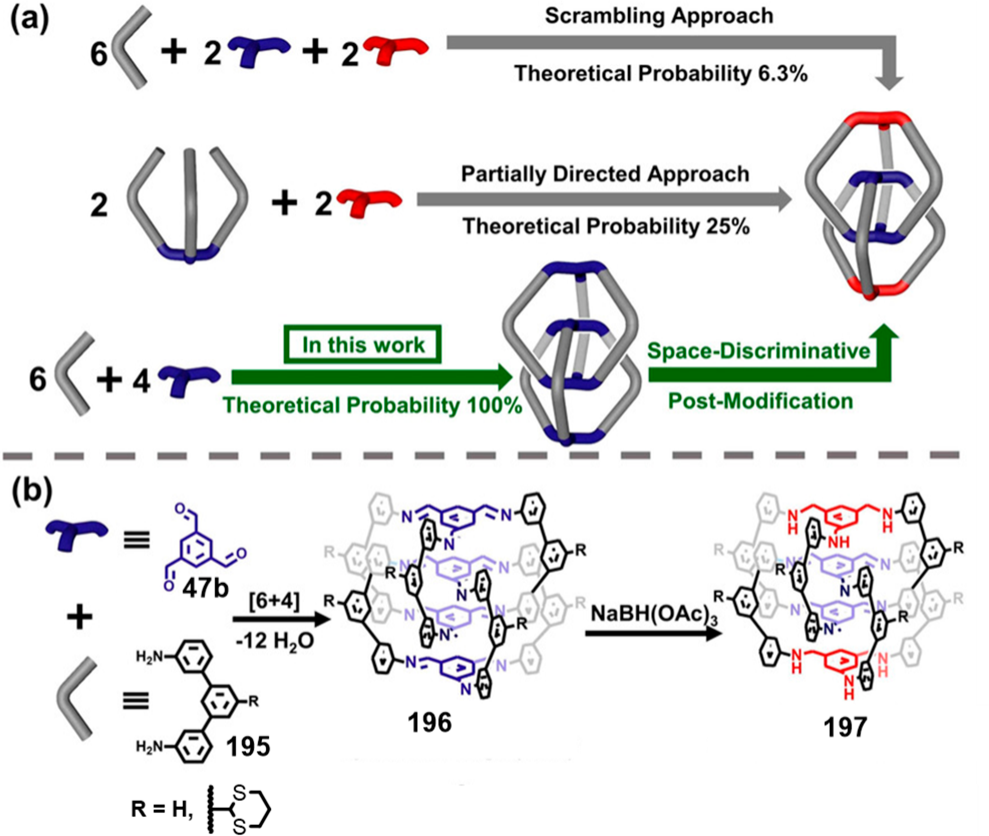
(a) Three methodologies to prepare dimeric catenane dissymmetric cages **197**. (b) The TFA-catalyzed [6 + 4] imine condensation yielded the catenane structure. The exterior imine groups were selectively reduced using NaBH(OAc)_3_.^149^ Reproduced with permission from ref (149). Copyright 2020 Wiley-VCH.

Zhang, Liu X., Liu Z., and co-workers employed precursors **47b** and **195** to prepare catenanes **196** and **197**. Catenane **198** is formed by two dissymmetric cages by means of a space-discriminative postassembly modification strategy using a voluminous reductant like NaBH(OAc)_3_ to selectively reduce the outer imine bonds. The authors calculated that the two possible direct syntheses offered a very low theoretical probability of cage formation after considering all the possible cages that could be obtained by the random assembly of building blocks. In particular, the theoretical probabilities of cage formation were 6.5% for the scrambling approach and 25% for the partially directed approach. In contrast, the postmodification approach was much more efficient given its 100% theoretical probability (Figure 59a). This method is based on the different environments around the imine bonds within catenane framework **196.** The imine bonds in the inner cavity are protected by steric congestion, and the outer imine bonds are exposed to the solvent and are, therefore, accessible to react with voluminous reductant NaBH(OAc)_3_. In contrast, smaller reductant NaBH_4_ produces nonselective reduction by reducing both the inner and outer imine bonds. The authors point out that this synthetic strategy can be used to develop catenated cages with sophisticated topologies to obtain materials with advanced functionalities (Figure 59).^149^

**Figure 60:**
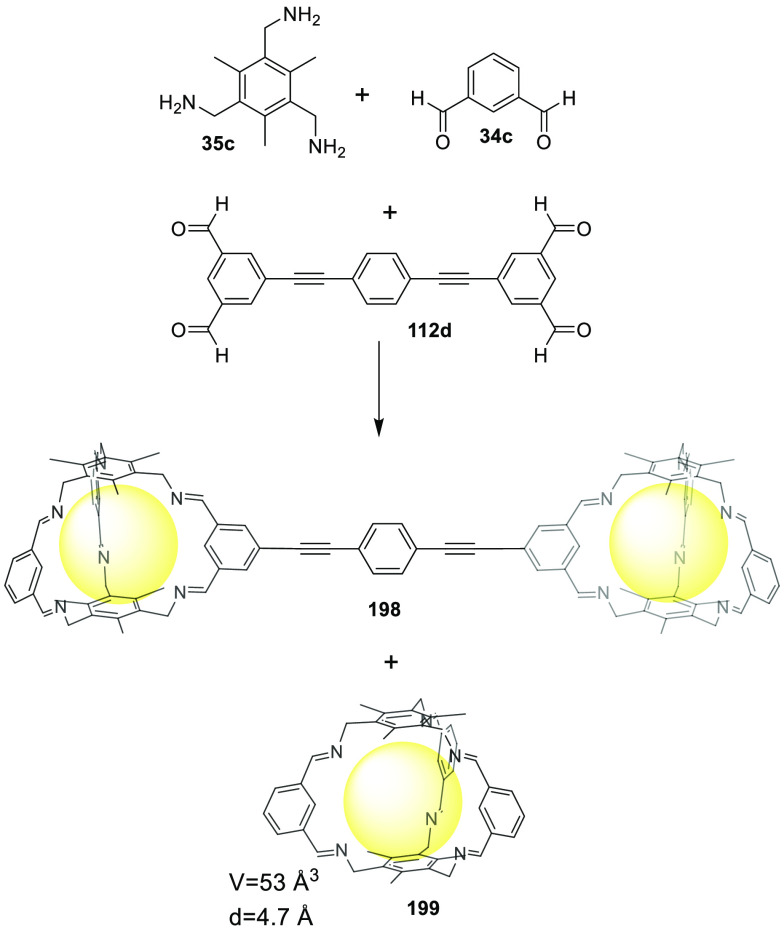
Social sorting of a tritopic amine (**35c**), ditopic aldehyde (**34c**), and tetra-topic aldehyde (**112d**) to form a mixture of cage **199** and dumbbell cage **198**.^150^

Greenway and co-workers performed the social-sorting of mixtures containing tritopic amine **35c**, ditopic aldehyde **34c**, and tetra-topic aldehyde **112d**, which resulted in a mixture of dumbbell cage **198** and cage **199** (Figure 60).^150^ This work provides a proof-of-concept for preparing complex systems containing many covalently bonded cages.

**Figure 61:**
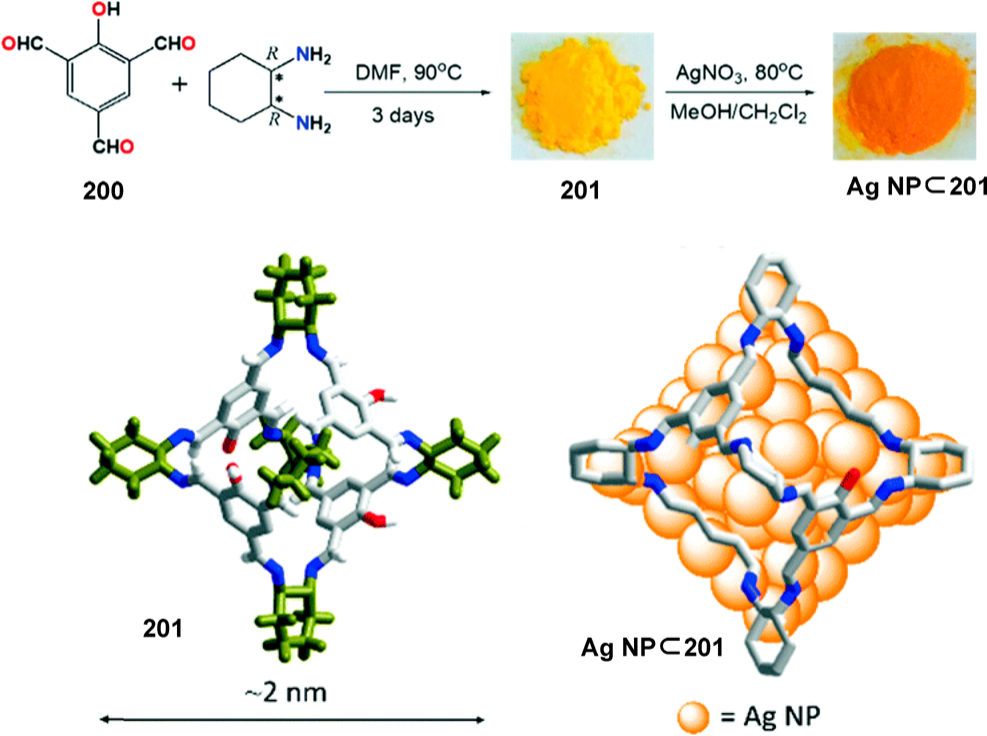
Synthesis of organic molecular cage **201** loaded with an Ag nanoparticle.^152^ Adapted with permission from ref (152). Copyright 2019 Royal Society of Chemistry.

Several authors have used cages to anchor nanoparticles in the internal cage and to obtain nanoparticles with a controlled narrow size distribution.^151^ For instance, Dong, Chen, and co-workers employed 2-hydroxy-1,3,5-triformylbenzene **200** and 1,2-cyclohexanediamine to prepare cage **201**, which was loaded with Ag nanoparticles. This cage has the core structure of cage **CC3**(89) but changed the 1,3,5-triformylbenzene building block used to prepare cage **CC3** by 2-hydroxy-1,3,5-triformylbenzene **200**. The authors found that the N and O atoms in the cage templated the formation of the Ag nanoparticles in the cavity. The formation of nanoparticles in the cage was studied by HRTEM, which showed comparable homogeneous nanoparticle distribution to the cage shell shape. The formation of Ag nanoparticles in the cage cavity was also supported by loss of porosity from the gas adsorption–desorption experiments (Figure 61).^152^ Beaumont, Jiang, and co-workers prepared palladium nanoparticles using cage **CC3**. They demonstrated that the initial Pd(NO_3_)_2_ encapsulation in the cage cavity was a key factor for the formation of the ultrasmall nanoparticles obtained by the reduction with H_2_, with no chemical damage to the cage structure. In contrast, if the larger sized Pd(acac)_2_ metal precursor was used, no control in nanoparticle size was observed, and this was attributed to the impossibility of the precursor fitting inside the cage cavity.^153^ The selective binding of palladium ions in an imine cage with a tubular structure has also been used for the encapsulation of Pd nanoparticles inside the cavity of a cage obtained by the condensation of a benzo[*c*][1,2,5]thiadiazole tetraaldehyde derivative and 1,2-cyclohexanediamine.^154^

**Figure 62:**
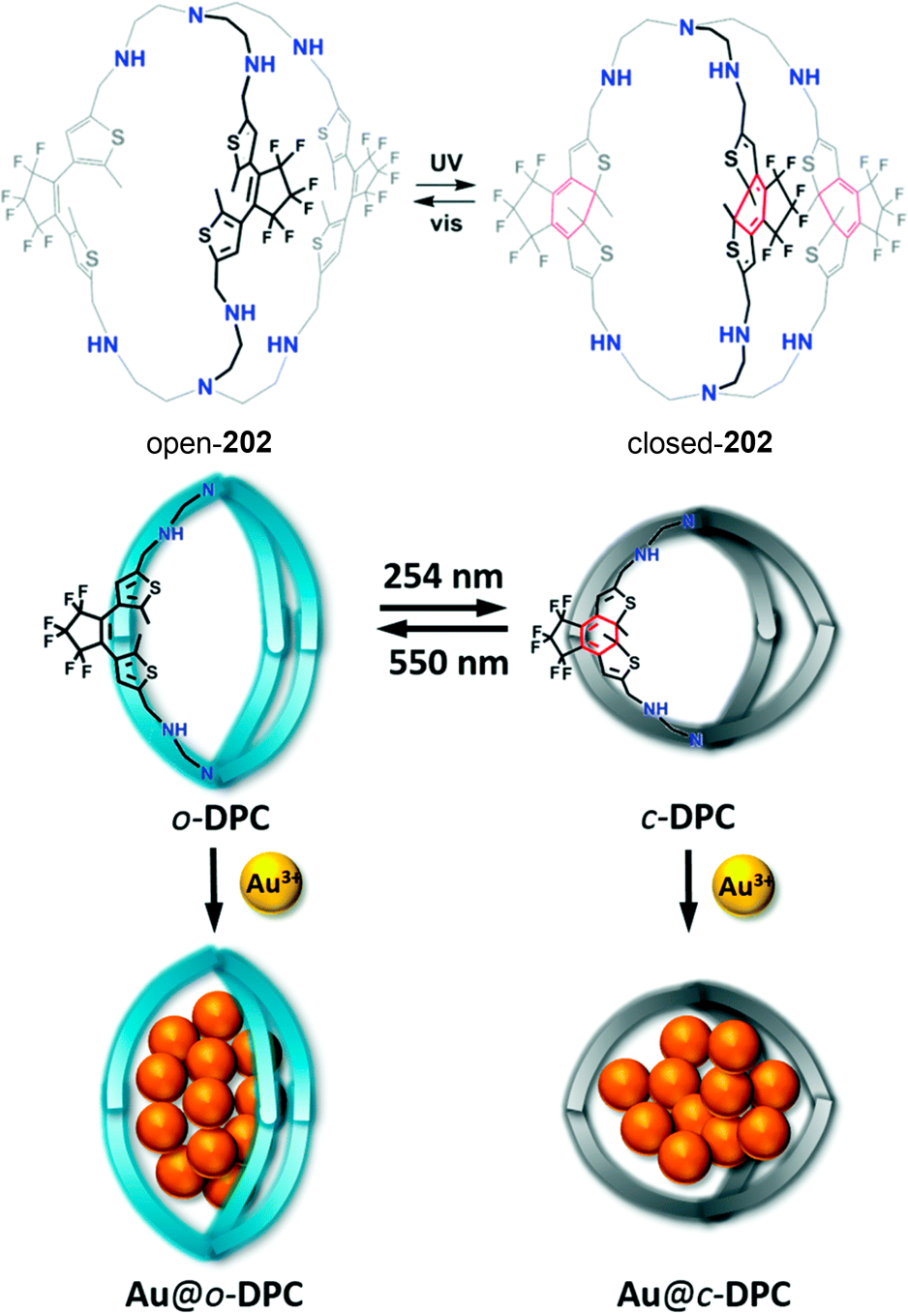
Conversion between the open and closed forms of diarylethene cage **202** displaying adjustable cavity size and the encapsulated Au nanoparticles.^155^ Adapted with permission from ref (155). Copyright 2020 Royal Society of Chemistry.

Gu and co-workers prepared diarylethene photochromic cage **202** (with an adjustable cavity size by irradiation) by the condensation of tren with a diarylethene-dialdehyde, followed by imine reduction. Cavity reduction took place with 254 nm UV light to yield the cage closed form, and cavity enlargement occurred with 550 nm visible light to yield the cage open form as described in Figure 62. The diameters of the cavity sizes of the open and closed forms were 1.17 and 0.91 nm, respectively. In addition, the authors found that the cage could coordinate Au(III), and after reduction with NaBH_4_, the Au NPs inside the cage cavity formed. The different microenvironments of the open/closed cages containing Au NPs had a direct effect on the catalytic activity of Au on the reduction of 4-nitrophenol with NaBH_4_. The observed apparent reaction kinetic constants were 4.1 × 10^–2^ s^–1^ for the open cage containing Au NPs and 1.7 × 10^–2^ s^–1^ for the closed cage containing Au NPs. This reduction in the kinetic constant is related to the structure of cages, which modifies the accessibility of reactants to the nanoparticle. The closed form of the cage has a flatter structure, which makes it more difficult for the 4-nitrophenol to reach Au NPs and results in a lower the reaction rate. Conversely in the open form, the cage diameter is larger and 4-nitrophenol can easily reach Au NPs (Figure 62).^155^

**Figure 63:**
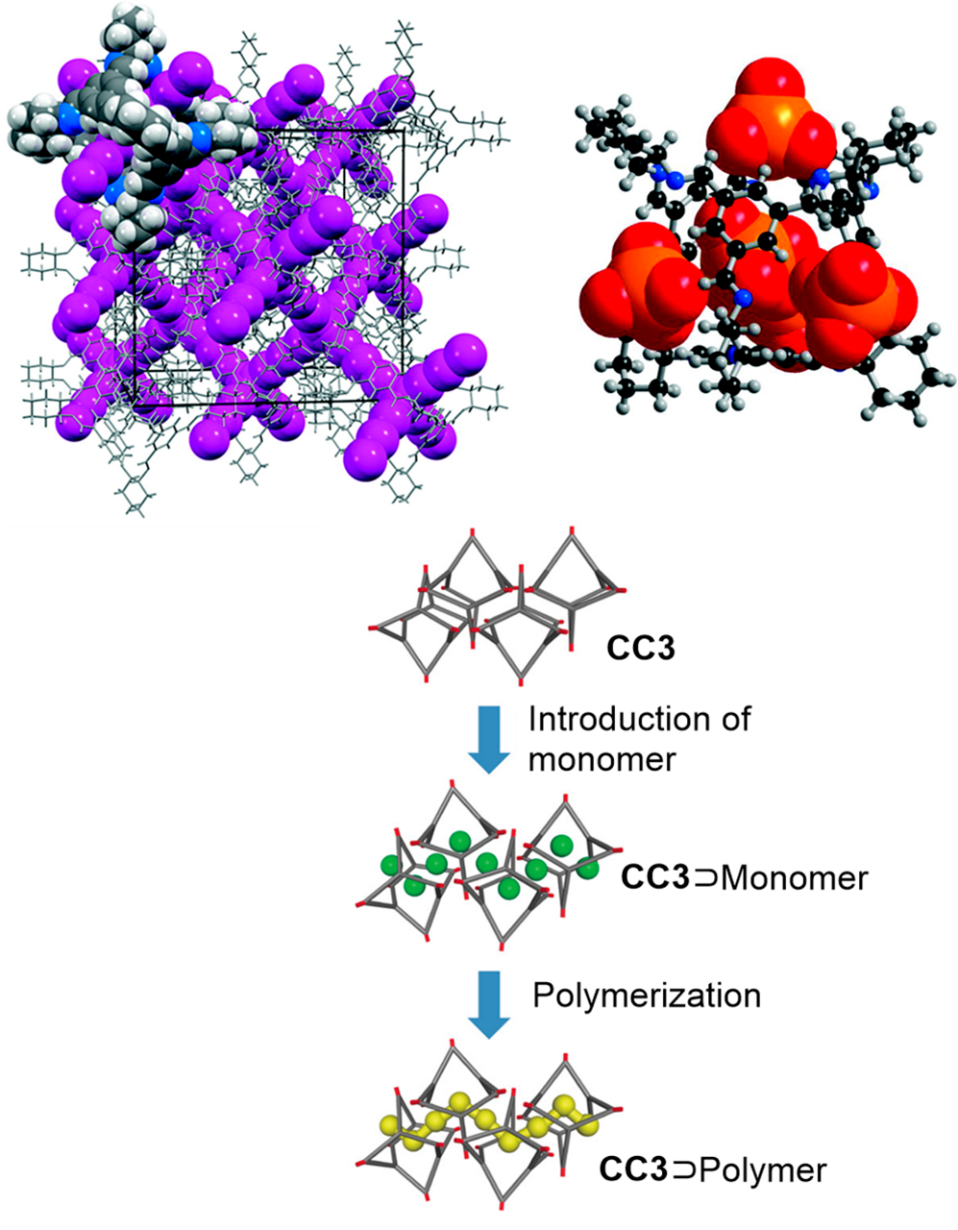
(top) Packing of cage **CC3** illustrating the 3-D diamondoid pore network filled with iodine or OsO_4_.^156^ (bottom) Radical polymerization of vinyl monomers.^157^ Adapted with permission from ref (156). Copyright 2011 American Chemical Society. Adapted with permission from ref (157). Copyright 2016 Wiley-VCH.

Cooper and co-workers loaded halogen and organometallic guest molecules into the porous structure of a **CC3** crystalline organic cage. To do so, the desolvated crystals of **CC3** were placed in a sealed vessel in the presence of solid iodine. Sublimed iodine at room temperature condensates into the pores of the **CC3** crystals, producing a change of color from white to almost black. Iodine atoms were organized in a diamondoid arrangement that formed I_5_^–^ in the channels of the **CC3** structure. Cage **CC3** was also able to load OsO_4_ as a guest. Moreover, the authors envisaged the potential delivery of the encapsulated guests upon cage dissolution in common solvents (Figure 63 top).^156^ Similarly, Uemura and co-workers developed a method to perform radical polymerization of vinyl monomers in the porous structure of organic cage **CC3**. For this purpose, the authors adsorbed styrene monomer units in the pores of solid **CC3**, which resulted in a suitable monomer arrangement for polymerization. The flexibility of the organic cage packing structure allows polymerization to take place in the pores of the structure, unlike the rigid porous hosts, such as zeolites and MOFs, where polymerization does not take place (Figure 63 bottom).^157^

**Figure 64:**
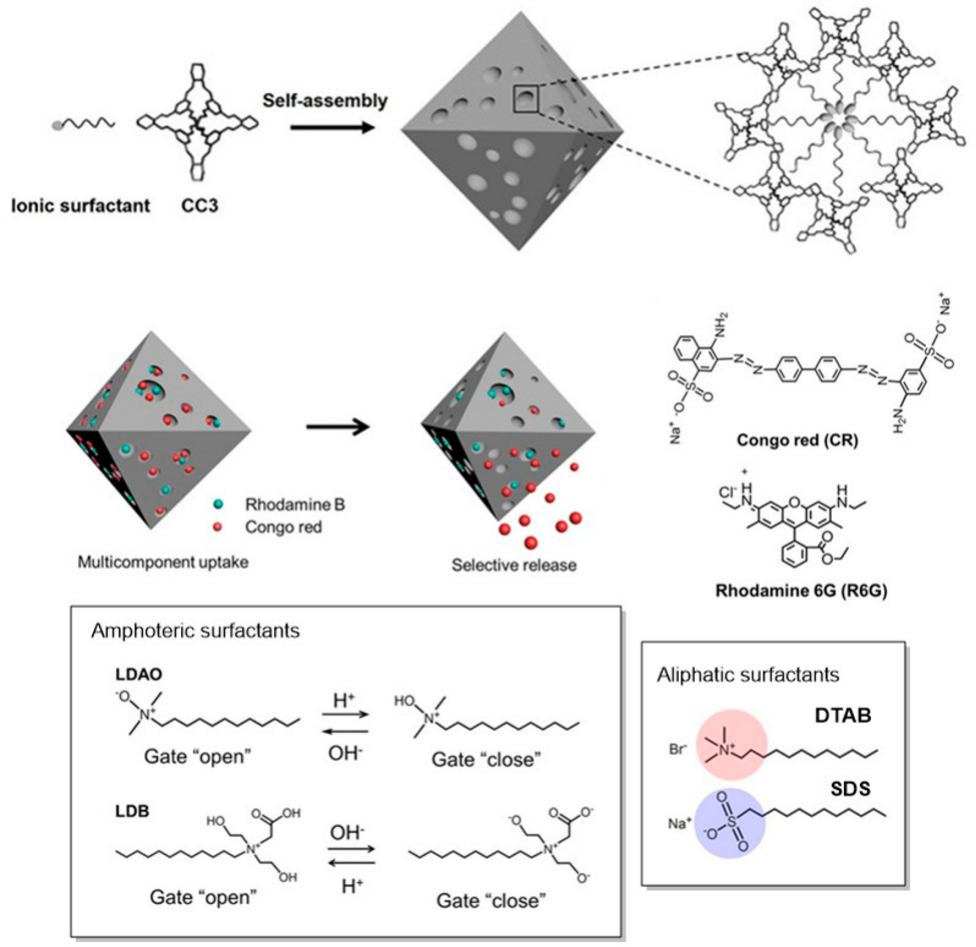
Hierarchically assembled porous colloids based on cages **CC3**.^158^ Adapted with permission from ref (158). Copyright 2021 Wiley-VCH.

Sun, Yang, Wei, and co-workers hierarchically assembled porous colloids based on **CC3** cages and surfactants with a positive or negative charge (Figure 64). The resulting materials have porous networks that can encapsulate guest molecules such as fluorescent dyes. In these materials, when the cationic rhodamine 6G and anionic Congo Red dye molecules are encapsulated in the **CC3**–surfactant porous colloid, charge selective uptake/release behavior is observed, which suggests that the electrostatic interaction between the surfactants and dyes play a key role. The authors encapsulated cationic rhodamine in the negatively charged **CC3**-SDS colloid and anionic Congo Red in the positively charged **CC3**-DTAB colloid. When using amphoteric surfactants, the polar head of the surfactants acts as a pH responsive electrostatic gate for the selective release of encapsulated guests. The authors used amphoteric surfactants lauryl dimethyl amine oxide (LDAO) and lauryl dihydroxyethyl betaine (LDB), which allowed the opening of the gate at a basic and an acidic pH, respectively. The properties of these materials open up new avenues to develop electrostatic gates based on surfactants by incorporating additional properties, such as pH-switchable behavior. The authors also used these systems to encapsulate an enzyme in the **CC3**–surfactant porous colloid to display greater catalytic activity compared to the free enzyme.^158^

**Figure 65:**
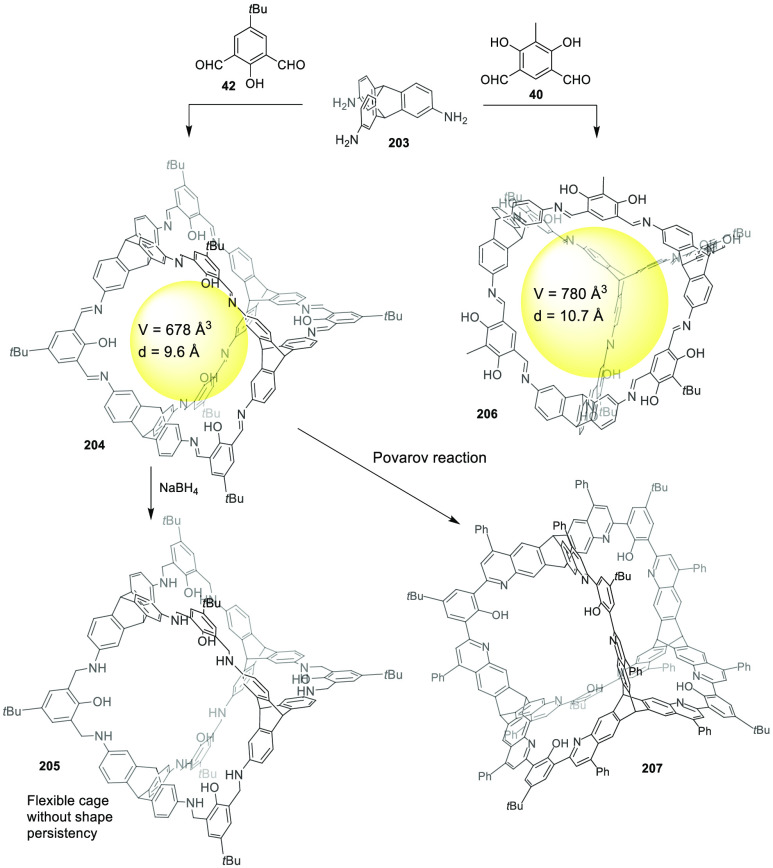
Synthesis of cages **204** and **205** by imine condensation. Conversion of imine cage **204** into amine cage **205** by imine reduction, and conversion of imine cage **204** into quinoline cage **207** by the Povarov reaction: Sc(OTf)_3_, chloranil, phenylacetylene (neat), 100 °C, 20 h, 25% yield.^160−163^

Postsynthetic modification of organic cages is a practical strategy for increasing their functions and applications because it allows the synthesis of structures that would otherwise not be possible.^159^ Mastalerz used dialdehyde **42** and triamine **203** to prepare the first shape-persistent adamantoid nanocage **204** with a cavity volume of 678 Å^3^. The cage was synthesized by reversible imine condensation in an effective and simple one-pot synthesis.^160^ To increase cage robustness, the simplest postsynthetic transformation of imine cages is to reduce imines to amines. However, reduction induces the transformation of sp^2^ into sp^3^-hybridized atoms, which results in a more flexible cage that may lose its shape persistency and, thus, its porosity. Hence the reduction of **204** to give the reduced **205** led to structure collapse in the solid state that did not allow gas penetration. Cage **205** displays a BET surface area of less than 1 m^2^/g, unlike parent imine cage **204** with a BET surface area of 1377 m^2^/g in the solid state.^161^ Besides, cage **206** (obtained from **40** and **203**) has a BET specific surface area of 1037 m^2^/g (N_2_, 77 K).^162^ To maintain the rigidity of imine cage **204** while increasing chemical robustness, Mastalerz and co-workers reported the conversion of **204** into quinoline cage **207** through a Povarov reaction. In this case, the obtained material had a BET specific surface area of 698 m^2^/g. Cage **207** was stable to a wide range of pH values, including very acidic conditions (concentrated sulfuric acid, pH −1.9) and very basic conditions (concentrated NaOH, pH 15.2) (Figure 65).^163^

**Figure 66:**
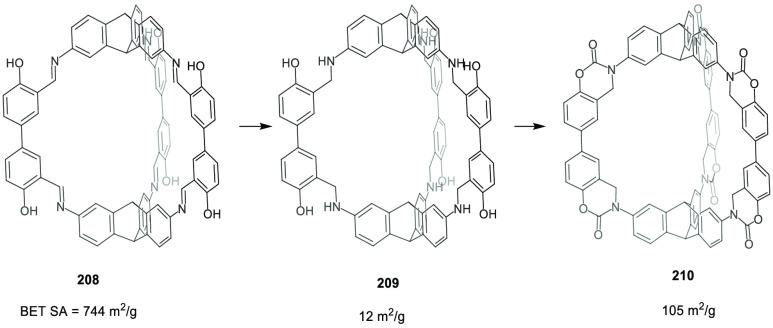
Synthesis of robust carbamate cage **210** that remained stable under acidic and basic conditions.^164^

Mastalerz and co-workers used imine transformation in cage **208** to carbamate (cage **210**) through intermediate cage **209** to increase stability while maintaining cage structure and rigidity. Cage structure rigidity after the corresponding reaction was confirmed by the BET surface areas (N_2_, 77 K) of 744, 12, and 105 m^2^/g for cages **208**, **209**, and **210**, respectively. Thus, whereas amine cage **209** displayed a small BET surface area as expected for its increased flexibility, carbamate cage **210** and especially imine cage **208** displayed a much bigger BET surface area (Figure 66). Cage **210** was stable under very acidic conditions and porosity remained after treatment with 10 M HCl for 16 h at room temperature. The cage was also stable in concentrated hydrochloric acid (pH = −1) at room temperature, 1 M hydrochloric acid (pH = 0) at 100 °C, or 1 M aqueous NaOH (pH = 14) (Figure 66).^164^

**Figure 67:**
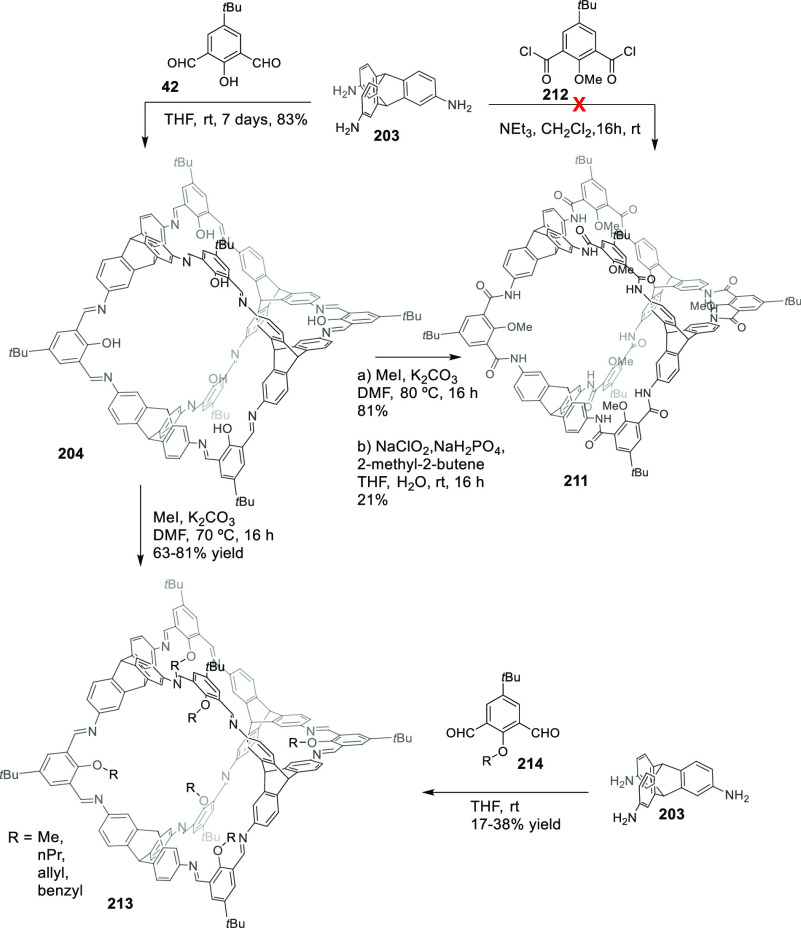
Synthesis of carbamate cage **211** from **204**(165) and postmodification of **204** by the reaction with MeI.^166^

In general, amide-based cages also have good chemical stability, which is, therefore, a suitable bond to obtain robust organic cages. However, direct synthesis through amide bond formation is an unsuitable procedure to obtain cages as shown in earlier sections of this review, where the cage assembly limitations through irreversible bonds were analyzed. To overcome these synthetic limitations, Mastalerz and co-workers used imine transformation to amide by the Pinnick reaction. This reaction was run to transform imine cage **204** into the corresponding amide cage **211**, which was impossible to obtain by the direct reaction of **203** and **212** (Figure 67). Amide cages **211** are porous in the solid-state with a BET surface area of 275 m^2^/g (N_2_, 77 K). The obtained amide cage is chemically robust at acidic (pH 1) and basic media (pH 14.5), which are one of the reported cages with the widest pH range stability. Only concentrated H_2_SO_4_ (36 M) produced cage decomposition. This exceptional robustness allowed the postfunctionalization of cage **211** with reactions like bromination and nitration, which require severe conditions. The resulting cages have electron-withdrawing substituents Br and NO_2_ that modify the polarity of amide bonds.^165^ Cage **204** was reacted with MeI to yield cage **213**, which can also be obtained by the reaction of **203** and **214** (Figure 67).^166^

**Figure 68:**
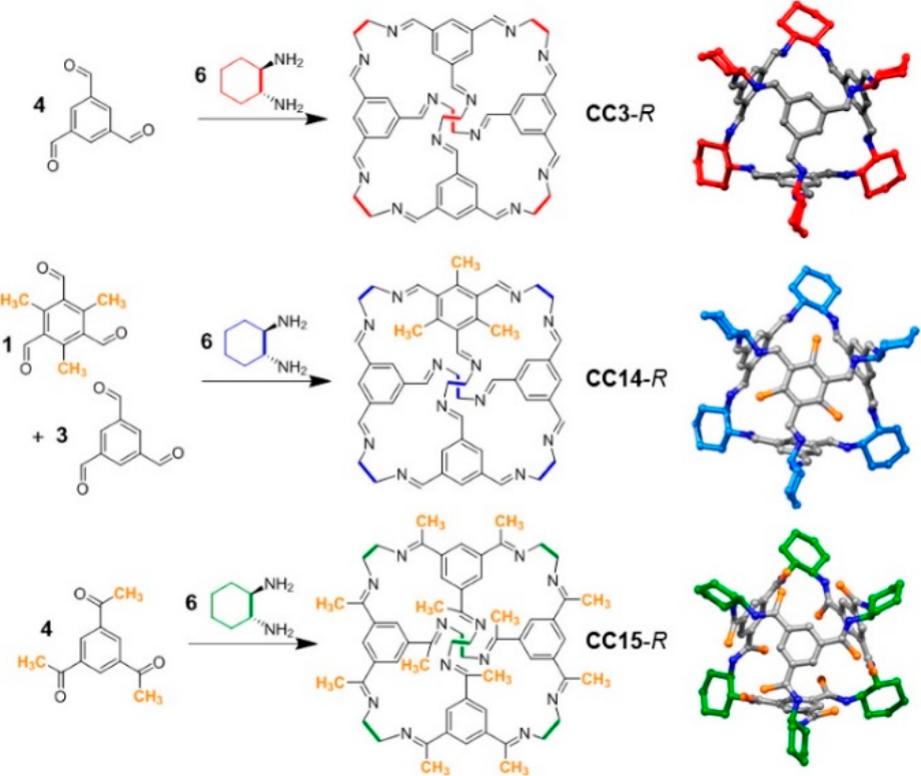
Synthesis of the parent **CC3**-*R* cage and the methylated analogues **CC14**-*R* and **CC15**-*R*.^167^ Reproduced with permission from ref (167). Copyright 2017 American Chemical Society. Further permissions related to this figure should be directed to the American Chemical Society.

Cooper, Day, and co-workers used computational crystal structure calculations to predict the packing preferences of homochiral methylated cages **CC14**-*R* and **CC15**-*R* (Figure 68). This methodology allowed cage cavity size and shape, along with pore connectivity, to be calculated, which are both key parameters to predict the gas sorption properties of porous materials based on molecular cages.^167^ The authors foresee that future design strategies based on the developed methodology will enable access to a bigger family of cages with specific physical properties for given applications.

**Figure 69:**
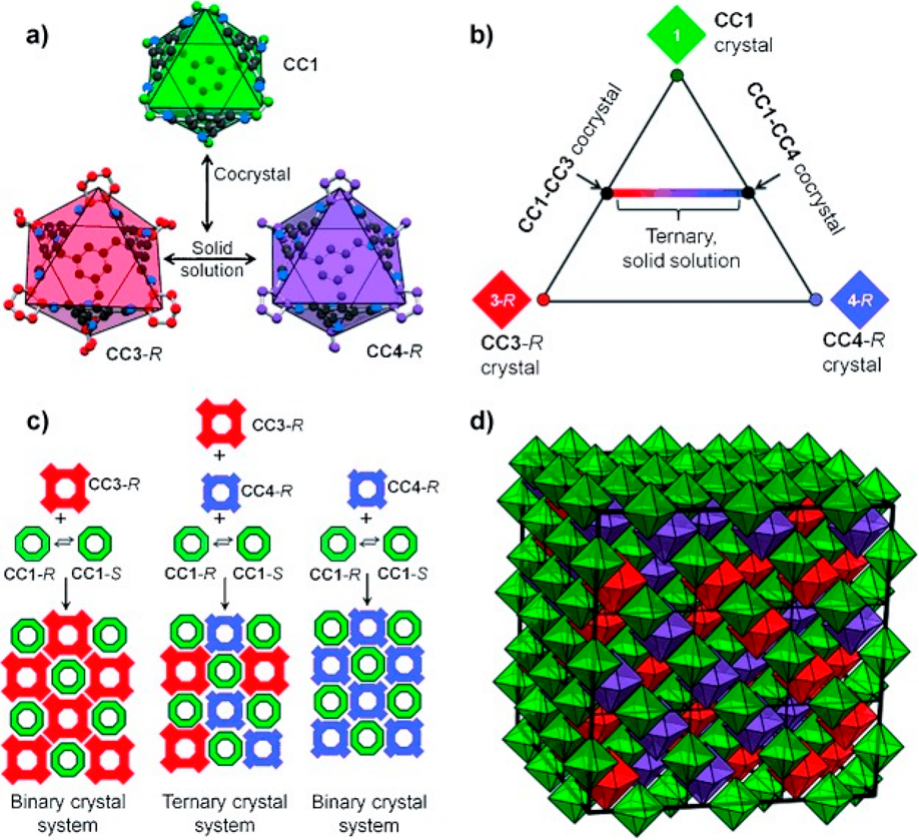
(a) Structures of cages **CC1**, **CC3**-*R*, and **CC4**-*R*, which form binary and ternary cocrystals, (b) where *R* enantiomers comprise a solid solution. (c) The chirality of **CC1** is resolved by the cocrystallization with **CC3**-*R*, **CC4**-*R*, or a mixture of both modules. (d) Cubic packing in the porous crystal. The CC1 modules (green) occupy half the lattice sites; **CC3**-*R* (red) and **CC4**-*R* (purple) are disordered over the remaining sites.^169^ Reproduced with permission from ref (169). Copyright 2012 Wiley-VCH.

With **CC***n* tetrahedral imine organic cages (see the structures in Figure 30), Cooper and co-workers showed the possibility of designing and preparing a solid-state solution ternary crystal with the interactions that occur between the porous cages that involve chiral recognition.^168^ In this solid, cage **CC1** holds together cages **CC3**-*R* and **CC4**-*R* without requiring stoichiometric ratios. The authors state that finely tuning composition to optimize porosity may be useful for molecular separations or catalysis (Figure 69).^169^ By making minor changes in the molecular structure and modifying the crystallization conditions, it is possible to modify the crystal packing of cages, which allows uncommon ternary cocrystals to be prepared.^170^ More complex crystal structures based on core–shell crystal packing have also been prepared, which permit synergistic combinations to fine-tune chemical shell composition that enables the control of properties like surface hydrophobicity or CO_2_/CH_4_ selectivity.^171^

**Figure 70:**
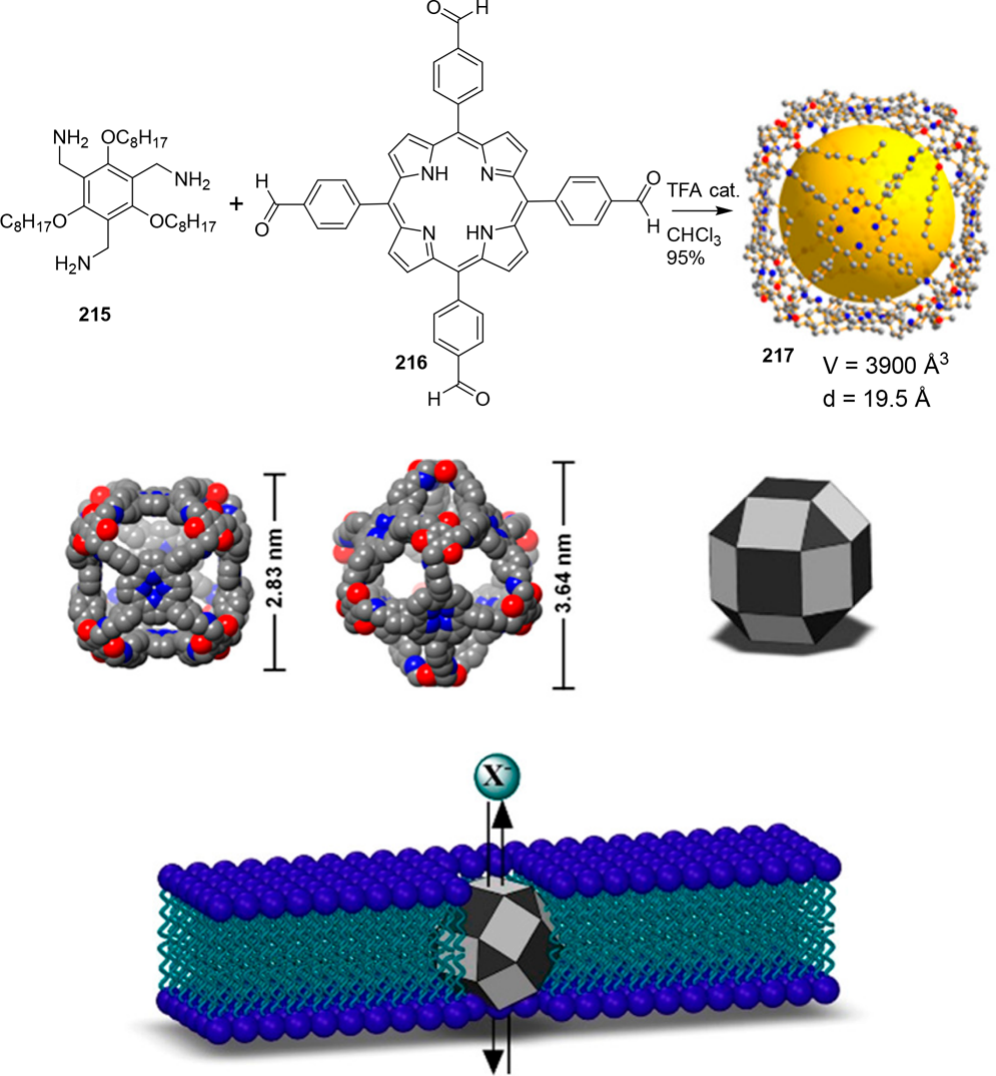
Synthesis and structure of cage **217**. Schematic representation ion channel formed by the cage in a lipid bilayer membrane.^172^ Adapted with permission from ref (172). Copyright 2017 American Chemical Society.

Kim, Roh, and co-workers used a shape-persistent hydrophobic organic cage as a synthetic ion channel. By means of the condensation of triamine **215** and tetra(aldehyde) porphyrin **216** in CHCl_3_ in the presence of catalytic amounts of trifluoroacetic acid, the authors prepared porphyrin-based covalent organic cage **217** in 95% yield. The obtained cage has a cavity diameter of ∼1.95 nm and 12 windows with average diameters of ∼3.7 Å. The authors used the cage embedded in a lipid bilayer to study anion transport through the membrane. The anion transport selectivity across the lipidic membrane follows the Hofmeister series: I^–^ > NO_3_^–^ > Br^–^ > Cl^–^ > SO_4_^2–^. This correlation between transport efficacy and anion hydrophobicity implies that anion dehydration is crucial for their transport through cage **217** (Figure 70).^172^

**Figure 71:**
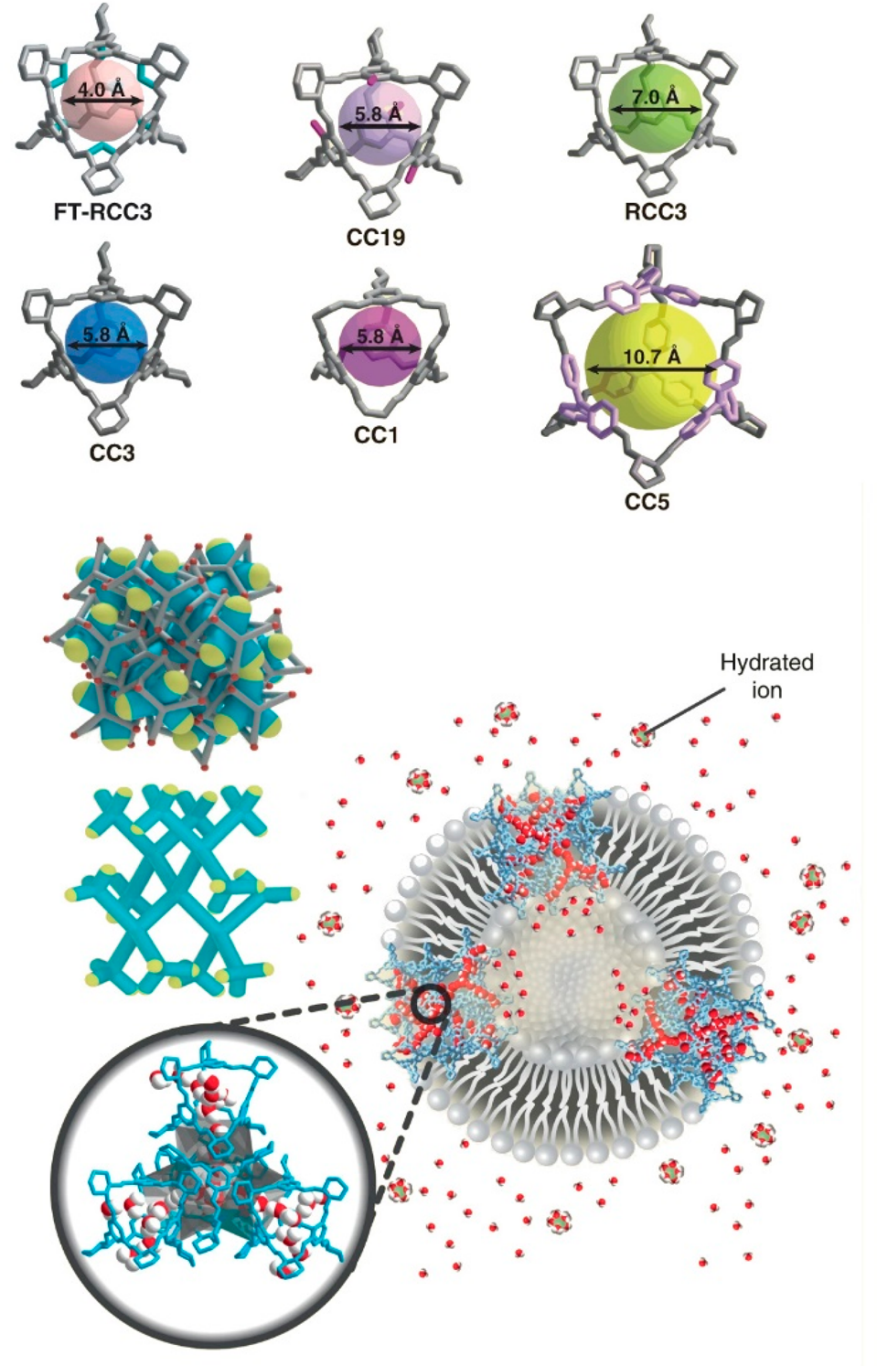
Structure of several Cooper’s **CC***n* tetrahedral imine organic cages, packing and nanoaggregates **CC3** in a lipid bilayer with water chains formed inside the channels.^174^ Reproduced with permission from ref (174). with the Creative Commons CC BY license http://creativecommons.org/licenses/by/4.0/. Copyright 2020 the authors of the original publication.

Zao and co-workers also described organic cages as water channels across a lipid bilayer. To do so, they used Cooper’s **CC***n* tetrahedral imine organic cages containing four triangular windows and one central cavity. These cages had already been used by Cooper and co-workers for reversible water uptake, which proved their ability to host the water molecules in the cavity.^173^ The authors employed six different cages (i.e., **CC1**, **CC3**, **RCC3**, FT-**RCC3**, **CC5**, **CC19**) to study water permeability and salt rejection properties. Water transport experiments were performed by embedding cages in liposomes formed by a lipid bilayer. The authors found that cages prevented the transport of small cations and anions but allowed fast water permeation at approximately 10^9^ water molecules per second. The most important factors to determine water and ion permeation are the pore window size, cage structural rigidity, and hydrophilicity, and their ability to form interconnected channel networks. As cage systems are highly symmetric, employing them as water channels did not depend on their orientation, which facilitates their use. As potential applications, the authors suggested using these cages in composite materials like membranes for water desalination (Figure 71).^174^

#### 3.2.2 Hydrazone

**Figure 72:**
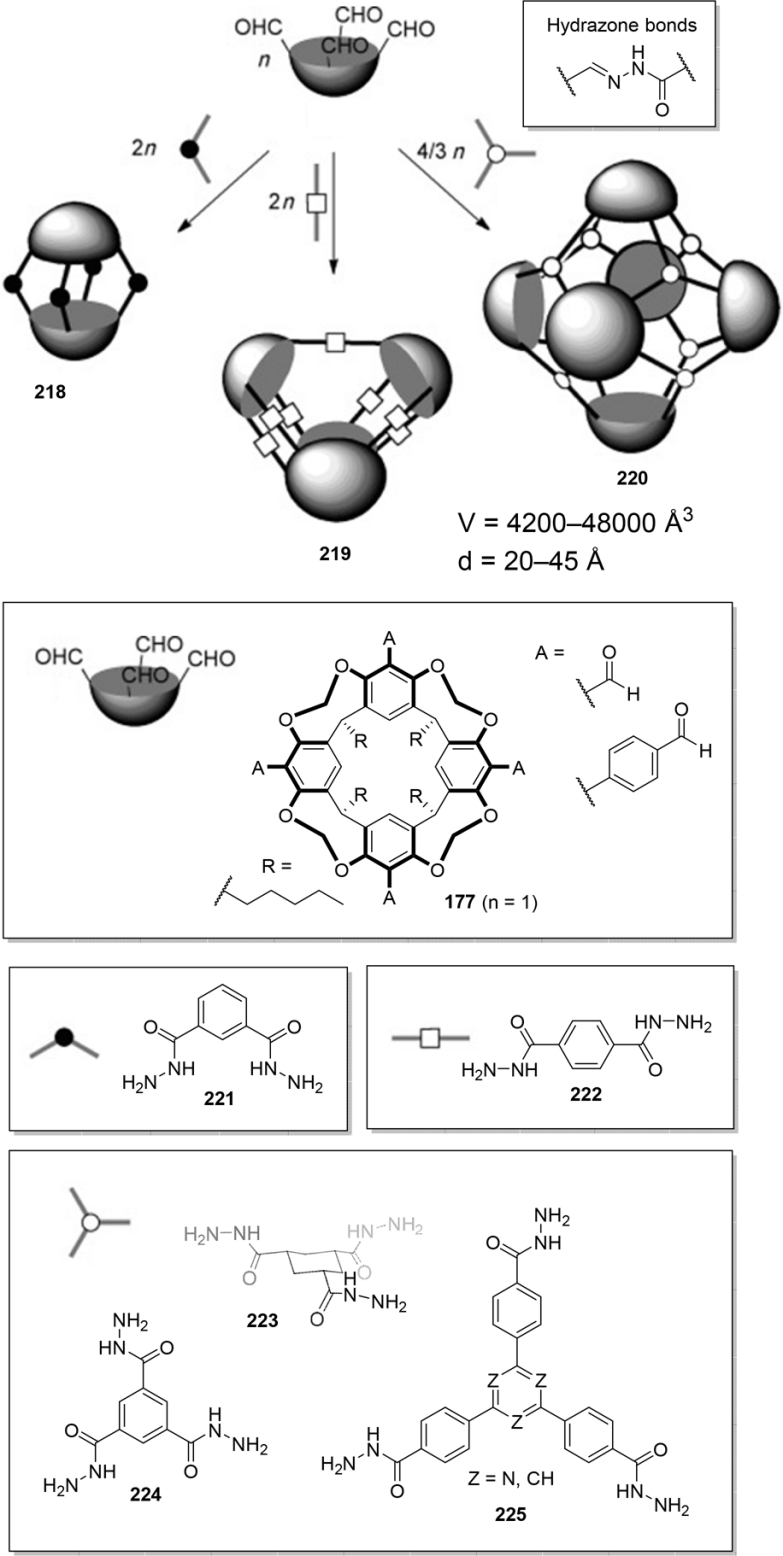
Hydrazone capsules **218**, **219**, and **220**.^175^ Adapted with permission from ref (175). Copyright 2011 Wiley-VCH.

Like reversible imine bond formation, hydrazone bond formation is reversible under acidic conditions, such as trifluoroacetic acid, and allows error correction mechanisms during the self-assembly process of binding blocks to finally obtain the stablest thermodynamic cage product. Compared to imine bonds, which have relatively limited stability, hydrazones are thermodynamically and kinetically more stable. With this concept, Warmuth and co-workers replaced the amino groups in their previously described building blocks (see Figure 15, Figure 16, and Figure 54) with a hydrazide group (C(O)NHNH_2_) and prepared the [2 + 4]-, [4 + 8]-, and [6 + 12]-poly acylhydrazone capsules **218**, **219**, and **220**. The reaction of tetraformylcavitand **177** with hydrazides **221**–**225** resulted in cage formation yields above 95% for some reported cages. Cages had cavity diameters ranging from 2 to 4.5 nm. The authors found that carefully choosing building blocks is crucial for obtaining the desired capsule structure and minimizing the formation of other capsules as side products. For this purpose, the linker strain in the cage structure must be as low as possible (Figure 72).^175^

**Figure 73:**
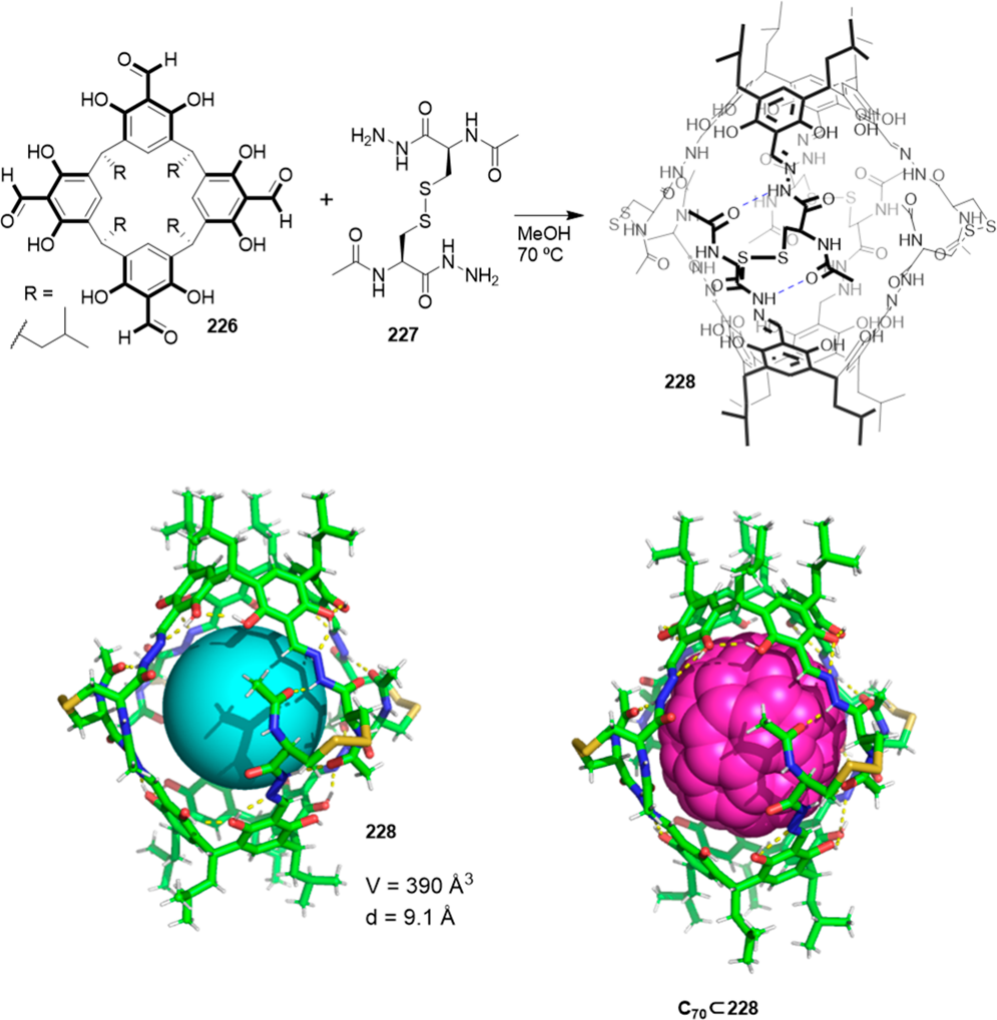
Synthesis of capsule **228** (top). The X-ray structure of capsule **228** and complex C_70_⊂**228** (bottom).^176^

Szumna, Potrzebowski, and co-workers used the cavitand **226** and cystine-based ligand **227** to prepare capsule **228**. This capsule was used for mechanochemical encapsulation in the solid state of fullerenes. Despite capsules not possessing large enough entrance windows, the disulfide and hydrazone bonds present in the structure of the cage became sensitive to mechanical stress and were activated by a mechanism in which mechanical stress energy facilitated the breaking, exchange, and reforming of the reversible chemical bonds to transform mechanical energy into chemical energy. This activation is specific for flexible porous cage structures because the activation of disulfide and hydrazone bonds does not take place in small molecules, nonporous or conformationally deformable capsules. Under solvent-free conditions, capsule pores are empty, and the cage is less stable and prone to collapse when subjected to mechanical stress if the cage structure is not rigid. The authors point out that neat milling is a promising encapsulation method that can be especially useful in the future in those cases that require breaking capsule covalent bonds for encapsulation or to perform guest encapsulation under solvent-free conditions to avoid the solvent competition that reduces guest affinity (Figure 73).^176^

**Figure 74:**
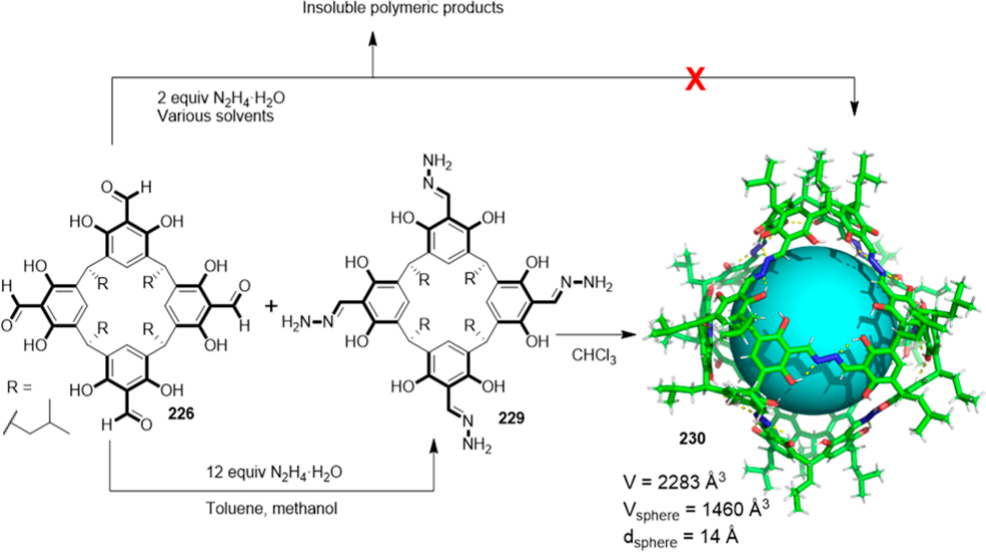
Synthesis of hexameric hydrazone cage **230**.^177^

From tetraformylresorcin[4]arene **226** and hydrazine, Szumna and co-workers were not able to obtain the target cage **230**. However, the modification of the aldehyde groups in tetraformylresorcin[4]arene **226** with hydrazine, to yield calixarene **229**, and the further reaction with unmodified tetraformylresorcin[4]arene **226** resulted in cage **230** formation in quantitative yields. The obtained cage has a cavity volume of 2283 Å^3^, and the structure is intrinsically chiral due to the directional arrangement of the hydrazone groups and the rigidity of the structure stabilized by hydrogen bonds. On the basis of this property, partial separation of enantiomers was achieved by chiral HPLC using **230** (Figure 74).^177^

#### 3.2.3 Hydrogen-Bonded Capsules

**Figure 75:**
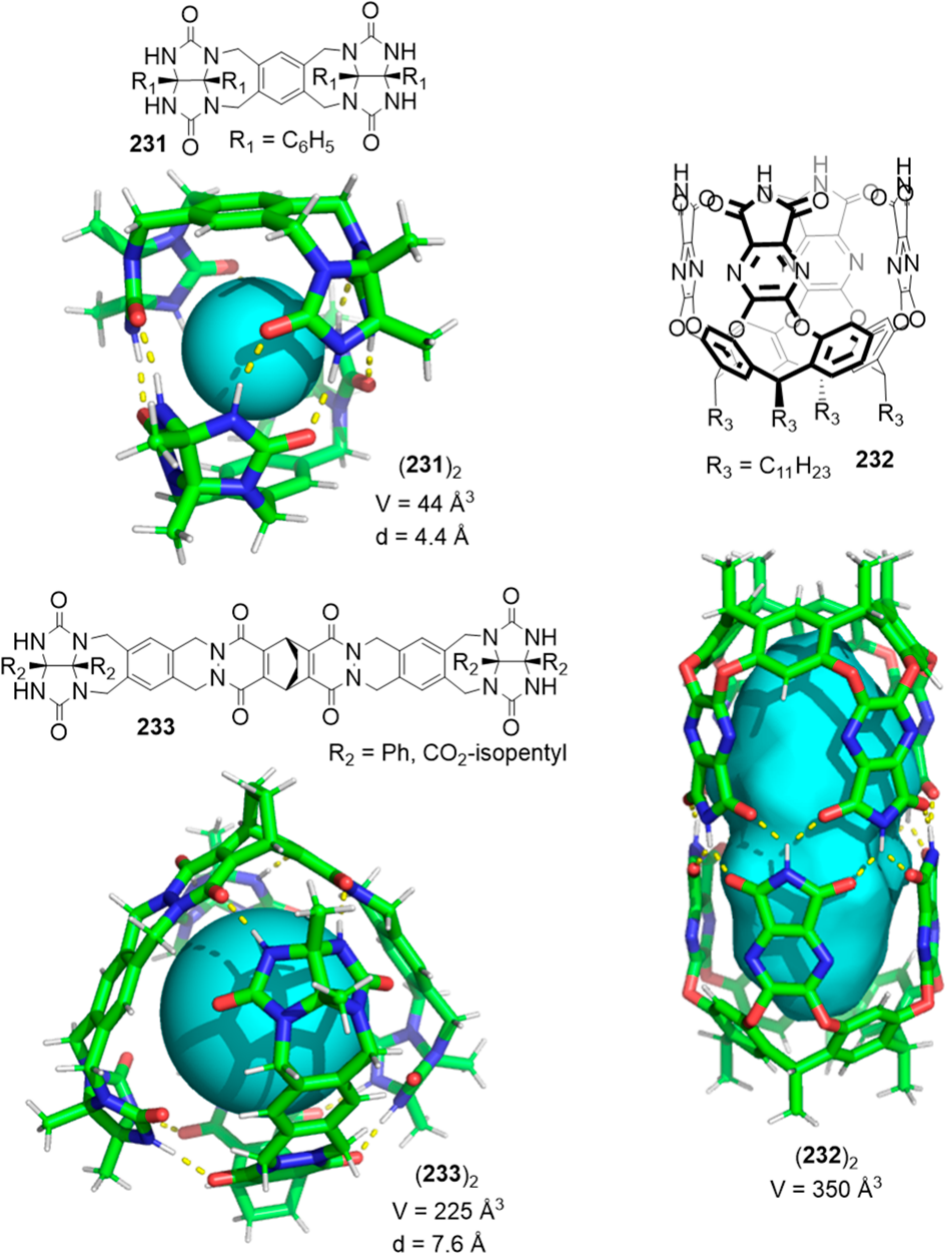
Rebek’s hydrogen-bonded capsules (**231**)_2_, (**232**)_2_, and (**233**)_2_.^178−180^

Hydrogen-bonding interactions can be used as the driving force to self-assemble organic cages from building blocks with an appropriate concave conformation (see structures **231**–**233** in Figure 75). Pioneering work on hydrogen-bonded capsules was done by the group of Rebek, who back in 1993 developed a molecular capsule (widely known as the “tennis ball”) held by complementary hydrogen bonding (**231**)_2_^178^ and later more robust assemblies like dimeric capsules (**232**)_2_^179^ and softballs (**233**)_2_,^180^ among others.^181^ These capsules have a hydrophobic cavity that allows encapsulating organic molecules, such as benzene, toluene, adamantane derivatives, dicyclohexyl carbodiimide, etc. This groundbreaking strategy has been further developed over decades, allowing the preparation of numerous and imaginative hydrogen-bonded capsules.^182,183^

**Figure 76:**
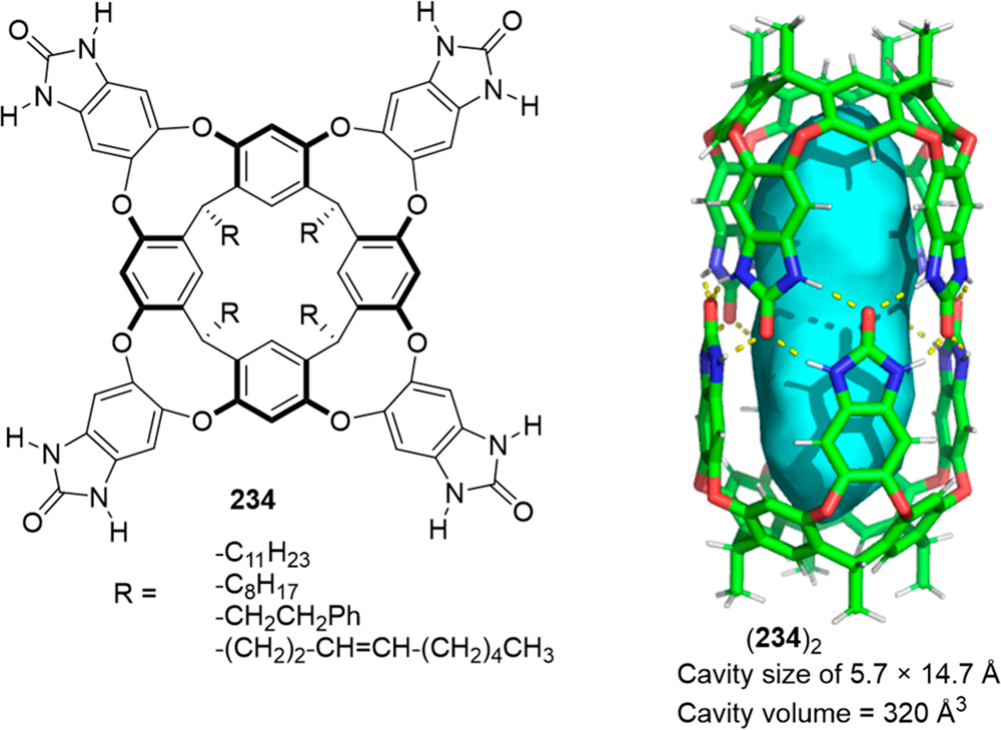
Resorcinarene **234** with 2-benzimidazolone bridges and the corresponding hydrogen-bonded dimeric (**234**)_2_ capsule.^184^

On the basis of this strategy, Mendoza and co-workers reported the self-assembly of resorcinarene **234** containing 2-benzimidazolone bridges that are held together by a cyclic array of complementary hydrogen bonds to result in capsule (**234**)_2_ with a cavity size of 5.7 Å × 14.7 Å. The cage behaves similarly to Rebek’s parent cage, and it encapsulates propionic, pivalic, cyclohexanecarboxylic, or 1-adamantanecarboxylic acids (Figure 76).^184^

**Figure 77:**
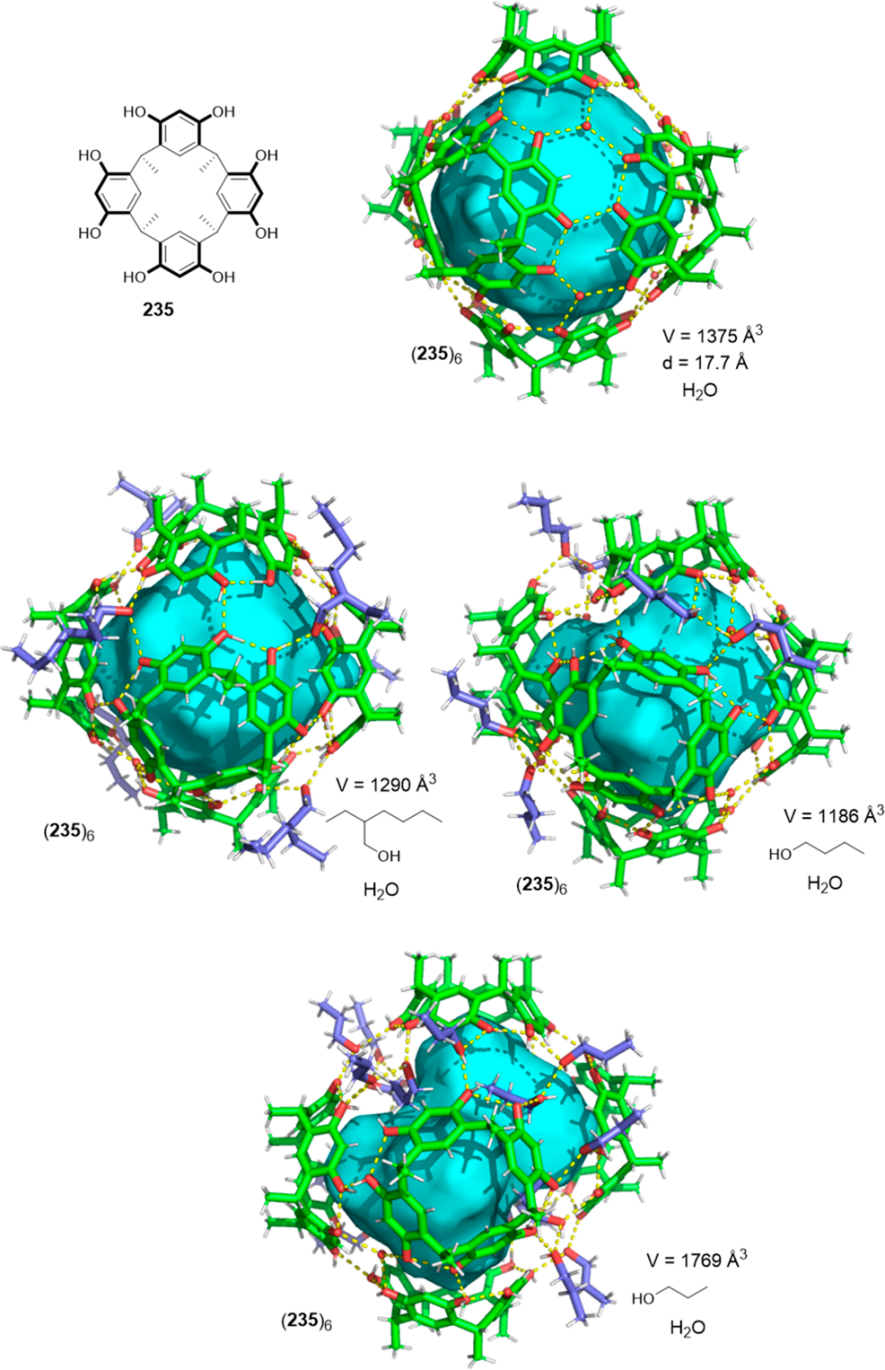
Hexameric (**235**)_6_ hydrogen-bonded capsule reported by Atwood and MacGillivray. Hexameric capsules stabilizes with water; 2-ethylhexanol and water, 1-butanol and water, and 1-propanol and water.^185−188^

Larger assemblies can be obtained by increasing the angle of the hydrogen-bonding motifs in the calixarene building block. In 1997, Atwood and MacGillivray reported the ability of cavitand **235** to self-assemble in spherical hexameric structure (**235**)_6_ in nonpolar solvents to form the largest molecular cage reported until that time. The structure is stabilized by 60 cooperative hydrogen bonds, which involves eight water molecules and the six calixarene motifs that form the structure. The capsule has a central cavity with a diameter of 17.7 Å and a cavity volume of 1375 Å^3^ (Figure 77).^185^ The structure can also be stabilized by replacing some water molecules with 2-ethylhexanol, which results in a cage with a cavity volume of 1290 Å^3^,^186^ 1-butanol (cavity volume of 1186 Å^3^),^187^ and 1-propanol (cavity volume of 1769 Å^3^). The last one corresponds to a 25% cavity volume increase caused by the 1-propanol molecules that separate the cavitand building blocks of the hexamer (Figure 57 bottom).^188^

**Figure 78:**
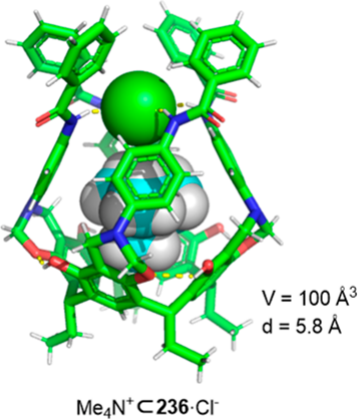
Encapsulation of Me_4_N^+^ in capsule **236** with halides (Cl^–^, Br^–^, and I^–^) to seal the calixarene cavity.^189^

Besides the direct hydrogen-bonding interactions between the constituent building blocks, the cage cavity can also be closed by the combination of the calixarene organic moiety and inorganic anions. Following this approach, Atwood and co-workers reported ion pair recognition in a molecular capsule (**236**) by summing the electrostatic and hydrogen-bonding interactions to form 1:1 host–guest complexes with tetramethylammonium salts and with halides Cl^–^, Br^–^, and I^–^ as counterions. The Me_4_N^+^ cation is fully encapsulated inside the cavity and the halide anion seals the calixarene cavity by hydrogen bonding (Figure 78). Encapsulation takes place in solvents like CDCl_3_ and CDCl_3_/MeOD 9:1, whereas cation release occurs in DMSO.^189^

**Figure 79:**
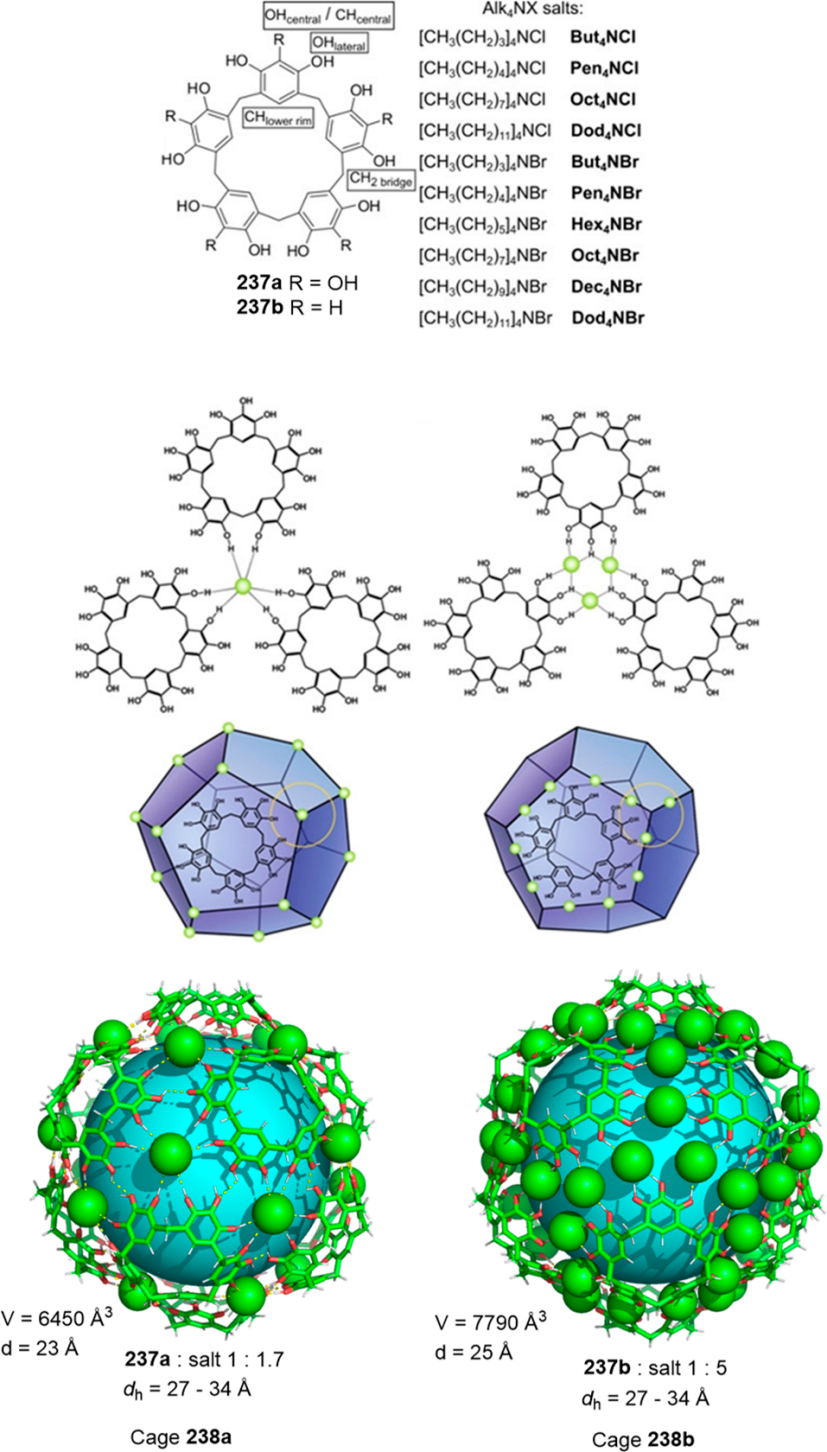
Anion-induced self-assembly of 12 units of 5-fold symmetry calixarene (pyrogallol[5]arene **237a** or resorcin[5]arene **237b**) and 60 anions (chloride or bromide) to give cages **238**. *d*_h_ = hydrodynamic diameter.^190^ Adapted with permission from ref (190). Copyright 2021 Wiley-VCH.

By using the same idea of putting the hydrogen-bonding interactions between calixarene and inorganic anions to good use, Szumna and co-workers prepared exceptionally large capsules with a dodecahedron geometry and a diameter of 30 Å by the self-assembly of 12 units of 5-fold symmetry calixarene (pyrogallol[5]arene **237a** or resorcin[5]arene **237b**) and 60 anions (chloride or bromide). The structure is stabilized by hydrogen bonds involving phenolic OH groups from the calixarene derivatives and anions. Titration curves of different **237a**:salt ratios suggest the presence of distinct cage structures, such as **238a** and **238b** (see Figure 79 bottom). Cage stability very much depends on the solvent. So whereas capsules are formed in THF and benzene, they do not exist in chloroform because the solvation of anions is stronger in this medium, which disfavors the interaction of anions with the calixarene building blocks (Figure 79).^190^

**Figure 80:**
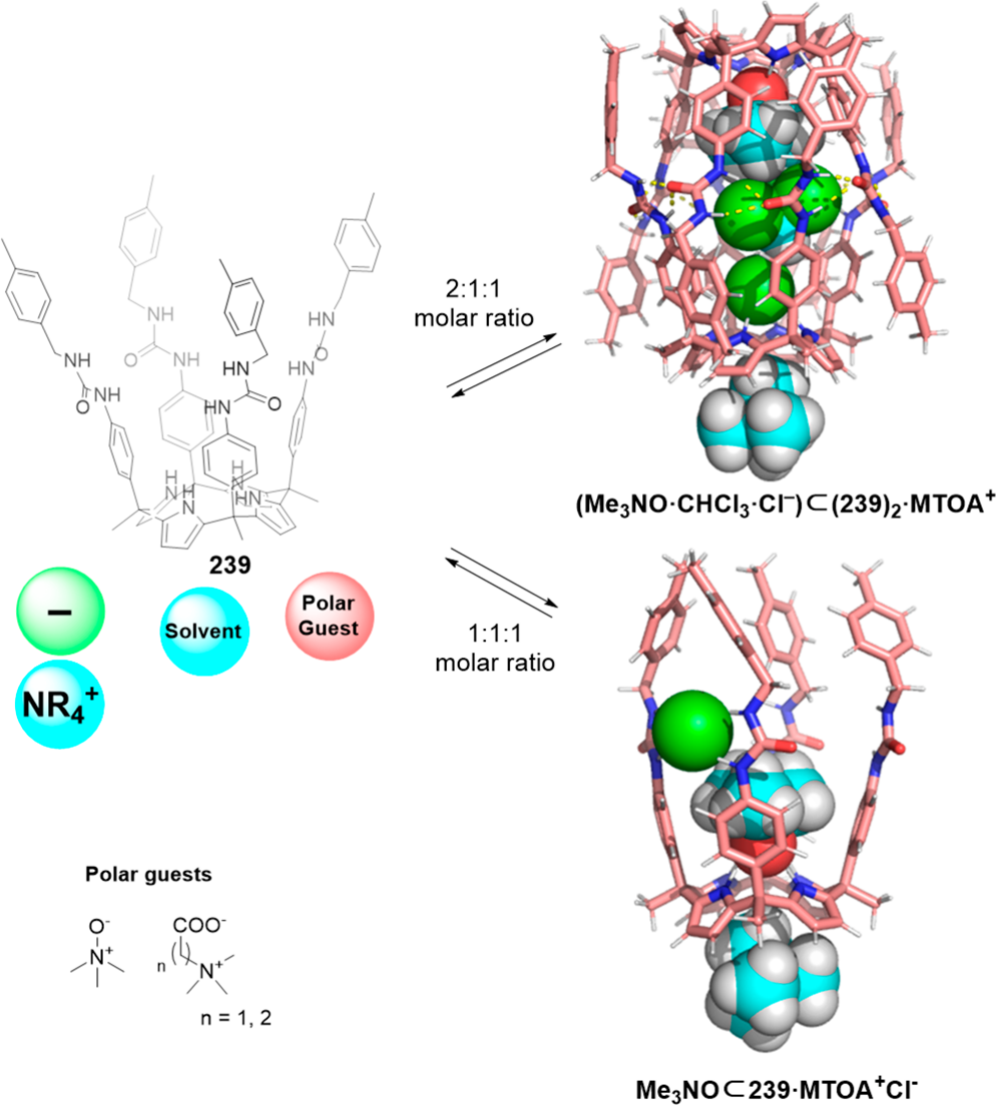
Structure of capsular assembly (Me_3_NO·CHCl_3_·Cl^–^)⊂(**239**)_2_·MTOA^+^ and Me_3_NO⊂(**239**)_2_·MTOA^+^Cl^–^. The alkyl chains in MTOA are hidden for clarity.^193^ Reproduced with permission from ref (193). Copyright 2015 the authors of the original publication. Published by the Royal Society of Chemistry with the Creative Commons CC BY license http://creativecommons.org/licenses/by/4.0/.

Conversely to the hydrophobic interior of calixarenes,^191^ calixpyrroles have a polar cavity that can efficiently bind to anions to fix the calixpyrrole in a cone conformation.^192^ Ballester and co-workers reported the use of a calix[4]pyrrole derivative **239** containing a urea group to generate a dimeric capsular assembly (**239**)_2_ with multiple ordered coencapsulated guests (including polar guests trimethylamine-*N*-oxide, 2-(trimethylammonio)acetate, and 3-(trimethylammonio)propanoate, Figure 80) when the stoichiometry of components is set at the 2:1:1 molar ratio (calix[4]pyrrole/polar guest/methyltrioctylammonium chloride (MTOA^+^Cl^–^)). In contrast, a 1:1:1 molar ratio exclusively produced the encapsulation of the polar guest in the cavitand with no dimeric capsular assembly formation (see the example described in Figure 80). Of the different combinations of assayed guests, the authors concluded that to achieve capsule formation, using a chloride counterion in MTOA^+^ salt was necessary. In addition, the sum of the volumes of all the encapsulated guests needed to come closer to the optimum 55% capsule cavity occupancy value (Figure 80).^193^

**Figure 81:**
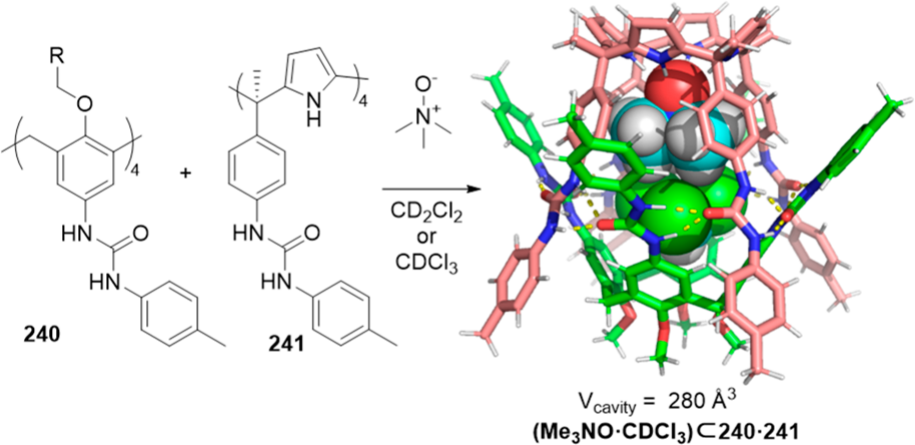
Self-assembly of calix[4]arene 240 and calix[4]pyrrole **241** in the presence of Me_3_NO to give dimeric capsule (Me_3_NO·CDCl_3_)⊂**240·241**. R = (CH_2_)_3_CH_3_. R groups are replaced with Me groups in the 3D structure for simplicity.^194^

More complex assemblies, such as heterodimeric assemblies, can also be prepared using two different cavitand molecules and the appropriate template. By this strategy, in another work, Ballester and co-workers reported the self-assembly of tetraurea-calix[4]arene **240** and tetraurea-calix[4]pyrrole **241** in dimeric capsules **240**·**241** with a cavity volume of 280 Å^3^. Self-sorting requires the presence of a trimethylamine-*N*-oxide guest that can modulate the outcome of the reaction to exclusively yield the heterodimeric capsule using CD_2_Cl_2_ or CDCl_3_ as the solvent (see structure (Me_3_NO·CDCl_3_)⊂**240**·**241** in Figure 81). The different properties of the cage cavity, hydrophobic calixarene and polar calixpyrrole drive molecular recognition and the position of guests in the cavity. The polar part of the trimethylamine-*N*-oxide guest forms hydrogen bonds with calixpyrrole moiety, and the nonpolar part interacts with the calixarene site. In addition to the trimethylamine-*N*-oxide guest, one solvent molecule is coencapsulated at the calixarene site (see structure in Figure 81).^194^

**Figure 82:**
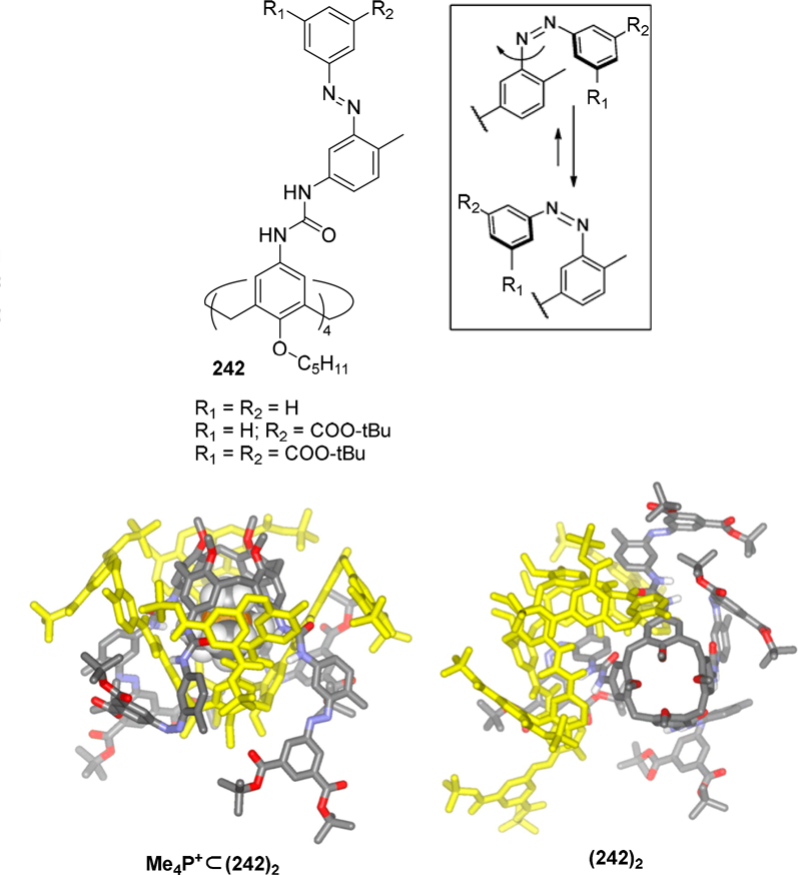
Chemical structure of the tetraureacalix[4]arenes **242** functionalized with the terminal azobenzene groups and capsular assembly Me_4_P^+^⊂(**242**)_2_ and noncapsular (**242**)_2_ dimer.^195^ Adapted with permission from ref (195). Copyright 2015 American Chemical Society.

Ballester and co-workers also reported that responsive capsules were obtained by incorporating light-responsive groups into a calix[4]pyrrole building block. In particular, the authors synthesized tetraureacalix[4]arenes **242**, which are functionalized with terminal azobenzene groups in the *trans* conformation. These *trans* calixarenes quantitatively dimerize in CD_2_Cl_2_ in the presence of the Me_4_P^+^ cation, which is additionally encapsulated in the cavitand (**Me**_**4**_**P**^**+**^⊂**(242)**_**2**_). When samples are irradiated with light, the *trans* azobenzene groups isomerize to the *cis* isomer, which results in a reduced cavity size that leads to a partial Me_4_P^+^ release with no cage degradation. The inclusion of bulky groups in the terminal phenyl group in azobenzene moiety played a key role in controlling the amount of released cargo (12–70%), which was larger when two tertbutoxy carboxyl groups were included. (Figure 82).^195^ The authors suggested that a further increase in the bulkiness of the substituents in the terminal phenylazo groups could result in a complete cargo release by *trans*–*cis* photoisomerization.

**Figure 83:**
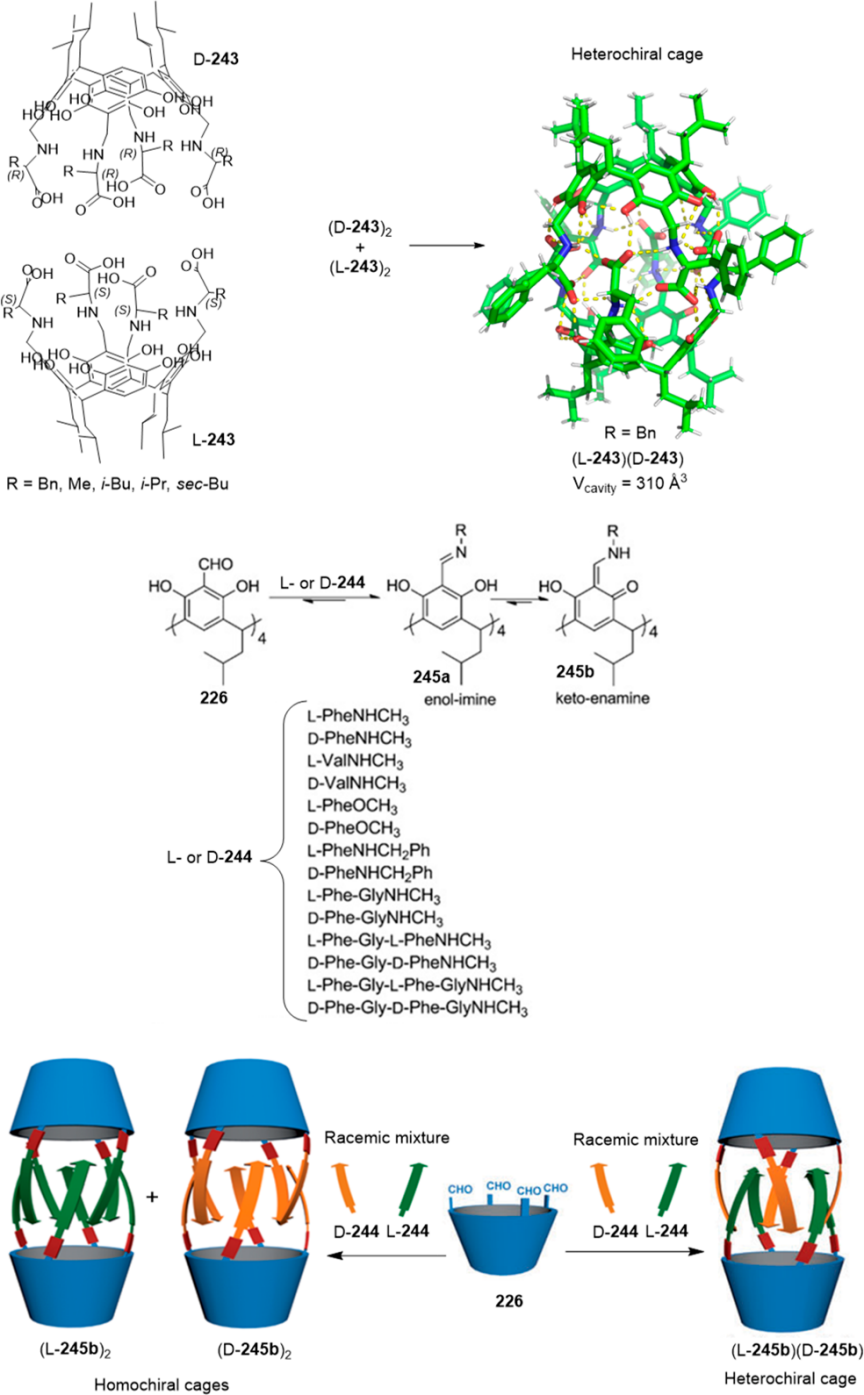
Heterochiral (top) and homochiral (bottom) capsules obtained by self-sorting cavitands **243** and **245**).^196,198^ Adapted with permission from ref (198). Copyright 2014 Wiley-VCH.

**Figure 84:**
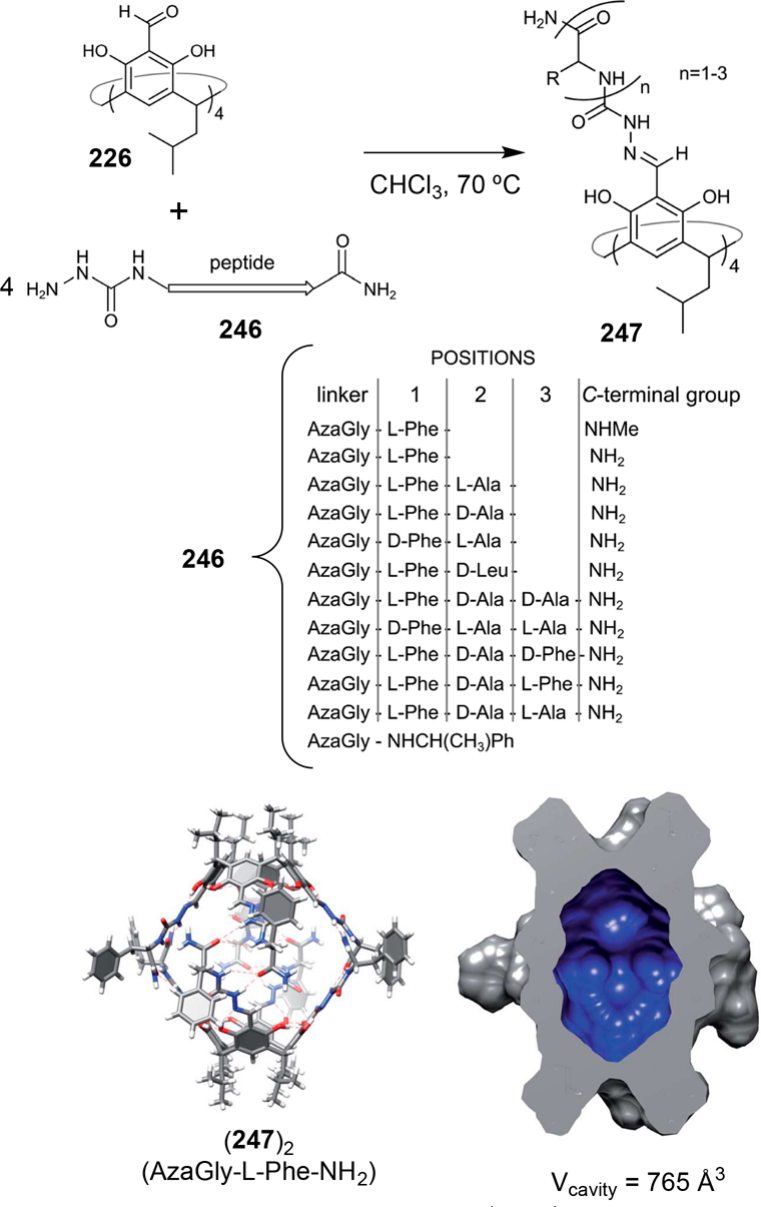
Cavitands **247** and capsules (**247**)_2_ formation. Modeled structure of capsule (**247**)_2_ with peptide chain AzaGly-l-Phe-NH_2_ in solution based on 2D NMR data and its molecular surface and cavity volume (765 Å^3^).^199^ Adapted with permission from ref (199). Copyright 2019 the authors of the original publication. Published by the Royal Society of Chemistry with the Creative Commons CC BY license http://creativecommons.org/licenses/by/4.0/.

Szumna and co-workers prepared capsules with pockets that exploited the inherent functionality of peptides. To this end, the authors functionalized aldehyde groups in cavitand **226** with peptide chains by the Mannich reaction (cavitand **243**, Figure 83, top)^196,197^ with imine bonds with short peptides **244** (cavitand **245**, Figure 83, bottom)^198^ or with semicarbazone bonds with peptides **246** (cavitand **247**, Figure 84).^199,200^ In these systems, the complementarity between peptidic chains is key for the outcome of the self-assembled structure. The cages assembled through the Mannich reaction (Figure 83, top) produce heterochiral capsules obtained by self-sorting. Selectivity arises from the steric repulsions of homochiral cages, which destabilize the capsule assembly. This is evidenced by the quantitative self-sorting of a mixture of (d-**243**)_2_ and (l-**243**)_2_ to yield the formation of the heterochiral dimeric capsule (d-**243**)(l-**243**) by performing a few dissolve–evaporate cycles or treating it with 10% MeOH. This protocol is necessary because capsule dimers are kinetically stable and the mixture of (l-**243**)_2_ and (d-**243**)_2_ in CDCl_3_ remains unchanged for >14 days at room temperature or while heating for 48 h at 60 °C. The capsule has a cavity volume of 310 Å^3^ and encapsulates small polar molecules, such as EtOH or two nitromethane and four water molecules, as determined by X-ray crystallography.^197^ The reversible attachment of short peptides **244** to resorcinarene scaffold **246** (to give **245**) by imine bonds allows chiral self-sorting experiments to be run using a mixture of racemic peptides **244**. Depending on peptidic sequence **244**, the process was driven by self-assembly to yield homochiral (l-**245b**)_2_ and (d-**245b**)_2_ or heterochiral capsules (l-**245b**)(d-**245b**) (see the scheme in Figure 83, bottom).^198^

The same group found that in semicarbazone compound **247**, obtained from peptides **246** and tetraformylresorcin[4]arene **226**, the backbones in **247** are placed in an appropriate position for the self-assembly of dimeric capsules (**247**)_2_ to form a structure that resembles the eight-stranded β-barrels in noncompetitive solvent CDCl_3_. Unlike the crowded interiors of natural β-barrels, the capsule structure is not perfectly sealed because of the imperfect complementarity of peptide-binding motifs, and they, therefore, present windows for guest uptake and release. Capsules have cavities in the order of 800 Å^3^ (see the example in Figure 84 displaying a cavity with 765 Å^3^). After optimizing the capsule structure, the authors found that the peptide chains **246** containing three amino acids in an (L, D, D) sequence gave well-defined dimeric capsules, with their side chains positioned inside the capsule cavity. Additionally, bulky polar side chains inside the capsule were avoided, and the only methyl group from Ala was accommodated in the cavity. The reversibility of the semicarbazone bonds, obtained by the reaction between azapeptides and aldehydes, allowed an efficient chiral self-sorting reaction to be performed in situ based on cavitand peptide chains. The authors point out that the elongation of peptides and changes in the position of side chains can open up numerous possibilities to obtain systems that mimic natural catalytic sites (Figure 84).^199^ Using the (**247**)_2_ isostructural semicarbazone-based peptidic capsules obtained from cavitand **226** and different acylhydrazone linkers, which also have cavities in the order of 800 Å^3^, it is possible to achieve the quantitative encapsulation of fullerene C_60_ or C_70_ using the reversible character of the semicarbazone bonds by following mechanochemical methods in the solid state.^201^

**Figure 85:**
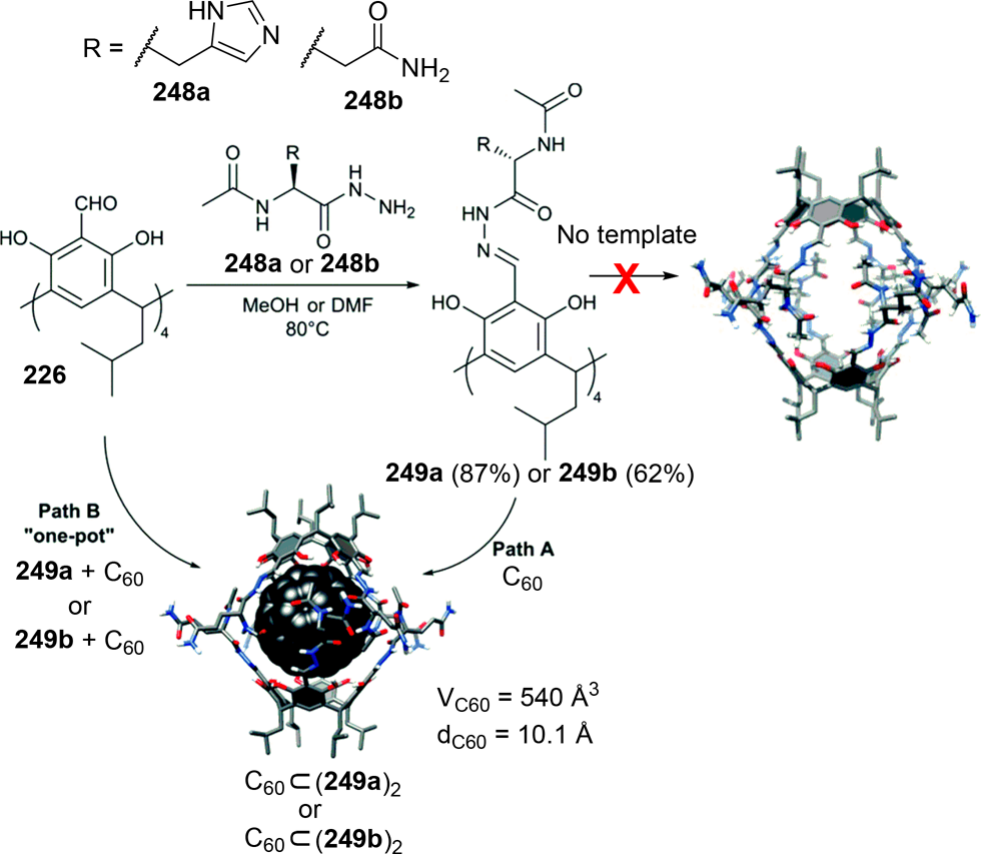
Formation of C_60_-containing peptidic capsules C_60_⊂(**249**)_2_.^202^ Adapted with permission from ref (202). Copyright 2017 the authors of the original publication. Published by the Royal Society of Chemistry with the Creative Commons CC BY license http://creativecommons.org/licenses/by/4.0/.

Szumna and co-workers also studied the effect of solvent polarity on the self-assembly of peptidic capsules, originally developed in nonpolar solvents (vide ante) like CDCl_3_. The authors prepared cavitands **249** that contained the more polar residues histidine (**248a**) and glutamine (**248b**) in the peptidic chain. However, this did not form sufficiently strong hydrogen bonding interactions to yield the cage in DMSO or methanol. This is not surprising because these solvents are often used to unfold natural proteins or breaking hydrogen-bonded aggregates. The authors also used C_60_ as a template to, in this case, allow complexes to be obtained in which C_60_ was wrapped by cavitands to form dimeric capsules C_60_⊂(**249a**)_2_ and C_60_⊂(**249b**)_2_. The cages containing C_60_ could be obtained by complexation during cavitand synthesis and also by heating for long time periods or by solid-state mechanochemical activation. In all cases, the resulting products contained a mixture of free cavitand **249** and the dimeric capsule with an encapsulated fullerene molecule (C_60_⊂(**249**)_2_). By using methanol, it was possible to isolate the C_60_-containing dimeric capsules due to solubility differences with the free cavitand (Figure 85).^202^

**Figure 86:**
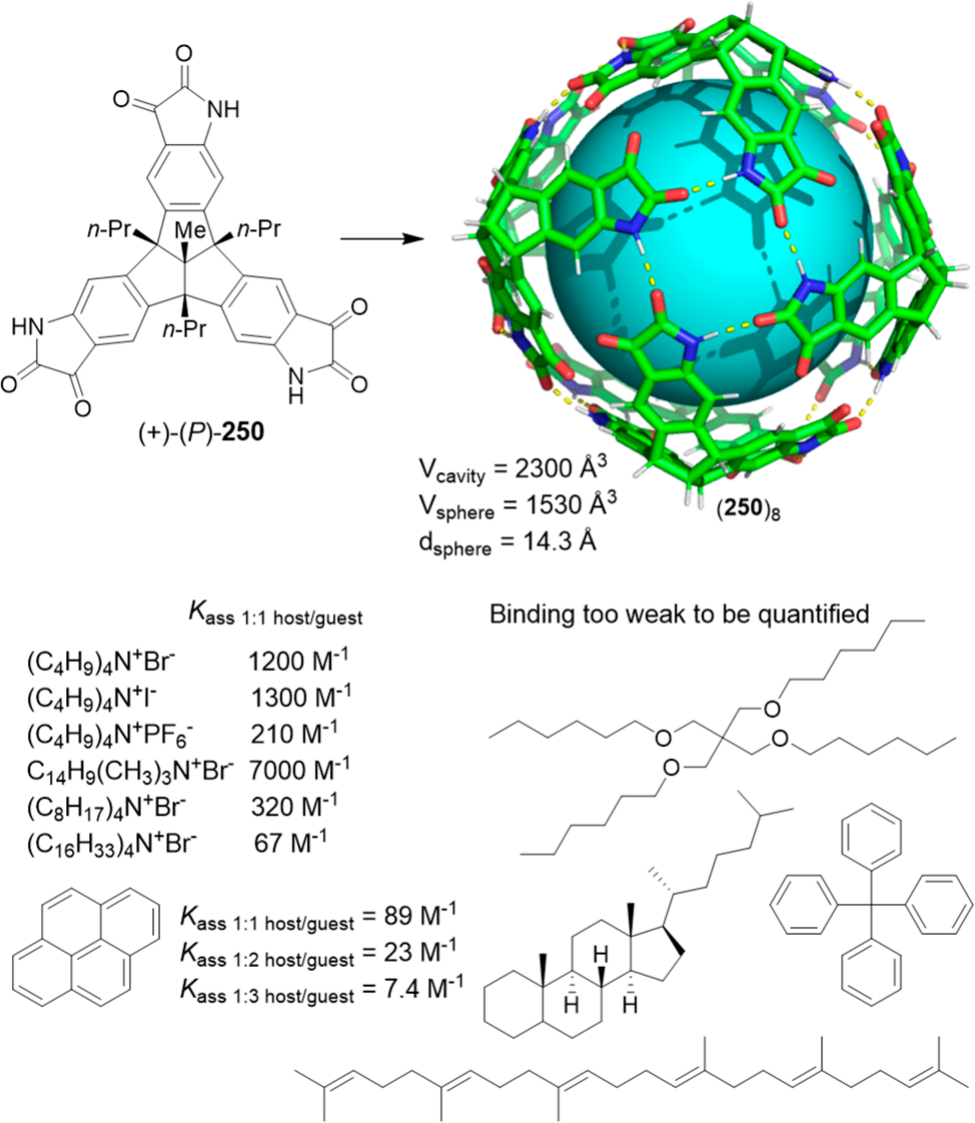
Structure of (+)-(*P*)-250, capsule (**250**)_8_ and binding constants with different guests.^203^

Mastalerz and co-workers reported the synthesis of octameric hydrogen-bonded capsule (**250**)_8_ from enantiomerically pure building block (+)-(*P*)-**250**. The chiral building block was designed to self-assemble in a single geometric orientation by reducing the formation of polymeric byproducts and producing a capsule with a cavity volume of 2300 Å^3^. The capsule was able to host different tetraalkylammonium salts with significant shape and size selectivity. For example, the association constant of *n*-C_14_H_29_(CH_3_)_3_NBr with the cage was 100-fold higher than that of (*n*-C_16_H_33_)_4_NBr (Figure 86).^203^

**Figure 87:**
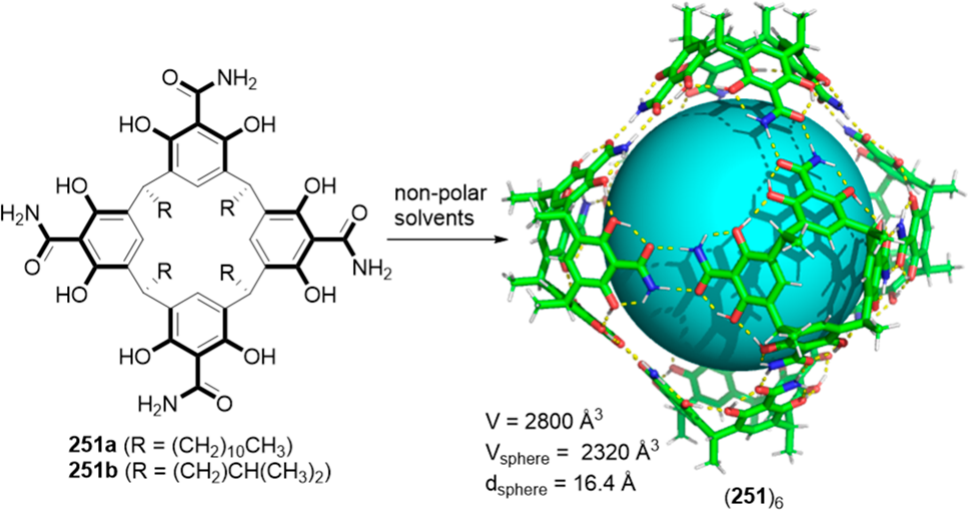
Hexameric hydrogen-bonded cage (**251**)_6_.^204^

Tiefenbacher and co-workers reported the hexameric cage (**251**)_6_ based on the self-assembly of calixarene building blocks **251** through intermolecular amide–amide interactions. The resulting structure was stabilized by 24 intermolecular hydrogen bonds and had a cavity volume of 2800 Å^3^, which is the largest cavity size reported to date for a hydrogen-bonded capsule or cage. Spontaneous cage self-assembly takes place in chloroform and other chlorinated solvents, and cage formation is favored at higher concentrations of monomers. As a reference, at the 1 mM concentration, the monomer is mainly observed, while at 50 mM concentration, the cage was the predominant species. The cage was demonstrated to encapsulate spherical C_60_ and C_70_ fullerenes through favorable dispersive and π–π-interactions between the guest and the aromatic rings in the cage cavity (Figure 87).^204^

**Figure 88:**
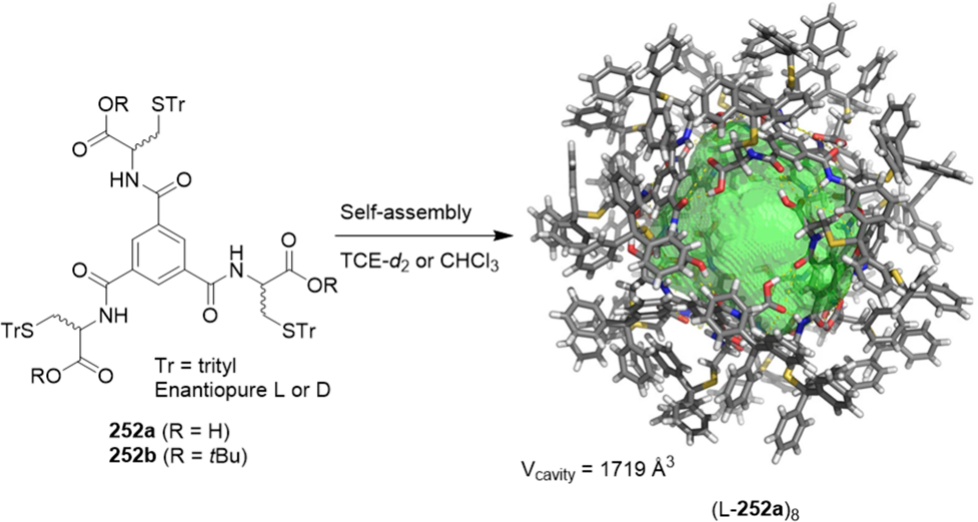
Hydrogen-bonded cage (**252**)_8_.^205^ Adapted with permission from ref (205) with the Creative Commons CC BY license http://creativecommons.org/licenses/by/4.0/. Copyright 2017 the authors of the original publication.

Markiewicz, Jenczak, and co-workers prepared a robust enantiopure octameric cage (**252**)_8_ held together by 48 cooperative hydrogen bonds between the **252** monomers. The capsule structure was retained in both the solid state, where crystals were obtained by the slow evaporation of a chloroform solution, and also in solution in nonpolar solvents, such as deuterated 1,1,2,2-tetrachloroethane (TCE-*d*_2_). Polar solvents like DMSO-*d*_6_ disrupted the hydrogen-bonding network of the cage and resulted in the free monomer. The large cavity size (*V*_cavity_ = 1719 Å^3^) allowed C_60_ and C_70_ to be encapsulated with preferential C_70_ binding due to structural and electronic complementarity (Figure 88).^205^

**Figure 89:**
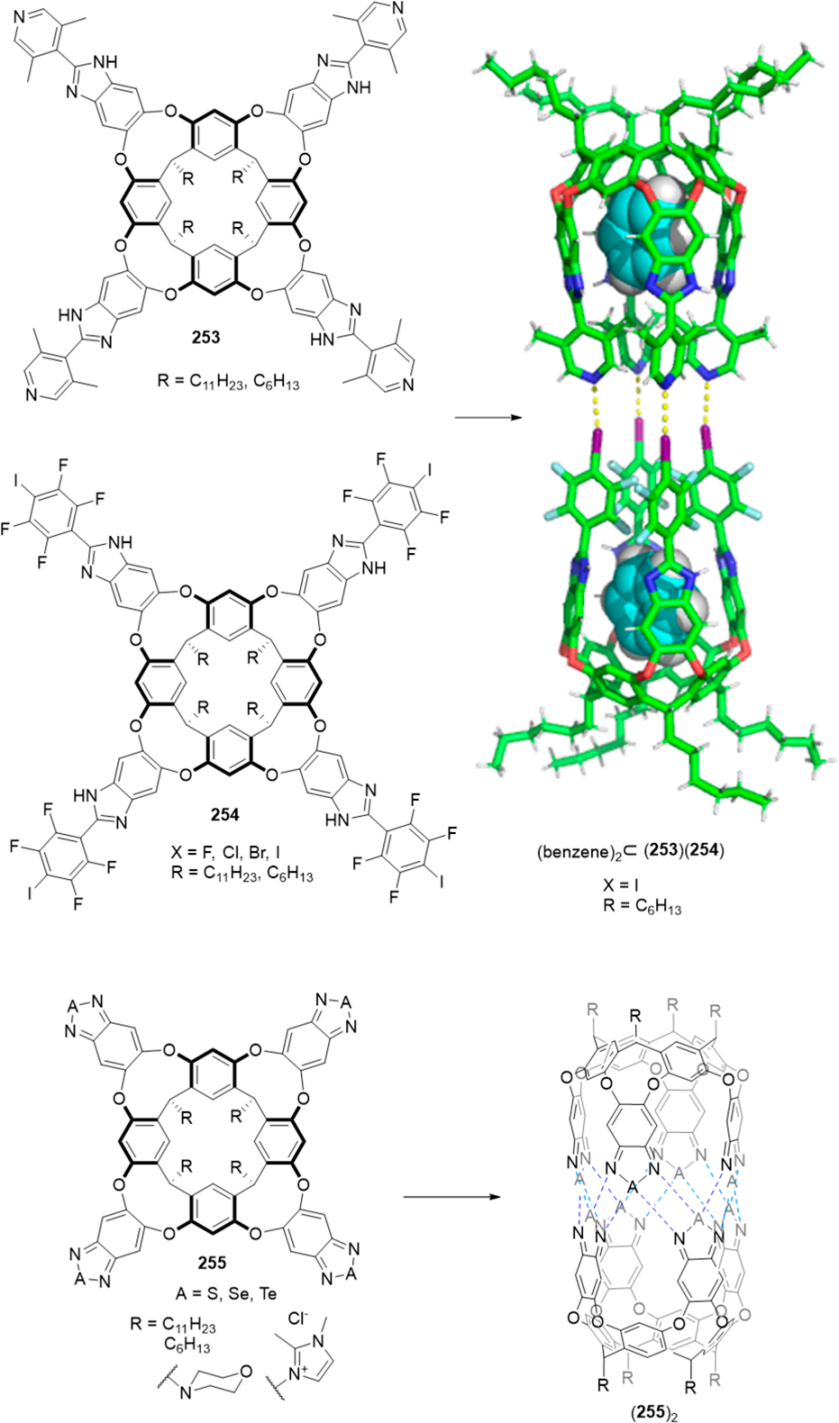
Capsules **253·254** and (**255**)_2_ formed through halogen and chalcogen bonding.^206−209^

Diederich and co-workers reported the first dimeric capsule **253**·**254** stabilized by halogen bonding (XB) from resorcin[4]arene cavitands containing tetrafluorohalophenyl motifs halogen bond donor groups (**254**) and lutidyl halogen bond acceptor groups (**253**). The X-ray solid-state structure of dimeric capsule **253**·**254** showed two encapsulated benzene molecules (Figure 89 top). Capsule assembly was observed for X = I and Br, but no capsule was found for X = F and Cl. Capsule formation was enthalpy-driven and entropically disfavored.^206,207^ The authors also reported the first chalcogen-bonded dimeric capsule (**255**)_2_ obtained from resorcin[4]arene cavitands **255** containing either tellurium or sulfur (Figure 89, bottom).^208^ Later Yu, Rebek, and co-workers prepared analogous chalcogen-bonded cages (**255**)_2_ containing selenium and water-solubilizing groups that formed stable dimeric capsules in water (Figure 89, bottom).^209^

#### 3.2.4 Boronate Esters

**Figure 90:**
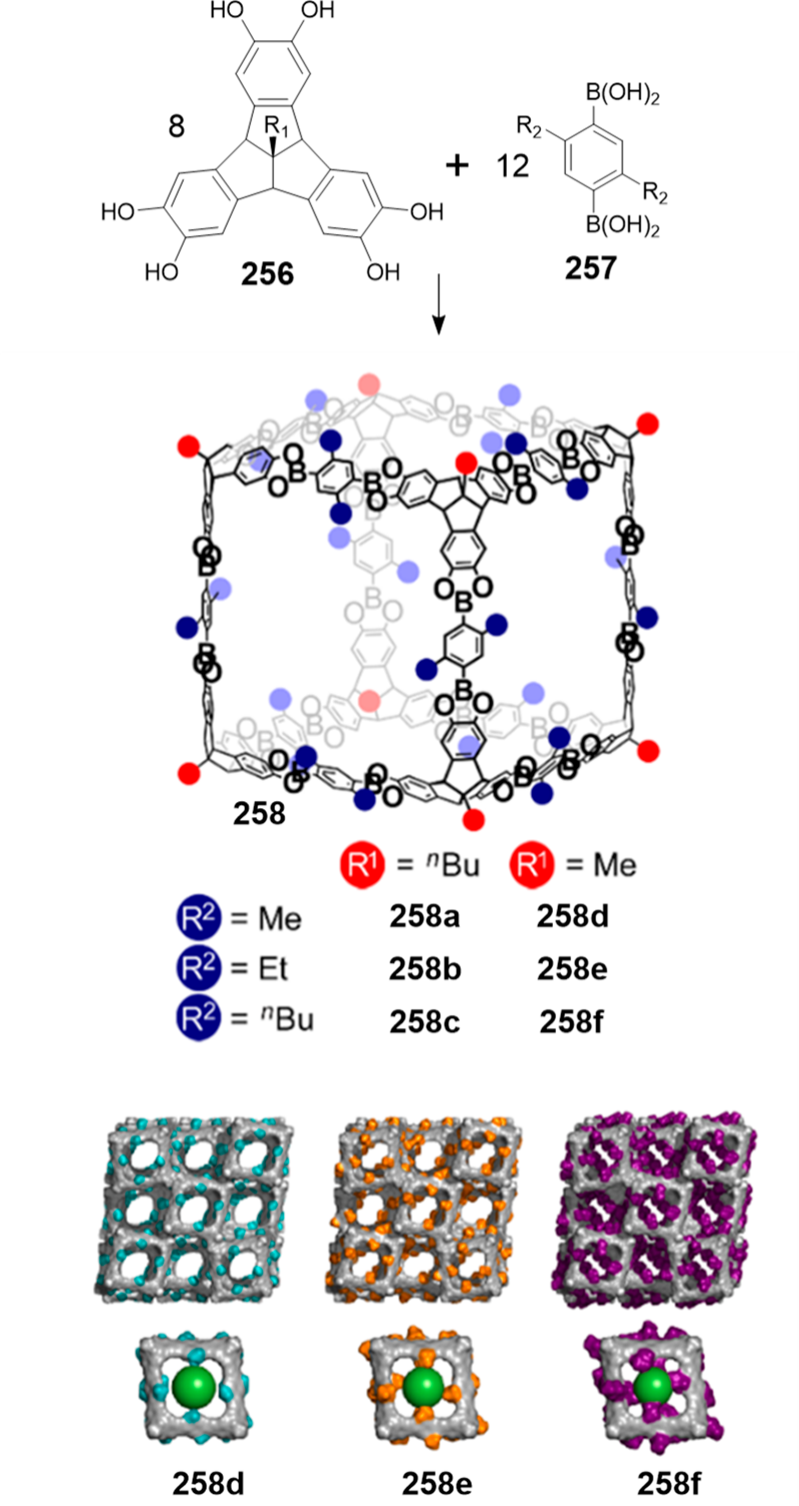
Formation of cube-shaped cages **258** and optical microscopy images of crystalline samples. Front view showing the parallel porous channels and detail of one pore window with a green sphere centered at the window displaying its size.^210,211^ Adapted with permission from ref (211). Copyright 2021 Wiley-VCH.

The use of reversible boronate ester bonds has also been employed for the self-assembly of cage structures. In particular, building blocks based on catechol and aryl-boronic acids have allowed the preparation of a wide range of cage structures. With boronate ester bonds, Beuerle and co-workers used catechol-functionalized tribenzotriquinacenes **256** and 1,4-phenylene diboronic acids **257** to self-assemble cube-shaped cages **258** (Figure 90). Cages **258** are the first examples of purely organic cubes with the highest possible cubic symmetry *O*_*h*_. The one-pot process involves the cross-linking of 20 individual components through dynamic boronate ester bonds. The authors predict that this strategy will help to prepare lower-symmetry cubic cages analogues.^210^ The authors also explored the effect on solubility by changing the alkyl substituents in the cage structure to find that solubility in organic solvents decreases gradually by shortening the alkyl chains at vertices. This structural modification modulated the size of the square pore windows to provide three different porous systems in the solid state: cage windows, extrinsic pores, and intrinsic cage cavities (see packing of **258d**, **258e**, and **258f** in Figure 90). The resulting materials are a rare example of organic cages that alternate micropores and mesopores with very large BET surface areas (N_2_, 77 K) of up to 3426 m^2^/g for **258f** (Figure 90 bottom).^211^ The authors point out that further modifications of systems, such as extending aromatic linkers or adding functional side chains, will allow the fine-tuning of materials’ porosity.

**Figure 91:**
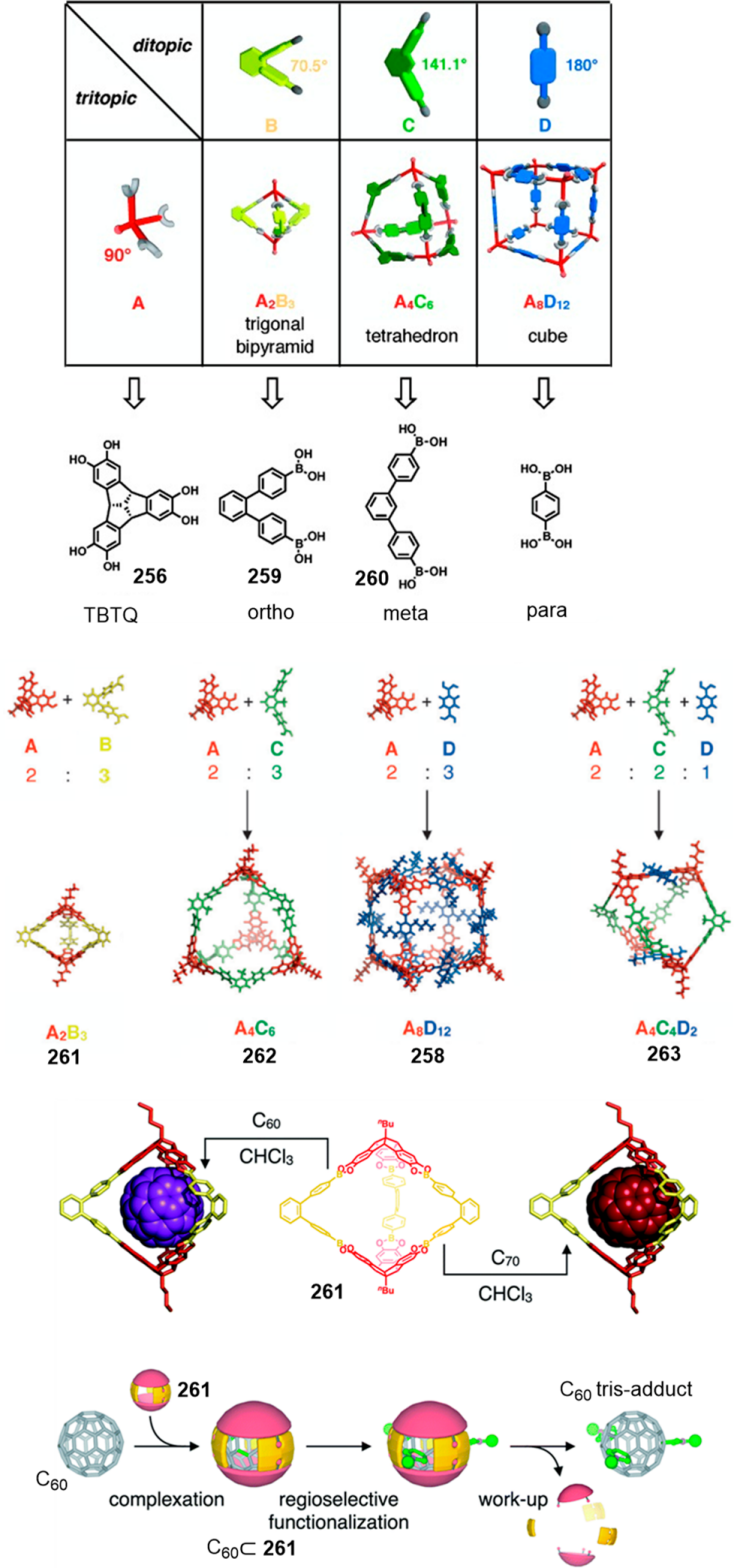
Building blocks **256**, **259**, and **260** and *p*-benzenediboronic acid for the formation of different boronate-ester cages.^212,214^ Adapted with permission from ref (212). Copyright 2015 Wiley-VCH. Adapted with permission from ref (214). Copyright 2020 the authors of the original publication. Published by the Royal Society of Chemistry with the Creative Commons CC BY license http://creativecommons.org/licenses/by/4.0/.

In addition to this family of cages, Beuerle and Klotzbach demonstrated that the bite angle of ditopic diboronic acid building blocks (**259**, 60°; **256**, 90°; **260**, 120°; and *p*-benzenediboronic acid, 180°) predictably defines the topology of the cage obtained by a reaction with the catechol-functionalized tribenzotriquinacene **256** (TBTQ) building block, with 90° between reactive sites (Figure 91 top). With these building blocks, it is possible to prepare cages with a trigonal-bipyramidal **261**, tetrahedral **262**, or cubic **258** geometry. Additionally, with building blocks **256**, **260**, and *p*-benzenediboronic acid, it is also possible to obtain a social self-sorted three-component cage **263**, which corresponds to the first example of this cage type. This methodology offers a controlled synthetic protocol to prepare cages with a predefined topology based on building blocks’ geometry (Figure 91).^212^ Additionally, the transformation of covalent organic frameworks based on **256** and *p*-benzenediboronic acid into molecular cage **261** is possible by linker exchange using ortho ligand **259**.^213^ Cage **261** efficiently encapsulates fullerenes C_60_ and C_70_. Besides, the cage acts as a masking template in the Prato reaction using C_60_⊂**261** by forcing reactions to happen at the guest positions, which implies that the windows of the cage provide selectivity for the unfavorable *N*-methylfulleropyrrolidine tris-adduct (Figure 91 bottom).^214^

**Figure 92:**
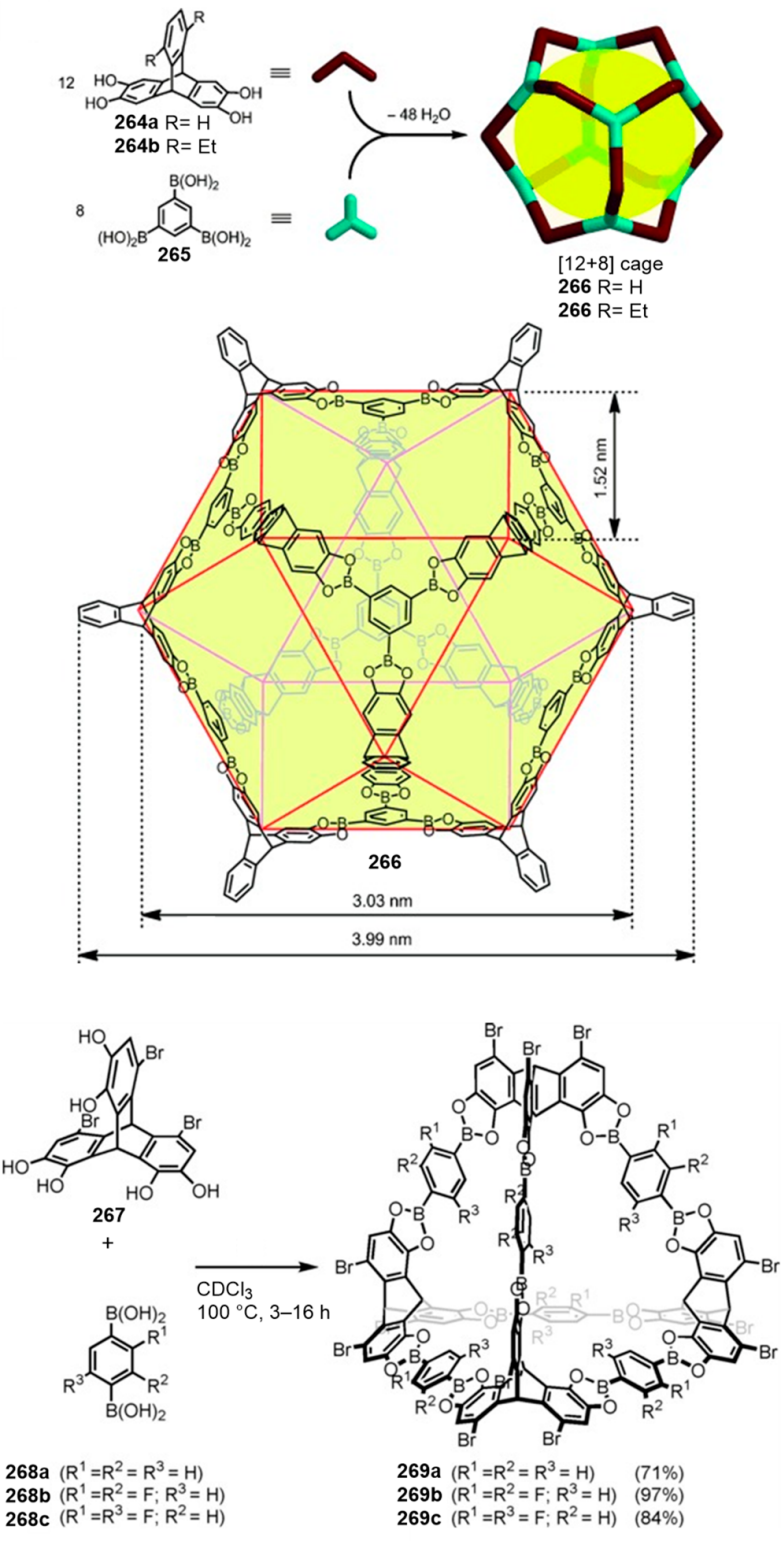
Formation of boronic cuboctahedral [12 + 8] **266** and tetrahedral [4 + 6] **269** cages.^215,217^ Adapted with permission from refs (215, 217). Copyright 2014, 2018 Wiley-VCH.

Mastalerz and co-workers used triptycene tetraol (**264**) and triboronic acid **265** to synthesize cage **266**. The self-assembly reaction involved 12 triptycene tetraol molecules **264** and eight triboronic acid molecules **265** via the formation of 24 boronic esters. The cage has minimum and maximum diameters of 2.6 and 3.1 nm, respectively. In solid-state, the material has a very high specific BET surface area of 3758 m^2^/g (N_2_, 77 K) (Figure 92 top).^215,216^ Using this strategy, the authors used triptycene hexaol **267** and *p*-benzenediboronic acid **268** derivatives to prepare cages **269**. The self-assembly reaction involves four triptycene hexaol molecules **267** and six *p*-benzenediboronic acid derivatives **268**. The obtained cages **269** are porous in the solid-state with a specific BET surface area of up to 511 m^2^/g (N_2_, 77 K) (Figure 92 bottom).^217^

**Figure 93:**
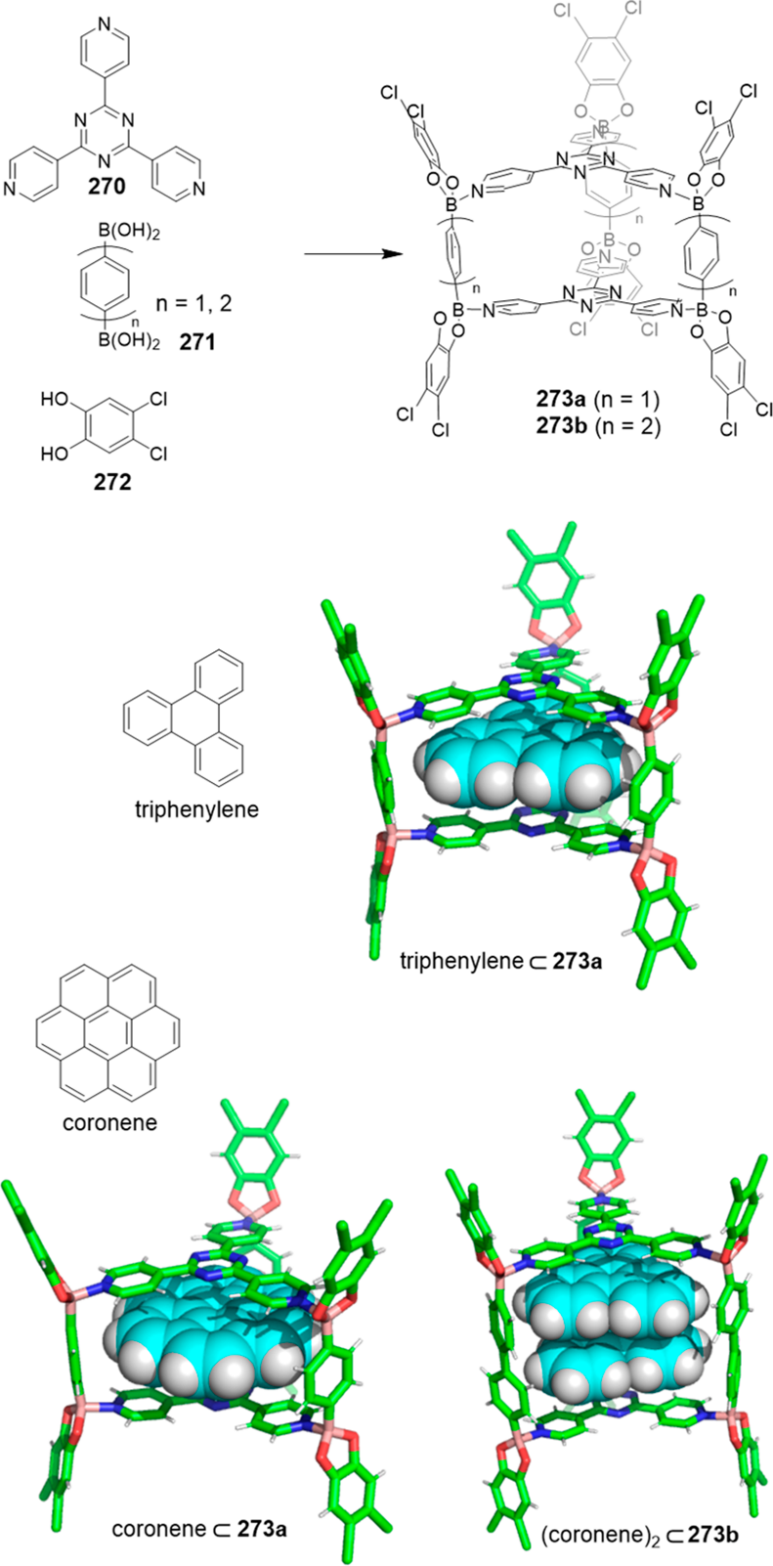
Multicomponent cages **273** and their inclusion complexes.^218^

Severin and co-workers reported the first example of cages containing boronate esters and dative B–N bonds. The multicomponent assembly of tripyridyl building blocks **270**, diboronic acid **271**, and catechol **272** yielded cages **273**. The obtained cage **273a** has a cavity size with a top–bottom wall distance of 6.8 Å, perfectly sized to encapsulate one molecule of coronene or one molecule of triphenylene (see the structures of the host–guest complexes in Figure 93). Extending the cavity by using 4,4′-biphenyldiboronic acid instead of *p*-benzenediboronic acid (top-bottom wall distance of 11.0 Å in cage **273b**) allowed the encapsulation of two triphenylene molecules. Despite these cages’ good synthetic availability, the authors point out that the low stability and limited solubility in organic solvents are significant limitations, although solid-state applications could be feasible (Figure 93).^218^

**Figure 94:**
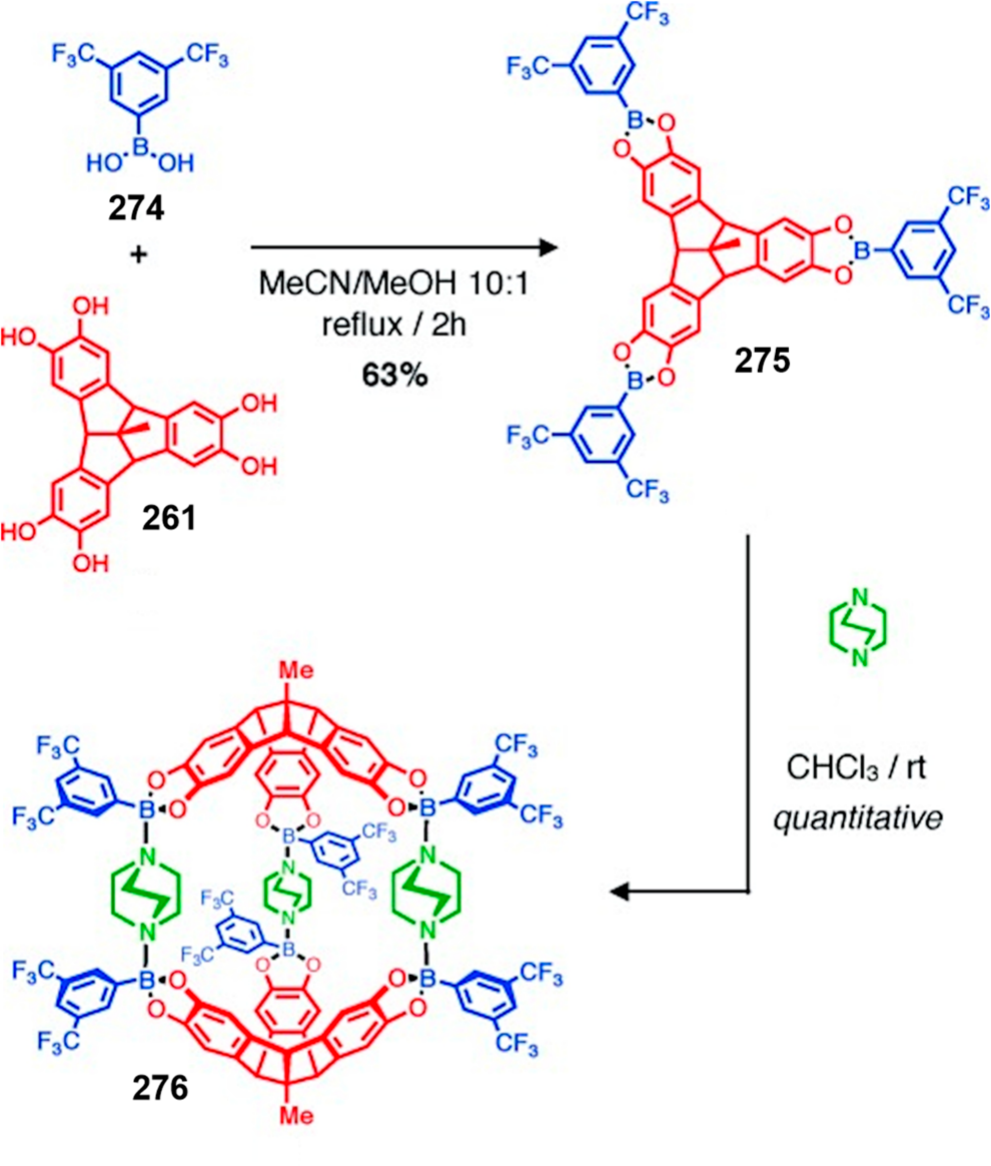
Synthesis of trisboronate ester **275** and self-assembly to form cage **276** by B–N dative bonds.^219^ Adapted with permission from ref (219). Copyright 2015 Wiley-VCH.

Beuerle and co-workers described another example of a supramolecular cage assembled through reversible boron–nitrogen dative bonds. To prepare the cage, an initial reaction of **261** and **274** yielded tribenzotriquinacene trisboronate ester **275.** The self-assembly reaction of **275** with 1,4-diazabicyclo[2.2.2]octane quantitatively yielded cage **276** in a concerted cooperative assembly pathway. Despite efficient cage formation, cage stability was limited. Thus, heating a solution containing the cage at a temperature around 360 K, produced cage disassembly. The disassembly was fully reversed by cooling down the mixture to room temperature. Besides, acidification produced irreversible cage decomposition (Figure 94).^219^

#### 3.2.5 Chiral

Chiral cages have drawn attention for having the potential to mimic the properties of enzyme-binding sites. As such, many cage structures include chiral building blocks, besides previous sections of this review also describing different examples of chiral cages, including **CC***n* cages, and cages containing: cyclohexanediamine (**74**, **107**, **113**, **114**, **172**–**174**, **176**, **189**, **191**, **193**, **194**), building blocks with *P*–*M* chirality (**163**, (**250**)_8_), and chiral amino acids (**228**, (**243**)_2_, (**245b**)_2_, (**247**)_2_, (**249**)_2_, and (**252**)_8_).

**Figure 95:**
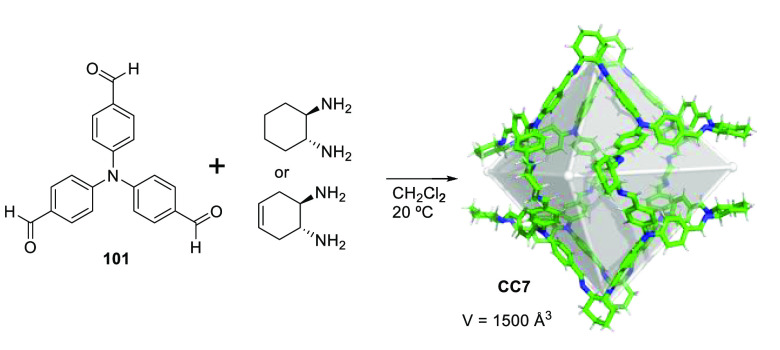
Synthesis of cages **CC7** ((*R*,*R*)-1,2-cyclohexanediamine) and **CC8** ((*R*,*R*)-1,2-cyclohex-4-enediamine). Both cages are isostructural and only cage **CC7** is shown in the figure.^221^

As stated above, a common method to introduce chirality into cage systems is to use chiral groups on the building blocks employed to assemble the cage structure. Following this approach, Cooper and co-workers reported numerous chiral cages prepared by imine condensation reactions of (*R*,*R*)-1,2-cyclohexanediamine and different aldehydes (see further examples in the gas separation in section 3.3.1 of this review). Cooper and co-workers prepared porous organic nanoparticles by means of chiral recognition between different organic cages. Cages were self-assembled in a modular fashion that can be predicted by molecular modeling calculations.^220^ The authors also prepared enantiopure cages **CC7** and **CC8** using tris(4-formylphenyl)amine **101** and chiral diamines (*R*,*R*)-1,2-cyclohexanediamine and (*R*,*R*)-1,2-cyclohex-4-enediamine. Both cages had inner diameters of 1.2 nm and a cavity volume of approximately 1500 Å^3^ (Figure 95).^221^

**Figure 96:**
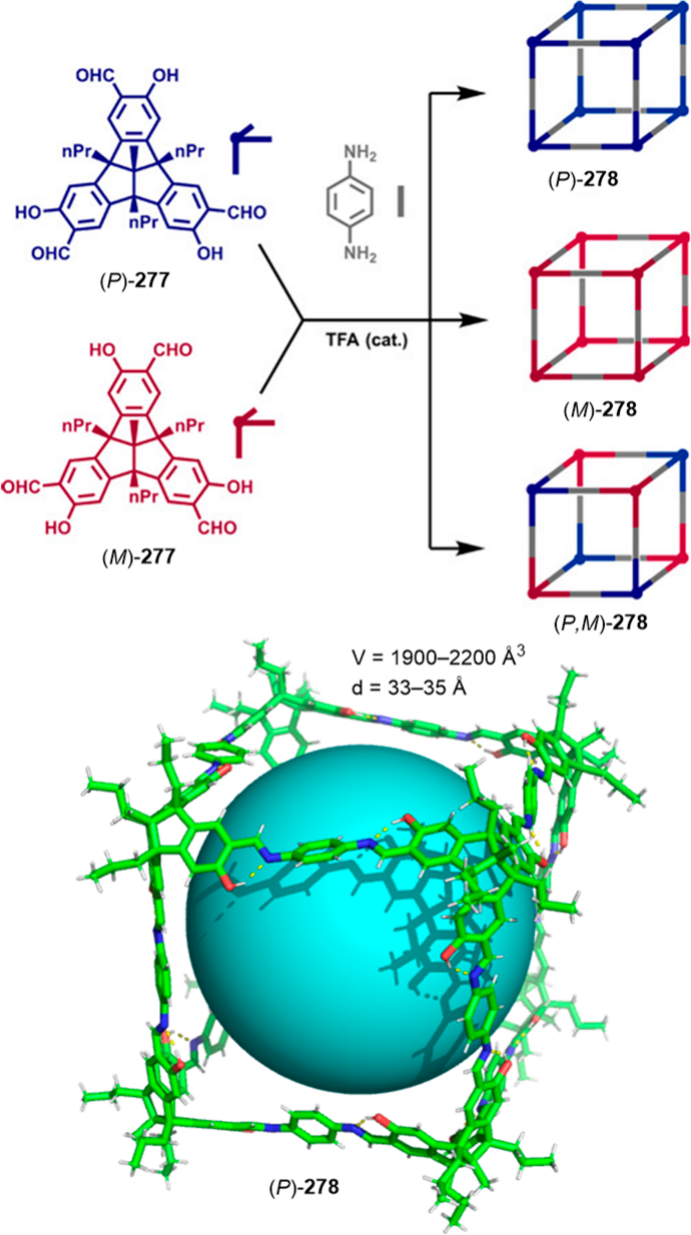
Reaction of *M* or *P* enantiopure or racemic tris(salicylaldehyde) **277** with *p*-phenylenediamine that leads to chiral cubic imine cages **278**. An example of the structure of enantiopure cage (*P*)-**278** is shown.^222^ Adapted with permission from ref (222). Copyright 2021 Wiley-VCH.

Mastalerz and co-workers employed chiral tris(salicylaldehyde) **277** to prepare the large cubic cage **278**. The reported synthesis involved the chiral self-sorting of eight chiral tris(salicylaldehyde) **277** and 12 *p*-phenylenediamine molecules. The cage structure has an internal diameter within the range 3.3–3.5 nm and constitute one of the largest reported organic cages. When the cage formation reaction was performed using racemic tris(salicylaldehyde) **278**, 23 cage isomers were possible, but only enantiopure and mesocage isomers were observed. This high selective chiral self-sorting toward the isomers with the highest degree of symmetry was the result of enthalpic and entropic factors. Despite the small energy differences of enantiopure and mesocage isomers, a careful selection of solvent allowed preferential meso cage formation, which can be achieved with an 83% yield. Gas adsorption measurements of the racemic cages in the solid state revealed a large BET specific surface areas of up to 1487 m^2^/g (N_2_, 77 K), which is one of the highest values obtained for organic cages (Figure 96).^222^

**Figure 97:**
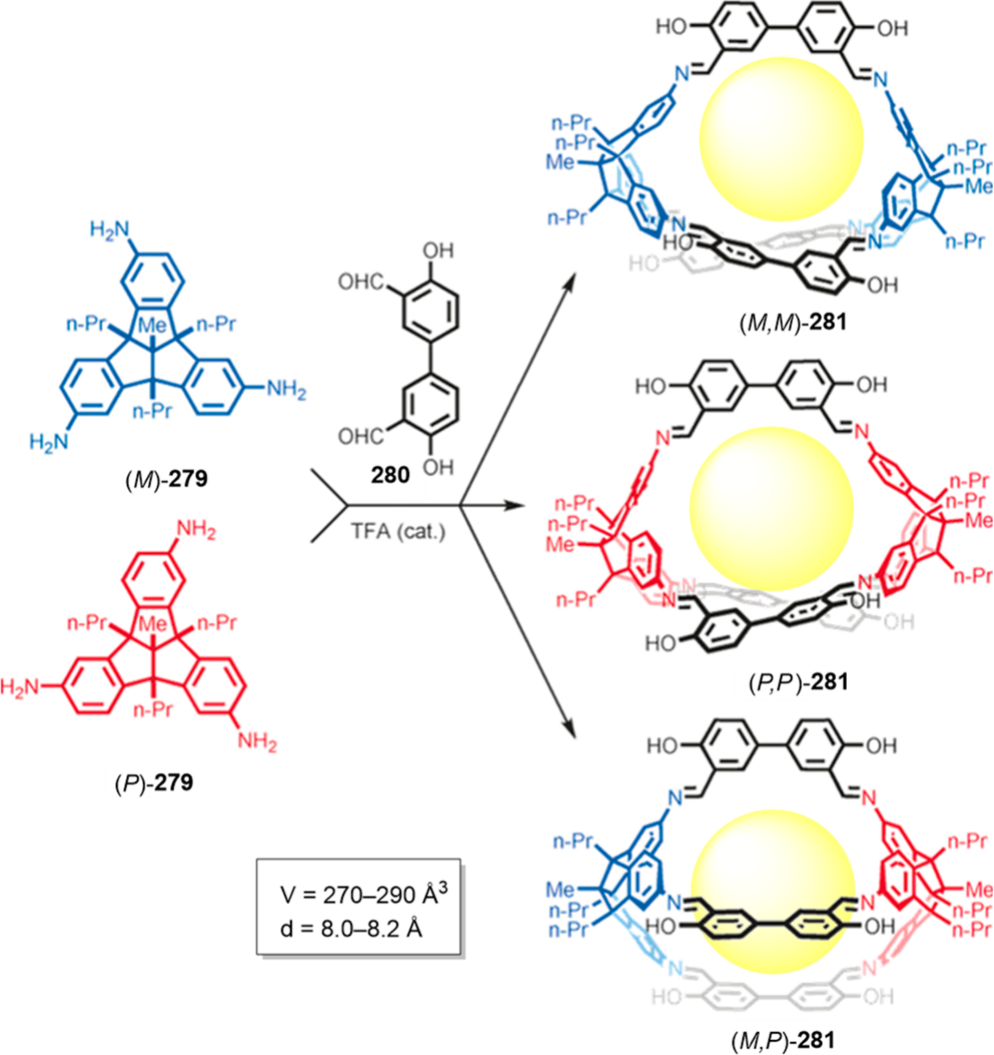
Cage **281** formation by condensation of a chiral or racemic triamine **279** with bis(salicylaldehyde).^223^ Adapted with permission from ref (223). Copyright 2017 Wiley-VCH.

Mastalerz and co-workers performed the condensation of triaminotribenzotriquinacene **279** with bis(salicylaldehyde) **280** to obtain three unstrained [2 + 3] cages (**281**) (Figure 97). The authors investigated if salicylimine cages **281** could be efficiently obtained from racemic building blocks by means of chiral self-sorting. An analysis of the reaction outcome with thermodynamic equilibrium evidenced narcissistic self-sorting that led to a racemic mixture of homochiral cage compounds (*M*,*M*)-**281** and (*P*,*P*)-**281** with the minor formation of heterochiral cage isomer (*M*,*P*)-**281**. These cages were in rapid equilibrium in the presence of TFA/D_2_O, which allowed an analysis by variable temperature ^1^H NMR in THF-*d*_8_ to estimate the enthalpy and entropy energies involved in the equilibrium. Enthalpy favored the formation of (*M*,*P*)-**281** over racemic-**281** (i.e., a racemic mixture of (*M*,*M*)-**281** and (*P*,*P*)-**281**) by 6.5 kJ/mol. In contrast, entropy favored the formation of racemic-**281** over (*M*,*P*)-**281** by 38 J/(K mol). The BET surface areas (N_2_, 77 K) were 918 m^2^/g for (*M*,*M*)-**281**, 550 m^2^/g for (*M*,*P*)-**281**, and 211 m^2^/g for racemic-**281**. Despite the large difference in surface areas, the calculated Henry’s selectivity, defined as the ratio between Henry’s constant of the strongly and weakly adsorbed components, were similar for all the compounds (CO_2_/CH_4_ = 3.75–4.93; CO_2_/N_2_ = 17.5–25.3; CH_4_/N_2_ = 4.32–5.52) (Figure 97).^223^

**Figure 98:**
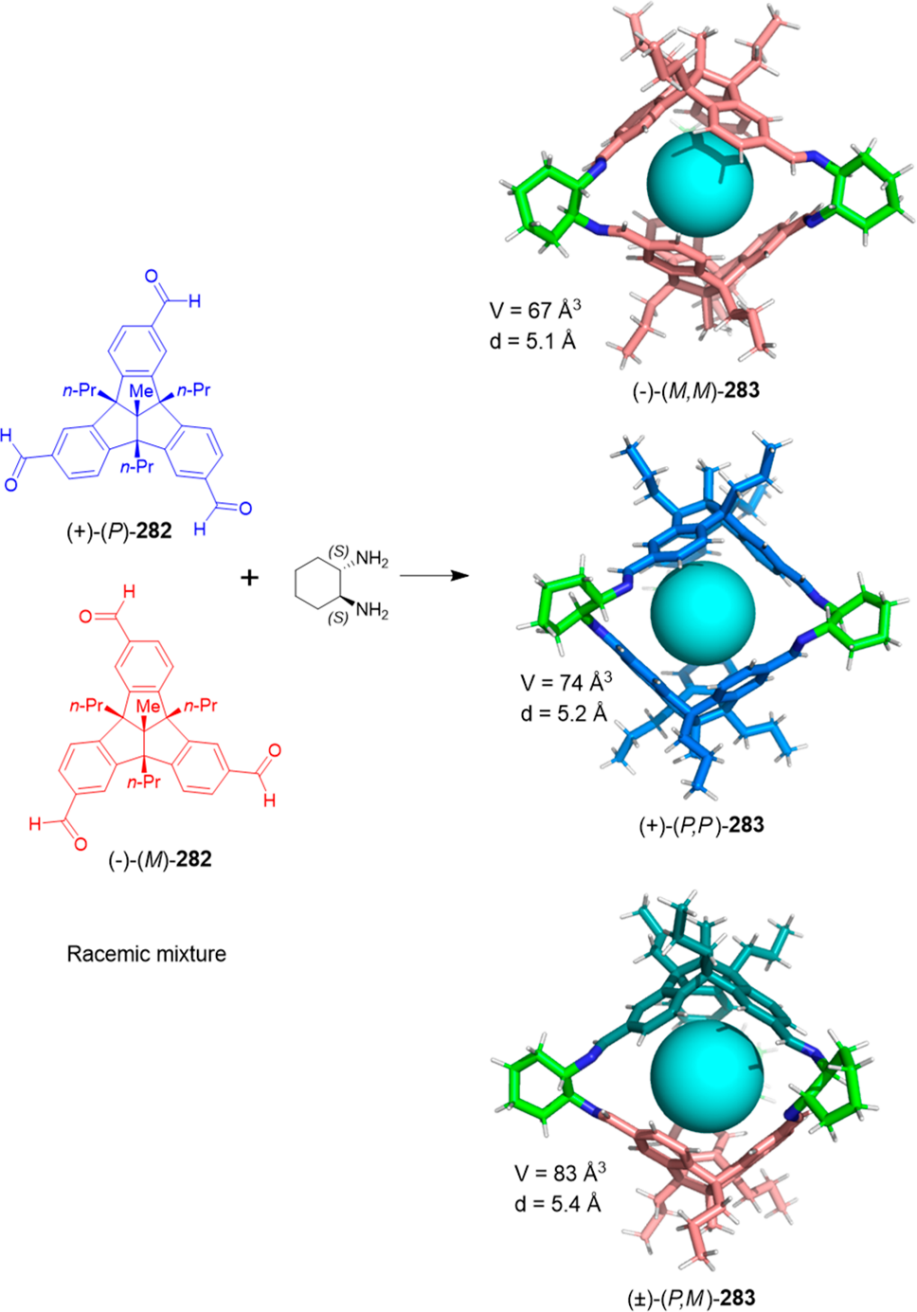
Synthesis of chiral organic cages **283** from precursor **282** and 1,2-cyclohexanediamine.^225,226^

Enantiopure hemicryptophanes are generally obtained by the chromatographic separation of diastereomers or by chiral HPLC resolution of racemic mixtures.^224^ Kuck, Chow, and co-workers developed a method using racemic tribenzobenzotriquinacene (TBTQ) (±)-**282** and enantiopure (1*S*,2*S*)-diaminocyclohexane to obtain a mixture of three diastereoisomeric imine cages (**283**) that can be isolated by silica gel chromatography. After isolation, the individual cryptophanes were hydrolyzed to yield the corresponding enantiopure trialdehydes (Figure 98).^225^ The authors also developed a method to obtain enantiomerically pure TBTQ (**282**) from diastereomeric TBTQ triamides using Boc-d- and Boc-l-phenylglycine as chiral auxiliaries. This building block was used to construct enantiomerically pure carbon cages.^226^

**Figure 99:**
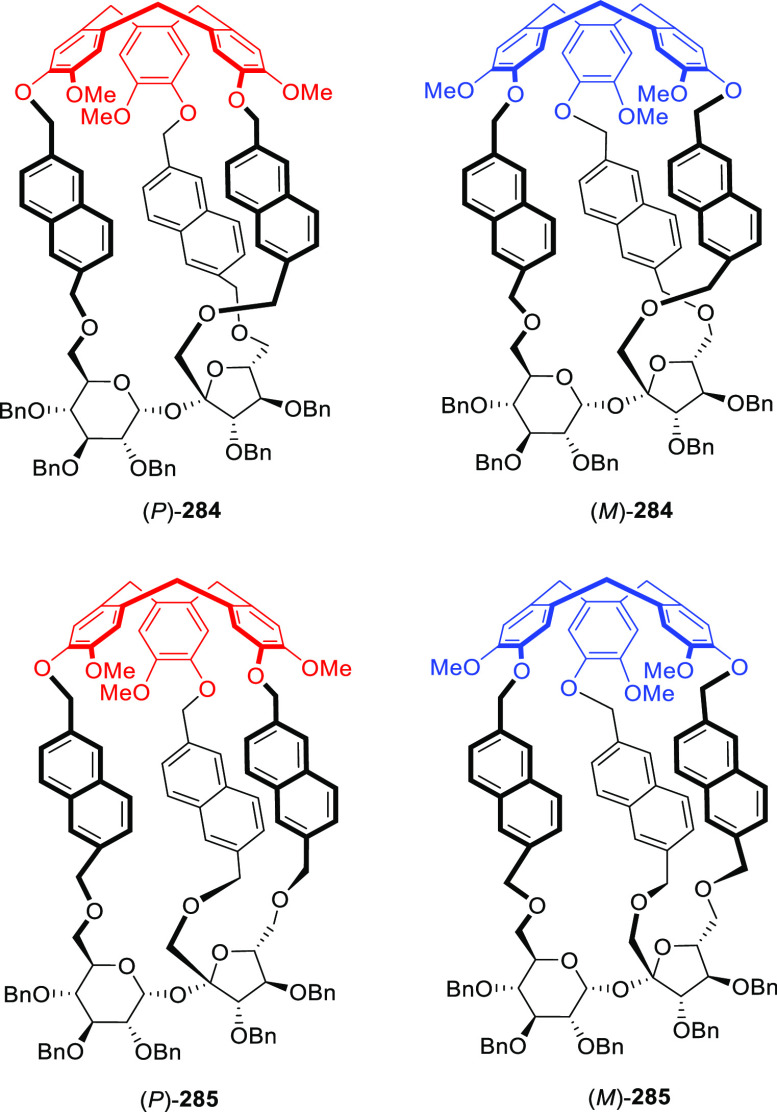
Fluorescent diastereoisomeric molecular cages **284** and **285** containing cyclotriveratrylene and sucrose.^227^

Jarosz, Szyszka, and co-workers reported the synthesis of fluorescent diastereoisomeric molecular cages **284** and **285** containing cyclotriveratrylene and sucrose. Cages are efficient receptors for acetylcholine and choline. Cages (*P*)-**285** and (*M*)-**285** have a similar affinity for choline than for acetylcholine, i.e., no selectivity is observed. In contrast, cage (*P*)-**284** has a better affinity for choline over acetylcholine, while cage (*M*)-**284** has a better affinity for acetylcholine over choline. The opposite selectivity seems to be associated with the complementarity of each guest, with the cavity size and shape of the chiral twisted ((*P*)-**285** and (*M*)-**285**) and non twisted ((*P*)-**284** and (*M*)-**284**) geometry of each diastereoisomer (Figure 99).^227^

**Figure 100:**
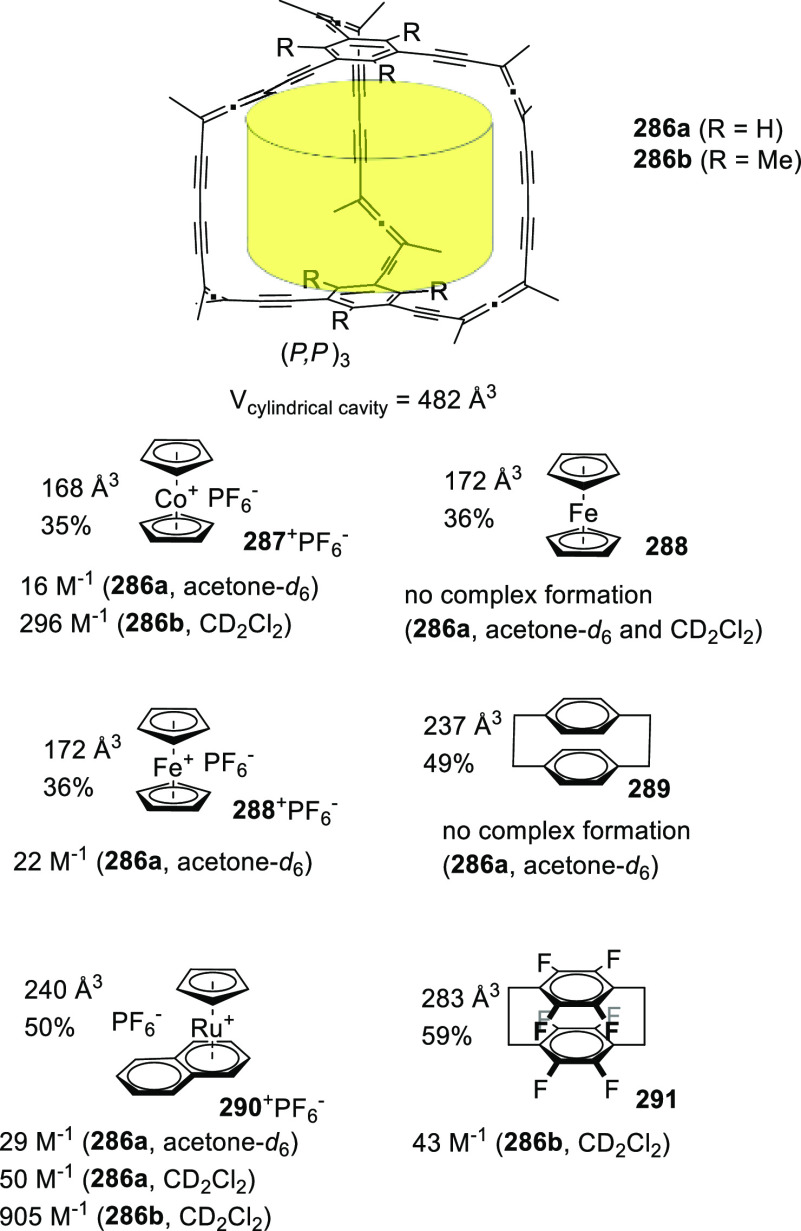
Helical prism-like cages **286** and guest structures. Guest volume, the corresponding cavity volume percentages of occupation and association constants are shown.^228,229^

Alonso-Gómez, Cid, and co-workers synthesized the two enantiomers of a helical prism-like cage (**286a**) to show 10-fold chiroptical amplification compared to its isolated building blocks. Initial studies have revealed that the cage could encapsulate an Fc^+^ molecule as determined by ^1^H NMR and HR-ESIMS, but no encapsulation was observed for Fc (Figure 100).^228^ To improve affinity toward electron-poor guests, the authors prepared a modified version of cage (**286b**) by introducing six methyl groups into phenyl rings to increase electron density and to also obtain a more defined cage cavity (Figure 100 bottom). They performed a series of host–guest studies, which were carried out with structurally similar guests to Fc^+^ (see structures **287**–**291** in Figure 100), with volumes within the 168–283 Å^3^ range, which fits into the cylindrical cage cavity of radius 4.3 Å, height 8.3 Å, and volume 482 Å^3^. The presence of methyl groups significantly enhanced binding affinities from, for example, 50 M^–1^ to 900 M^–1^ for **290**^**+**^ in CD_2_Cl_2_. An analysis of guest volumes and electronic properties revealed that the observed affinity was associated mainly with guest electronic properties and electron-poor guests had a better affinity.^229^

**Figure 101:**
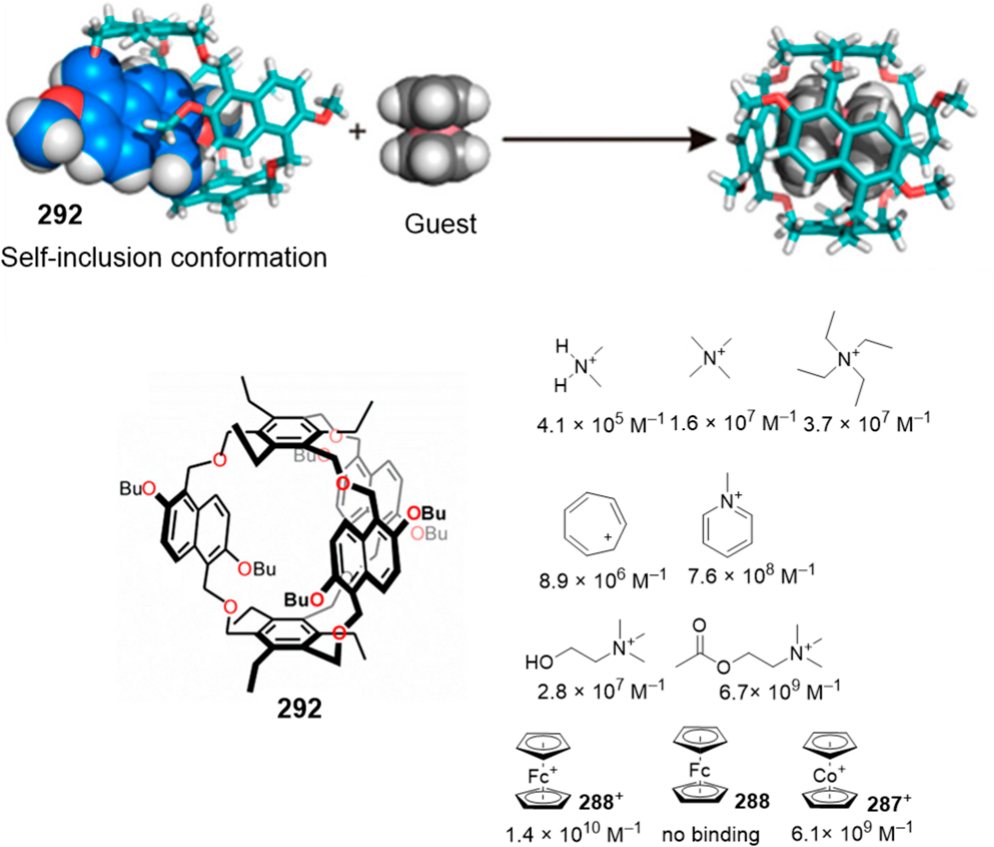
Self-inclusion conformation of naphthol-based cage **279** and encapsulation of the charged organic and organometallic cations.^230^ Adapted with permission from ref (230). Copyright 2019 American Chemical Society.

Jiang, Schalley, and co-workers reported the synthesis of naphthol-based cage **292** (naphthocage). The chiral cage, whose enantiomers were not separated in that study, is flexible and can adopt a self-inclusion conformation. Cage **292** has a high binding affinity to different monocharged organic cations, including methyl and ethylammonium salts, methylpyridinium, tropylium, ferrocenium (**288**^**+**^), and cobaltocenium (**287**^**+**^) (Figure 101) with *K*_assoc_ > 10^7^ M^–1^ in CD_2_Cl_2_/CD_3_CN 1:1. The extremely stable ferrocenium⊂naphthocage (**288**^+^⊂**292**) complex (*K*_assoc_ = 10^10^ M^–1^) can be electrochemically switched by reducing ferrocenium **288**^**+**^ to ferrocene **288** (Figure 101).^230^

**Figure 102:**
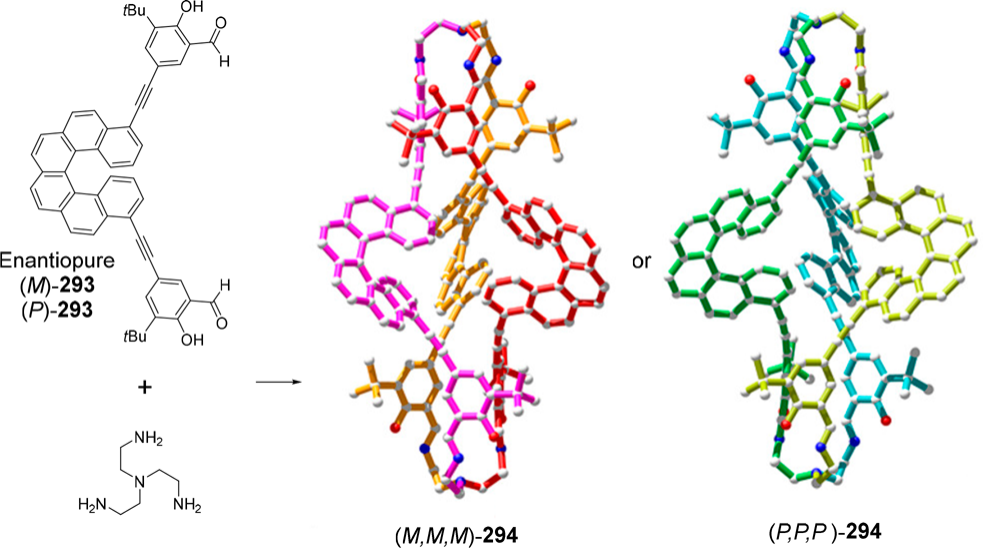
Cage **294** synthesis using helicene building blocks.^231^ Adapted with permission from ref (231). Copyright 2018 American Chemical Society.

Qiu, Shu, Shen, and co-workers reported employing enantiopure helicine building blocks **293** to prepare covalent organic cages (**294**) via imine condensation of enantiopure **293** and “tren”. The obtained cages had three helicene units aligned in a propeller shape to provide a triple-stranded helical architecture. This unique architecture was used for the enantioselective recognition of 1-phenylethylamine, 1-phenylethanol, and 1,1′-binaphthalene-2,2′-diol, which is promising for future asymmetric catalysis uses (Figure 102).^231^

### 3.3 Applications

Porous materials have unique properties for gas separation, gas sorption, catalysis, and other exceptional applications.^232^ Molecular cages also form part of the family of porous materials by forming solids with both intrinsic porosity (associated with cage cavity) and extrinsic porosity (associated with voids between individual cage molecules). Molecular organic cage applications involve mainly the encapsulation of guest species in their cavity. Some applications have already been described in the review for selected cages, and this section describes in more detail the use of organic cages to obtain porous materials for gas encapsulation and other applications.^233^ In particular, different representative examples that highlight materials’ porous nature based on organic cages are included in this section. Typically, these materials’ porosity is obtained by measuring the BET surface area using N_2_ at 77 K. Most examples also include the uptake of CO_2_ measurements, and determination of selectivity for, e.g., CO_2_/methane. Note that we have also included information on porosity in some examples described in previous sections of this review because this parameter is related to cage rigidity/flexibility and is, therefore, useful in cage designs.

#### 3.3.1 Encapsulation of Gases

**Figure 103:**
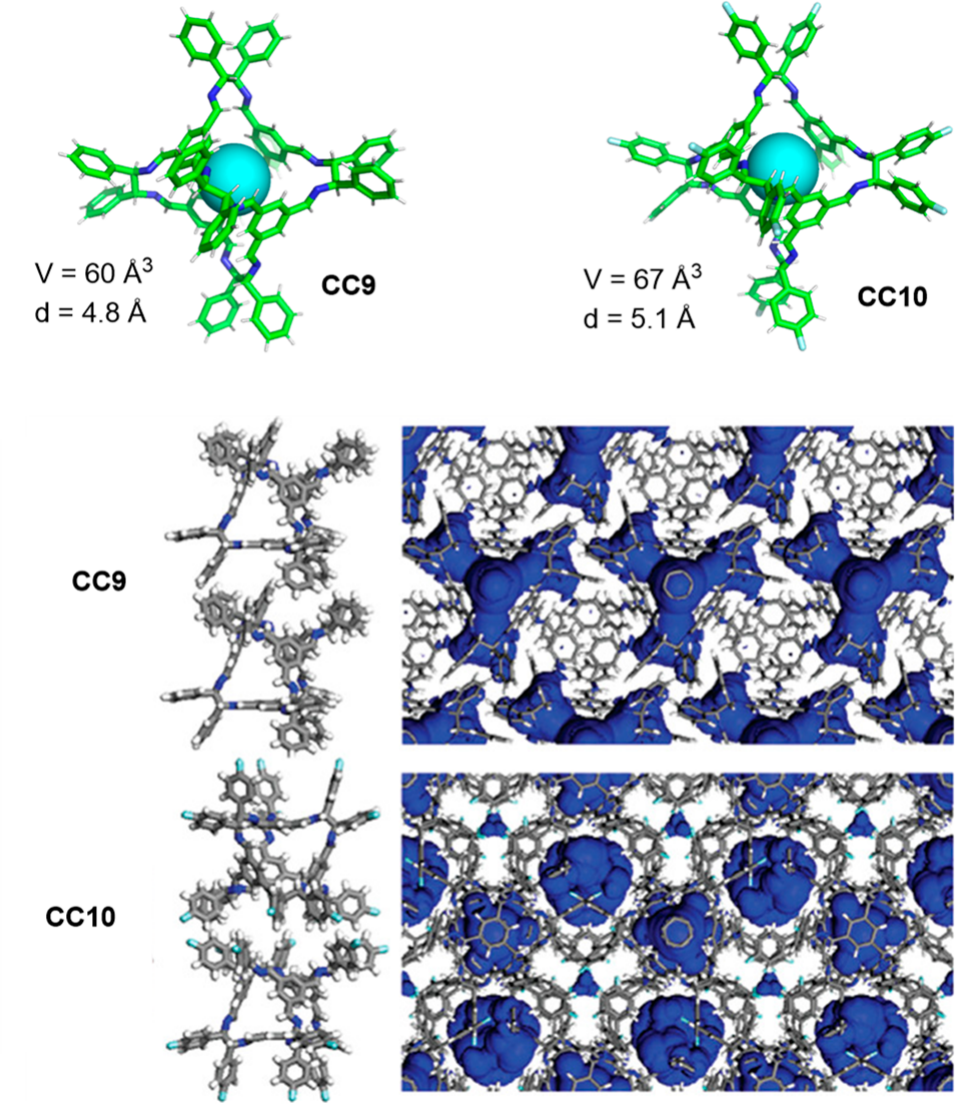
Porous cages (**CC9** and **CC10**) with extrinsic porosity. Surface plots (probe radius of 1.82 Å) indicating pore connectivity for cages **CC9** (R3 polymorph) and **CC10**. Both extrinsic and intrinsic pores are presented in blue.^236^ See Figure 30 for further examples of **CC***n* cages. Adapted with permission from (236). Copyright 2011 American Chemical Society.

Separating similar sized and shaped molecules is a challenging task that usually involves cryogenic methods. As an alternative, materials based on the properties of cage cavities can be used for this purpose with excellent performance. To finely tune porous materials’ properties, it is necessary to control pore size, shape, and connectivity.^234^ Increasing cage cavity volumes and cage structure rigidity allows porous materials to be obtained whose surface areas approach 4000 m^2^/g.^235^ A material’s overall porosity includes the intrinsic porosity from the cage cavity and the extrinsic porosity between cage molecules. Several methods have been followed to increase porosity in cage materials. By way of example, by means of the bulky directing groups in **CC9** and **CC10**, it is possible to hinder crystal packing from generating voids between cage molecules and to, hence, generate extrinsic porosity that is affected by cage packing in the solid state. Solid materials formed by cages **CC9** and **CC10** show solid-state packing differences and, therefore, differences in both extrinsic porosity and pore connectivity due to heteroatomic and aromatic intermolecular interactions of the fluorine atoms in **CC10** (Figure 103).^236^ Additionally, the flexibility of cage structures strongly impacts adsorption properties, which contributes to porosity into three different classes: static porosity, dynamic porosity, and cooperative porosity. Static porosity is associated with the connected voids in solid material. Dynamic porosity is related to the flexibility of the connected void network when voids are empty. Cooperative porosity is associated when it is required to place guests in the cavity to transport guests along the void network because voids are disconnected in the empty host.^237^

**Figure 104:**
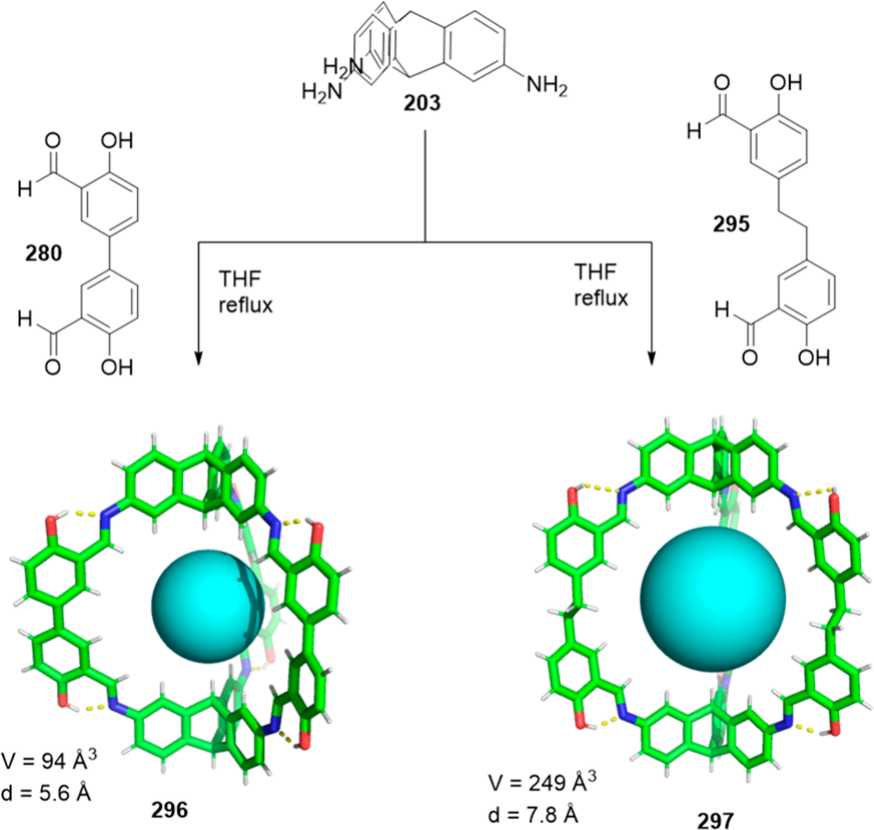
Cages with different flexibility, rigid cage **296**, and flexible cage **297**.^238^

Cage rigidity is a key factor to obtain porous materials in the solid state. Mastalerz and co-workers studied the effect of building blocks’ rigidity on the formation of [2 + 3] cages and the effect on gas sorption properties in the solid state. For this purpose, the authors used triptycene triamine **203** and two different bis-salicylaldehydes (**280** and **295**) to prepare the [2 + 3] cages **296** and **297**. Rigidity directly impacted cage formation, and using more rigid aldehyde (i.e., **280**) formed a cage with a 69% yield, whereas more flexible aldehyde (i.e., **295**) produced a cage with a 33% yield, presumably because flexibility allowed more oligomeric and polymeric byproducts to be obtained. Significant differences were also found in solid-state porosity because the cage with the more flexible linker underwent a phase change during nitrogen gas evacuation after gas sorption that yielded permanent low porous material. So whereas rigid cage **296** had a BET surface area of 744 m^2^/g (N_2_, 77 K), the more flexible cage **297** had one of only 30 m^2^/g, which indicates that pores were very small and the adsorption was kinetically hindered. Cages **296** and **297** showed a good CO_2_ uptake at 298 K by adsorbing 2.7 and 2.3 mmol/g, respectively. Additionally, good CO_2_/methane selectivity was achieved; i.e., 4 for cage **296** and 10 for cage **297** (Figure 104).^238^

**Figure 105:**
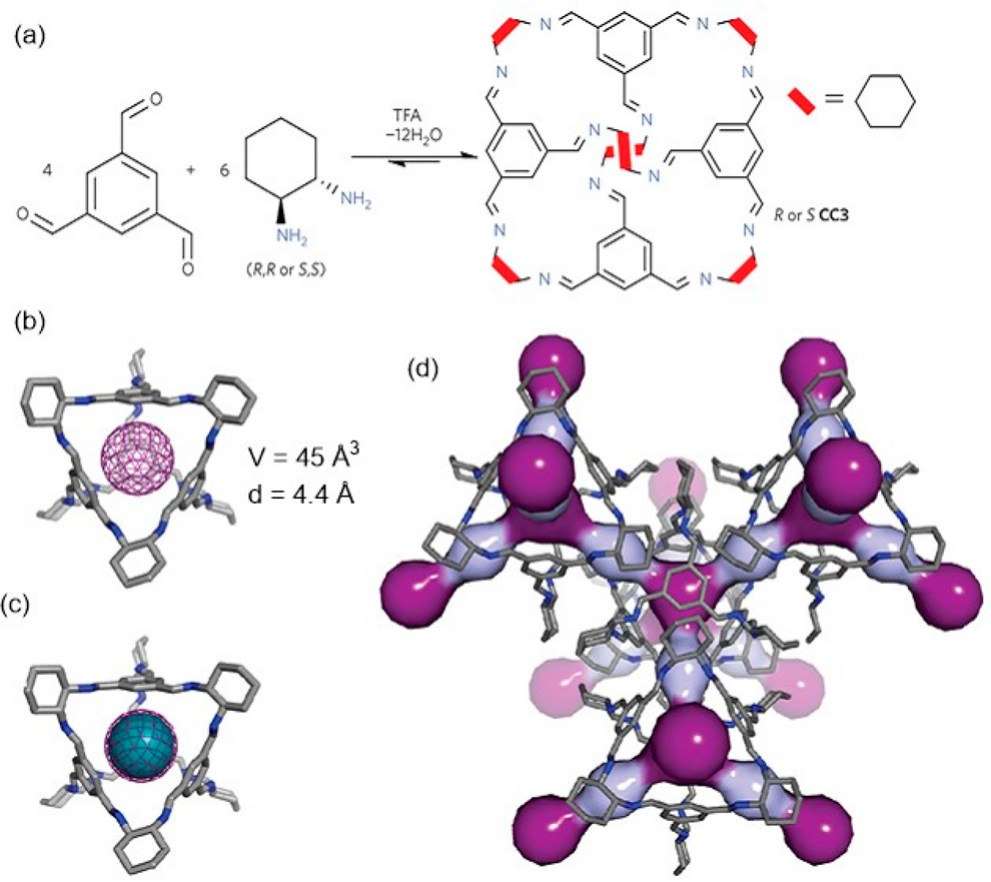
(a) One-pot synthesis of cage **CC3**. (b) The largest inclusion sphere inside the cage (dark purple mesh). (c) Xenon atom (cyan sphere) in the cage cavity. Radon atom also fits perfectly (not shown). (d) Two cavity types in the solid structure: a cage cavity inside the cage itself (dark purple) and a cage windows between adjacent cage windows (light purple).^239^ Reproduced with permission from ref (239). Copyright 2014 Springer Nature.

Cooper and co-workers used organic cage **CC3** for the solid-state separation of rare gases with unprecedented performance.^57^ This cage had a cavity with a diameter of 4.4 Å that came very close to the diameters of xenon (4.10 Å) and radon (4.17 Å). From the static view of the 3D structure in the solid state, the narrowest point in pore channels was placed at the cage windows between adjacent cages, with a diameter of only 3.6 Å. This diameter was slightly smaller than the diameter of Kr (3.69 Å) and was, in theory, too narrow to permit the diffusion of either xenon or radon. However, the cage structure vibrations associated with cage flexibility led to an enlarged pore diameter for a small fraction of time. This phenomenon was quantified by molecular dynamics simulations and showed a time-averaged pore-limiting envelope that was broad enough to allow the diffusion of both xenon and radon as experimentally observed. In fact, experimental gas adsorption isotherms demonstrated the substantial selective uptake of both krypton and xenon in **CC3**. Promising results for chiral separations were observed for the homochiral **CC3**-*R* and **CC3**-*S* crystals with the preferential adsorption of the 1-phenylethanol enantiomer with the opposite chirality, which suggests future applications in enantioselective separations (Figure 105).^239^

One key aspect for determining solid materials’ properties (e.g., guest uptake kinetics) is surface morphology. To understand the properties of the surface of these porous solids, Cooper and co-workers took AFM measurements, which allowed the surface in crystals of cage **CC3**-*R* to be studied. The results showed no distortion from the expected surfaces from their single-crystal X-ray structure, which proves that the surface does not relax or deviate from bulk crystal packing.^240^

In addition, the solid-state structure can be altered by changing the solvent because it modifies the crystal packing preferences of cages,^241^ and also by the interconversion in the solid state by the response to specific chemical triggers,^242^ which are associated with cage flexibility and the presence of different conformers in the solid state.^243^

**Figure 106:**
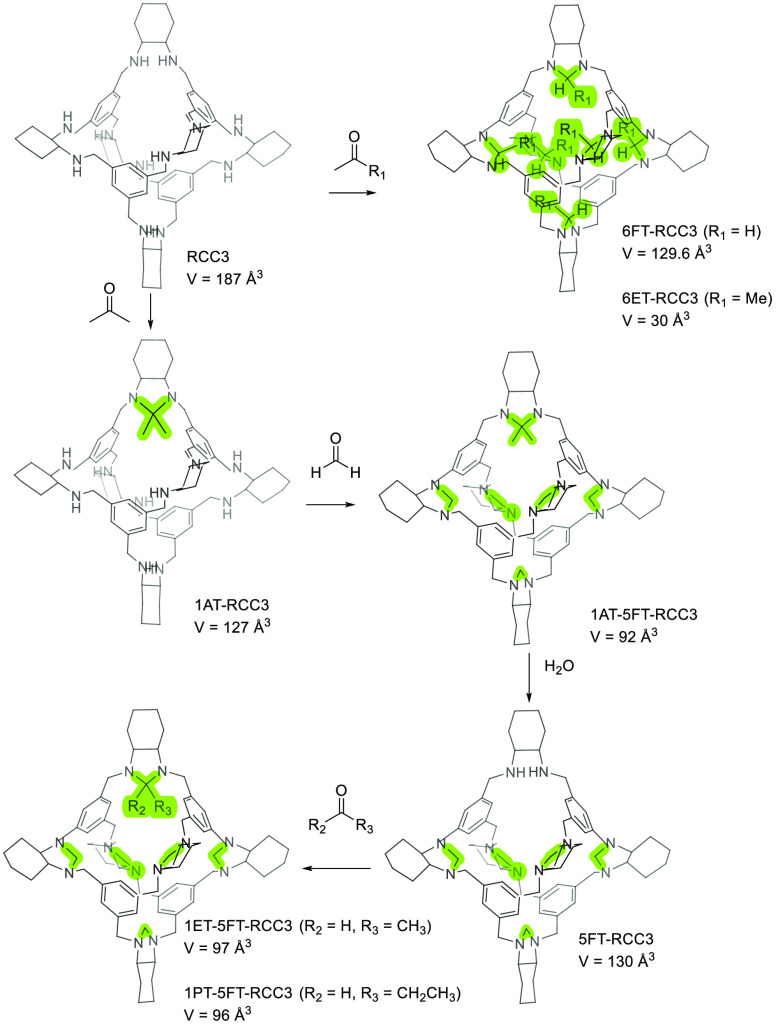
Cages deriving from cage **CC3** with modified internal cavities.^244^

Cooper and co-workers modified cage **CC3**’s internal cavity by performing a series of organic reactions to tune its size (Figure 106). This method obtained hybrid materials capable of separating deuterium and hydrogen by kinetic quantum sieving (KQS). This methodology involves a series of protect–functionalize–deprotect that allows to tweak cavity size from 187 Å^3^ of **CC3** to 30 Å^3^ of 6ET-**RCC3**. The fine-tuning of cavity size gave a very small difference between the volumes of the hydrogen molecule and the cavity. In fact the minimum molecular dimension of H_2_ was 2.2 Å and cage 6ET-**RCC3** had a pore-limiting envelope centered at 1.95 Å and broad time-averaged size distribution, which is ideal for selective gas separation. In fact, the cage 6ET-**RCC3** allows the separation of D_2_ and H_2_ with a selectivity of 8.0 with a high D_2_ uptake of 4.7 mmol/g.^244^ The authors also demonstrated that reversible SO_2_ capture can be also achieved using cage **CC3** derivatives. Whereas cage **CC3** displayed modest and reversible SO_2_ capture, the secondary amine **RCC3** cage exhibited high irreversible SO_2_ capture, and tertiary amine 6FT-**RCC3** showed an excellent and very high reversible SO_2_ capture (13.78 mmol/g; 16.4 SO_2_ molecules per cage). The high uptake at low partial pressures revealed the potential of cage 6FT-**RCC3** for trace SO_2_ adsorption.^245^

**Figure 107:**
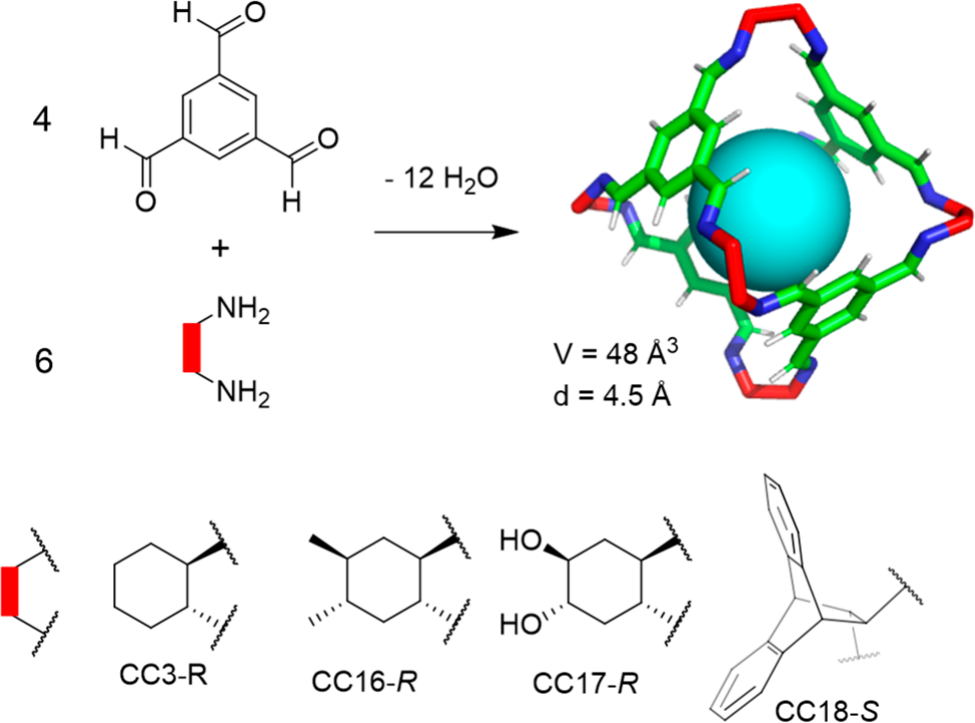
Synthesis of the **CC3** cage derivatives.^246^

Cooper, Briggs, and co-workers synthesized derivatives of *trans*-1,2-diamino-cyclohexane to prepare **CC3** cage analogues. The introduction of two methyl groups into this building block resulted in cage **CC16**, which had frustrated packing with increased porosity compared to parent cage **CC3**. The BET surface area (N_2_, 77K) for these cages was 1023 m^2^/g (**CC16**) and 409 m^2^/g (**CC3**). The building block with two hydroxyl groups (cage **CC17**-*R*) maintained the porosity of parent cage **CC3** with a BET surface area (N_2_, 77K) of 423 m^2^/g. In contrast, the incorporation of bulky dihydroethanoanthracene groups changed the self-assembly toward the formation of a larger non porous [8 + 12] cage **CC18**-*S* (BET surface area (N_2_, 77K) of 10 m^2^/g) (Figure 107).^246^ Scrambling reactions of cages containing 1,2-ethylenediamine (**CC1**) and *R*,*R*-1,2-cyclohexanediamine (**CC3**) lead to a distribution of molecules with different shapes that cannot effectively pack, which creates a on-crystalline porous amorphous solid. Changing the scrambling reaction ratio allows the fine-tuning of porous properties by allowing materials with high H_2_/N_2_ selectivity to be obtained. In some cases, H_2_/N_2_ selectivity is as low as 1–1.5 (mol/mol). By simply varying the composition of the scrambling reaction, it is possible to increase selectivity up to 5 as a result of the poor packing of the scrambled cage mixture.^247^

**Figure 108:**
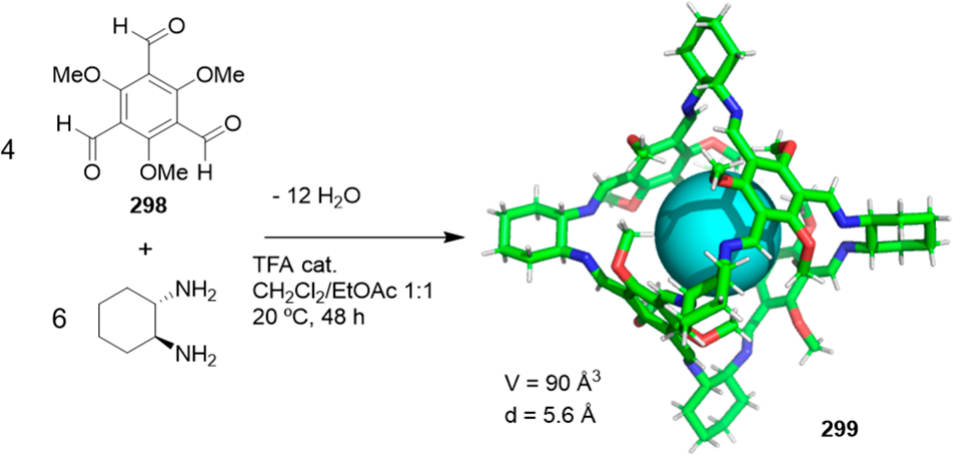
Synthesis of stable **CC3** cage derivative **299**.^248^

Banerjee and co-workers prepared a **CC3** cage analogue using 2,4,6-trimethoxy-1,3,5-triformyl benzene **298** and 1,2-cyclohexanediamine as building blocks to form the imine-based cage **299** (Figure 108). In the cage structure, the OMe groups built significant steric hindrance and hydrophobicity around imine bonds and protected them from acid or basic hydrolysis, which resulted in enhanced cage stability. Additionally, the electronic effects of the OMe groups introduced into the cage aromatic rings resulted in electron-rich aromatic rings, which also improve cage stability. Cage **299** presents three different solid-state polymorphs, which are selectively obtained using different solvents. Of the three polymorphs, only one is porous to nitrogen. The porous polymorph (β) presents a window-to-window arrangement that results in a porous structure with a BET surface area (N_2_) of 370 m^2^/g (Figure 108).^248^

**Figure 109:**
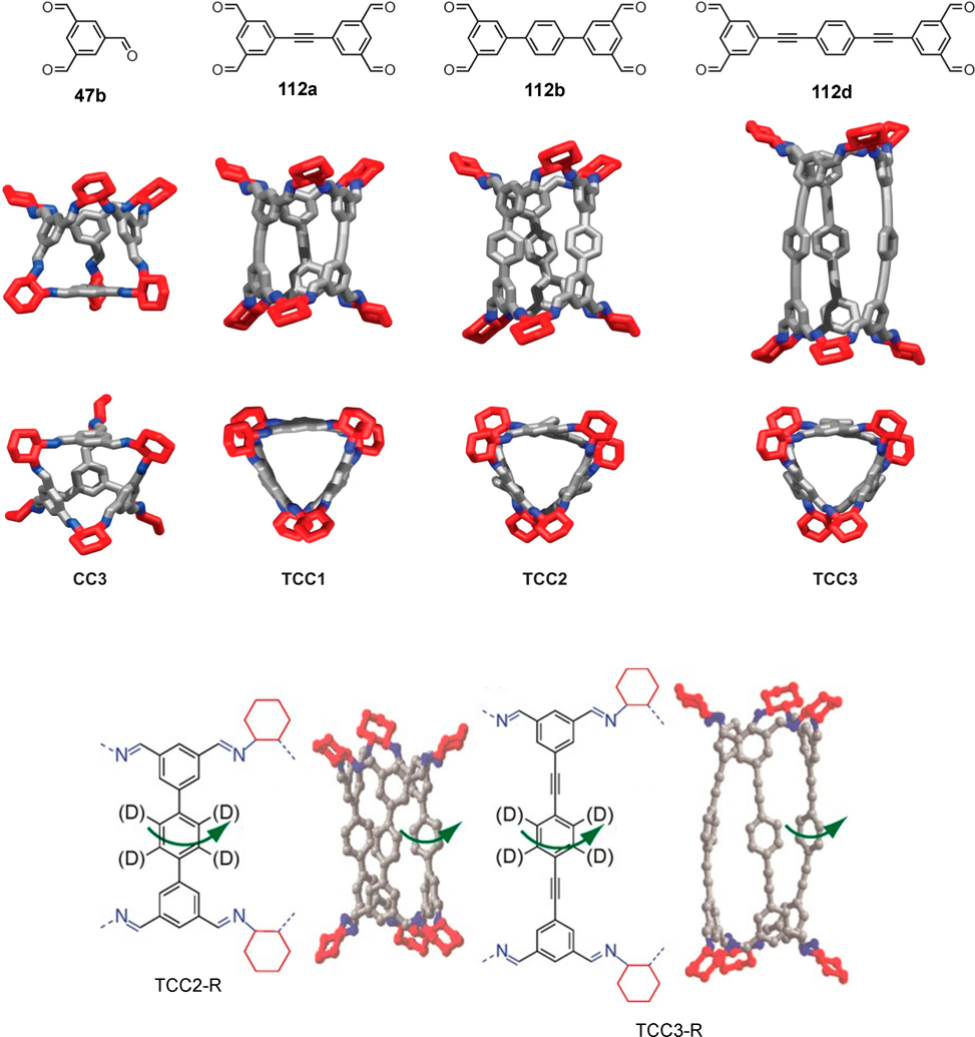
Structure of the tetraaldehyde building blocks used for the synthesis of **TCC** and the trialdehyde building block used for **CC3**.^249,250^ Adapted with permission from ref (249). Copyright 2017 Springer Nature. Adapted with permission from ref (250). Copyright 2017 Wiley-VCH.

Cooper, Day, and co-workers used 1,2-cyclohexanediamine and tetraaldehyde building blocks (**112a**, **112b**, and **112c**) to obtain cage structures with a tubular geometry (**TCC** cages, Figure 109). At the ends of a tube, **TCC** cages had two roughly triangular windows that resembled the windows of cage **CC3**. However, window-to-window packing was observed between the homochiral **TCCs** unlike in cages **CC3**. In contrast, racemic **TCC** mixtures form 1D nanotubes structures in the solid state with a window-to-window arrangement of the cages along cages’ tube direction. The materials’ porosity depends on each cage structure, and the BET surface areas (N_2_, 77 K) for **TCC1**-*R*/**TCC1**-*S* are 881 m^2^/g and 1022 m^2^/g for **TCC3**-*R*/**TCC3**-*S*. In contrast, **TCC2**-*R*/**TCC2**-*S* does not display any microporosity and exhibits a BET surface of only 26 m^2^/g. The cocrystals of **TCC2**-*R* with **CC3**-*S* at the 1:2 molar ratio allowed a porous crystal structure to be obtained that was formed by the **CC3**-*S* layers pillared by **CC3**-S/**TCC2** through window-to-window interactions. The obtained material had a BET surface area (N_2_, 77 K) of 1363 m^2^/g, which is bigger than the individual components.^249^ In **TCC** systems, the ^2^H solid-state echo NMR experiments were performed to determine the ^13^C T_1_ values to study the rotational dynamics of the *para*-phenylene rings that constitute the central cavity walls. The rotational rates fell within the 1.2–8 × 10^6^ Hz range at 230 K with low activation energy barriers within the range 12–18 kJ/mol. The rotation rate significantly lowered to 5–10 × 10^4^ Hz at 230 K by the encapsulation of iodine molecules. The authors pointed out that these results highlight the importance of the dynamics of cage systems in contrast to a simple analysis of static structures that can provide incomplete information. In light of this, studying cage dynamics could be important in competitive guest loading, molecular separation, and guest release (Figure 109).^250^

**Figure 110:**
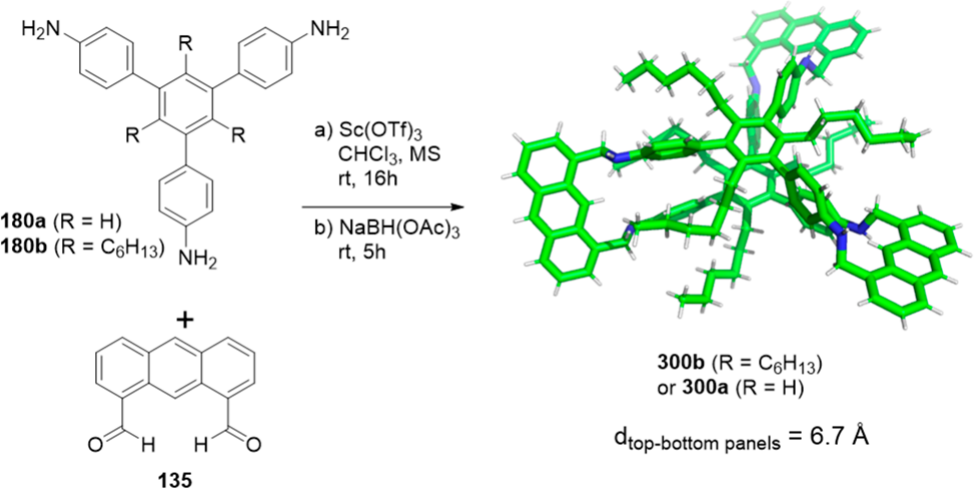
Cage **300** synthesis from a triamine (**180**) and a dialdehyde (135). MS = molecular sieves, Tf = trifluoromethanesulfonyl. Distance between the top and bottom panels obtained from molecular modeling.^251^

Zhang and co-workers prepared shape-persistent organic molecular cage **300** by the reaction of **135** and **180**. Cage **300** in the solid state displayed completely reversible adsorption of CO_2_ and N_2_ with a selectivity of 73 for CO_2_ adsorption over N_2_ (at standard temperature and pressure (STP) of 20 °C, and 1 bar). The uptake value of CO_2_ was 4.46 cm^3^/g (amount of gas at STP), whereas N_2_ was practically not adsorbed at all (0.061 cm^3^/g at STP). The observed gas-adsorption selectivity can be ascribed to the formation of reversible carbamate bonds by CO_2_ interacting with the cage secondary amine groups, which are favored by the well-defined porous cage cavity structure (Figure 110).^251^

**Figure 111:**
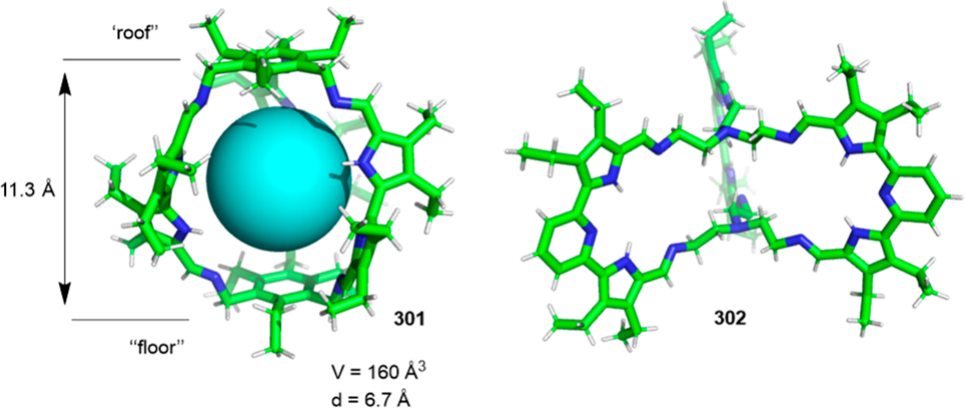
Pyrrole-based organic cages **301** and **302** for selective CO_2_ gas adsorption.^252^

Sessler, Humphrey, Zhang, and co-workers prepared pyrrole-based organic cages **301** and **302** with an affinity toward CO_2_ gas associated with the polar cavity that provided pyrrole subunits. Cage **301** had a well-defined central cavity with a “floor” to “roof” distance of 11.3 Å. In contrast, cage **302** had a much less defined cavity. The gas sorption experiments using CO_2_, N_2_, H_2_, O_2_, and CH_4_ showed that both cages were selective toward CO_2_. The BET surface areas for CO_2_ were 279 m^2^/g for cage **302** and 111 m^2^/g for cage **301** (Figure 111).^252^

**Figure 112:**
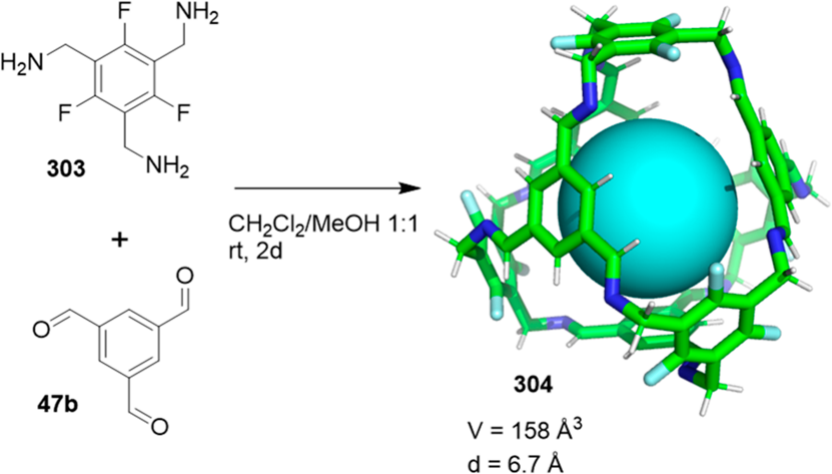
Synthesis of porous fluorinated imine cage **304** with a pore volume of 158 Å^3^.^253^

Schmidt and co-workers used trialdehyde **47b** and triamine **303** to synthesize the first porous organic cage containing perfluorinated aromatic rings **304** (Figure 112). Solid-state packing from X-ray - crystal diffraction studies showed window-to-window channels connecting cages **304**, which gave a BET surface area of 536 m^2^/g. The fluorinated aromatic ring increased CO_2_-philicity by allowing 19.0 wt % of CO_2_ to be captured (4.2 mmol/g, 273 K, 1 bar), which is higher than similar sized cages **CC2** (13.2 wt % CO_2_) and **CC3** (11.0 wt % CO_2_). The authors describe how the observed higher gas uptake is likely to be associated with the higher hydrophobicity of the cavity caused by fluorinated aromatic rings.^253^

**Figure 113:**
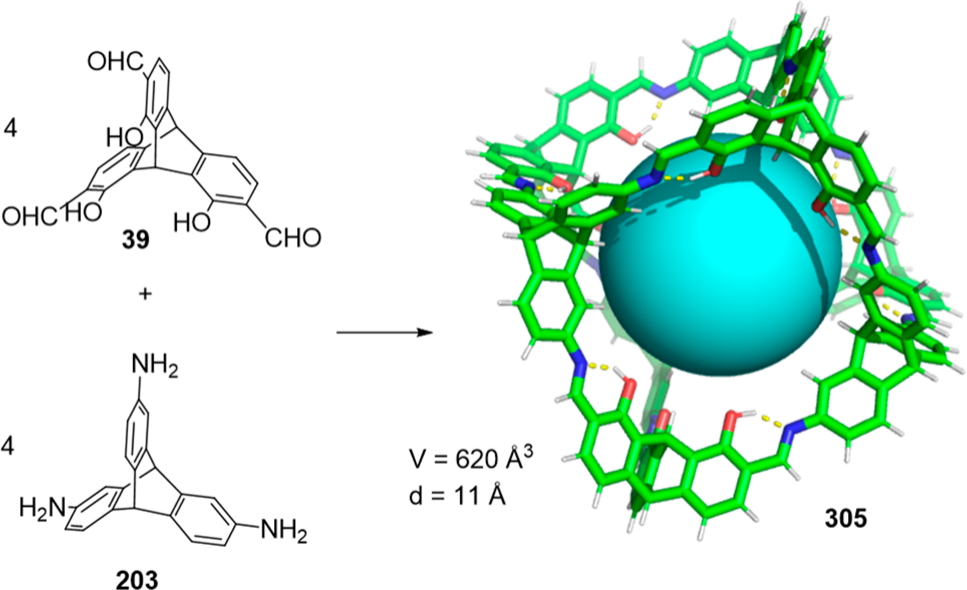
Synthesis of the rigid [4 + 4] cube **305**.^254^

Mastalerz and co-workers used trialdehyde **39** and triamine **203** to synthesize the [4 + 4] triptycene-based porous cube **305** with a large BET surface area of 1014 m^2^/g (N_2_, 77 K), high CO_2_ uptake (18.2 wt % at 273 K and 1 bar), and CO_2_/N_2_ selectivity of 33.7 (Figure 113).^254^

**Figure 114:**
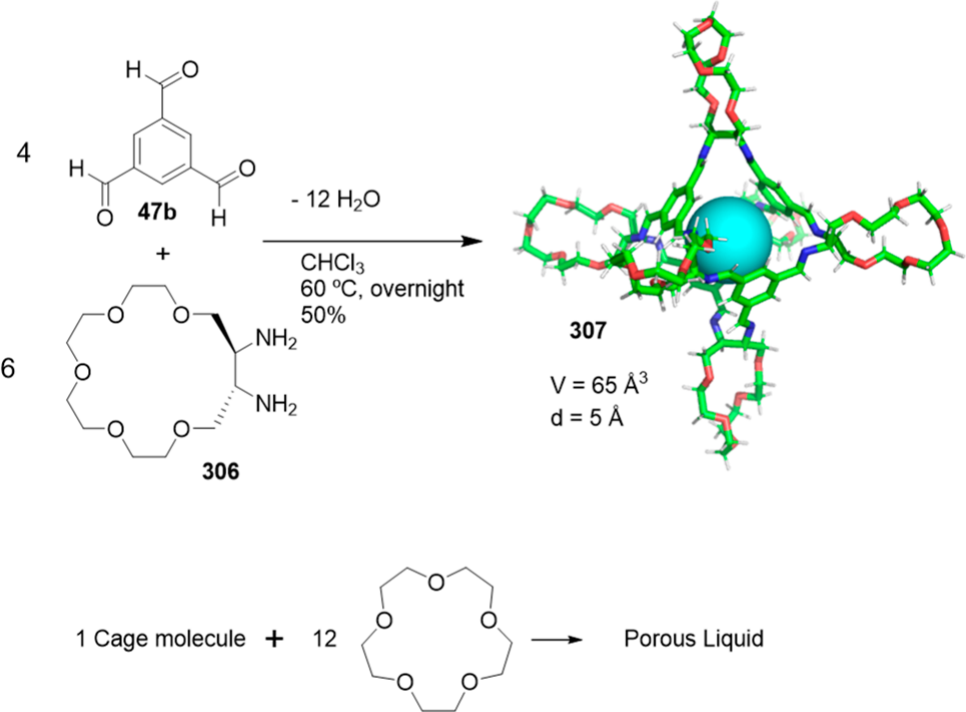
Synthesis of the porous liquid containing cage **307**.^258^

The concept of liquids with permanent porosity was theoretically proposed by James and co-workers in 2007 by defining three different types of porous liquids: type I (neat porous liquids), type II (porous hosts dissolved in sterically hindered solvents), and type III (microporous framework materials dispersed in hindered solvents).^255−257^ Later in 2015, James, Copper, and co-workers used trialdehyde **47b** and diamine **306** to prepare porous liquids (type II) based on organic cages **307**, which is the first reported synthesis of this type of materials (Figure 114).^258^ For this purpose, they used the core structure of cage **CC3**, which has a cage pore diameter of 5 Å and windows of 4 Å diameter. They anchored a crown-ether in diamine building block **306** to provide the required solubility in the cavity-excluded solvents (Figure 114). To obtain the liquid cage phase, a crown ether was chosen as the solvent (15-crown-5) because it did not fit into cage pores and, therefore, cage pores remained empty. Using this solvent, it is possible to obtain a concentrated solution of the cage (44 wt %) that corresponds to only 12 solvent molecules per cage molecule. Different sets of experiments proved that the cage cavity was empty in this liquid, although the porous liquid was able to host gas molecules, such as N_2_, CH_4_, CO_2_, or Xe. Qiao and co-workers performed molecular simulations and determined that the intrinsic gas storage capacity of cage molecules followed the order of CH_4_ > CO_2_ > N_2_ and determined that each molecule had a preference for a different placement in cage cavity. CO_2_ is preferentially positioned in the center of the cavity, CH_4_ is located in both central and branched cavity regions, and N_2_ molecules are randomly located.^259^

By means of robotic high-throughput optimization strategies, Cooper and co-workers managed to prepare 29 **CC***n* cage–solvent systems as type II porous liquids.^260^ Dynamic covalent scrambling can be used to increase the solubility of the **CC***n* porous organic cages in different voluminous organic solvents to obtain type II porous liquids with empty cavities. Such porous liquids were found to adsorb large quantities of gases with a high percentage of cage occupancy: 72% for Xe (i.e., from 100 molecules of cage 72 containing a Xe molecule) and 74% for SF_6_.^261^

**Figure 115:**
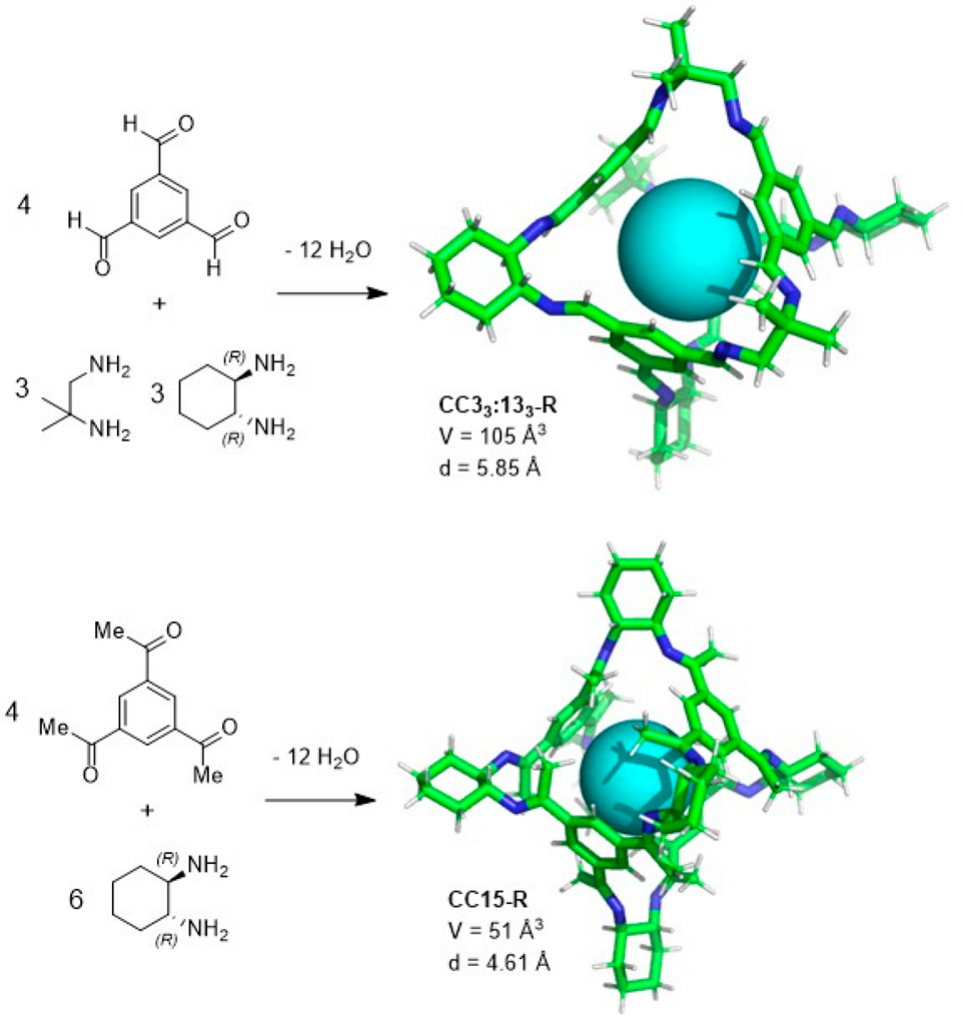
Chemical structures of **CC3**_**3**_:**13**_**3**_-*R* and **CC15-***R*. Porous liquids type II were obtained by dissolving the cage in hexachloropropene.^262^

Cooper, Greenway, and co-workers prepared a series of **CC***n* cage structures (Figure 115) with different window sizes that allowed the tuning of the gas selectivity of the type II porous liquids obtained by dissolving the cage with hexa-chloropropene. The methyl groups in cage **CC15**-*R* were located at the cage windows (see Figure 115, bottom), which brought about a decrease in window size to diameter 1.7 Å compared to 4.0 Å for the scrambled cage mixture **CC3**_**3**_**:13**_**3**_-*R*. A decrease in window size produces a significant reduction in Xe absorption by altering cage selectivity from Xe-selective (for cage **CC3**_**3**_**:13**_**3**_-*R*) to CH_4_-selective (for cage **CC15**-*R*).^262^

**Figure 116:**
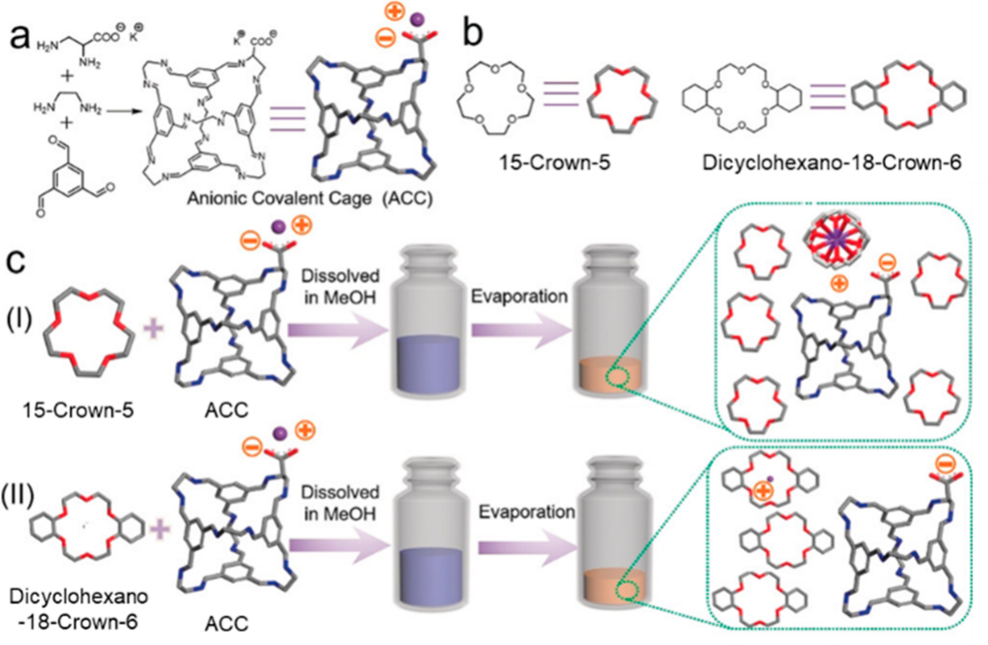
(a) Synthesis of anionic covalent cages. (b) Chemical structures of 15-crown-5 and dicyclohexano-18-crown-6. (c) Synthetic procedures for the crown ether-**ACC** porous liquids.^263^ Adapted with permission from ref (263). Copyright 2020 Wiley-VCH.

Dai and co-workers prepared a proof-of-concept type I porous liquid using anionic porous organic cages (**ACC**) and the K^+^/crown complex as the cation. To this end, they employed a cage system based on the analogous neutral **CC1** cage^57^ by incorporating one carboxylic group per **ACC** cage molecule (Figure 116). To transform the porous **ACC** solid into a porous liquid, the supramolecular complexation of K^+^ with dicyclohexano-18-crown-6 (higher affinity) and 15-crown-5 (lower affinity) allowed a supramolecular cationic complex with a larger size to be obtained, which transformed the initial solids into viscous liquids. The addition of 15-crown-5 in a 2:1 cage/crown-ether ratio yielded solids, whereas excess 15-crown-5 afforded a type II porous liquid. In contrast, the addition of 18-crown-6 at the 3:1 cage/crown-ether ratio yielded a type I porous liquid. This system enhanced thermal stability as a consequence of the high dicyclohexano-18-crown-6/K^+^ binding strength and the high boiling point of dicyclohexano-18-crown-6. The authors suggest that this strategy can be extrapolated to prepare porous liquids with larger pores or anionic porous nanoparticles. The authors demonstrated that cages had empty cavities in the liquid phase by measuring CO_2_ sorption capacities. The values obtained for the porous liquid formed by 15-crown-5 and dicyclohexano-18-crown-6 were 0.375 and 0.429 mmol/g, respectively (Figure 116c). These adsorption capacities are lower than for solid **ACC** (1.062 mmol/g), which has been attributed to the intrinsic and extrinsic pores present in solid **ACC**, while the two porous liquids do not have extrinsic porosity and, therefore, the observed porosity is lower for the porous liquids than for the solid cage.^263^

#### 3.3.2 Other Applications

Apart from using cages for gas adsorption, the development of materials based on molecular cages allowed the porous properties of cages to be employed in other different applications, such as manufacturing cage-based porous columnar materials as solid-stationary phases for chromatographic separations, porous membranes, and solid supports for catalysis.^264^ Composite materials that incorporate molecular cages’ porosity properties can be obtained by depositing molecular cages on surfaces to form coherent crystalline thin films and to obtain membranes that can be effective for molecular separations.^265^ Besides the possibility of manufacturing composite materials, cage stability plays a key role and, in this regard, the crystals of cage **CC3**, stable in water, yielded water encapsulation. All of this suggests that the practical applications of these materials in wet environments are possible.^266^ Hybrid quantum dots functionalized with cages have also been used for sensing nitrophenol isomers and enantiomers of phenylalaninol and phenylethanol by fluorescence measurements.^267^

Enantioselective potentiometric sensing is also possible using cage systems, as demonstrated by Yan and co-workers. By employing membrane electrodes based on cage **CC9** (see structure in Figure 30), they were able to perform the enantioselective sensing of 2-aminobutanol.^268^ Cage **CC3**-*R* has been used for enantiomeric resolution by NMR, which illustrates the potential of cages for determining enantiomeric ratios.^269^ Further applications in sensing include the detection of vapors of aromatic solvents by guest encapsulation in thin films of cage molecules on quartz crystal microbalances,^270^ as well as the sensing of the vapor of the γ-butyrolactone (GBL) drug.^271^

Other applications include nanofiltration, which utilizes porous organic cage-based membranes for water purification,^272^ gas chromatography enantiomeric separation with homochiral porous organic cage **201** (see Figure 61) as the chiral stationary phase,^273−275^ open tubular capillary electrochromatography,^276^ or gas storage.^277^

## 4 Cages and Containers Soluble in Water and Their Applications

The cage structures presented in this review up to this point have a considerable hydrophobic character, which makes them soluble in organic solvents but insoluble in water. To achieve water solubility, employing water-solubilizing groups is a key aspect as described in section 2.2. This section describes examples of cavitands, capsules, and cages with water-solubilizing groups and their applications as hosts of hydrophobic guests. Guest encapsulation in an aqueous medium is driven by the hydrophobic effect, which plays a key role and is the main driving force of supramolecular complex formation. As the hydrophobic effect is driven by the exclusion of water molecules from a hydrophobic surface, the hydrophobic force increases with the hydrophobic surface area, i.e., larger surfaces have higher interaction energies.^278^

### 4.1 Cavitands

**Figure 117:**
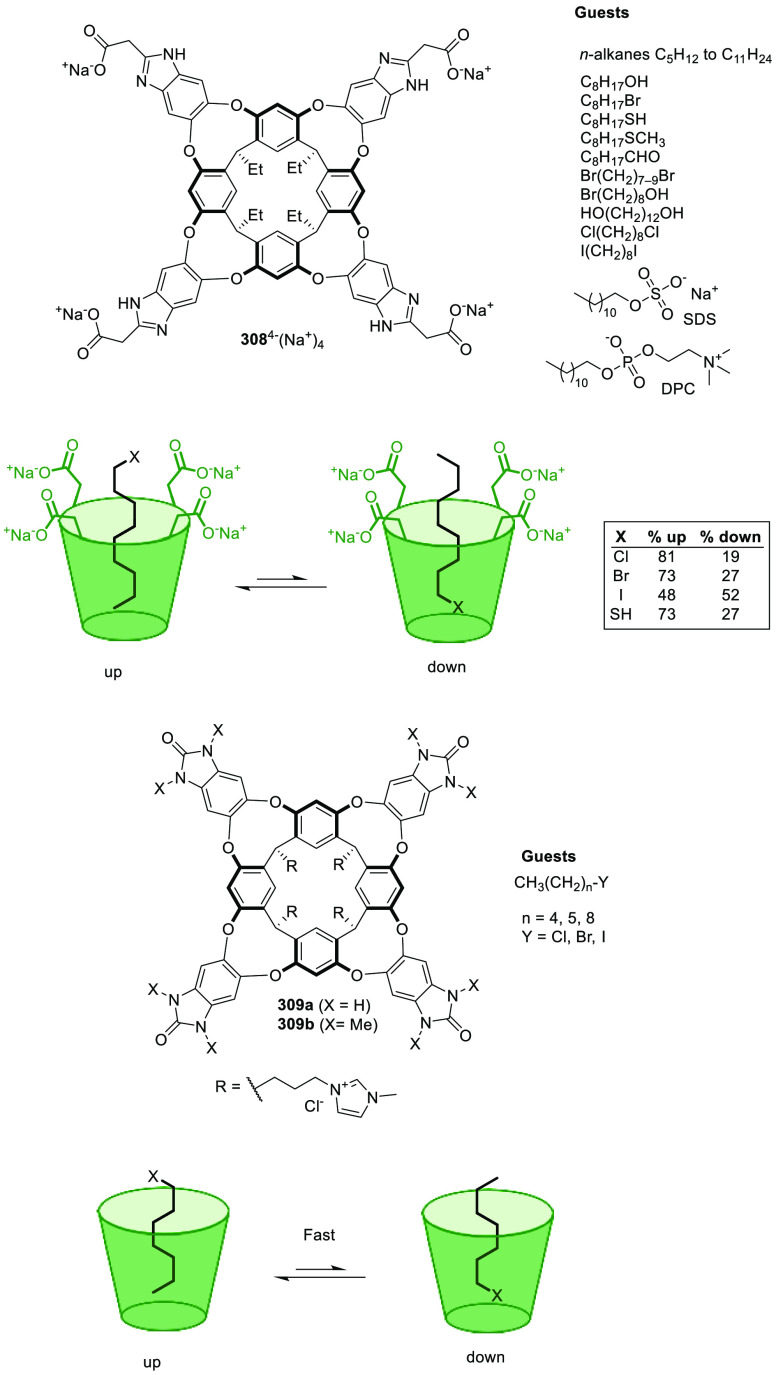
Chemical structure of water-soluble cavitands **308** and **309** and its guest. Biased tumbling of the α-substituted alkanes bound in the cavitand.^279−281^ Chemical structures of water-soluble cavitands **309** and guests C_*n*_–Y (*n* = 5, 6, and 9; Y = Cl, Br, and I).^282^

In 2003, Rebek and co-workers reported water-soluble cavitand **308** after incorporating four carboxylic groups to achieve water solubility. Moreover, **308** shows high affinity toward hydrophobic molecules (Figure 117). While alkanes adopted extended conformations in solution that minimized steric interactions and maximized the surface area, when encapsulated into cavitand **308** alkanes folded to reduce the amount of hydrophobic surface exposed to solvent. This resulted in unfavorable gauche interactions. The authors found that the alkyl chains of two common surfactants (sodium dodecyl sulfate, SDS, and dodecyl phosphatidyl choline, DPC) adopted helical conformations by encapsulation. The polar head groups placed in the cavitand opening exposed them to the aqueous solvent, and the filling of space and hydrophobic surface burial were the driving force.^279^ This behavior is also observed when binding *n*-alkanes (C_5_H_12_–C_11_H_24_), which coil into helices in the hydrophobic cavity while rapidly tumbling on the NMR time scale.^280^ Substituted hydrocarbons (C_8_H_17_OH, C_8_H_17_Br, C_8_H_17_SH, C_8_H_17_SCH_3_, C_8_H_17_CHO, Br(CH_2_)_7–9_Br, Br(CH_2_)_8_OH, HO(CH_2_)_12_OH, Cl(CH_2_)_8_Cl, I(CH_2_)_8_I) are bound in quite a different way because the nature of the headgroup determines guest orientation in the cavity. Polar oxygen substituents are found exclusively on the cavity rim, whereas less hydrophilic groups, such as halides and thiols, adopt conformations with the groups at the base and at the cavity rim by rapidly interconverting (see the up–down conformations in Figure 117).^281^ On the basis of water-soluble cavitand **308**, Rebek and co-workers also prepared water-soluble cavitands **309a** and **309b**, which offer good (>1 mM) solubility in water. These hosts bind primary alkyl halides C_*n*_–Y (*n* = 5, 6, and 9; Y = Cl, Br, and I) to form dynamic complexes where the host and guest adapt their shapes to accommodate one another. The guest undergoes yo–yo-like tumbling in the cavity with a preferred orientation toward the floor of the cavitand. The preferred orientation is consistent with their hydrophobicity, and the most hydrophobic iodide avoids coming into contact with aqueous medium more than bromide and chloride do. The larger iodide fraction in the down position indicates halogen bonding, even though the deconvolution of hydrophobic and halogen bonding was not possible (Figure 117).^282^ These findings generally suggest a general mode of binding for substrates by making the best of hydrophobic host–guest interactions.

**Figure 118:**
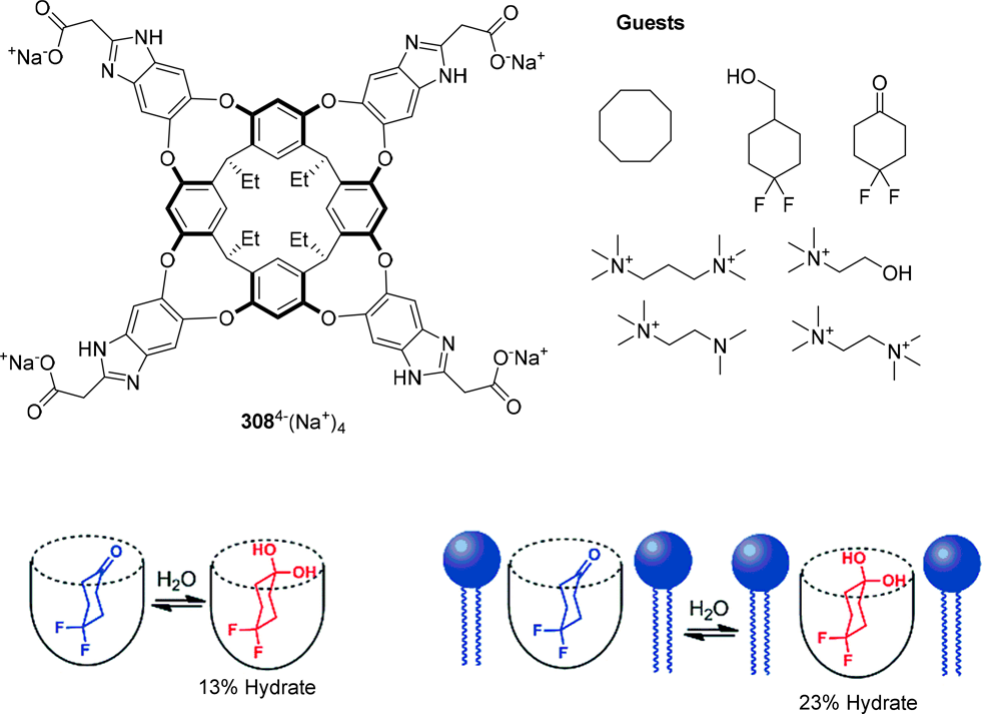
Structure of water-soluble deep cavitand **308** and the guests and the lipidic bilayer.^283^ Adapted with permission from ref (283). Copyright 2018 the authors of the original publication. Published by the Royal Society of Chemistry with the Creative Commons CC BY license http://creativecommons.org/licenses/by/3.0/.

Hooley and co-workers used guests with NMR detectable nuclei (^13^C and ^19^F) to analyze how the external environment affected the recognition properties of water-soluble deep cavitand **308**. The authors found that by inserting deep cavitand **308** into lipidic bilayer environments produced the compression of cavitand flexible walls at the same time as it provided an additional barrier to wall-opening. This compression pushed cavitand walls closer to the guest by forcing it to adopt unfavorable conformations and by modifying reaction equilibria by, for instance, favoring 4,4-difluorocyclohexanone hydration from 13% in the presence of the cavitand to 23% in the presence of both the cavitand and the lipidic bilayer (Figure 118).^283^ In deep cavitand **308**, the encapsulation of trimethylammonium cations is driven by cation−π interactions between the surface of the guest and the polarized aromatic rings of the cavitand. This strong interaction (with binding affinities >10^4^ M^–1^) has been used to prepare water-soluble nanoswitch systems based on derivatized Au nanoparticles with trimethylammonium that close the pores of mesoporous silica loaded with a fluorophore. Upon the addition of the cavitand, pores open and the guest is released.^284^

**Figure 119:**
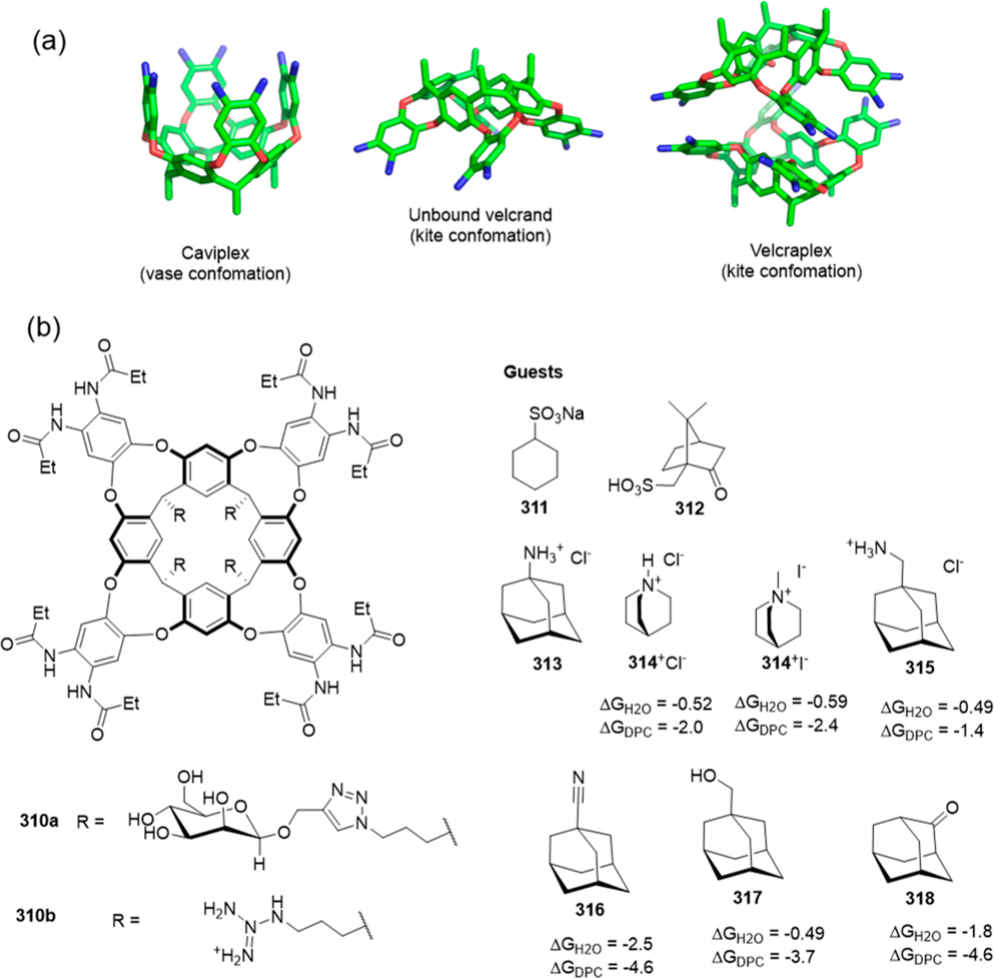
(a) 3D representation of caviplex, unbound velcrand, and velcraplex structures (groups in the nitrogen atoms and water-solubilizing groups are omitted for clarity). (b) Water-soluble cavitand **310a** is used to detect small molecules in human urine and human serum.^285,286^ Water-soluble cavitand **310b** is used for the encapsulation of guests in water and dodecylphosphocholine (DPC) micelles. The displayed Gibbs energy in kcal/mol corresponds to cavitand **310a**.^287^

Rebek and co-workers also developed water-soluble tetrakis(β-d-glucosyl) deep cavitand **310a** with covalently bound carbohydrates to provide solubility in water. Cavitand **310a** was prepared by an azide–alkyne “click” reaction between the tetraazido cavitand and propargyl glycoside. When guests are lacking, in water the cavitand forms a homodimer, probably in a kite conformation that self-assembles into a velcraplex structure, to likely reduce the hydrophobic surface area that is exposed to the solvent. Adding water-soluble guests **311**–**315**, which have the appropriate size to fill the cavitand cavity with hydrophobic alkane surfaces, induces caviplex formation through vase conformation (see the structures of caviplex, unbound velcrand, and the velcraplex structures in Figure 119a). The binding of hydrophobic small organic molecules **314**^+^Cl^–^ and **313** exhibits slow guest exchange on the NMR time scale at 600 MHz, with no cavity occupation by the water-solubilizing carbohydrate. The authors also observed complex formation with alkylammonium salts **314**^+^ Cl^–^ and I^–^ in both human serum and spiked samples of human urine. These results reveal the potential of synthetic receptors for applications in biofluid diagnostics, but the authors point out that the binding affinity of the cavitand to guests needs to be increased for real applications (Figure 119b).^285,286^

The same cavitand framework, but by incorporating four guanidine groups (**310b**) for water solubility, also presents two conformations in equilibrium: unbound velcrand and guest-induced vase stabilized by the formation of intramolecular hydrogen bonds (N···H···O) by the amide groups of the cavitand. In the host–guest complex, the guest polar groups are oriented toward the solvent-accessible open end of the receptor, while the hydrophobic parts remain inside the host’s electron-rich cavity. The highest affinities are for the most hydrophobic guests **316**–**318**, whereas polar adamantanes **315** show the lowest binding affinity. Quinuclidinium guests **314** possess intermediate affinities, which suggests that they can participate in additional cation−π interactions in the complex. The binding observed for the different guests in water is generally modest. However, in the presence of dodecylphosphocholine (DPC) micelles, the cavitand is positioned at the DPC–water interface, which increases the binding strength, with the affinities typically observed for organic solvents. The increase in binding free energies in the presence of DPC (up to 3.2 kcal/mol) is enthalpic in nature and is attributed to the disruption of velcrand dimers (Figure 119b).^287^

**Figure 120:**
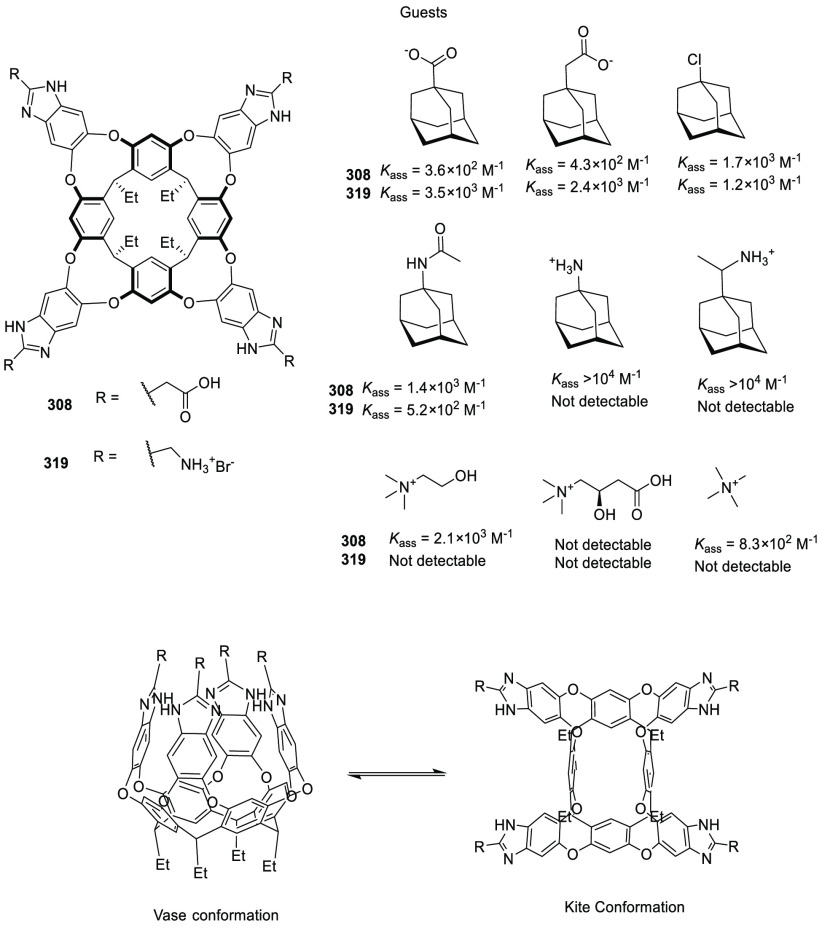
Water-soluble cavitands **308** and **319** used for the encapsulation of different guests and the vase–kite conformation equilibrium.^288^

Rebek and co-workers described the synthesis of water-soluble cavitand **319** by incorporating ammonium solubilizing groups. The cavitand has a kite conformation with *C*_2*v*_ symmetry in water, whereas when using DMSO, THF, or methanol as cosolvents, the cavitand turns into a vase conformation with *C*_4*v*_ symmetry. The authors found that the cationic compounds described in Figure 120 did not bind by tetrammonium cavitand **319**, unlike cavitand **308** that binds these cationic guests with high affinity. Contrary to that trend, cavitand **319** is a very specific receptor for neutral or anionic adamantane derivatives and has similar affinities to cavitand **308** (see the association constants in Figure 120).^288^

**Figure 121:**
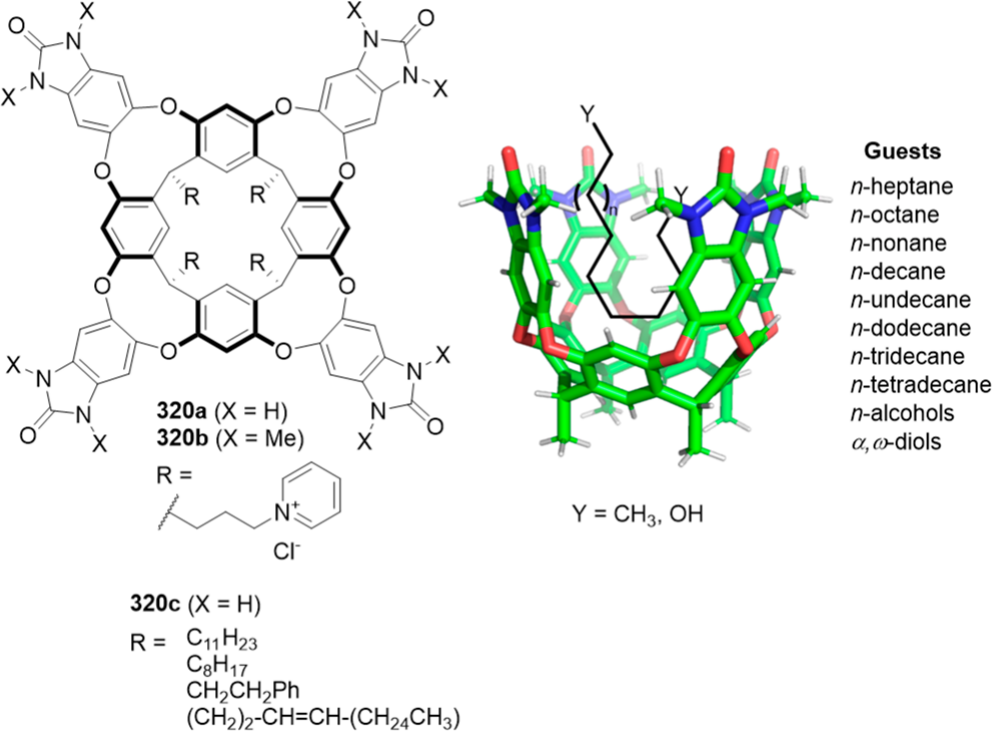
Water-soluble capsules **320** and representation of the complexes with guests in a folded conformation.^291,292^ R groups are replaced with Me groups in the 3D representation for simplicity.

Rebek and co-workers reported the synthesis of deep cavitand **320a**–**320b** with pyridinium “feet” to show good solubility in aqueous media.^289^ This cavitand system with benzimidazolones on the upper “rim” **320c** was reported in 2002 by de Mendoza and co-workers (Figure 76). Cavitand **320c** dimerizes in organic solvents to form a capsule that encapsulates diverse guest molecules.^290^ In another work, Rebek and co-workers found that, despite the solvent’s hydrogen-bond competitive nature, water-soluble cavitand **320a** also dimerizes in water in the presence of lengthy hydrophobic guests (*n*-heptane, *n*-octane, *n*-nonane, *n*-decane, *n*-undecane). Whereas shorter alkanes flip rapidly on the NMR time scale; *n*-decane shows a second set of signals, which indicates the exchange of *n*-decane between the two magnetic environments (i.e., yo-yo motion of the guest in the cage cavity) on the NMR time scale (Figure 121).^291^ To prevent dimer formation, Rebek and co-workers *N*-methylated the benzimidazolones on the upper rim to obtain cavitand **320b**. Surprisingly, they found that long-chain *n*-alkanes, *n*-alcohols, and α,ω-diols adopt folded conformations to fit into the hydrophobic part in the cavity. Small *n*-alkanes (C_6_ to C_11_) tumble rapidly in the cavity and show time-averaged symmetric environments by NMR. In contrast, longer *n*-alkanes (C_13_ to C_14_) display different behavior with greater shielding on the central CH_2_ groups, which are placed near the cavitand floor in a folded conformation that also induces the widening of the open end. For *n*-alcohols and α,ω-diols, the position of CH_2_–OH is near the cavitand upper rim, which changes the alkyl chain conformation (Figure 121).^292^

**Figure 122:**
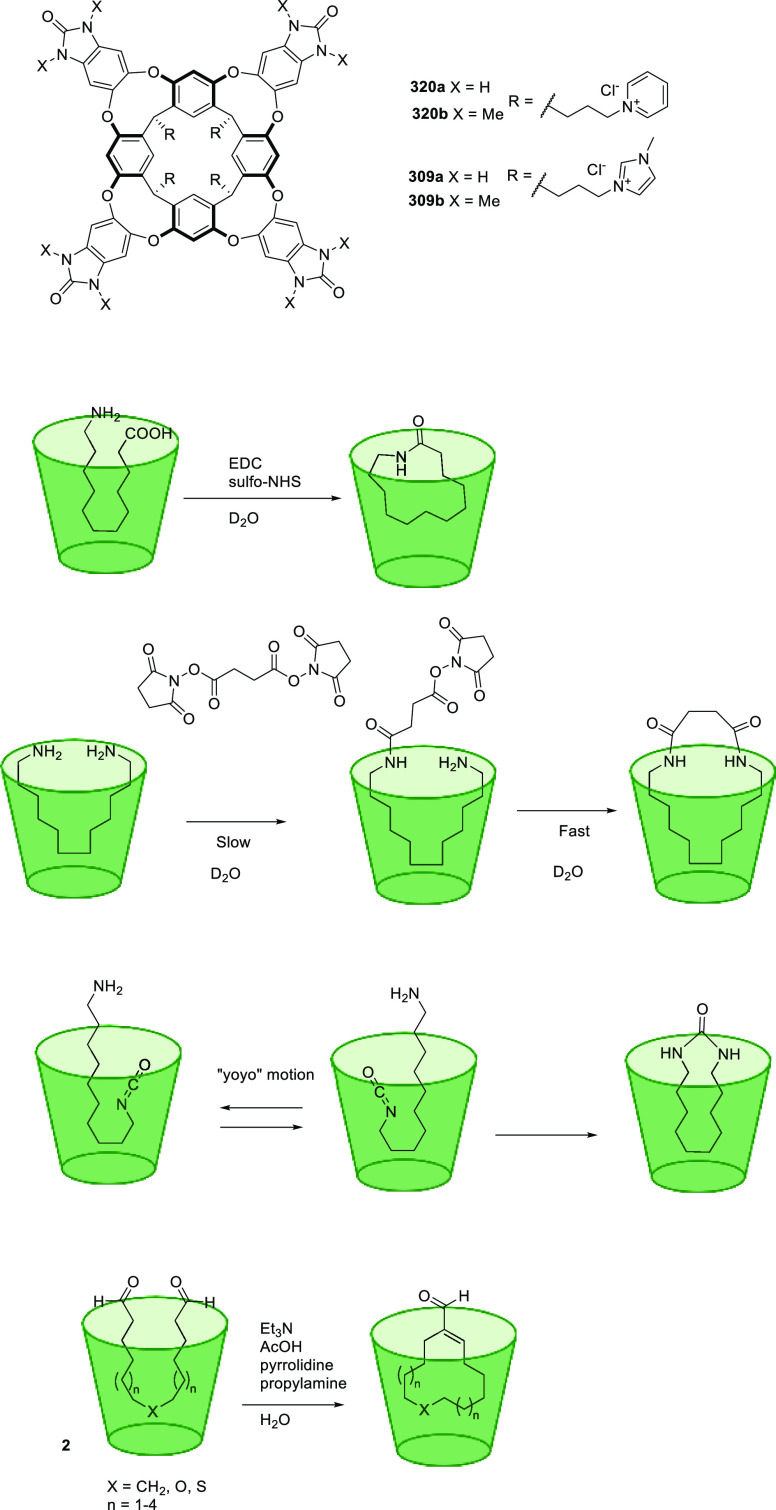
Water-soluble deep cavitands **309** and **320** used to template the macrocyclization of lactams,^293,294^ the cyclization of long-chain diisocyanates to cyclic ureas in water,^296^ and selective cyclization through an aldol/dehydration reaction of long-chain α,ω-dialdehydes in water.^297^

Rebek and co-workers reported the binding and reactivity of long-chain ω-amino acids (C11 and C12) and α,ω-diamines (C11 to C18) in cavitand hosts **309** and **320** in water. Binding involves the folding of the long carbon chain to bury the hydrocarbon chain into the cavity to expose the polar amino and carboxylic groups to the aqueous medium. The cavitand acts as a template for the cyclization of ω-amino acids with EDC/Sulfo-NHS and also for diamine acylation with succinic acid with EDC. In both reactions, yields improved 4-fold compared to the nontemplated reaction (Figure 122).^293,294^ Long-chain aliphatic guests were encapsulated in the hydrophobic cavity of the cavitand in the vase conformation. In contrast, when no guest was present, the cavitand self-assembled quantitatively into a velcrand dimer in D_2_O at millimolar concentrations.^295^ As the binding strength of the lactamization product is higher than the binding strength of reactants, classic product inhibition takes place. Therefore, the cavitand acts as a stoichiometric template, limiting the application as a catalytic template. The same cavitand structure templates the cyclization of medium- to large-sized rings through the conversion of long-chain diisocyanates to cyclic ureas in water. Hydrophobic forces drive the long chain of diisocyanates into cavitands in folded conformations, which are not observed in bulk solution, and the reacting ends in close proximity to favor and accelerate the cyclization reaction (Figure 122).^296^ Cavitand **309** templates selective cyclization through an aldol/dehydration reaction of long-chain α,ω-dialdehydes in water. Hydrophobic forces drive the encapsulation of dialdehydes into cavitands in folded conformations, which favors macrocyclization over the unwanted intermolecular reactions observed in bulk solution. The 11- to 17-membered ring macrocyclic aldol reaction products are isolated in 30–85% good yields using the cavitand template.^297^ One of the drawbacks is the need for a stoichiometric amount of the cavitand. The authors point out that efforts are being to develop a catalytic version. Overall, the development of templates based on container molecules opens up new catalysis avenues. This allows use of container concave surfaces, which induce folded conformations to template reactions, unlike conventional templates that wrap reacting components around to bring relevant functions together. The observed cavitand templated effect is encouraging for water-soluble cavitands applications to achieve other selective reactions, such as remote functionalization.

**Figure 123:**
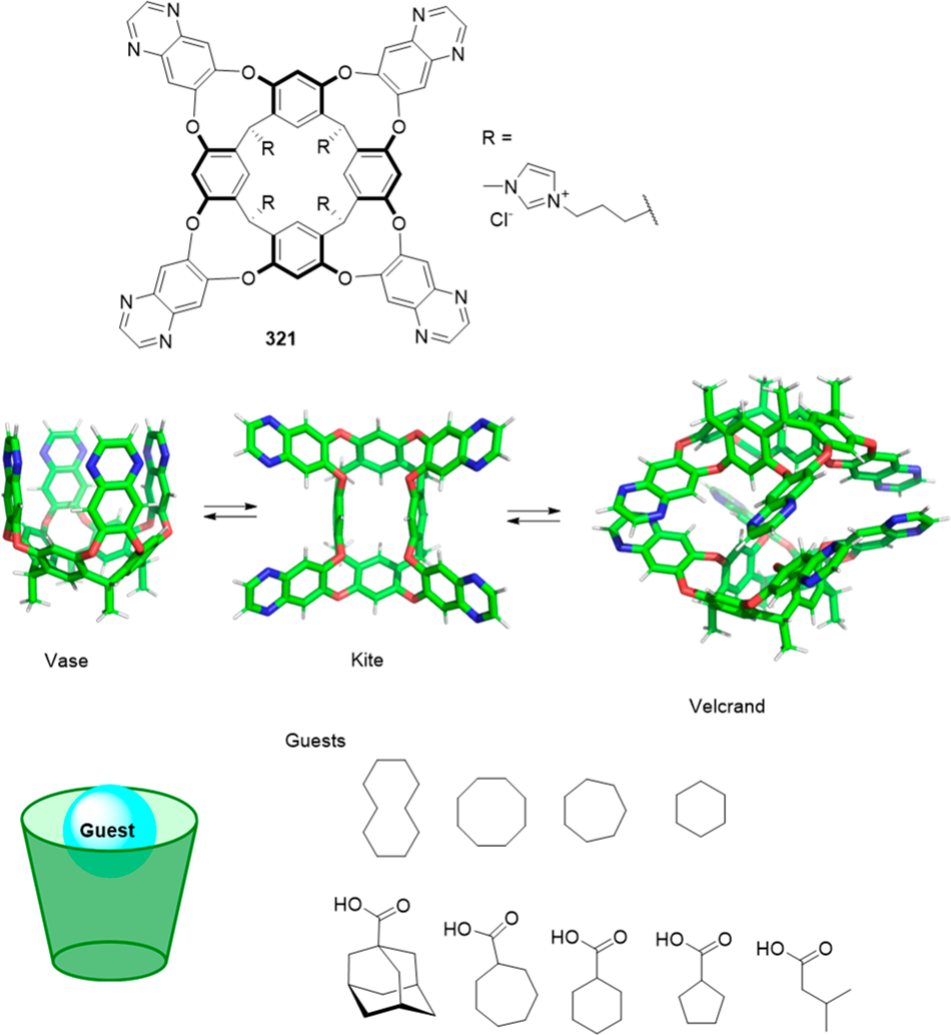
Different possible conformations of **321** and the host–guest complexes observed with the vase conformer.^298^

Yu and co-workers prepared water-soluble cavitand **321**, based on Rebek’s previous cavitand **309a** (see Figure 122), which incorporates quinoxaline motifs by adding solvent-dependent conformational preferences (vide infra) to the cavitand structure (see Figure 123). This cavitand had better water solubility and a larger aromatic cavity compared to Rebek’s cavitand. Cavitand **321** in DMSO-*d*_6_ appeared only in the vase conformation, in D_2_O in the kite or dimeric kite conformation (velcrand), whereas a complex mixture of conformations was observed in MeOD-*d*_4_ as determined by NMR. Despite the unfavorable kite conformation observed in water, the cavitand was able to encapsulate hydrophobic (cycloalkanes) and amphiphilic (cycloalkyl carboxylic acids) guests, which suggests the presence of a deep aromatic pocket. In fact upon guest binding, the kite conformation is converted into the vase conformation to better accommodate the hydrophobic guest. The binding of cycloalkanes involved placing the molecule in the cavity, and with cycloalkyl carboxylic acids, the alkyl part was placed inside the cavity with the hydrophilic carboxylic acid group exposed to the aqueous environment. The authors suggest that the N-donor atoms near the top of the cavitand can be used for the coordination of metals for catalysis applications (Figure 123).^298^

**Figure 124:**
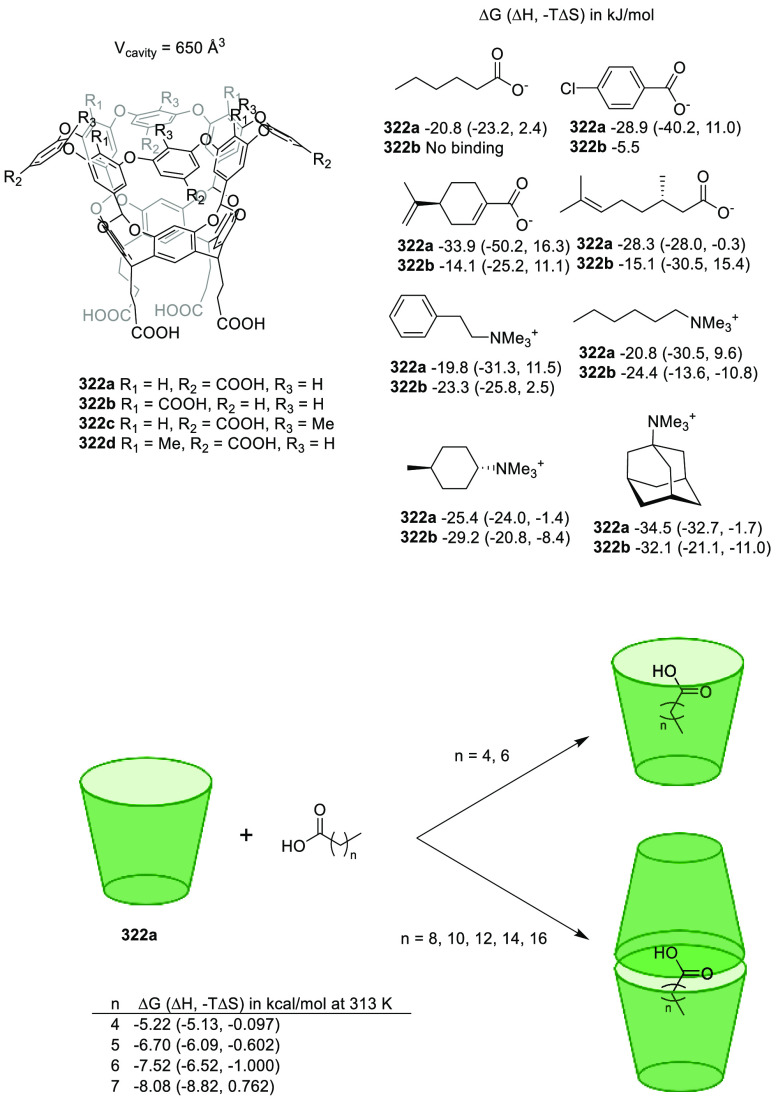
Structures of the octa-acid cavitands and binding data.^299^ Tetra-*endo*-methyl octa-acid **322c** self-assembles into tetrameric and hexameric assemblies in the presence of *n*-alkanes.^301^ The dewetting process of **322c**.^302^ Encapsulation of fatty acids.^300^

Gibb and co-workers determined the structural factors for efficient guest encapsulation in water-soluble cavitands **322a** and **322b**, which differed only in the position of the carboxylic groups that resulted in subtle shaped differences of pocket surfaces by providing a distinct hydrophobicity that resulted in a pocket in a dry or wet state depending on the energy of water molecules in the cavity. These differences in energy produced weaker binding for the pockets in a wet state compared to the pockets in a dry state. This finding suggests a new perspective for the role of water molecules in nonpolar host cavities. To draw these conclusions, the authors determined the affinity of eight guests (Figure 124) with the two cavitands, with a lower affinity for cavitand **322b** than for cavitand **322a** in all cases. A plausible explanation for this observation was obtained by molecular dynamics simulations, which pointed to the increased wetting of the pocket of **322b**, with stronger ion–dipole interactions between the host and bound water and, therefore, rendered it energetically unfavorable for the guest to displace cavity water. Another important determined aspect was the difference in affinity between positive and negative guests binding; negative guests binding was slightly stronger to **322a** than the positively charged ones. The authors suggest that this is the result of complex ion–ion and ion–dipole interactions in the different host–guest complexes that cannot be simply modeled (Figure 124).^299^ The encapsulation of fatty acids by **322a** shows dependence on hydrocarbon chain length, and is enthalpically driven and entropically neutral. While shorter chains favor 1:1 complex formation, longer chains prefer a 2:1 host–guest stoichiometry, i.e., encapsulation of the guest in a capsule structure. For the medium-sized chains of fatty acids, guest protonation/deprotonation plays a role in the formation of the 1:1 or 1:2 complex, which favors the 2:1 host–guest complex at a low pH where fatty acid is protonated. At basic pH values, the energy balance between the solvation of hydrophobic surfaces in both the host and guest, and the solvation of the guest carboxylate groups, favors the 1:1 complex (Figure 124 bottom).^300^

Tetra-*endo*-methyl octa-acid **322c** (Figure 124) in the presence of *n*-alkanes (C17–C26) self-assembles via the hydrophobic effect into tetrameric (*V*_cavity_ = 1400–1500 Å^3^) and hexameric (*V*_cavity_ = 3200–3700 Å^3^) assemblies by encapsulating two and three guest molecules, respectively. For *n*-hexacosane (C26), the inner nanospace is over 5-fold the volume of the dimer of octa-acid **322c**.^301^ The orientation of methyl groups can trigger the drying of nonpolar pockets in water and, therefore, the position of methyl groups triggers the dewetting process. In the absence of methyl groups (**322a**) or when they point *upward* (**322d**), the cavity remains wet. However, when methyl groups are placed *inward* (**322c**), the water evacuation thermodynamics from the host pocket are similar to capillary evaporation. In this way, the dewetting process of **322c** leads to increased affinity to guest complexation, which is enthalpically and entropically favored (Figure 124).^302^ Studies using pyrene as the templating guest reveal that capsule formation involves the rapid (<1 ms) formation of a pyrene⊂cavitand inclusion complex (pyrene⊂**322a**), followed by the slower binding of a second cavitand molecule to yield the pyrene⊂capsule complex (pyrene⊂(**322a**)_2_) with a dissociation lifetime of 2.7 s.^303^

The molecular dynamics simulations of **322a** and **322c** show that the minor chemical modifications to the constituents of cavitands can significantly impact their structure and host–guest properties. Cavitand **322a** forms host–guest complexes with alkanes (C1–C16, methane to hexadecane) with 1:1, 2:2, and 2:1 molar host–guest ratios. In contrast, **322c** also forms host–guest complexes with alkanes with the same molar host–guest ratios (1:1 to 2:2 to 2:1) but, in this case, switching between the monomeric and the dimeric assembly with increasing chain length is not observed (see Figure 124, bottom). The extra endo-methyl substituents that differentiate **322c** from **322a** tend to crowd alkanes in dimers by reducing the conformational freedom of guests and destabilizing the complexes of **322c** with guests containing 12 carbons or more. Additionally, this tightening increases the repulsive contributions in the 2:2 and 2:1 complex dimerization free energy for the shorter alkanes in **322c** versus than observed for **322a** (Figure 124).^304^ The authors point out that the developed methodology based on molecular dynamics simulations will be useful for understanding the impact of host chemical modifications on their host–guest properties to help with bottom-up designs.

**Figure 125:**
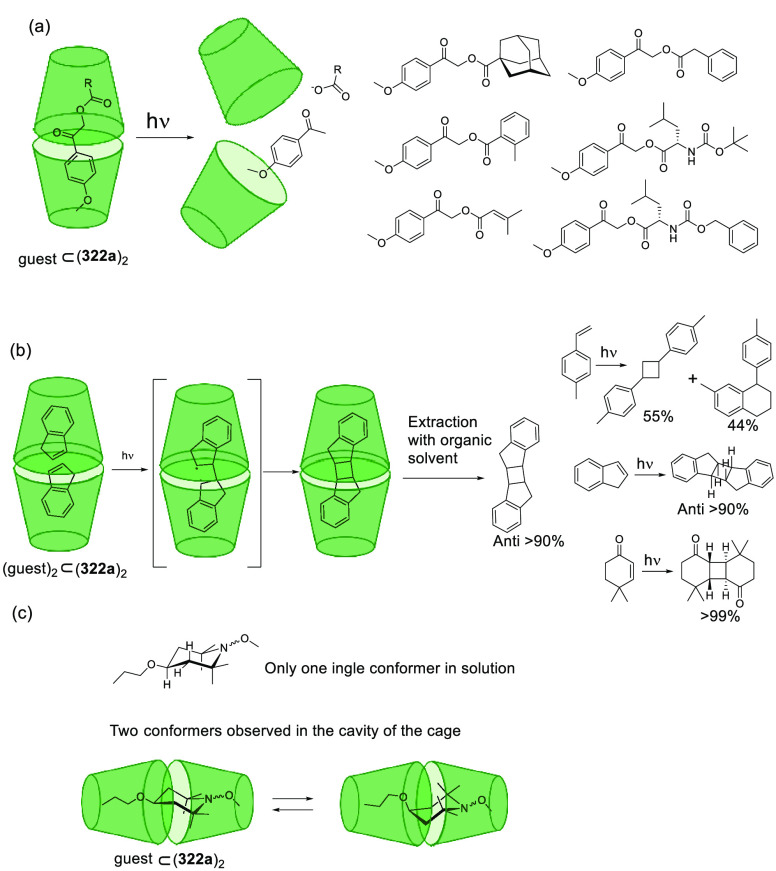
Reactions using cavitand **322a** from Figure 124. (a) Release of organic acids from encapsulated *p*-methoxyphenacyl esters.^305^ (b) Photodimerization of encapsulated hydrophobic organic guests^306^ with the proposed reaction pathway to exclusively yield the anti dimer.^307^ (c) Trapping of high-energy conformers by encapsulation.^309^

Ramamurthy and co-workers reported the formation of stable capsuplexes (guest⊂capsule) in aqueous solution using cavitand **322a** and a range of guests that can undergo light-induced β-cleavage. This property allows capsuplexes to be opened to release guest molecules in aqueous media by using light. The studied guests are not soluble in water in the absence of cavitand **322a**. This proof-of-principle example could result in a general supramolecular photochemical strategy to release chemicals to aqueous solution containing encapsulated hydrophobic precursors (Figure 125a).^305^ The authors also tested the photodimerization of encapsulated hydrophobic organic guests. For this purpose, they encapsulated in the hydrophobic cavity of water-soluble cavitand **322a** the water-insoluble guests, *p*-methylstyrene, indene, and 4,4-dimethylcyclohex-2-enone, via the formation of 2:2 host–guest inclusion complexes (guest)_2_⊂(**322a**)_2_. Irradiation of the aqueous solutions of the inclusion complex with a mercury vapor lamp induced the corresponding dimerization reactions with good yields (Figure 125b).^306^ Encapsulation produces the preorganization of guest molecules by favoring dimer formation, which are not generally formed under conventional conditions. In particular, the templated photodimerization of indene proceeds with high selectivity to the antiproduct with >90% yield. This high efficiency was ascribed to guest cavity occupancy and the guest–cage noncovalent interactions with a reaction taking place by energy transfer from the host to the encapsulated indene guest. This energy transfer results in the formation of an excited triplet of indene, which further reacts to yield the final photodimer (Figure 125b).^307^ The presence of a benzoate anion in the top periphery is essential for the cavitand to be a triplet sensitizer.^308^ The authors envisage that this approach can be used in organic synthesis to perform photoreactions in the cavitand.

The same water-soluble octa-acid cavitand **322a** was used by Ramamurthy and co-workers to trap a high-energy conformer of a piperidine derivative by encapsulation (see structure guest⊂(**322a**)_2_ in Figure 125c). Whereas free propyloxy-substituted piperidine in water exclusively adopts a single conformation in which the alkoxy group is equatorially positioned, an additional less stable conformer is also observed by NMR upon encapsulation. This phenomenon was only found for the guest with an *O*-propyl chain, and the related guests with ethyl, butyl, pentyl, or hexyl exclusively adopt the favored conformation with the *O*-alkyl chain placed equatorially in the capsule (Figure 125c).^309^ The researchers suggest that this achievement can be used to trap high-energy conformations of other species in a capsule cavity.

The same research group studied the encapsulation of phenyl-substituted hydrocarbons (alkanes, alkenes, alkynes) of different chain lengths in octa-acid cavitand **322a**. Upon encapsulation inside the hydrophobic microenvironment of the octa-acid capsule (guest⊂(**322a**)_2_), small hydrocarbons (length <15 Å) adopted a linear conformation, but longer ones adopted folded conformations. Such folded conformations, which are unusual and inaccessible in solution, are stabilized by specific C–H/π and π–π interactions between the guest and the capsule.^310^

**Figure 126:**
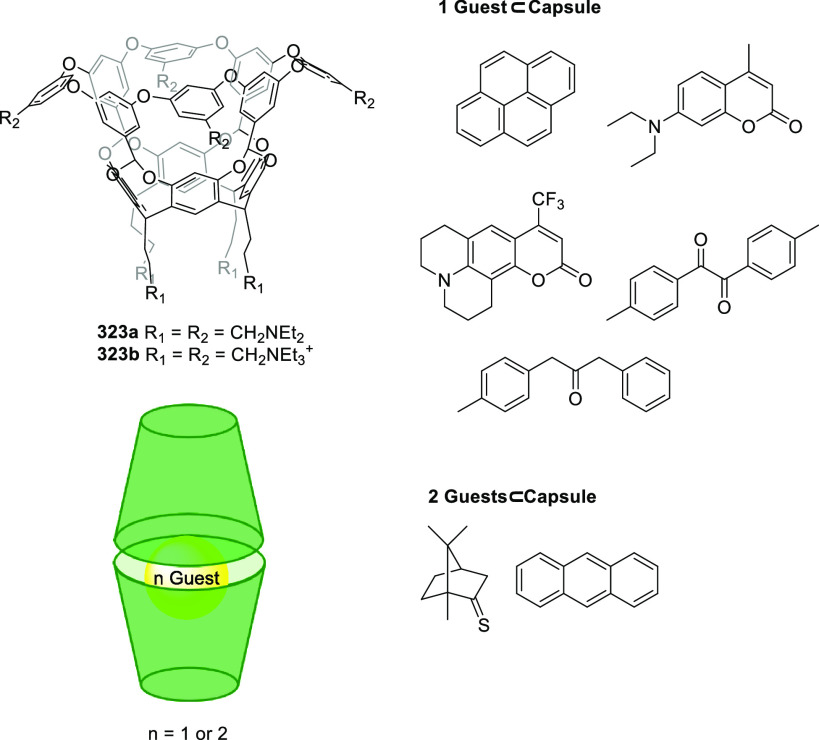
Cavitand containing ammonium water-solubilizing groups **323** and formed capsules.^311^

Ramamurthy and co-workers incorporated ammonium groups in the same cavitand structure (**323b**) to achieve water solubility and also maintaining the host’s ability to alter the guest molecules’ excited state behavior upon encapsulation. Cavitand **323a** with amine groups is soluble in water under acidic conditions, which complements octa-acid capsule **322** that is soluble under basic conditions. Moreover, cavitand **323b** with tetraalkyl ammonium groups is soluble in water under neutral conditions. Cavitands **323a** and **323b** also form capsules with a range of organic molecules in a similar way to parent octa-acid cavitand **322**. The authors found that upon encapsulation, pyrene displayed only monomer emission with an *I*_1_/*I*_3_ ratio of 0.90. The absence of an excimer emission, which was observed for free pyrene in solution, supported the encapsulation of one single molecule in the cage cavity, while the observed *I*_1_/*I*_3_ ratio suggested an internal cavity polarity close to that of benzene (Figure 126).^311^ Cavitand (see the structures in Figure 124 and Figure 126) dimerization to form the corresponding capsule structure took place only in the presence of the templating guest and, additionally, both octa-acid capsule **322a** and octa-amine capsule **323a** exhibited pH-dependent capsule assembly disassembly in water. Octa-acid capsule **322a** formed only under basic conditions, while octa-amine capsule **323a** formed under acidic conditions. Once the capsule had formed, the capsule disassembly rate depends on pH, and also on the guest’s hydrophobicity, which is slower for the more hydrophobic guests.^312^

**Figure 127:**
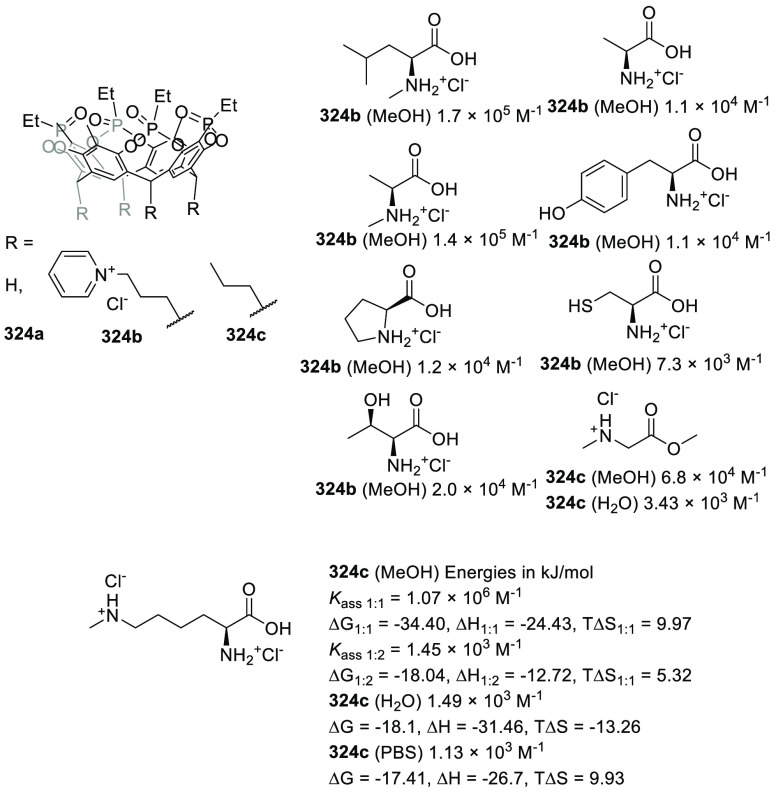
Tetraphosphonate cavitands **324** used for the encapsulation of amino acids.^313^

Dalcanale, Geremia, and co-workers used tetraphosphonate cavitands **324** for the molecular recognition of amino acids. To perform host–guest complexation studies in water, with the core structure of cavitand **324a**, the authors synthesized a water-soluble cavitand by incorporating four pyridinium water-solubilizing groups **324b**. The experiments in methanol were performed using an analogue cavitand containing propyl chains instead of pyridinium groups (**324c**). Whereas the complexation of the guest molecules described in Figure 127 in methanol was entropy-driven, the complexation was enthalpy disfavored in water and resulted in a drop in *K*_assoc_ of almost 3 orders of magnitude. Despite the reduction in binding affinity in water, a remarkable increase in selectivity was observed toward the *N*-methylated amino acids. An analysis of the X-ray solid-state structures indicated that cation−π interactions were key for encapsulation. A direct correlation was found between the *K*_assoc_ in methanol and the depth of the guest in the cavity, evaluated as the distance between the ^+^N–CH_*x*_ guest carbon atom and the cavity entrance plane defined by the oxygen atoms of the P=O groups (Figure 127).^313^

**Figure 128:**
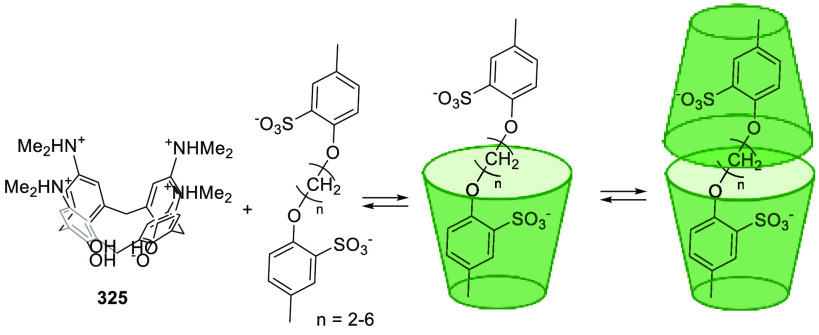
Formation of a capsule from calixarene **325** encapsulating gemini organic dianions.^314^

Sgarlata, Bonaccorso, and co-workers reported tetracationic calix[4]arene **325** as a receptor of several gemini organic dianions containing two aromatic rings and negatively charged sulfonic groups. The formation of homodimeric capsule (guest⊂(**325**)_2_) between tetracationic calix[4]arene **325** and guest molecules was due to hydrophobic and electrostatic interactions. The end of the gemini guest was inserted into the calixarene cavity stabilized by CH−π interactions and hydrogen bonds. The guests with an even number of methylene units acted as better templating agents. Isothermal titration calorimetry (ITC) measurements showed that the formation of the inclusion complex was both enthalpically and entropically favored in association with the release of the water molecules from inside the cavity, followed by the encapsulation of the guest (Figure 128).^314^

**Figure 129:**
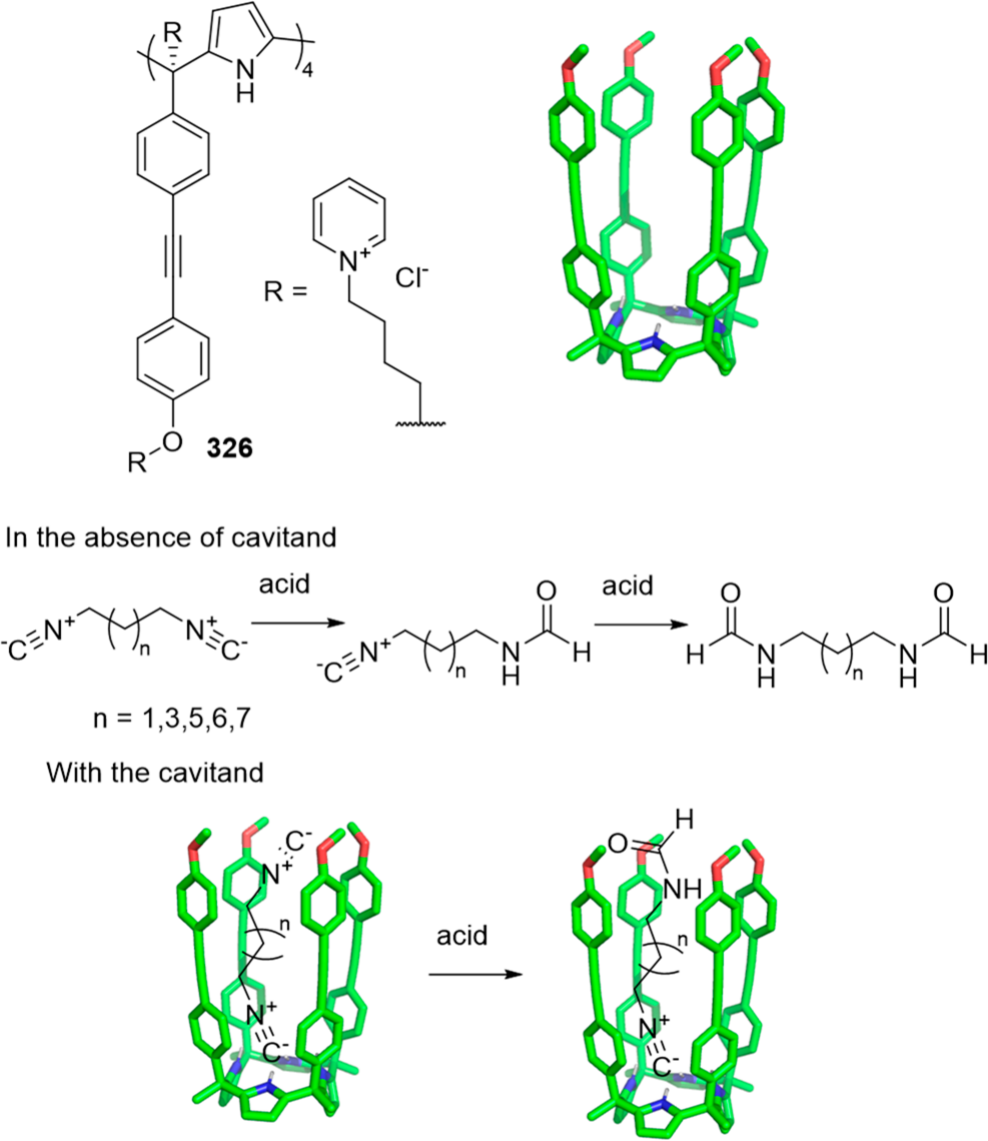
Octa-pyridinium superaryl-extended calix[4]pyrrole **326** that binds neutral difunctional aliphatic guests in water.^315^ R groups omitted for clarity.

Although calixarene cavitands only provide a hydrophobic binding pocket, the use of calix[4]pyrrole hosts provides both hydrogen-bonding and hydrophobic interactions. Ballester and co-workers prepared octa-pyridinium superaryl-extended calix[4]pyrrole receptor **326**, which binds neutral difunctional aliphatic guests in water, the hydrophobic effect, CH−π, NH−π, π–π, and hydrogen-bonding interactions playing an essential role in the binding process. Calix[4]pyrrole receptor **326** forms 1:1 inclusion complexes in water with bis-isonitriles, bis-formamides, and formamide-isonitriles, all with high affinity to display association constants in the order of 10^5^ M^–1^. The *cis*-conformation of the formamide end group is preferentially included in the deep aromatic cavity to form four hydrogen bonds between the oxygen atom of the *cis*-formamide and the four pyrrole NH groups of the host. This host modifies the outcome of the acid-catalyzed hydrolysis of bis-isonitriles by favoring the formation of monoformamides because of the lowering hydrolysis reaction rate constants of the bound substrate. Protection is chain length-dependent, and the hydrolysis of the substrates with five methylene groups allows the corresponding monoformamide with 80% selectivity from the corresponding bis-isonitrile to be prepared (Figure 129).^315^

### 4.2 Capsules and Cages

**Figure 130:**
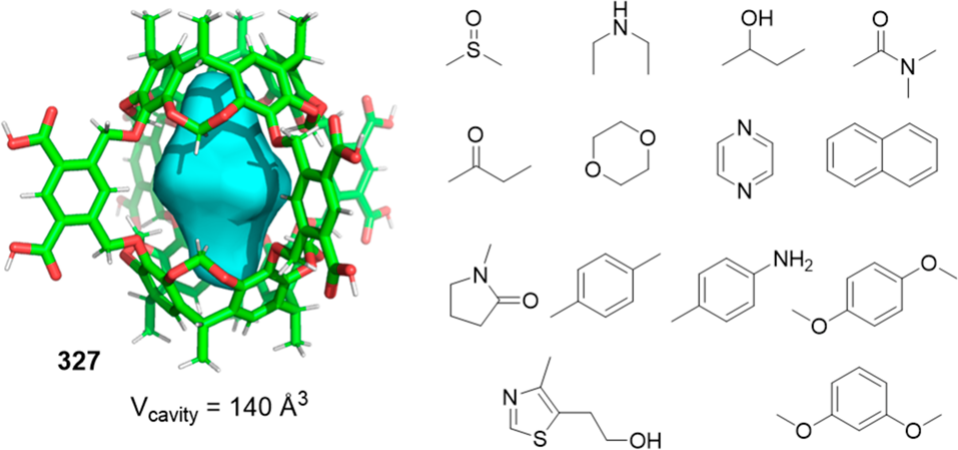
First water-soluble hemicarcerand **327** and its guests.^316^

Cram and co-workers reported the first water-soluble hemicarcerand **327** back in 1997 by using eight carboxylate groups as water-solubilizing groups (Figure 130). The water-solubilizing groups overcome the lipophilic nature of hundreds of known hemicarceplexes at that time, which hampers them from being soluble in water. Guest encapsulation very much depends on guest and cavity solvation, and the fine balance of the energies involved is key in the molecular recognition process. The release of many host innerphase solvating water molecules and guest solvating bulkphase water molecules provides a substantial entropic driving force. A wide range of guests of different sizes, polarity, and water solubility can be encapsulated in **327** to form 1:1 complexes, which are stable at ambient temperature in a medium containing excess guest. The tested guests included different types of compounds (Figure 130), which varied from highly polar Me_2_SO, intermediate polarity-MeC_6_H_4_NH_2_ and 1,4-(Me)_2_C_6_H_4_, to relatively nonpolar 1,4-dimethoxybenzene and 1,3-dimethoxybenzene; and nonpolar naphthalene. For cationic guests (Me_4_NBr, PhNMe_3_Br, BnNMe_3_Br, and 3-MeC_6_H_4_CO_2_Na), the enthalpic water solvation energy of their charges was much higher in the bulk phase than in the interior of the hemicarcerand. Therefore, this factor is dominant in inhibiting complexation (Figure 130).^316^

**Figure 131:**
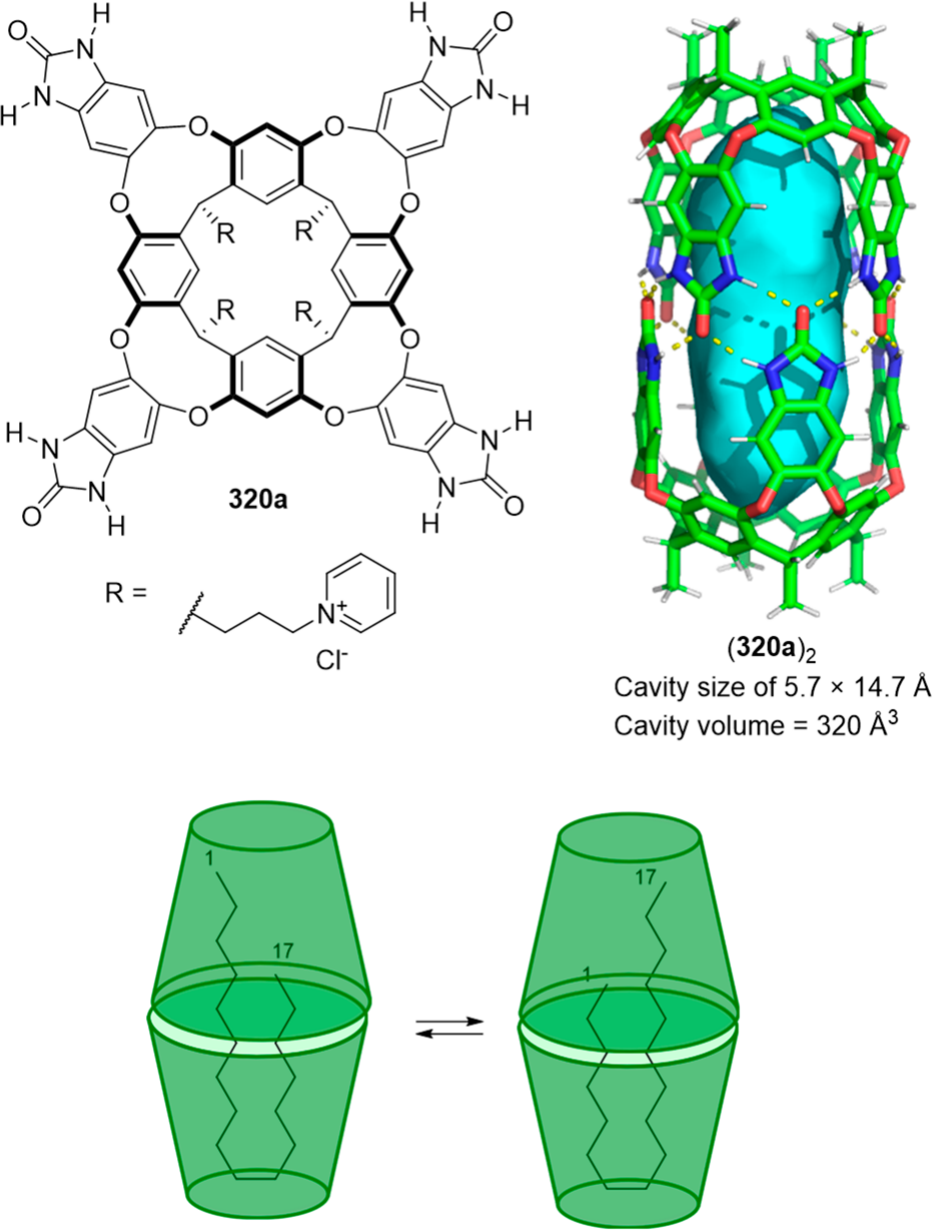
Water-soluble cavitand **320a** and the model of the capsular dimer (**320a**)_2_. Cartoon showing the bended conformation of C17 with a few C atoms in the gauche conformation and the rest of the chain in an extended conformation.^317^

Rebek and co-workers reported the dimerization of water-soluble cavitands around hydrophobic compounds (vide ante). As described in the previous section for water-soluble cavitands, capsules can also encapsulate *n*-alkanes and α,ω-diols (bolaamphiphiles). The authors described how *n*-alkanes are flexible and adapt their shape to the container (Figure 131).^317^ For capsule (**320a**)_2_, addition of 15% hexafluoroisopropanol to water favors the encapsulation of amphiphilic guests.^318^ Flexible guests adopt the conformation that best fills the available space in the cavity, even if high-energy conformations are required. In those cases in which the guest molecule size approaches the cavity volume, a fine balance of attraction and repulsion determines encapsulation performance.^319^ For this latter case, Ouari and co-workers used capsule (**320a**)_2_ to encapsulate several nitroxide spin probes. An EPR spectral-shape analysis provided guest rotational dynamics information inside the capsule. These authors found that minor deformations in either the capsule or the guest took place. Distortion of the ideal hydrogen-bonding pattern between the two cavitands enlarges the central part of the cavity, which allows the encapsulation of larger guests.^320^ This behavior is similar to the dissociation of host subunits by providing a “gap” that allows a guest to enter/exit.^321^

**Figure 132:**
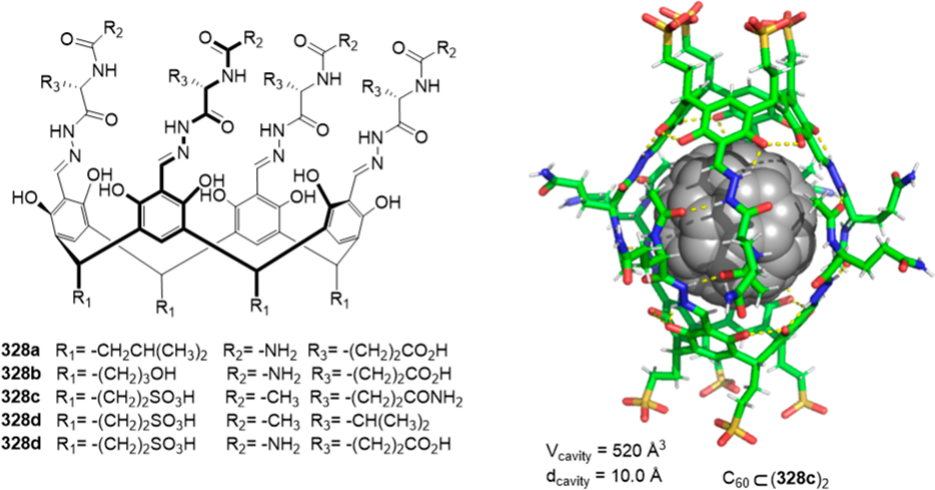
Peptide-based cavitands **328** and the X-ray crystal structure of C_60_⊂(**328c**)_2_.^322^

Szumna and co-workers prepared peptide-based cavitands **328a**–**328e** that could encapsulate fullerene C_60_ (Figure 132).^322^ The NMR experiments showed that cavitands **328a** and **328b** in aqueous K_2_CO_3_ media and **328c**–**328e** in water formed dimeric capsules by encapsulating one molecule of fullerene C_60_ in the central cavity. Small-angle X-ray scattering (SAXS) allowed the experimental core sizes and shapes to be obtained, which agreed with the molecular models and confirmed the encapsulation of C_60_. The formation of these dimeric capsules was ascribed to a combination of C_60_-cage hydrophobic forces and hydrogen-bonding interactions between the peptide chains of cavitands.

**Figure 133:**
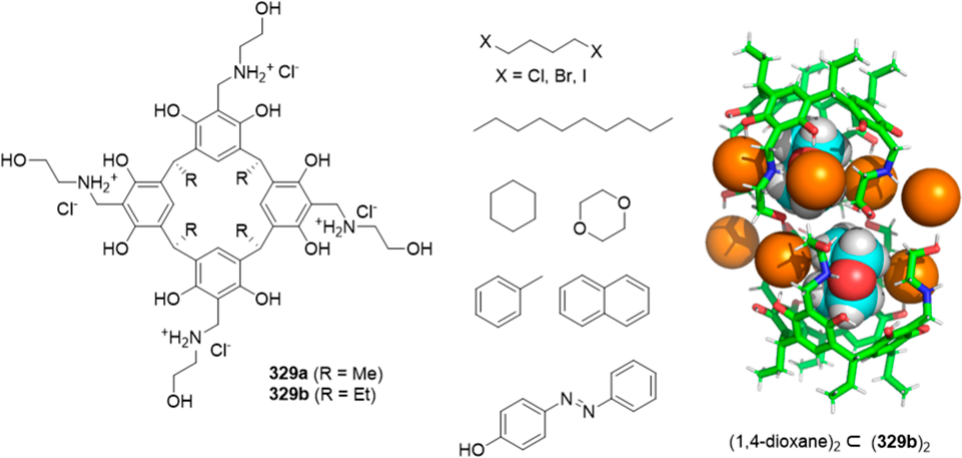
Structure of *N*-ethanol ammonium resorcinarene chloride **329** and guests. The X-ray crystal structure of hydrogen-bonded dimeric capsule (1,4-dioxane)_2_⊂(**329b**)_2_ with two molecules of 1,4-dioxane in the cavity (chloride atoms are represented in orange).^323^

Ras, Beyeh, and co-workers reported the synthesis of water-soluble *N*-ethanol ammonium resorcinarene chlorides **329** functionalized with terminal hydroxyl groups on the upper rim. Cavitands were monomeric in aqueous media and formed 1:1 host–guest complexes by encapsulating linear and cyclic alkanes, linear halogenated alkanes, and aromatic fluorophores via hydrophobic interactions with binding constants of up to 559 M^–1^ in D_2_O/MeOD (9:1, v/v) at 298 K. These cavitands were also able to form capsules in nonaqueous solvents with a cavity volume of 258 Å^3^, where, in this case, NH···halogen and OH···halogen interactions were responsible for capsule formation. The authors were able to obtain the crystal structure of the host–guest complex with 1,4-dioxane. The capsule encapsulated two 1,4-dioxane molecules (molecular volume of 1,4-dioxane = 94.1 Å^3^), which resulted in a packing coefficient of 72.9%. The encapsulation of 1,4-dioxane was driven by a combination of CH−π and hydrogen-bond interactions (Figure 133).^323^

**Figure 134:**
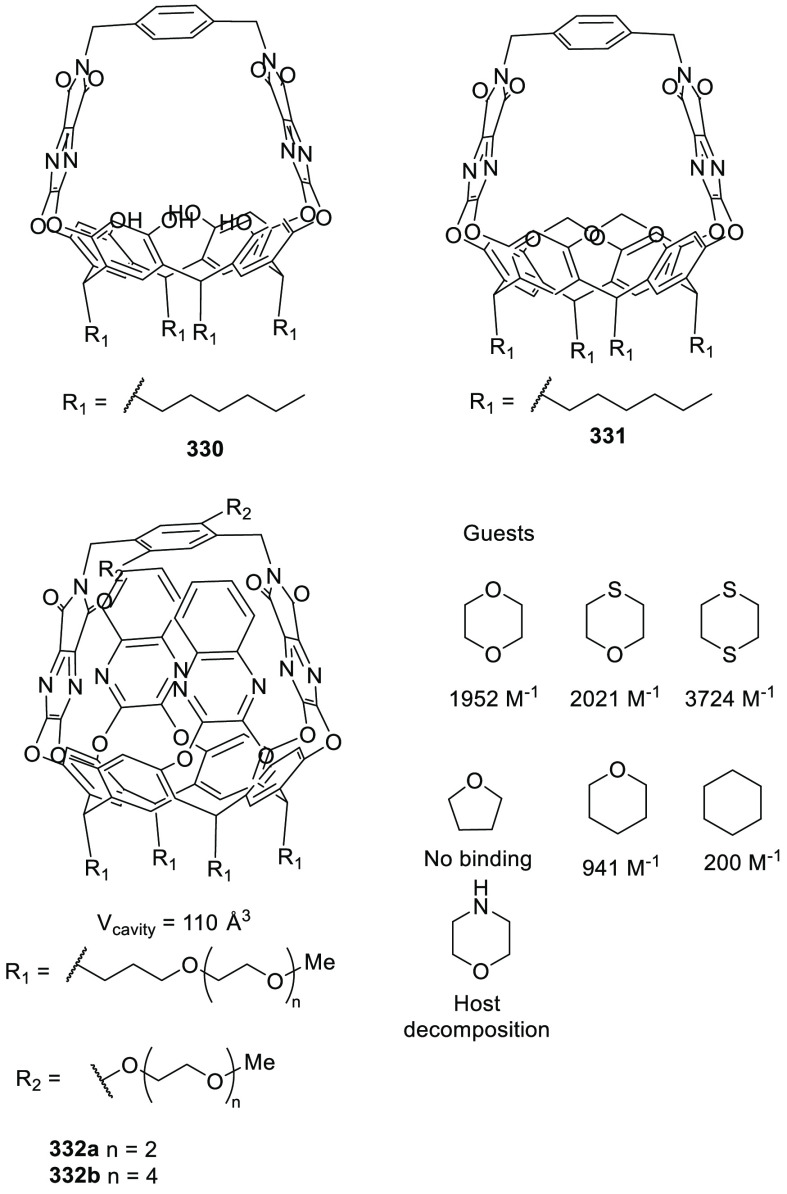
Molecular baskets **330**–**332**. Association constants of **332b** with the guests in D_2_O/CD_3_CN (2:1).^324^

Diederich and co-workers reported the synthesis of molecular baskets with a modification of a cleft-type resorcin[4]arene-based cavitand by introducing rigid bridges to generate **330** and **331** (Figure 134). In addition, the incorporation of PEG groups yielded water-soluble baskets **332a** and **332b**. These host molecules can encapsulate a variety of small heteroalicyclic guests, where dispersion, C–H··· π interactions, and polar interactions (C–O···C = O or S···π) stabilize host–guest complexes. Molecular basket **330** encapsulated the O-containing and S-containing guests in CDCl_3_ with modest *K*_assoc_ values following this tendency: 1,4-dithiane (non binding) < 1,4-thioxane (29 M^–1^) < 1,4-dioxane (43 M^–1^). For water-soluble host **332b**, the same stabilizing interactions described for basket **330** (in addition to solvophobic effects) took place, and the host–guest interaction strength increased. In addition, the S···π interactions significantly contributed and resulted in the reverse tendency of the *K*_assoc_ values in D_2_O/CD_3_CN 2:1: 1,4-dithiane (3724 M^–1^) > 1,4-thioxane (2021 M^–1^) > 1,4-dioxane (1952 M^–1^).^324^

**Figure 135:**
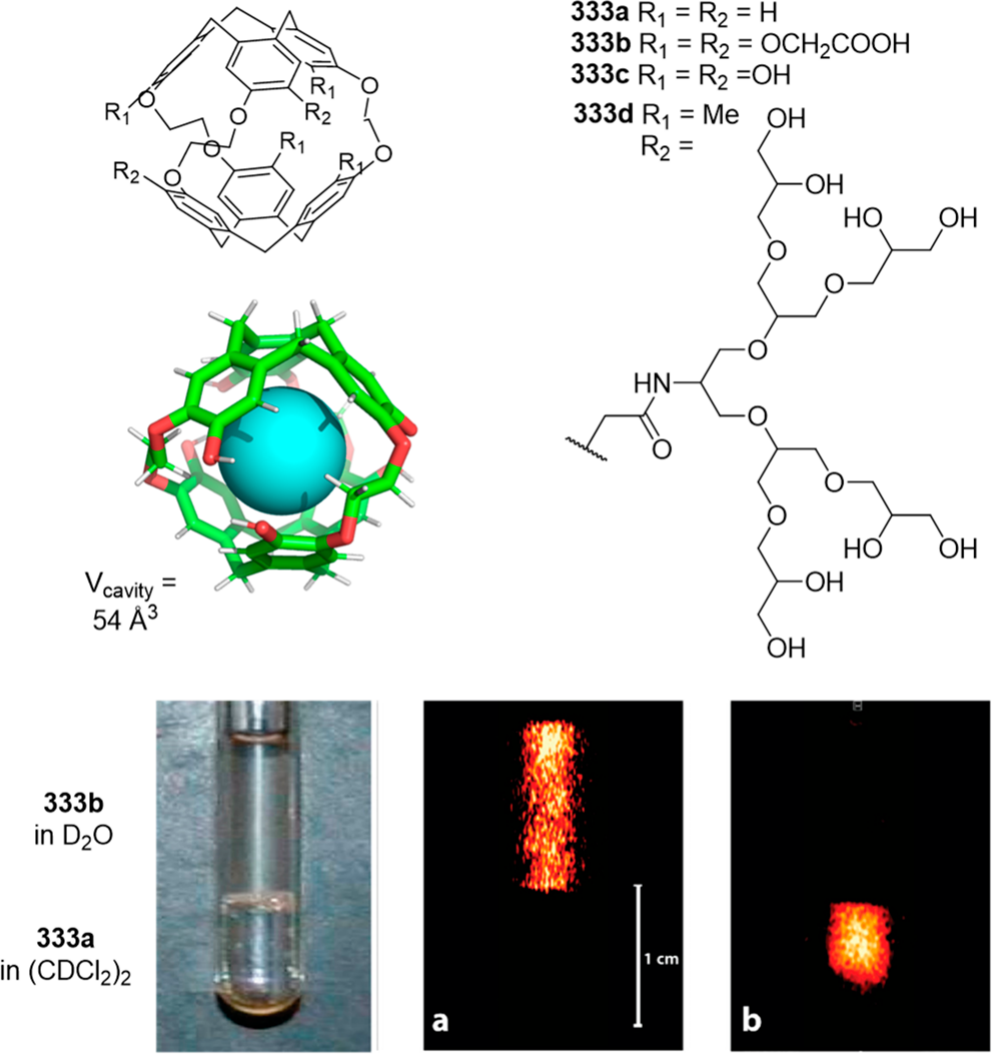
Water-soluble cryptophanes **333** for the encapsulation of xenon by ^129^Xe magnetic resonance imaging.^326,328,129^ Xe images of encapsulated xenon in (a) **333b** in D_2_O and (b) **333a** in (CDCl_2_)_2_.^325^ Adapted with permission from ref (325). Copyright 2008 American Chemical Society.

Berthault and co-workers developed water-soluble cryptophanes **333a**–**333c** for the encapsulation of xenon for ^129^Xe magnetic resonance imaging (Figure 135).^325,326^ To achieve water solubility, these cryptophanes incorporate six carboxylic acids (**333b**) or six phenol groups (**333c**). In addition to solubilizing properties, these groups interact with the environment, such as the local pH or the presence of cations by providing them with sensing properties. Cryptophane **333b** is sensitive to the pH of the surrounding media and displays a change in the ^129^Xe NMR spectra of the encapsulated ^129^Xe (68 ppm at pH below 4.7 and 64 ppm at pH above 4.7), which provides a local pH measurement of around 4.7. For cryptophane **333b**, the xenon exchanging rate is markedly affected by the nature of the counterions present in the media (e.g., Cs^+^, K^+^, Na^+^, or Li^+^), and for Cs^+^, xenon binding is inhibited (Figure 135). In fact the cage **333b** system with Cs^+^ and Tl^+^ in basic media displays association constants as high as 5.3 × 10^8^ M^–1^ for Cs^+^ and 2.9 × 10^9^ M^–1^ for Tl^+^. The high observed association constant for Cs^+^ agrees with the fact that xenon does not enter the cavity in the presence of Cs^+^ by either blocking the entrance to the cavity or occupying the hydrophobic cryptophane cavity.^327^ On the basis of this system, Schröder, Haag, and co-workers incorporated dendronized water-solubilizing groups based on polyglycerol and also used the resulting systems **333d** for ^129^Xe nuclear magnetic imaging (Figure 135). Non-ionic polyglycerol dendrons are well-known for their compatibility in biological applications because they reduce nonspecific binding to biological targets. The cavity of these cryptophanes is extremely hydrophobic with good affinity to Xe atoms, and with *K*_assoc_ = 2750 M^–1^ as determined by ^129^Xe NMR Hyper-CEST experiments (Figure 135).^328^ The authors believe that the dendronization of cryptophanes could be a useful and promising approach to develop new Xe-based biosensors.

**Figure 136:**
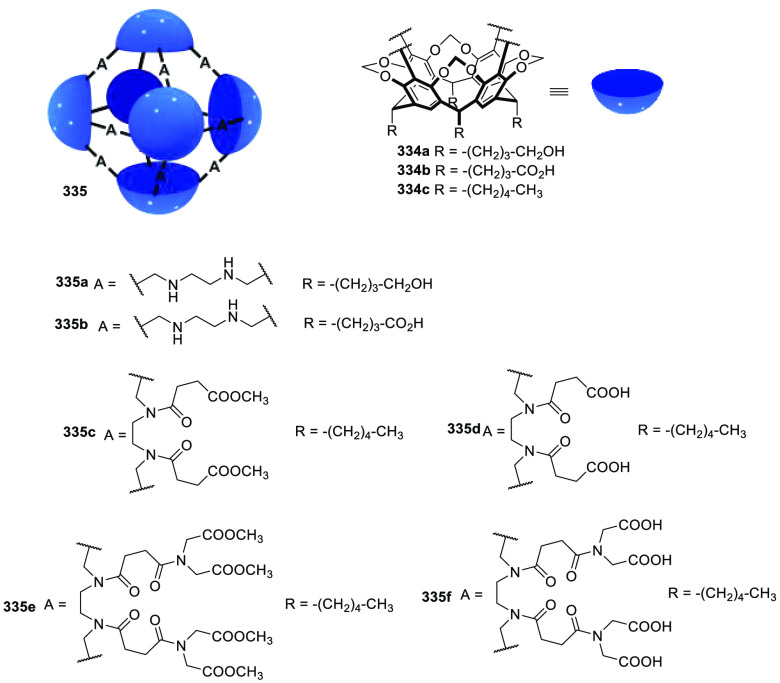
Water-soluble octahedral nanocapsules **335** obtained from cavitands **334**.^329^

Warmuth and co-workers used cavitands **334** to prepare water-soluble octahedral nanocapsules **335a**–**335f** and studied their molecular recognition features using NMR titrations (Figure 136).^329^ Nanocapsules were prepared by the reacting tetraformyl cavitands with several ethylene diamines, followed by reduction with NaBH_4_, to obtain cavities with volumes ranging from 1700 to 2000 Å^3^. NMR studies showed that nanocapsule **335** encapsulated small negatively charged and hydrophobic guests (*p*-toluensulfonic acid, 4-methylumbelliferyl phosphate, and Boc-protected aspartic acid) into the six identical and independent cavitands by forming 1:6 host–guest complexes. **335a** and **335b** also bind with nucleotides (ATP, dAMP, dGMP, and TTP) but on the outside of nanocapsules. The binding behavior of nanocapsules **335c**–**335f** was not studied for its low solubility at a neutral pH and given the formation of aggregates.

**Figure 137:**
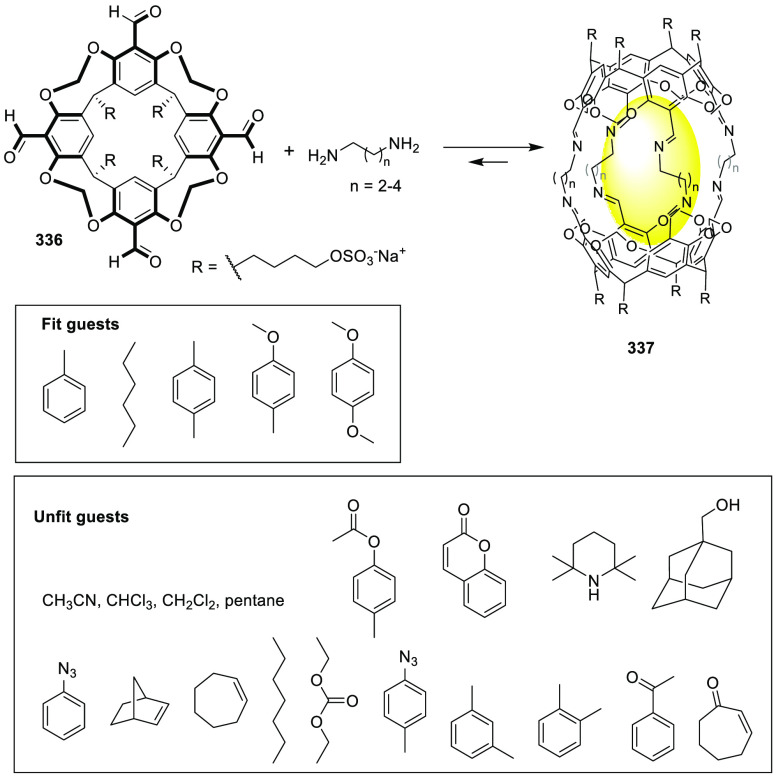
Water-soluble nanocapsules **337**.^330^

The same group used the water-soluble tetraformylcavitand **336** by incorporating R-OSO_3_^–^ water-solubilizing groups to prepare capsule structures **337** upon the reaction with diamines. The thermodynamically controlled reactions of the cavitand with two equivalents of diamines NH_2_(CH_2_)_*n*_NH_2_ (*n* = 2–4) yielded only oligomeric aggregates. However, in the presence of a suitable templating guest (see the fit guests in Figure 137), the corresponding capsule was formed with >90% yield. The guests that did not fit in the cavity (have the wrong shape or too small/large) did not template cage formation. These capsules are dynamic, dissociate upon the addition of acid, and reform upon basification. Guest encapsulation is driven by the hydrophobic effect and capsules are able to exchange guests through the temporary hydrolysis of imine bonds. In nonaqueous solvents, guest exchange is very slow or may not even be possible (Figure 137).^330^

**Figure 138:**
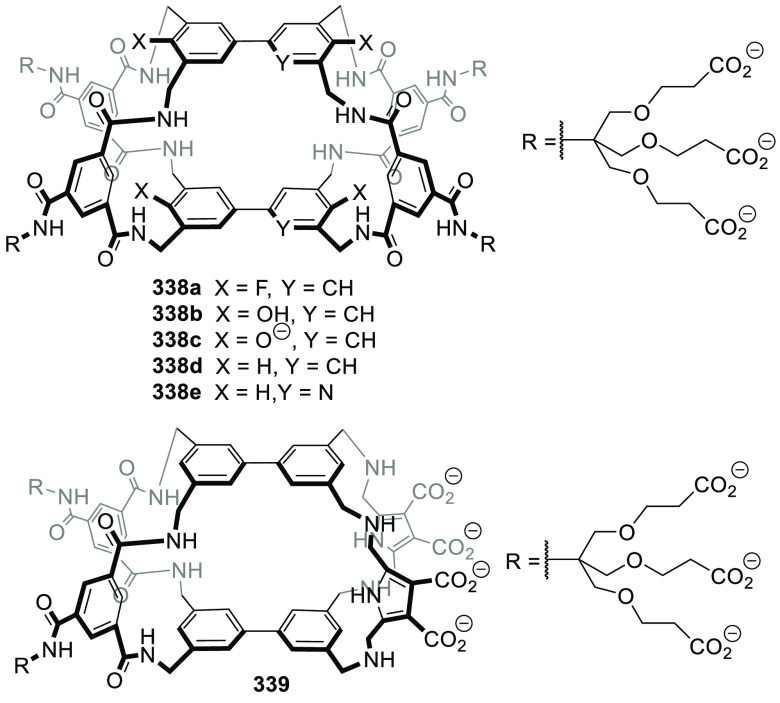
Water-soluble synthetic lectins **338** and **339**.^331−333^

In 2011, Davis and Barwell prepared synthetic lectins **338a**–**338c** and studied their recognition behavior toward carbohydrates (Figure 138).^331^^1^H NMR studies, carried out in water (at pH 7.5 for **338a** and **338b** and at pH 12.0 for **338c**), showed that synthetic lectins bound with carbohydrates (glucose, galactose, mannose, methyl β-d-glucoside, and methyl α-d-glucoside) to form 1:1 inclusion complexes with different strengths (through C–H···π interactions). In line with this, the higher binding constants were for **338b** with all the equatorial carbohydrates glucose and methyl β-d-glucoside. The measured binding constants with cage **338a** were lower because the presence of electron acceptor fluorine atoms decreased the strength of the C–H···π interactions. Lower binding constants were observed with cage **338c** due to the presence of water molecules that cannot be easily displaced in the vicinity of the cavity around phenoxide moieties.

Further modifications yielded cages **338d** and **338e** and also binding carbohydrates in water.^332^ As in previous studies, ^1^H NMR titrations in D_2_O were carried out to measure the stability constants of **338d** with selected carbohydrates (d-glucose, methyl β-d-glucoside, d-mannose, d-galactose, d-xylose, d-lyxose, 2-deoxy-d-glucose, d-ribose, *N*-acetyl-d-glucosamine, d-cellobiose, d-lactose, d-maltose, d-gentiobiose, d-sucrose, and *N*-acetilneuraminic acid). Hexose monosaccharides were bonded with low affinities, and pentoses (i.e, xylose) were slightly more strongly coordinated. However, the higher binding constants were measured for disaccharides d-cellobiose, d-lactose, d-maltose, and d-gentiobiose. In all cases, 1:1 binding inclusion complexes (through C–H···π interactions) were formed. Cage **338e** presented negligible binding constants with all of the tested carbohydrates. These lower affinities could be ascribed to weaker C–H···π interactions due to the electrodeficient character of the pyridyl ring.

The binding of synthetic lectin **339** (Figure 138) to the selected carbohydrates (d-glucose, methyl β-d-glucoside, *N*-acetyl-d-glucosamine, d-mannose, d-galactose, d-xylose, d-lactose) was tested by ^1^H NMR titrations at pD = 13 in D_2_O.^333^ These titrations showed that cage **339** was able to selectively bind with d-glucose to form a 1:1 inclusion complex. Coordination included C–H···π interactions and hydrogen bonding with lateral cage chains.

**Figure 139:**
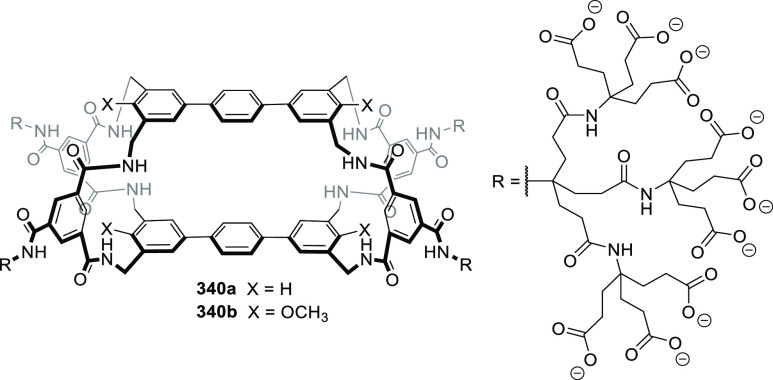
Synthetic lectins **340** that form inclusion complexes with all the equatorial disaccharides.^334^

Increasing the length of aromatic spacers results in a larger cavity to suitably host larger carbohydrates. Tricyclic synthetic lectins **340a** and **340b** (Figure 139) were able to form inclusion complexes with equatorial disaccharides.^334^ Fluorescence titrations, carried out in water with cage **340a** and the selected mono- and disaccharides (methyl β-d-cellobioside, d-cellobiose, d-xylobiose, *N*,*N*′-diacetyl-d-chitobiose, d-lactose, d-maltose, d-gentiobiose, d-trehalose, d-sucrose, d-cellotriose, d-glucose, *N*-acetyl-d-glucosamine, d-galactose) showed the formation of 1:1 inclusion complexes only with d-cellobiose (3140 M^–1^), d-lactose (230 M^–1^), and d-maltose (67 M^–1^). ^1^H NMR and isothermal titration calorimetry (ITC), carried out in water with cage **340b**, and the same guests also led to the formation of 1:1 inclusion complexes for all the saccharides except d-trehalose, d-sucrose, and d-galactose. The higher association constants with cage **340b** were measured for methyl β-d-cellobioside (4500 M^–1^) and d-cellobiose (3340 M^–1^). The disaccharide/monosaccharide selectivity achieved with cage **340b** was remarkable and amounted to 1300:1 when comparing the association constants for d-cellobiose and d-glucose. NOESY experiments, carried out with **340a** and methyl β-d-cellobioside, showed that disaccharide entered the cage cavity through C–H···π hydrophobic interactions and hydrogen bonding.

**Figure 140:**
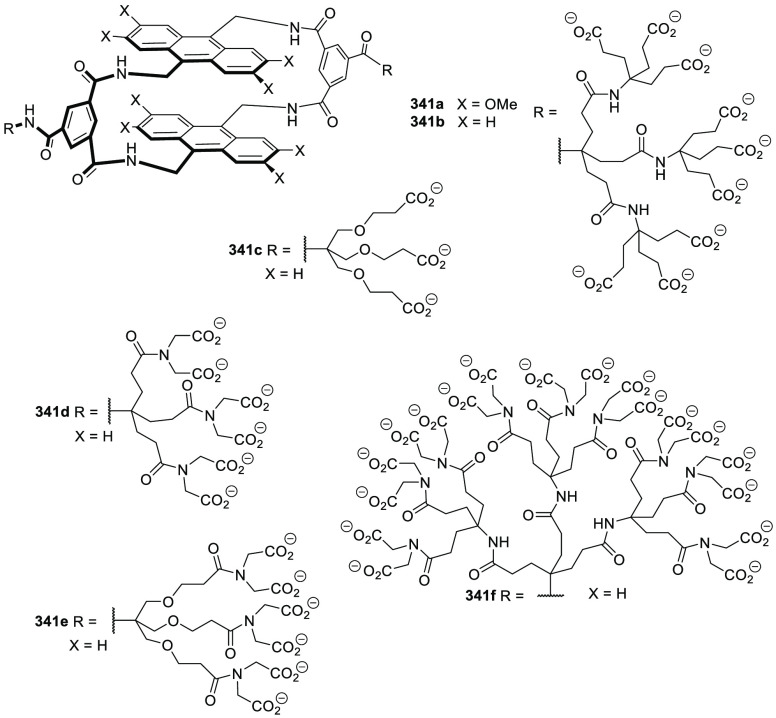
Synthetic lectins **341** for binding carbohydrates.^335,336^

By preserving a similar core structure, Davis and co-workers prepared synthetic lectin **341a**, which was able to recognize maltodextrin among several mono- and oligosaccharides (Figure 140).^335^^1^H NMR titrations, carried out in D_2_O, using **341a** and several mono- and oligosaccharides (d-glucose, methyl β-d-glucoside, methyl α-d-glucoside, *N*-acetyl-d-glucosamine, methyl β-d-*N*-acetylglucosaminide, d-galactose, d-mannose, d-cellobiose, d-cellotriose, d-cellotetraose, d-lactose, d-maltose, d-maltotriose, d-maltotetraose), showed the formation of 1:1 inclusion complexes. However, cage **341a** displayed marked selectivity toward α-linked maltodextrins (with association constants of 580, 1150, and 1620 M^–1^ for d-maltose, d-maltotriose, and d-maltotetraose, respectively) over the other tested saccharides. This selectivity pattern was ascribed to the presence of the eight methoxy groups located in the anthracene moieties of cage **341a**.

Davis and co-workers also prepared synthetic lectins **341b**–**341f** (Figure 140) with different side chains and studied their effect on the affinity binding of carbohydrates (d-glucosamine, d-galactosamine, d-glucose, methyl β-d-glucoside, *N*-acetyl-d-glucosamine, d-galactose, d-mannose).^336^^1^H NMR and fluorescence titrations, carried out with all of the cages and the selected saccharides in water, showed the formation of 1:1 inclusion complexes (through C–H···π hydrophobic interactions and hydrogen bonding). The higher association constants were measured for d-glucosamine for all five cages (160, 1400, 2000, 2400, and 7000 M^–1^ for **341c**, **341d**, **341e**, **341b**, and **341f**, respectively). As seen for the association constants for d-glucosamine, as the dendrimer in the side chain expanded, the affinities for guests increased. NOESY experiments, carried out with cage **341f** and d-glucosamine, indicated that both side chains contributed to guest binding through strain-free salt bridges (d-glucosamine was positively charged at a neutral pH, whereas dendrimers were negatively charged).

**Figure 141:**
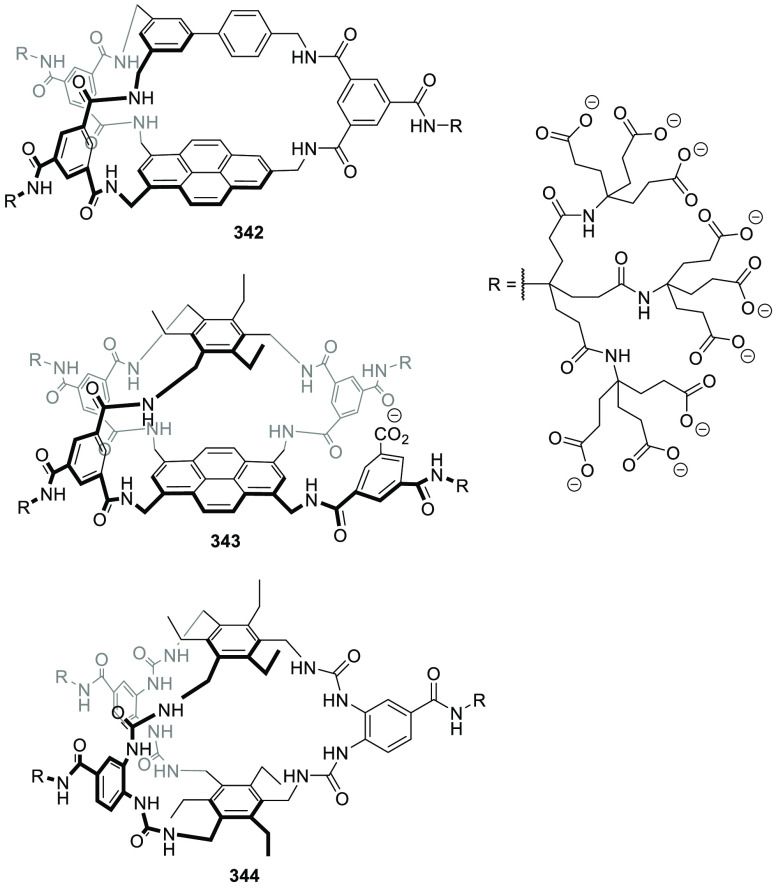
Synthetic lectins **342**, **343**, and **344** containing three connecting bridges.^337−339^

Synthetic lectin **342** (Figure 141), composed of two different aromatic components (pyrene and biphenyl), also forms inclusion complexes with carbohydrates.^337^^1^H NMR titrations of **342** with several carbohydrates (d-mannose, d-galactose, cellobiose, methyl β-d-glucoside, and methyl β-d-*N*-acetylglucosaminide) carried out in D_2_O showed the formation of 1:1 inclusion complexes (through hydrogen bonding and C–H···π interactions). Of all the tested carbohydrates, the stronger association constant was measured for cellobiose (260 M^–1^).

In another work, Davis and co-workers also prepared synthetic lectin **343** for the enantioselective recognition of carbohydrates (Figure 141).^338^ After the synthesis of cage **343**, attempts to separate both enantiomers were unsuccessful. However, binding ^1^H NMR studies in D_2_O with selected carbohydrates (*N*-acetyl-d-glucosamine, d-glucose, methyl β-d-glucoside, d-mannose, l-mannose) were carried out with the racemate, and the coordination features of both enantiomers were quite different. The best enantiomeric discrimination was observed for *N*-acetyl-d-glucosamine, for which stability constants of 1280 and 81 M^–1^ for the formation of 1:1 stoichiometry diastereomeric complexes (enantioselectivity of 16:1) were found.

Biomimetic synthetic lectin **344** (Figure 141) was prepared. Its coordination behavior toward carbohydrates (d-glucose, methyl β-d-glucoside, methyl a-d-glucoside, d-glucuronic acid, d-xylose, 2-deoxy-d-glucose, d-galactose, d-mannose, d-ribose, d-fructose, d-cellobiose, *N*-acetyl-d-glucosamine, d-maltose, l-fucose, d-gluconic acid) and other biomolecules (d-mannitol, ascorbic acid, uracil, uric acid, cytosine, adenosine, l-phenylalanine, l-tryptophan, paracetamol) was tested in water using ^1^H NMR and ITC titrations.^339^ Of all the tested carbohydrates and biomolecules, the strongest inclusion complex (1:1 stoichiometry) was formed between cage **344** and d-glucose with an affinity constant of ca. 18000 M^–1^. The all-equatorial d-glucose bound with **344** by forming hydrogen bonds with the six urea groups on the side chains and through C–H···π hydrophobic interactions. Besides, methyl β-d-glucoside, glucuronic acid, and xylose (with pyranose structures and all-equatorial substitution patterns) showed affinities >5000 M^–1^. Finally, other tested carbohydrates were bound ca. 100-fold more weakly than d-glucose, while biomolecules did not form inclusion complexes with **344**. Finally, cage **344** was used for d-glucose detection in the presence of l-glucose in complex biological settings (prefiltered human serum, free glucose cell culture medium, beer with an artificially reduced glucose concentration) using circular dichroism.^340^

**Figure 142:**
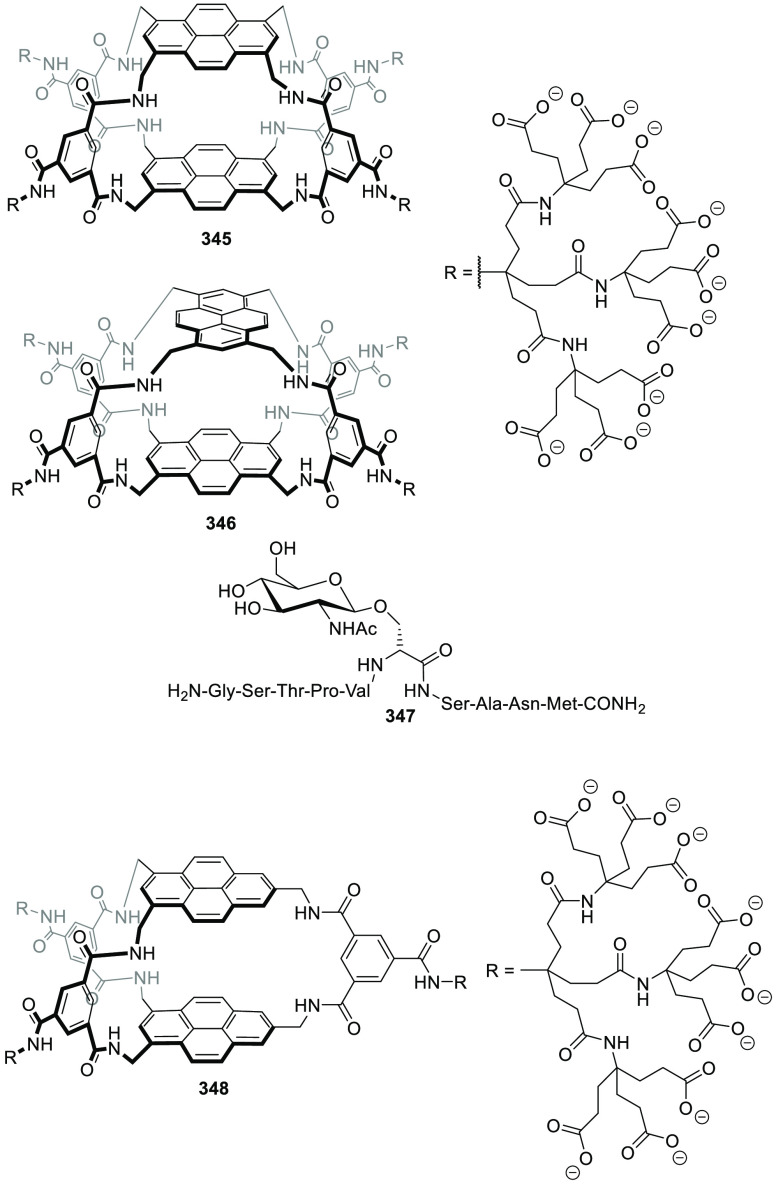
Pyrene-containing synthetic lectins **345**, **346**, and **348**.^341,342^

Davis and co-workers prepared pyrene-containing synthetic lectins **345** and **346** capable of forming strong inclusion complexes with *N*-acetylglucosamine derivatives in water (Figure 142).^341^^1^H NMR studies, carried out in D_2_O, showed that eclipsed receptor **345** only bound with methyl *N*-acetyl-β-d-glucosaminide to form a 1:1 host–guest complex with an association constant of 2100 M^–1^. However, staggered receptor **346** formed 1:1 host–guest inclusion complexes with *N*-acetyl-β*-*d-glucosaminide, *N*-acetyl-a-d-glucosaminide, *N*-acetyl-d-glucosamine, methyl, β-d-glucoside, and d-glucose, but with a marked selectivity toward *N*-acetyl-β-d-glucosaminide (association constant of 18200 M^–1^). NOESY, TOCSY, and COSY studies showed that carbohydrates were sandwiched between pyrene units through C–H···π interactions and formed hydrogen bonds with the amide moieties on side chains. Besides, cage **345** was able to bind glycopeptide **347** with a remarkable high association constant of 67000 M^–1^.

Davis and co-workers prepared cage **348** and studied the formation of inclusion complexes with polysaccharides in water (Figure 142).^342^^1^H NMR titrations, carried out in D_2_O with **348**, monosaccharides (d-mannose, d-galactose, d-glucose, methyl β-d-glucoside, methyl *N*-acetyl-β-d-glucosaminide), and polysaccharides (d-cellobiose, d-cellotriose, d-cellotetraose, d-cellopentaose, d-cellohexaose, *N*.*N*′-diacetyl-d-chitobiose, *N*.*N*′,*N*′′-triacetyl-d-chitobiose, d-chitobiose), indicated the formation of weak 1:1 inclusion complexes with monosaccharides and the formation of 1:1 threaded complexes with polysaccharides with affinities within the 3600–19000 M^–1^ range.

**Figure 143:**
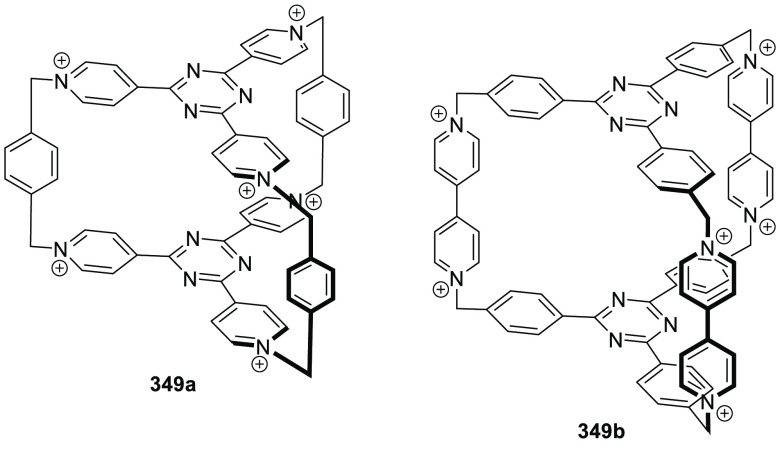
Chemical structure of hexacationic organic cages **349**.^343,344^

Introducing multiple positive charges into the cage structure is another strategy to achieve water solubility. Stoddart and co-workers prepared hexacationic BlueCage·6PF_6_**349a**, which contained six pyridinium rings fused with two central electron-deficient triazines, bridged by three *p*-xylene units (Figure 143).^343^ The six PF_6_^–^ counterions interacted with BlueCage^6+^ through anion−π interaction, and one of them was located inside the cavity cage. BlueCage·6PF_6_**349a** bound pyrene in acetonitrile with a *K*_assoc_ value of 4.93 × 10^5^ M^–1^, whereas, upon changing the counterion from hexafluorophosphate to the bulkier tetrakis(3,5-bis(trifluoromethylphenyl)borate, *K*_assoc_ was increased to 3.95 × 10^6^ M^–1^. This enhancement in pyrene coordination was ascribed to the fact that all the bulky tetrakis(3,5-bis(trifluoromethylphenyl)borate counterions were located outside of the cage cavity, which facilitates guest binding through π–π stacking interactions. Hexacationic triangular covalent organic cage **349b** presented a rigid cavity with an inner distance between the two 2,4,6-triphenyl-1,3,5-triazine groups of 10.97 Å, whereas the distance between two bipyridinium moieties was around 11.60 Å (Figure 143).^344^^1^H NMR and 2D DOSY studies, carried out in D_2_O, showed that the inner cavity of **349b** was able to accommodate two pyrene-1-carbaldehyde molecules. Both aromatic guests underwent π–π interactions with the 2,4,6-triphenyl-1,3,5-triazine groups in an A-D-D-A (A = acceptor, D = donor) fashion. Besides, there were dipole–cation or dipole–dipole interactions between the formyl group of the guest and the pyridinium rings in the spacers of the cage. Cage **349b** was able to accommodate 1,5-bis[2-(2-(2-hydroxyethoxy)ethoxy)ethoxy)ethoxy]naphthalene in water by forming a 1:1 inclusion complex, in which a naphthalene moiety was located inside the cage cavity as a consequence of a strong hydrophobic effect.

**Figure 144:**
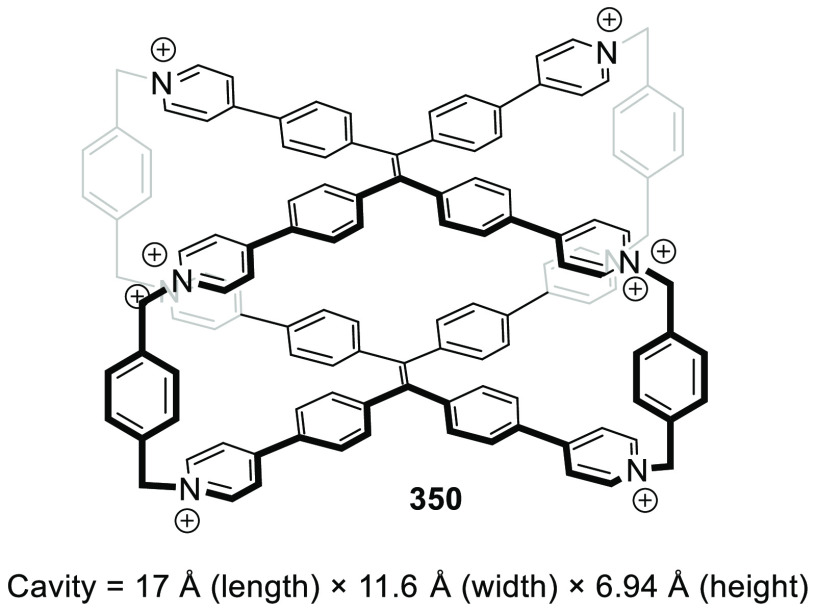
Tetraphenylethene-based octacationic cage **350**.^345^

Cao and co-workers reported the synthesis and complexation behavior of tetraphenylethene-based octacationic cage **350** (Figure 144).^345^ The X-ray crystal structure of **350**·8PF_6_ showed the presence of a cuboid internal cavity of 17 Å (length) × 11.6 Å (width) × 6.94 Å (height). ^1^H NMR and DOSY studies in CD_3_CN, showed that cage **350**·8PF_6_ formed a 1:1 inclusion complex with coronene through multiple C–H···π interactions (between the hydrogen atoms of the tetraphenylethene benzene rings and the guest) and sandwich-type π–π interactions (between the π-electron deficient pyridinium rings and the π-electron-rich aromatic guest). Besides, **350**·8Cl showed remarkable coronene adsorption features, i.e., a solid sample of **350**·8Cl was able to extract coronene from an organic solution. Water-soluble **350**·8Cl was able to form a 1:1 inclusion complex with sulforhodamine 101 as the ^1^H NMR, NOESY, and ITC measurements demonstrated. The formation of inclusion complexes was also followed by fluorescence titrations. Indeed, the broad emission band of **350**·8Cl in water at 545 nm (excitation at 410 nm) was progressively quenched, and a new emission at 621 nm appeared after the progressive addition of sulforhodamine 101 as a consequence of inclusion complex formation.

**Figure 145:**
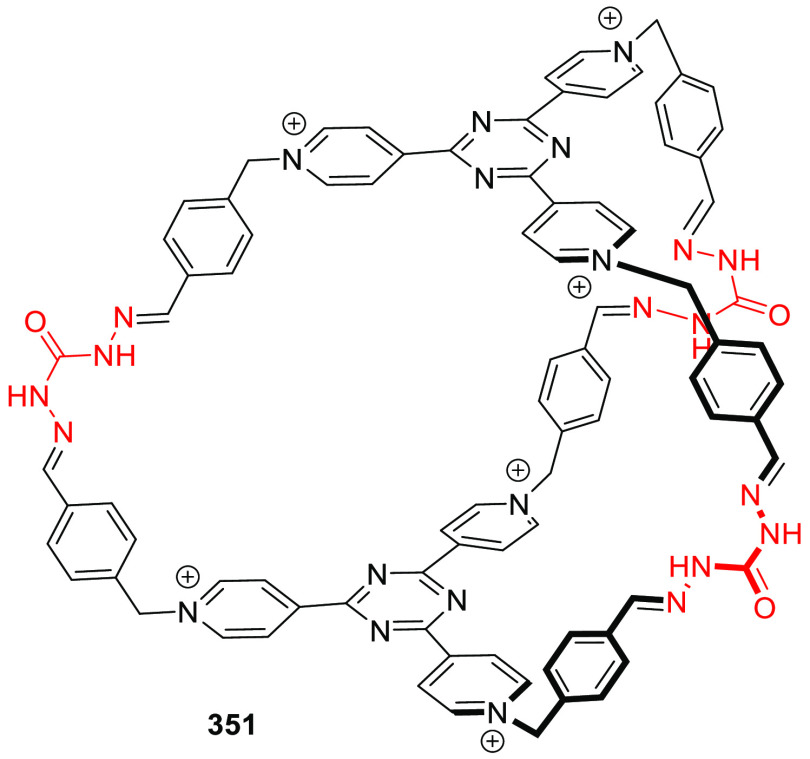
Water-soluble hydrazone cage **351**.^346^

Li, Yang, and co-workers prepared organic prismatic cage **351** by self-assembly in water at 80 °C of an aldehyde derivative and a carbonyl dihydrazine through the formation of hydrazone bonds (Figure 145).^346^ NMR studies showed that this cage was able to form 1:2 cage–guest inclusion complexes with 1,5-dihydroxynaphthalene derivatives. Besides, the addition of naphthalene diimide derivatives yielded complexes in which one molecule of the dihydroxynaphthalene derivative and another of the naphthalene diimide derivatives (through charge-transfer interactions) were located inside the cage cavity.

**Figure 146:**
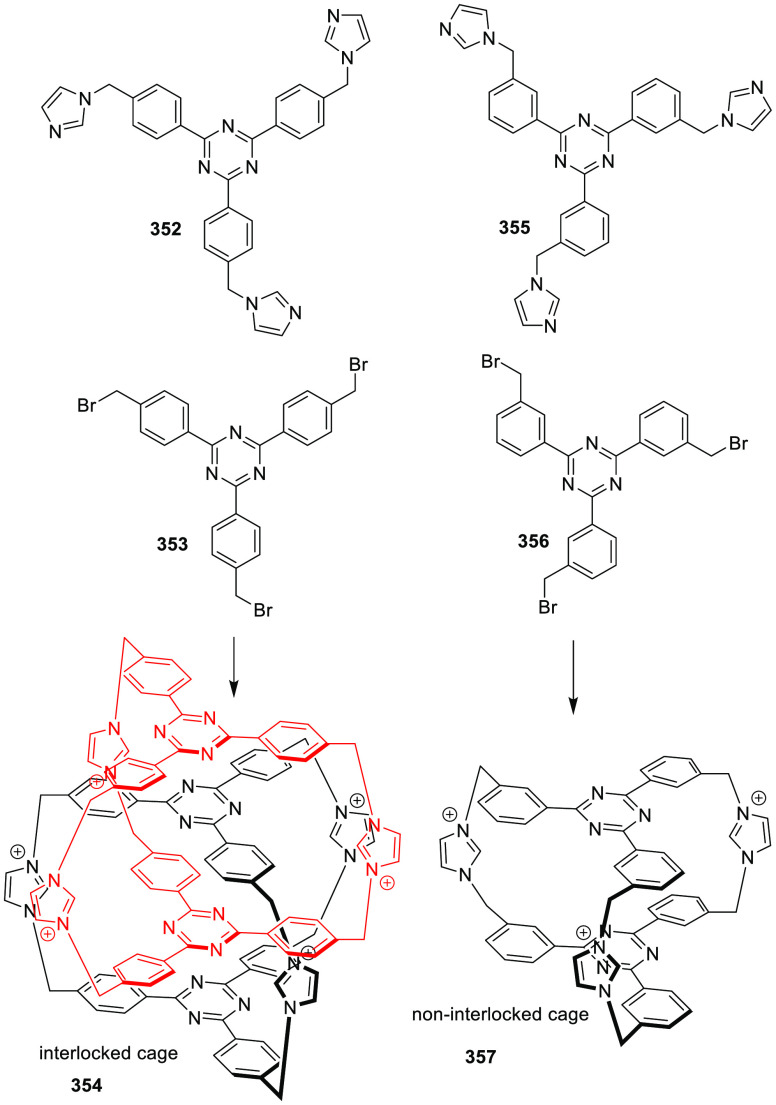
Preparation of cages **354** and **357**.^347^

When equimolar amounts of **352** and **353** in chloroform–acetonitrile 4:1 v/v were reacted for 6–8 h, a white precipitate appeared. It was assigned to the formation of water-soluble interlocked cage **354** (Figure 146).^347^ The DFT calculations showed that the π–π interactions between triazine cores (separated by ca. 3.4 Å) stabilized the interlocked structure. The reaction (in the same solvent) of equimolar amounts of **355** and **356** yielded water-soluble non-interlocked cage **357**. Meta isomers were unable to form an interlocked cage because the benzene rings connected to the triazine core rotated due to the steric hindrance between benzene and imidazolium hydrogens, as demonstrated by the DFT calculations. This loss of planarity of benzene rings twists triazine cores and leads to unfavorable π–π interactions.

**Figure 147:**
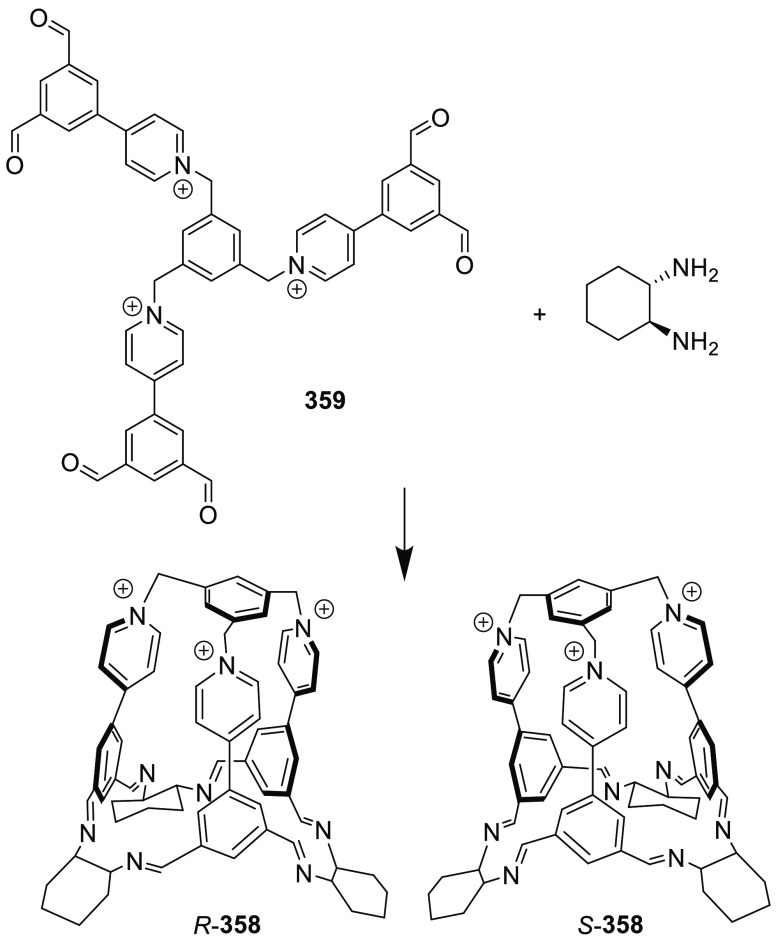
Synthesis of water-soluble cages **358**.^348^

By the reaction of **359** with (1*S*,2*S*)-(−)-1,2-diaminocyclohexane or (1*R*,2*R*)-(−)-1,2-diaminocyclohexane at a 1:3 ratio in water at 80 °C, Li and co-workers prepared basket-shaped tris-cationic chiral cages *S*-**358** and *R*-**358** (Figure 147).^348^ Both cages did not decompose in water and remained kinetically inert in the presence of competitive amines (ethanolamine). These authors ascribed this stability to multivalence (aldehyde and amino precursor are connected through six imine bonds) and the preorganization of 1,2-diaminocyclohexane (imino units are immobilized in gauche positions). The ability of cage *S*-**358** to recognize the selected guests was studied by ^1^H NMR in D_2_O. Cage *S*-**358** formed 1:1 inclusion complexes with ethane, pentane, ethylene, 1-chloropentane, 1-bromopentane, 1-bromohexane. 1,2-dichloroethane, 1,5-dichloropentane, 1,2-dibromoethane, 1,3-dibromopropane, 1,4-dibromobutane, and 1,5-dibromopentane. The formation of inclusion complexes is ascribed to the dipole-cation and C–H···π hydrophobic interactions between methylene protons in guests and the phenyl moieties on the cage. Besides, cage *S*-**358** was able to preferentially accommodate (*R*)-1,2-epoxibutane (association constant of 35 M^–1^) compared to (*S*)-1,2-epoxibutane (association constant was too small to be accurately determined). The values of the association constants for (*S*)-(−)-propylene oxide and (*R*)-(−)-propylene oxide with cage *S*-**358** were 11 and 14 M^–1^, respectively.

**Figure 148:**
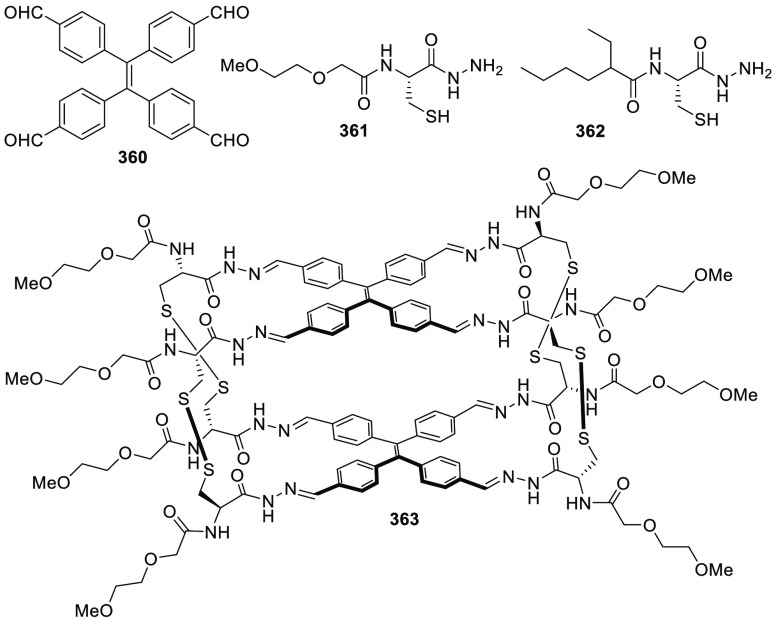
Self-assembled fluorescent aromatic cages **363**.^349^

Stefankiewicz and co-workers prepared self-assembled fluorescent aromatic cages using tetraphenylethylene tetraaldehyde (**360**) and two cysteine hydrazides functionalized with hydrophilic (**361**) and hydrophobic (**362**) chains (Figure 148).^349^ LC-MS experiments, carried out in water (or in water–DMSO solutions) and, with a mixture of **360** and **361**, showed the formation of cage **363** after 3 days at 50 °C. The same results, namely the formation of a similar, yet hydrophobic, cage, were obtained after 3 days at 50 °C for a mixture of **360** and **362** in chloroform–DMSO (9:1 v/v). The ^1^H NMR studies also confirmed the formation of both cages. The authors reported that hydrophilic cage **363** had a volume cavity of 4200 Å^3^, whereas this value for its hydrophobic counterpart came to 3200 Å^3^. Besides, the solutions of both cages presented broad emission bands at ca. 510 nm (excitation at 320 nm), which were ascribed to the presence of the tetraphenylethylene fluorophore.

**Figure 149:**
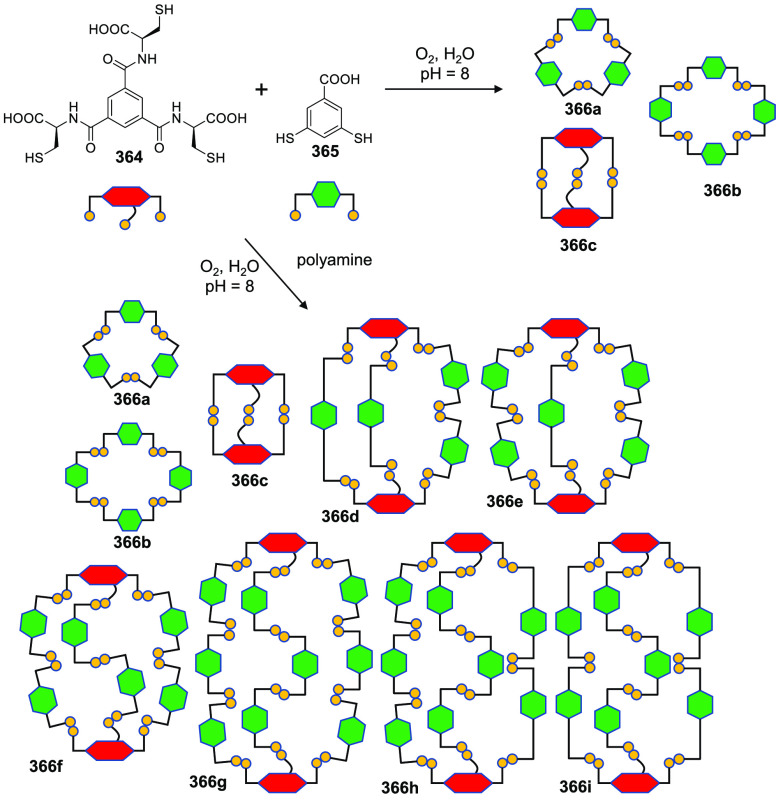
Dynamic combinatorial library yielding water-soluble cages **366**.^350^

Sanders and co-workers used dynamic combinatorial chemistry for the template synthesis of water-soluble organic cages.^350^ The air oxidation of the solutions of **364** and **365** (1:2 molar ratio) in water at pH 8.0 for 7 days yielded a mixture of cyclic trimer **366a** (26%), the tetramer of **366b** (39%), and the dimeric capsule of **366c** (35%), in which both compounds (**364** and **365**) were bound through disulfide bonds. Larger architectures (**366d**–**366i**) were obtained using polyamines as templates under the same experimental conditions (**364** and **365** at a 1:2 molar ratio, water at pH 8.0, and a 7-day reaction). Using spermine, six different cage structures **366d**–**366i** were generated and characterized using LC-MS. The most abundant cage **366i** was formed by two molecules of **364** and seven of **365**. The yields of the different formed cages could be modulated by using other linear polyamines, such as 1,4-butanediamine, spermidine, and triethylenetetramine (Figure 149).

**Figure 150:**
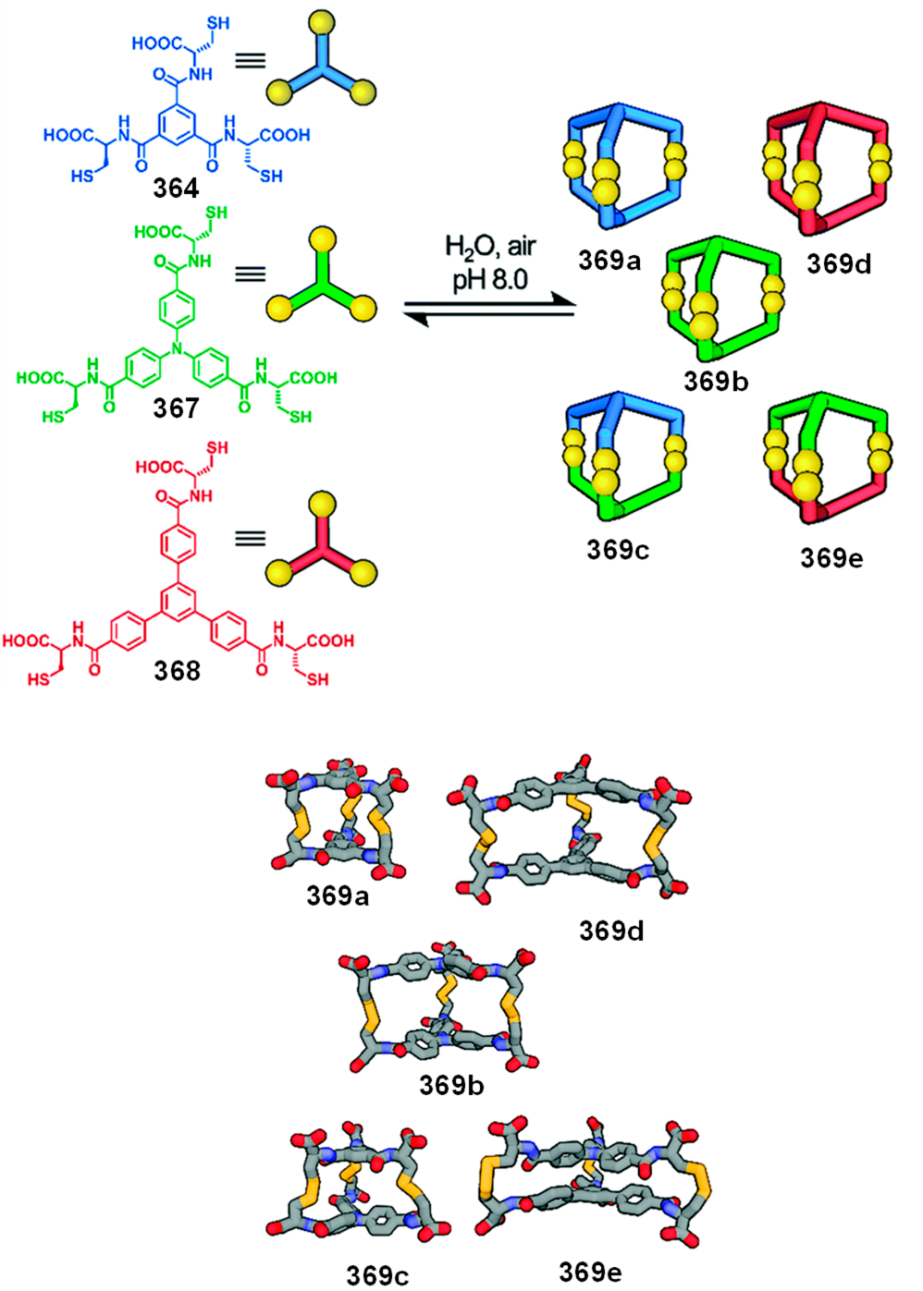
Water-soluble heterodimeric disulfide cages **369**.^351^ Adapted with permission from ref (351). Copyright 2021 the authors of the original publication. Published by the Royal Society of Chemistry with the Creative Commons CC BY license http://creativecommons.org/licenses/by/3.0/.

With a similar approach, Stefankiewicz and co-workers used pseudopeptide trithiol precursors **364**, **367**, and **368** to prepare water-soluble pseudopeptide molecular cages **369a**–**369e** based on disulfide bonds. Precursors **364**, **367**, and **368** come in different sizes (small, medium, large), and the self-assembly reaction shows a degree of self-sorting related to the size of the trithiol. In a mixture containing small and large precursors (**364** and **368**), the reaction self-sorts into a pair of two homodimeric cages (**369a** and **369d**). Besides, when the precursors of three different sizes are used (**364**, **367**, and **368**), the expected homodimeric cages form (**369a**, **369b**, and **369d**), as well with small-medium (**369c**) and medium-large (**369d**) heterodimeric cages (Figure 150).^351^ The developed cages display good stability in aqueous media, which opens the door of these hosts for drug delivery applications.

## 5 Conclusions and Perspectives

This review offers an in-depth view of purely organic cages, including hemicarcerands, carcerands, cavitands, capsules, and cages. The systems described throughout the review show the wide scope achieved in this field by allowing preparation of molecules with cavities ranging from small to large volumes. In addition to size, flexibility/rigidity, size of windows, hydrophobicity, etc., all play a key role in defining their properties. From the synthetic point of view, clearly the self-assembly of cage molecules through reversible bonds is the most widespread methodology because its efficiency and versatility surpass the limitations of irreversible bond formation, which does not allow the correction of the mistakes made during the self-assembly reaction pathway.

Imine cages dominate synthetic strategies due to their very efficient synthesis but have limitations, such as their stability, especially in aqueous environments. There is no doubt that using other bond formation strategies, which are presently minor, will contribute to extend the synthetic toolbox to prepare cages with custom properties. Indeed, the development of organic chemistry, particularly robust reversible reactions, will contribute to develop cage structures with properties that cannot be achieved with current synthetic methods.

Although most organic cages are inherently insoluble in water because of their hydrophobic nature, the addition of water-solubilizing groups allows water solubility to be accomplished. Such groups mainly contain positive or negative charges, although long polyethylene glycol chains also enable water solubility. By this strategy, the cage’s hydrophobic nature is retained, which allows encapsulating hydrophobic guests with high affinity in stabilized water and other noncovalent interactions.

Despite the vast progress made in the area of purely covalent molecular cages and containers, this field is clearly extending, and significant advances can be expected in the near future. We envisage that the field will continue to grow and offer structures with new features to improve efficiency in different applications, including synthesis, gas separation, gas storage, biomedical applications, etc. Finally, developing computational tools to predict the outcome of a cage formation reaction from the selected building blocks, as well as cage properties, is another essential objective to accelerate the discovery of cages with customized properties.

The fine-tuning of cage structures will allow the numerous functions reported to date to extend. Cages with specific cavities will permit materials to be prepared for gas separation by targeting gases that cannot be operated in today’s state of the art and will achieve high absorption efficiency and selectivity in gas mixtures. Cages with big cavities, including water-soluble cages with hydrophobic cavities, will allow further guests to be encapsulated, chemical reactions in its cavity to be performed, or they can be used as delivery systems.
